# CRIP1 Reshapes the Gastric Cancer Microenvironment to Facilitate Development of Lymphatic Metastasis

**DOI:** 10.1002/advs.202303246

**Published:** 2023-07-06

**Authors:** Zhonghua Wu, Bicheng Qu, Minxian Yuan, Jingjing Liu, Cen Zhou, Mingwei Sun, Zhexu Guo, Yaqing Zhang, Yongxi Song, Zhenning Wang

**Affiliations:** ^1^ Department of Surgical Oncology and General Surgery The First Hospital of China Medical University Key Laboratory of Precision Diagnosis and Treatment of Gastrointestinal Tumors China Medical University Ministry of Education 155 North Nanjing Street, Heping District Shenyang 110001 China; ^2^ Institute of Health Sciences China Medical University Shenyang Liaoning 110122 China

**Keywords:** CRIP1, gastric cancer, lymphangiogenesis, lymphatic metastasis

## Abstract

Lymphangiogenesis in tumors provides an auxiliary route for cancer cell invasion to drainage lymph nodes, facilitating the development of lymphatic metastasis (LM). However, the mechanisms governing tumor lymphangiogenesis and lymphatic permeability in gastric cancer (GC) remain largely unknown. Here, the unprecedented role and mechanism of cysteine‐rich intestinal protein‐1 (CRIP1) in mediating the development of GC LM is uncovered. A series of assays are performed to identify downstream targets of CRIP1, and rescue experiments are performed to confirm the effects of this regulatory axis on LM. CRIP1 overexpression facilitates LM in GC by promoting lymphangiogenesis and lymphatic vessel permeability. CRIP1 promotes phosphorylation of cAMP responsive element binding protein 1(CREB1), which then mediates vascular endothelial growth factor C (VEGFC) expression necessary for CRIP1‐induced lymphangiogenesis and transcriptionally promotes C‐C motif chemokine ligand 5 (CCL5) expression. CCL5 recruits macrophages to promote tumor necrosis factor alpha (TNF‐*α*) secretion, eventually enhancing lymphatic permeability. The study highlights CRIP1 regulates the tumor microenvironment to promote lymphangiogenesis and LM in GC. Considering the current limited understanding of LM development in GC, these pathways provide potential targets for future therapeutics.

## Graphical abstract

Cysteine‐rich intestinal protein‐1 (CRIP1) can interact with the cAMP responsive element binding protein 1 (CREB1) and enhance CREB1‐mediated VEGFC and CCL5 transcription. Secretion of VEGFC can induce lymphangiogenesis, while increased CCL5 in tumor microenvironment can form a concentration gradient to recruit M2 TAM, then promote TNF‐*α* secretion to increase lymphatic permeability. Through the reciprocal effects of VEGFC and CCL5, CRIP1 can promote lymphangiogenesis and lymphatic metastasis development in gastric cancer.

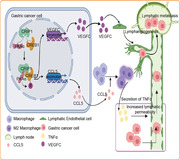

## 1 Introduction

Gastric cancer (GC) is diagnosed in ≈1 million people annually, making it one of the most common malignancies worldwide.^[^ ^1^ ^]^ Although diagnostic and therapeutic modalities have greatly improved over the past decades, the prognosis of patients with advanced GC remains poor, with overall survival ranking around 40%.^[^ ^2^ ^]^ Lymphatic metastasis (LM), the most common form of GC metastasis, occurs in early GC as well as in advanced GC, influencing the prognosis and therapeutic decision‐making for GC.^[^ ^3^ ^]^ However, the mechanisms governing tumor lymphangiogenesis and LM development in GC have rarely been explored.

Lymphangiogenesis in tumors mainly refers to the process of generating new lymphatic vessels based on coordination of the tumor microenvironment and lymphangiogenic growth factors. As a cutting edge frontier in biological research, lymphangiogenesis is gradually regarded as a crucial hallmark of cancer since its excessive activation or dysfunction contributes to cancer metastasis.^[^ ^4^, ^5^, ^6^ ^]^ Many factors affect lymphangiogenesis, including the vascular endothelial growh factor (VEGF)‐vascular endothelial growh factor receptor (VEGFR) pathway,^[^ ^7^, ^8^ ^]^ the angiopoietin system,^[^ ^9^ ^]^ and hepatocyte growth factor.^[^ ^10^ ^]^ Although studies have shown that some lymphangiogenic growth factors have the potential to predict prognosis,^[^ ^11^ ^]^ a deeper understanding of the mechanism of lymphangiogenesis and LM in GC is important for diagnosis and early treatment as well as for prolonging survival time. Lymphatic permeability is an important restriction for cancer cell intravasation through lymph vessels and then formation of lymph node metastasis. The permeability is closely associated with the organization of intercellular junctions which are formed by the homotypic interaction of vascular endothelial cadherin (VE‐cadherin). In recent years, tumor‐associated macrophages (TAM) have been shown to promote lymphoinvasion and LM in cancer,^[^ ^12^, ^13^ ^]^ indicating TAM may alter the lymphatic permeability to influence LM formation.^[^ ^14^ ^]^ However, the detailed mechanism by which TAM regulate lymphatic permeability remains unclear.

Cysteine‐rich intestinal protein 1 (CRIP1), a member of the LIM/double zinc‐finger protein family, has been shown to be elevated in various tumors.^[^ ^15^ ^]^ CRIP1 can activate the Wnt/*β*‐catenin pathway to promote cervical cancer cell invasion and metastasis.^[^ ^16^ ^]^ In colorectal cancer, CRIP1 promotes ubiquitination and degradation of Fas protein to reduce the chemosensitivity of tumor cells to 5‐Fluorouracil.^[^ ^17^ ^]^ Recent studies have shown that CRIP1 promotes homologous repair upon DNA damage and attenuates the efficacy of chemotherapy in GC.^[^ ^18^ ^]^ In addition, previous studies^[^ ^19^, ^20^, ^21^ ^]^ also reported the positive relationship between CRIP1 upregulation and LM in clinical cancer cohort. However, the mechanism by which CRIP1 acts in lymphangiogenesis and LM in GC has not yet been reported and requires further exploration.

In this study, we aimed to characterize the role and mechanisms of CRIP1 in promoting lymphangiogenesis and lymphatic permeability in GC. Using a series of in vitro experiments, in vivo animal models, liquid chromatography–tandem mass spectrometry (LC–MS/MS), and chromatin immunoprecipitation (ChIP), we show that CRIP1 can reshape the tumor microenvironment by increasing secretion of vascular endothelial growth factor C (VEGFC) and C‐C motif chemokine ligand 5 (CCL5) from GC cells. We demonstrate that CRIP1‐mediated VEGFC can promote lymphangiogenesis in the tumor microenvironment and that GC cell‐derived CCL5 can also recruit TAM and enhance their secretion of tumor necrosis factor alpha (TNF‐*α)* which then increases the permeability of lymphatic vessel to facilitate LM of GC cells.

## 2 Results

### 2.1 CRIP1 is a Crucial LM‐Associated Marker in GC

**Figure 1:**
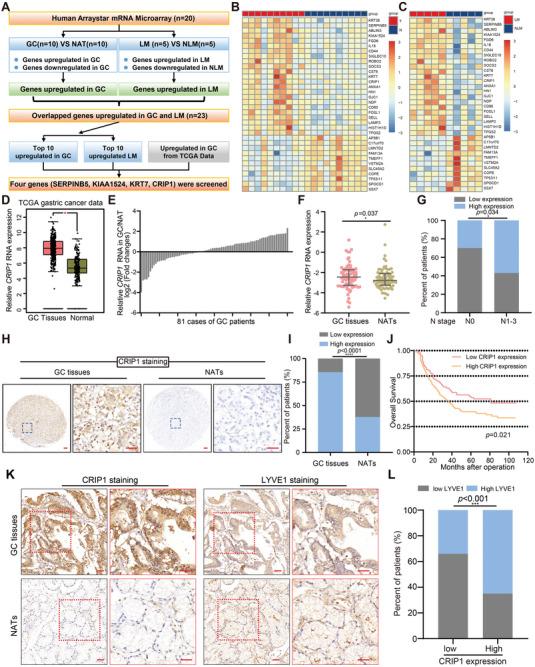
CRIP1 is a crucial lymphatic metastasis‐associated oncogene in GC. A) Flowchart for identifying potential genes regulating LM progression in GC. B,C) Heatmap showing expression of overlapping genes in GC tissues (“T”) compared with nontumorous adjacent tissues (NATs, “N”). B) *n* = 10 for each group and in LM‐positive GC tissues (“LM”) compared with LM‐negative GC (‘NLM'). C) *n* = 5 for each group. D) CRIP1 expression in GC and normal tissues was analyzed using data from TCGA database. The nonparametric Mann–Whitney U test was used. E,F) Realtime quantitative PCR (RT‐qPCR) analysis of *CRIP1* mRNA expression in 81 GC cases. Results shown as log2 (fold change) for GC versus NATs from each individual patient E) and relative expression in GC and NATs respectively F). The Wilcoxon rank sum test was used. G) The relationship between CRIP1 expression and N stage were analyzed in 81 GC specimens via the *χ*‐square test. H) Immunohistochemistry (IHC) analysis of CRIP1 protein expression in GC tissues and NATs (*n* = 305). Representative IHC images are presented. Scale bar = 50 µm. I) *χ*‐square test analyzing the expression of CRIP1 in this cohort. J) Patients were divided into two groups according to the cutoff value calculated by the receiver operating characteristics (ROC) method. The overall survival rate of these patients was analyzed with the Kaplan–Meier method and log‐rank test. K) Representative IHC images of CRIP1 and LYVE1 in GC and NATs. Scale bar = 50 µm. L) Analysis of LYVE1 expression relative to CRIP1 expression across patients. Statistical significance was assessed by *χ*‐square test. *p < 0.05, **p < 0.01, ***p < 0.001, ****p < 0.0001.

To identify potential genes regulating LM progression in GC, LM‐positive GC tissues (*n* = 5), LM‐negative GC tissues (*n* = 5), and their paired nontumorous adjacent tissues (NATs; *n* = 10) were subjected to mRNA microarray analysis. Clinicopathological characteristics of these patients were summarized in (Table S1, Supporting Information). Microarray results indicated that 1010 genes were significantly upregulated and 1069 genes were downregulated in GC tissues compared with NATs (Table S2, Supporting Information). Furthermore, 72 genes were upregulated in LM‐positive GC tissues as compared with LM‐negative tissues, with 80 genes downregulated (Table S3, Supporting Information). As depicted in the flowchart in (**Figure** **1**A), we cross‐referenced the datasets from GC versus NATs and LM‐positive versus LM‐negative GC to identify genes that were simultaneously upregulated both in GC tissues compared with NATs and in LM‐positive GC tissues compared with LM‐negative GC. The heatmaps in (Figure 1B,C) showed expression of the overlapped genes presented in both datasets. We next intersected the top 10 genes upregulated in GC and in LM‐positive GC with genes significantly upregulated in The Cancer Genome Atlas (TCGA) database.^[^ ^22^ ^]^ Consequently, four genes (SERPINB5, KIAA1524, KRT7, CRIP1) were preliminarily implicated (Figure 1D; and Figure S1A–C, Supporting Information). Subsequently, loss of function assay showed that knockdown of CRIP1, but not the others, reduced proliferation and invasion capacity in GC cells (Figure S1D–G, Supporting Information). Therefore, we focused on CRIP1 for further study.

To explore the function of CRIP1 in GC, we first verified CRIP1 mRNA expression in a cohort of 81 GC tissues and paired NAT samples via real‐time quantitative PCR (RT‐qPCR). The RT‐qPCR results showed CRIP1 was significantly upregulated in GC tissues compared with NATs, consistent with the microarray analysis (Figure 1E,F). Moreover, a positive correlation was observed between high CRIP1 expression and LM in the above 81 GC samples (Figure 1G). Additionally, analysis of TCGA data also revealed that CRIP1 was upregulated in various types of cancer compared with the normal tissues (Figure S2A–J, Supporting Information), which suggested that CRIP1 may be a crucial oncogene. Taken together, we identified CRIP1 as an important LM‐associated marker via large scale microarray analysis and validated its upregulation and positive association with LM in GC samples from our institution.

### 2.2 CRIP1 Upregulation Positively Correlates with Higher Lymphatic Vessel Density and Induces Lymphangiogenesis

To further validate the expression of CRIP1 in GC tissues, immunohistochemistry (IHC) was performed to visualize CRIP1 protein expression in another cohort of 305 paraffin‐embedded GC tissues. As shown in (Figure 1H,I), CRIP1 protein was markedly upregulated in GC tissues compared with NATs. CRIP1 upregulation positively correlated with LM, T stage, and M stage (Table S4, Supporting Information). Meanwhile, Kaplan–Meier survival analysis also showed that CRIP1 upregulation positively associated with poor prognosis (Figure 1J), which was consistent with the TCGA data in several types of human cancers (Figure S2K–N, Supporting Information).

**Figure 2:**
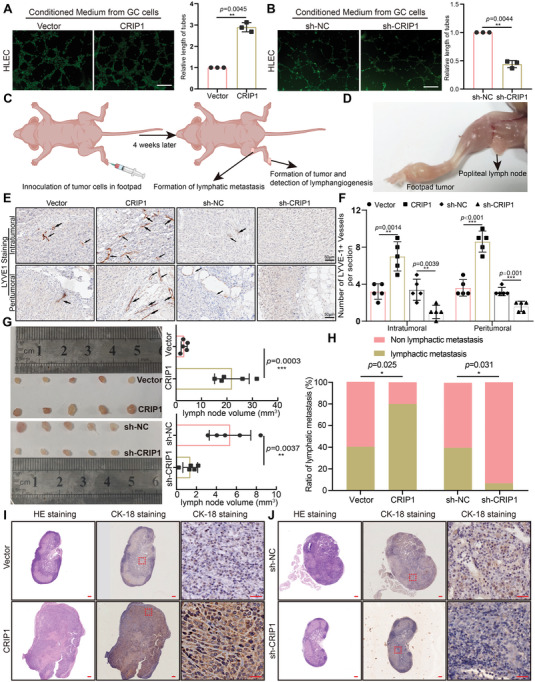
CRIP1 induces lymphangiogenesis and promotes the development of lymphatic metastasis in vivo. A,B) Representative images (left panels) and histogram quantification (right panels) of the Matrigel tube formation assay with human lymphatic endothelial cells (HLECs). HLECs were cultured with conditioned medium derived from GC cells treated as indicated. Scale bars = 200 µm. All experiments in vitro were performed with at least 3 biological replicates. The 2‐tailed Student's *t*‐test was used. C,D) Schematic representation for stablishing the nude mice model of popliteal LN metastasis (created with BioRender.com). E) Representative images of lymphatic vessels stained with anti‐LYVE1 in footpad tumor. F) Number of LYVE1^+^ lymphatic vessels stained with anti‐LYVE1. G) Representative images of enucleated popliteal lymph nodes (left panel) for groups and histogram analysis of the lymph node volumes (right panel). Statistical significance was assessed using 2‐tailed Student's *t*‐test. H) Ratios of metastatic to total dissected popliteal lymph nodes from mice inoculated with the indicated cells (*n* = 15 for each group). *χ*‐square test was performed to assess the statistical significance. I,J) Representative images of popliteal lymph nodes immunostained with anticytokeratin 18 antibody for CRIP1 overexpression group I) and CRIP1 knockdown group J). Scale bars = 50 µm. Error bars represent the mean±SD of three independent experiments. *p < 0.05, **p < 0.01, ***p < 0.001.

Tumor‐associated lymphangiogenesis, an independent prognostic factor in cancer, is considered to be closely associated with LM.^[^ ^23^ ^]^ Since CRIP1 expression significantly correlated with LM in GC, we examined the correlation between CRIP1 expression and lymphatic vessel density (LVD) in GC tissues. As shown in (Figure 1K,L), CRIP1 expression positively associated with LVD, as indicated by staining for the lymphatic vessel‐specific marker LYVE‐1, suggesting that CRIP1 might contribute to lymphangiogenesis in GC tissues. We then examined the effect of CRIP1 on lymphangiogenesis. First, the human lymphatic endothelial cells (HLECs) tube formation assay showed that conditioned medium from CRIP1 overexpressing GC cells promoted tube formation, whereas CRIP1 knockdown eliminated the ability of GC cells to induce tube formation (**Figure** **2**A,B). Subsequently, LVD was examined by immunostaining for LYVE‐1 in a in vivo popliteal LM model which was constructed via injecting GC cells with CRIP1 stable overexpression or knockdown into the footpads of immunodeficient BALB/c nude mice (Figure 2C,D). IHC results showed significantly increased LVD in footpad tumors from CRIP1 overexpressing cells, and decreased LVD in footpad tumors from CRIP1 knockdown GC cells (Figure 2E,F). Collectively, these results demonstrated that CRIP1 could significantly induce lymphangiogenesis both in vitro and in vivo.

### 2.3 CRIP1 Promotes the Development of LM

To further investigate the role of CRIP1 on LM, the lymph nodes from in vivo popliteal LM model were extracted. As shown in (Figure 2G), lymph node volumes in the CRIP1 overexpression group were significantly larger than in the vector group. In contrast, lymph nodes from the CRIP1 knockdown group presented obviously smaller volumes. We further examined the metastatic ratio via immunostaining for cytokeratin 18 (CK‐18), a specific marker for MGC‐803 cells, and cancer cells (Figure 2H–J). The lymphatic metastatic ratio (number of metastatic lymph nodes divided by total number of dissected lymph nodes) was larger in the CRIP1 overexpression group than in the vector group. Conversely, the CRIP1 knockdown group showed a significantly lower lymphatic metastatic ratio (Figure 2H–J). Taken together, these results from in vivo models indicated that CRIP1 could promote LM in GC.

### 2.4 CRIP1 Enhances the Proliferative and Metastatic Capacities of GC Cells

**Figure 3:**
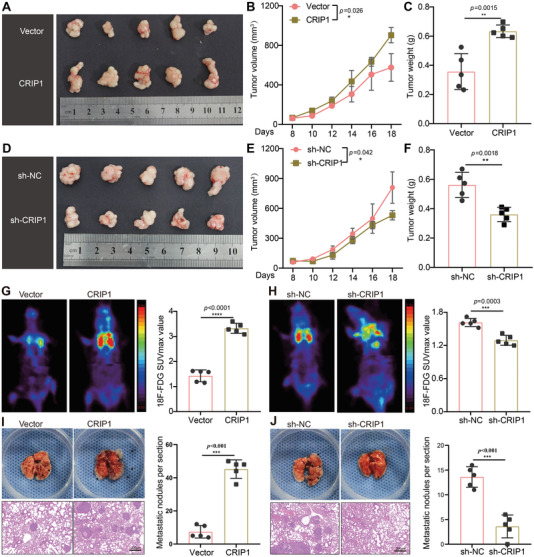
CRIP1 promotes GC cell proliferation and metastasis in vivo. A–C) Gross tumor images of subcutaneous xenografts from CRIP1 overexpression GC cells and controls A). Histogram quantification showing the tumor growth curve of in vivo xenograft volume (mm^3^) measured every 2 days B) and the final tumor weight (g) of xenografts in nude mice C). D–F) Representative subcutaneous xenograft images of CRIP1 knockdown GC cells and controls. G) The median coronal section of 18‐fluorodeoxyglucose (FDG) PET images of mice 49 days after tail vein injection with CRIP1 overexpression GC cells or controls, and max SUVs for each group. H) The median coronal section of 18‐ FDG PET images of mice 49 days after tail vein injection with CRIP1 knockdown GC cells or controls, and max SUVs for each group. I) Gross and microscopic lesions of lung tissues isolated from nude mice inoculated with CRIP1 overexpression and control cells via tail vein. Black arrows indicate metastatic nodule. Histogram showing number of metastatic nodules per lung section. J) Gross and microscopic lesions of lung tissues isolated from nude mice inoculated with CRIP1 knockdown and control cells via tail vein. Histogram showing number of metastatic nodules per lung section. Scale bar = 200 µm. Error bars represent the mean±SD of three independent experiments. *p < 0.05, **p < 0.01, ***p < 0.001.

Proliferative and metastatic capacity are crucial for influencing LM formation. Subsequently, in vitro proliferation assays including Cell Counting Kit‐8 (CCK‐8), 5‐Ethynyl‐2′‐deoxyuridine (EdU), and colony formation assays were performed to explore the effect of CRIP1 on GC cell proliferation and metastasis. These experiments all showed that ectopic CRIP1 expression enhanced proliferative capacity, whereas CRIP1 knockdown inhibited this process (Figure S3A–I, Supporting Information). CRIP1 overexpression or knockdown GC cells were then subcutaneously inoculated into nude mice. CRIP1 overexpression promoted tumor growth (**Figure** **3**A–C), while CRIP1 knockdown in GC cells reduced tumor growth (Figure 3D–F).

Transwell cell migration and invasion assays showed that CRIP1 overexpression promoted GC cell motility and invasiveness, whereas CRIP1 knockdown inhibited these effects (Figure S4A–D, Supporting Information). We constructed a lung metastasis model via tail‐vein injection of GC cells into nude mice, then used micro‐PET scanning to monitor in vivo metastasis formation. CRIP1 overexpression significantly promoted lung colonization by tumor cells, as evidenced by enhanced uptake of 18‐fluorodeoxyglucose (FDG) by GC cell colonies within the lungs, whereas CRIP1 knockdown strongly decreased lung metastasis (Figure 3G,H). Pathologically, CRIP1 overexpression resulted in more metastatic nodules in gross lung phenotype compared with the control group, whereas CRIP1 knockdown decreased metastatic nodule formation (Figure 3I,J). Collectively, our results revealed that CRIP1 could increase the proliferative and metastatic capacities of GC cells both in vitro and in vivo.

### 2.5 CRIP1 Promotes the Secretion of VEGFC and Leads to Increased Lymphangiogenesis and LM

**Figure 4:**
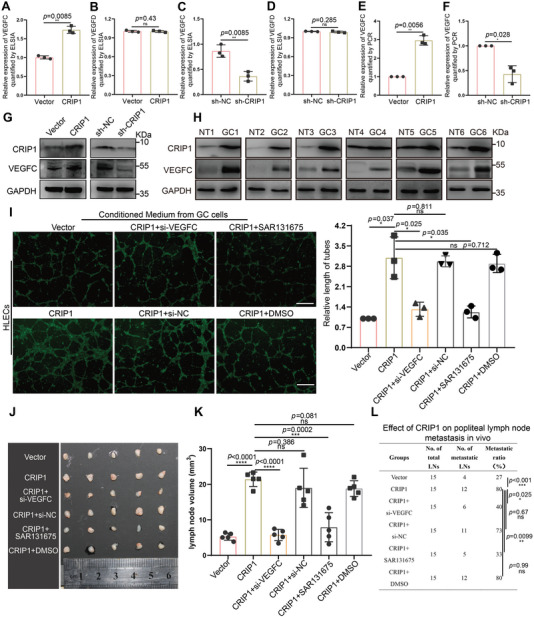
VEGFC is upregulated by CRIP1 in GC cells and involved in CRIP1‐mediated lymphangiogenesis and LM. A,B) ELISA showing the effect of CRIP1 overexpression on VEGFC A) and VEGFD B) secretion. C,D) ELISA showing the effect of CRIP1 knockdown on VEGFC C) and VEGFD D) secretion. E,F) RT‐qPCR showing the effect of CRIP1 overexpression E) and knockdown F) on VEGFC mRNA expression. G) Western blot showing the effect of CRIP1 overexpression and knockdown on VEGFC protein expression. H) Western blot showing the expression of CRIP1 and VEGFC in human GC samples. NT, nontumorous tissues; GC, gastric cancer tissues. I) Representative images (left panel) and quantification (right panel) of the Matrigel tube formation assay with human lymphatic endothelial cells (HLECs). HLECs were cultured with conditioned medium derived from GC cells treated as indicated. Scale bars = 200 µm. J) Representative images of enucleated popliteal lymph nodes across groups. K) Histogram analysis of lymph node volumes across groups. L) Ratios of metastatic to total dissected popliteal lymph nodes from mice inoculated with the indicated cells. *χ*‐square test was performed to assess the statistical significance in (L). Error bars represent the mean±SD of three independent experiments. *p < 0.05, **p < 0.01, ***p < 0.001.

Previous studies have documented that the VEGF family members VEGFC and VEGFD are biologically and clinically relevant to lymphangiogenesis and LM in multiple cancers.^[^ ^24^ ^]^ We then tested whether CRIP1‐mediated lymphangiogenesis is dependent on VEGFC or VEGFD. Enzyme‐linked immunosorbent assay (ELISA) was performed to examine levels of VEGFC and VEGFD in conditioned medium from GC cells with CRIP1 overexpression or knockdown, revealing that VEGFC expression was significantly elevated in conditioned medium from CRIP1 overexpressing cells and decreased upon CRIP1 knockdown, with no effect observed on VEGFD (**Figure** **4**A–D). RT‐qPCR and western blot further showed that CRIP1 could upregulate VEGFC expression at both the mRNA and protein levels (Figure 4E–G). Western blot analysis of human GC tissues further showed that CRIP1‐upregulated GC tissues exhibited overexpression of VEGFC (Figure 4H).

We next explored whether VEGFC was required for CRIP1‐induced lymphangiogenesis and LM. Downregulation of VEGFC rescued CRIP1‐promoted lymphangiogenesis in vitro (Figure 4I). A specific VEGFR3 inhibitor (SAR131675) was used to block the VEGFC/VEGFR3 signaling pathway. In vitro results showed that SAR131675 could rescue the effect of CRIP1 promoting lymphangiogenesis (Figure 4I). The in vivo popliteal LM model also showed that modulation of GC microenvironment through both inhibition of VEGFC expression and blocking VEGFC/VEGFR3 signaling could rescue the effect of CRIP1, as shown by reduced lymph node volume and decreased LM ratio (Figure 4J–L; and Figure S5, Supporting Information). Taken together, these in vitro and in vivo results demonstrated that CRIP1 could reshape GC environment through promoting the secretion of VEGFC which was required for CRIP1‐induced lymphangiogenesis and LM.

### 2.6 CRIP1 Interacts with cAMP responsive element binding protein 1 (CREB1) and Promotes Its Transcriptional Activity

**Figure 5:**
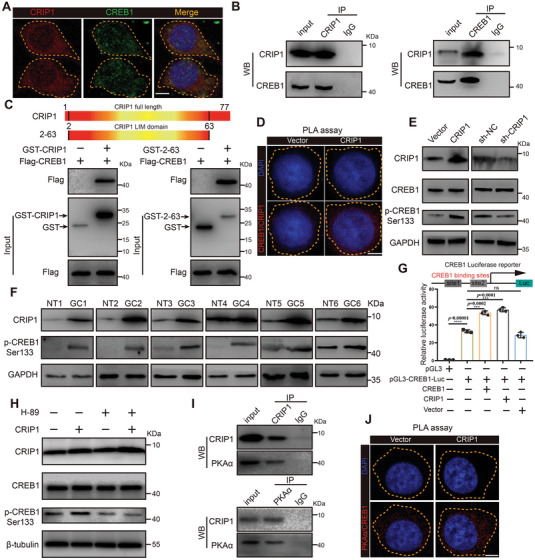
CRIP1 interacts with CREB1 and promotes its transcriptional activity. A) IF colocalization analysis of CRIP1 and CREB1 in MGC‐803 cells. Scale bar = 5 µm. B) Co‐IP analysis of interactions of endogenous CRIP1 and CREB1 immunoprecipitated by CRIP1 antibody (upper) and CREB1 antibody (lower). C) Constructed GST‐CRIP1 vectors containing full length CRIP1 and GST‐2‐63 sequences consisting of the amino acids for the LIM domain in the CRIP1 protein (upper). GST pull down results (lower) showing GST‐CRIP1 and GST‐2‐63 could pull down Flag‐CREB1 in vitro. D) Proximity ligation assay (PLA) analysis of the interaction between CRIP1 and CREB1 upon overexpression of CRIP1 in MGC‐803 cells. Scale bar = 5 µm. E) Western blot showing the effect of CRIP1 overexpression (left) and knockdown (right) on phosphorylation level of CREB1 at Ser 133. F) Western blot showing the expression of CRIP1 and p‐CREB1 in human sample. NT, nontumorous tissues; GC, gastric cancer tissues. G) Classical CREB1 binding sites were inserted into the promoter region of the CREB1 luciferase reporter. Promoter luciferase assays showing the influence of CREB1 or CRIP1 on the CREB1 promoter luciferase reporter. H) Western blot showing expression of CRIP1, CREB1, and p‐CREB1 (Ser 133) in GC cell groups under treatment with the PKA*α* inhibitor H‐89. I) Co‐IP analysis of interactions between endogenous CRIP1 and PKA*α* immunoprecipitated by CRIP1 antibody (left) and PKA*α* antibody (right). J) PLA analysis of the interaction between PKA*α* and CREB1 upon overexpression of CRIP1 in MGC‐803 cells. Scale bar = 5 µm. Error bars represent the mean±SD of three independent experiments. *p < 0.05, **p < 0.01, ***p < 0.001.

As the CRIP1 has a LIM domain which is a key mediator of protein–protein interactions, we applied LC–MS/MS to identify CRIP1‐interacting proteins using immunoprecipitation (IP) from GC cell protein lysates. Following proteomic analysis, we focused on CREB1, a basic/leucine zipper (bZIP) transcription factor abundant in the immunocomplex immunoprecipitated by the CRIP1 antibody (Table S5, Supporting Information). To confirm the interaction between CRIP1 and CREB1, we performed immunofluorescence (IF) to detect protein colocalization and found that CREB1 and CRIP1 expression overlapped intracellularly (**Figure** **5**A). Furthermore, co‐immunoprecipitation (co‐IP) assay showed that endogenous CREB1 was enriched in the protein complex immunoprecipitated by the CRIP1 antibody and endogenous CRIP1 was enriched in the protein complex immunoprecipitated by the CREB1 antibody (Figure 5B). Additionally, we constructed a GST‐CRIP1 vector which contained the full‐length *CRIP1* gene, and GST‐2‐63, which contained only the amino acids for the LIM domain in the CRIP1 protein. Both GST‐CRIP1 and GST‐2‐63 effectively pulled down Flag‐CREB1 protein in vitro (Figure 5C). Finally, a proximity ligation assay (PLA) showed that CRIP1 overexpression could enhance the CRIP1‐CREB1 interaction (Figure 5D).

CREB1 is a bZIP transcription factor whose transcriptional activity could be affected by various factors including posttranslational modifications. Studies have reported that phosphorylation of serine 133 strongly promotes the transcriptional activity of CREB1.^[^ ^25^ ^]^ We tested the influence of CRIP1 on CREB1 phosphorylation levels. Western blot showed that although CRIP1 overexpression had no influence on CREB1 protein level, it effectively increased serine 133 phosphorylation levels on the CREB1 protein (Figure 5E). Western blot analysis of human GC tissues further showed that CRIP1‐upregulated GC tissues exhibited overexpression of p‐CREB1 (Figure 5F). Thus, we speculated that CRIP1 could increase the transcriptional activity of CREB1. To verify this hypothesis, we constructed a CREB1 luciferase reporter with classical CREB1 binding sites inserted into the promoter region of the luciferase reporter. Endogenous CREB1 significantly increased the luciferase activity of the CREB1 reporter. Moreover, cotransfection of CREB1 or CRIP1 with the CREB1 luciferase reporter further increased its luciferase activity as compared to transfection of the CREB1 luciferase reporter alone (Figure 5G).

We further explored how CRIP1 increased serine 133 phosphorylation of the CREB1 protein. Previous studies report that PKA*α* is the main kinase responsible for serine 133 phosphorylation of CREB1.^[^ ^26^ ^]^ We used a PKA*α* specific inhibitor (H‐89) to block the effect of PKA*α*. Overexpression of CRIP1 increased serine 133 phosphorylation on CREB1, yet blocking PKA*α* with H‐89 rescued this effect (Figure 5H). Co‐IP results also showed that CRIP1 could interact with PKA*α* (Figure 5I). A PLA assay was further performed to examine the effect of CRIP1 on PKA*α* and CREB1 interaction. The assay showed that CRIP1 strongly enhanced the interaction between PKA*α* and CREB1 (Figure 5J). Taken together, CRIP1 could appear to interact with CREB1 via its LIM domain, and such interaction promote phosphorylation of serine 133 on CREB1 through enhancing the interaction between kinase PKA*α* and its substrate CREB1.

### 2.7 CREB1 Mediates the CRIP1‐Regulated Promotion of VEGFC Expression and Functions as a Transcription Factor for VEGFC

**Figure 6:**
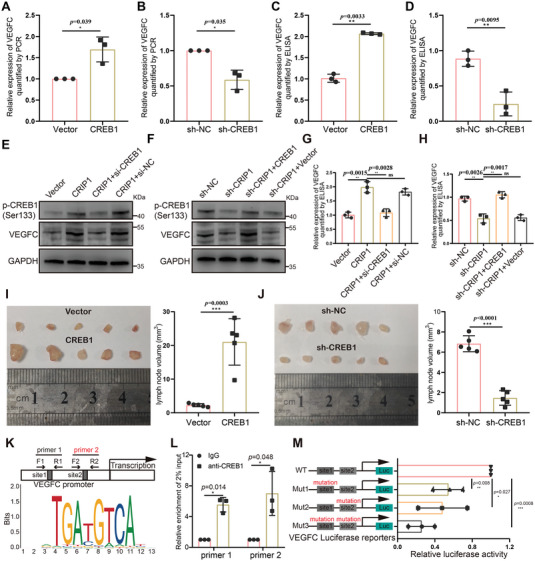
CREB1 mediates the CRIP1‐regulated promotion of VEGFC expression and functions as a transcription factor for VEGFC. A,B) RT‐qPCR showing the effect of CREB1 overexpression A) and knockdown B) on VEGFC mRNA expression. C,D) ELISA showing the effect of overexpression C) and knockdown D) of CREB1 on VEGFC secretion. E,F) Western blot analysis of p‐CREB1 (Ser 133) and VEGFC expression in CRIP1 overexpression GC cells rescued by CREB1 knockdown E) and in CRIP1 knockdown GC cells rescued by CREB1 overexpression F). G,H) ELISA showing the expression of VEGFC in CRIP1 overexpression GC cells rescued by CREB1 knockdown G) and in CRIP1 knockdown GC cells rescued by CREB1 overexpression H). I) Representative images of enucleated popliteal lymph nodes for CREB1 overexpression cells (left panel). Histogram analysis of the lymph node volumes for groups (right panel). J) Representative images of enucleated popliteal lymph nodes for CREB1 knockdown cells (left panel). Histogram analysis of the lymph node volumes for groups (right panel). K) Specific primers were designed for potential CREB1 binding sites in the VEGFC promoter. L) Chromatin immunoprecipitation (ChIP) showing DNA fragments from the VEGFC promoter enriched in the DNA‐protein complex immunoprecipitated via CREB1 antibody. M) Promoter luciferase assay showing that binding of CREB1 to the VEGFC promoter sites could enhance VEGFC transcription. Error bars represent the mean±SD of three independent experiments. *p < 0.05, **p < 0.01, ***p < 0.001.

To determine whether CRIP1 modulates VEGFC expression and lymphangiogenesis through CREB1, we explored the effect of CREB1 on VEGFC expression. RT‐qPCR, ELISA and western blot showed that CREB1 significantly elevated VEGFC expression in GC cells (**Figure** **6**A–D; and Figure S6A, Supporting Information). We also found that knocking down CREB1 eliminated the effect of CRIP1 promoting VEGFC expression, while CREB1 overexpression rescued the lower VEGFC seen upon CRIP1 knockdown (Figure 6E–H). These data revealed that CREB1 was an important mediator for CRIP1 promoting VEGFC expression. We further tested the role of CREB1 on lymphangiogenesis and LM. Conditioned medium from CREB1 overexpressing GC cells promoted HLECs lymphatic tube formation in vitro, while CREB1 knockdown inhibited this process (Figure S6B, Supporting Information). The popliteal LM model also indicated that CREB1 could promote LM formation, as shown by increased lymph node volume and LM ratio in the CREB1 overexpression group and reduced lymph node volume and LM ratio after CREB1 knockdown (Figure 6I,J; and Figure S6C,D, Supporting Information).

Then, we speculated whether it could function as an upstream transcription factor for VEGFC. We found two potential CREB1 binding motifs within the VEGFC promoter region sequence. Specific primers were designed for these sequences, as shown in (Figure 6K). Chromatin immunoprecipitation (ChIP) showed DNA fragments of the VEGFC promoter region enriched in the DNA‐protein complex immunoprecipitated by the CREB1 antibody (Figure 6L). We performed promoter luciferase reporter assays to explore whether binding of CREB1 to the VEGFC promoter region could enhance VEGFC transcription. Compared with the WT luciferase reporter, the MUT luciferase reporters presented obviously decreased luciferase activity after mutating one or both CREB1 binding sites (Figure 6M), indicating that CREB1 could transcriptionally activate VEGFC expression through binding the above two motifs.

### 2.8 CRIP1 Could Enhance the Lymphatic Permeability, Which is Partially Rescued by Blocking of VEGFC‐VEGFR3 Pathway While Largely Reversed by CCL5 Neutralizing Antibody

**Figure 7:**
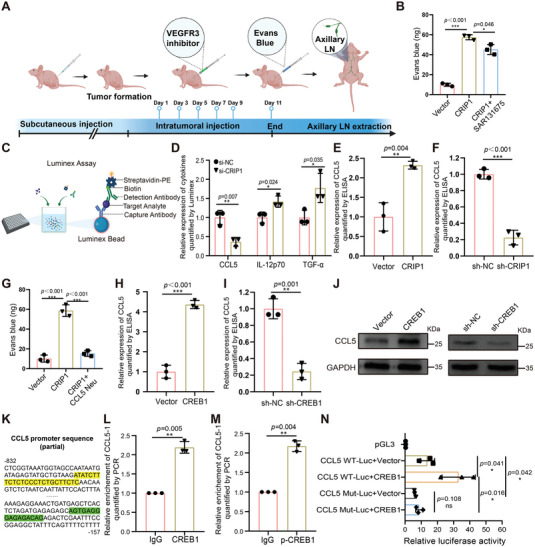
CRIP1 promotes the lymphatic permeability through enhanced secretion of CCL5. A) An in vivo model using nude mice was constructed by subcutaneous injection of CRIP1 overexpression cells. After tumor formation, VEGFR3 inhibitor was intratumorally injected once every 2 days for five cycles. Evans blue was injected into tumors and the pair of axillary lymph nodes was obtained for analysis. (Created with BioRender.com.) B) The amount of Evans blue reflux in axillary lymph nodes obtained from nude mice inoculated with CRIP1 overexpression cells and treated with SAR131675. C) Schematic diagram of the Luminex experiment. D) Relative expression of cytokines and chemokines which showed significant differences between groups as quantified by Luminex. E,F) Relative expression of CCL5 detected by ELISA in CRIP1 overexpression or knockdown groups. G) The amount of Evans blue reflux in axillary lymph nodes obtained from nude mice inoculated with CRIP1 overexpression cells and treated with CCL5 neutralizing antibody. H,I) Relative expression of CCL5 detected by ELISA in CREB1 overexpression or knockdown groups. J) Relative expression of CCL5 detected by Western blot in CREB1 overexpression or knockdown groups. K) Two predicted binding sites for CREB1 in the CCL5 promoter sequence, CCL5‐1 (green) CCL5‐2 (yellow). L,M) ChIP was used to detect interactions between CCL5 and CREB1 or p‐CREB1. CREB1 and p‐CREB1 antibodies both enriched for CCL5‐1. N WT and MUT CCL5 promoter luciferase reporter plasmid activities. Error bars represent the mean±SD of three independent experiments. *p < 0.05, **p < 0.01, ***p < 0.001.

Lymphatic permeability is an important restriction for cancer cell intravasation through lymph vessels before colocalization in lymph node. To investigate the effect of CRIP1 on lymphatic permeability, we constructed an in vivo model using nude mice, shown in (**Figure** **7**A). We began by subcutaneous injection of CRIP1 overexpression cells and control group cells into nude mice. At the end of experiments, we performed intratumoral injection of Evans blue and resected the pair of axillary lymph nodes. The reflux of Evans blue into the axillary lymph nodes could be used as an indicator of lymphatic permeability. We found significant increase of Evans blue reflux in the CRIP1 overexpression group compared with the vector group, showing that CRIP1 could significantly enhance the lymphatic permeability. To confirm whether the increased lymphatic permeability is led by VEGFC, we set a group which is treated by VEGFR3 inhibitor once every 2 days for five cycles during the in vivo permeability detection model. The results showed that blocking the VEGFC‐VEGFR3 pathway could partially rescue the lymphatic permeability (Figure 7B), indicating that there might be other factors mainly responsible for the increased permeability caused by CRIP1 overexpression.

To explore this factor, the Luminex assay was used to analyze the conditioned medium of CRIP1 knockdown GC cells (Figure 7C). Forty‐five total cytokines and chemokines were detected in the conditioned medium, among which CCL5 levels significantly declined in the CRIP1 knockdown group while IL‐12p70 and TGF‐*α* were elevated (Figure 7D; and Figure S7A–C, and Table S6, Supporting Information). We used ELISA to validate the Luminex assay, finding that CCL5 concentration in conditioned medium was markedly increased in the CRIP1 overexpression group and decreased in knockdown group (Figure 7E,F). These results indicated that CRIP1 could indeed upregulate CCL5 secretion. To explore whether the increased CCL5 secretion was responsible for the increased permeability led by CRIP1 overexpression, we again constructed the in vivo permeability model described in (Figure 7A). We found significant increase of Evans blue reflux in the CRIP1 overexpression group compared with the vector group, and intratumoral injection of CCL5 neutralizing antibody largely reduced the amount of Evans blue in the lymph nodes (Figure 7G). Taken together, these results revealed that CRIP1 could enhance the lymphatic permeability. Such effects could be partially rescued by blocking of VEGFC‐VEGFR3 pathway, while it was largely reversed by CCL5 neutralizing antibody.

### 2.9 CCL5 Expression is Upregulated by CRIP1 through CREB1‐Mediated Transcription to Enhance TAM Recruitment

These above results indicated that CRIP1 could indeed upregulate CCL5 secretion. We next used ELISA to explore whether CREB1 played a similar role in regulating CCL5 expression. Similar to CRIP1, CREB1 markedly upregulated the secretion of CCL5 (Figure 7H,I). Subsequently, RT‐qPCR and western blot indicated upregulated CCL5 mRNA and protein levels upon CREB1 overexpression and downregulated CCL5 upon CREB1 knockdown compared with controls (Figure 7J; and Figure S8A,B, Supporting Information). To further explore whether CREB1 transcriptionally regulated CCL5 expression, we analyzed the CCL5 promoter region and found two predicted binding sites for CREB1 (Figure 7K). Specific primers were designed based on the sequences of the two binding sites, named CCL5‐1 and CCL5‐2 respectively. ChIP results showed CCL5‐1 was pulled down by CREB1 or p‐CREB1 antibodies compared with IgG, while CCL5‐2 enrichment was unchanged (Figure 7L,M; and Figure S8C,D, Supporting Information), confirming that CREB1 and p‐CREB1 could indeed interact with the CCL5‐1 site. To further investigate the interaction between CREB1 and CCL5, we constructed a luciferase reporter based on the WT promoter sequence of CCL5 and a CCL5‐1 mutated reporter. Dual luciferase reporter assays revealed significantly increased luciferase activities when CREB1 was overexpressed in the WT group, with little change in the MUT group (Figure 7N).

**Figure 8:**
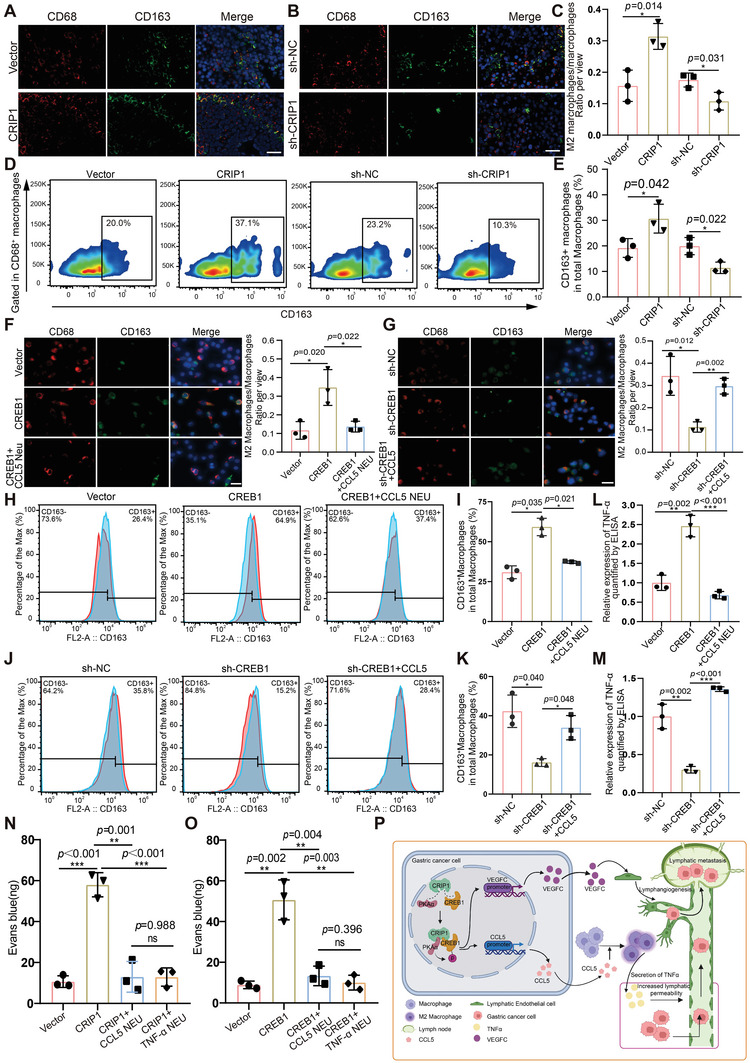
CRIP1‐mediated CCL5 secretion promoted M2 macrophage recruitment to increase lymphatic vessel permeability. A,B) M2 TAM recruitment was detected by IF staining for CD68 (red) and CD163 (green) in CRIP1 overexpression A) or knockdown B) groups. C) Percentage of M2 TAM across groups. D,E) Flow cytometry analysis of phenotype of macrophage from subcutaneous xenograft. F) The percentage of M2 macrophages increased when cocultured with CREB1 overexpression supernatant and decreased upon adding CCL5 neutralizing antibody to the supernatant. G) The percentage of M2 macrophages decreased when cocultured with CREB1 knockdown supernatant and rescued upon adding CCL5 recombinant protein to the supernatant. H,I) Flow cytometry analysis of M2 macrophages increased after cocultured with CREB1 overexpression supernatant. J,K) Flow cytometry analysis of M2 macrophages decreased after cocultured with CREB1 knockdown supernatant. L,M) Relative expression of TNF‐*α* in cultured supernatant quantified by ELISA. N) The amount of Evans blue reflux in axillary lymph nodes obtained from nude mice injected by CRIP1 overexpression cells. O) The amount of Evans blue reflux in axillary lymph nodes obtained from nude mice injected by CREB1 overexpression cells. P) Schematic diagram describing that CRIP1 could promote lymphangiogenesis and LM development in GC (created with BioRender.com). CRIP1 could interact with the CREB1 to increase its transcriptional activity. CREB1 functioned as a transcription factor for *VEGFC* and *CCL5*. Enrichment of VEGFC in the GC microenvironment could induce lymphangiogenesis, while increased CCL5 in tumor microenvironment could form a concentration gradient to recruit M2 TAM, then promote TNF‐*α* secretion to increase lymphatic permeability. Through the reciprocal effects of VEGFC and CCL5, CRIP1 could promote lymphangiogenesis and LM development in GC. Error bars represent the mean±SD of three independent experiments. *p < 0.05, **p < 0.01, ***p < 0.001.

Then we asked how the upregulation of CCL5 brought by CRIP1 regulate the lymphatic permeability. Many studies have implicated CCL5 as a major chemokine responsible for recruiting TAM which was reported to promote lymphoinvasion in breast cancer.^[^ ^12^, ^27^, ^28^ ^]^ We thus used IF and flow cytometry analysis to detect whether CRIP1 could recruit TAM in vivo. The IF and flow cytometry analysis results both showed CRIP1 overexpression increased the percentage of M2 phenotype TAM (CD163^+^CD68^+^) whereas CRIP1 knockdown decreased M2 infiltration instead of M1 phenotype TAM (CD80^+^CD68^+^) (**Figure** **8**A–E; and Figure S8E–G, Supporting Information). In addition, we also designed an in vitro coculture system to detect macrophage differentiation in vitro. The IF results showed that proportion of M2 macrophages was significantly elevated after coculture with CREB1 overexpression supernatant, and this elevation was significantly suppressed by CCL5 neutralizing antibody (Figure 8F). Conversely, the proportion of M2 macrophages was significantly decreased after coculture with CREB1 knockdown supernatant, and this downtrend was reversed upon adding recombinant CCL5 protein to the CREB1 knockdown supernatant (Figure 8G). Meanwhile, the results of flow cytometry analysis were consistent with the finding as revealed by IF detection (Figure 8H–K; and Figure S8H–K, Supporting Information).

### 2.10 CRIP1 Increases Lymphatic Vessel Permeability through CCL5‐Mediated TAM Recruitment and Subsequent TNF‐*α* Secretion from TAM

TAM often secretes cytokines that influence other cells within the microenvironment. TNF‐*α*, an important cytokine secreted by macrophages, has been widely reported to act on vascular endothelial cells.^[^ ^29^ ^]^ To investigate the potential effect of TAM on lymphatic endothelial cells, we first performed ELISA to detect TNF‐*α* secretion of TAM in the supernatant of the above coculture system. As shown in Figure 8L, TNF‐*α* concentration was markedly increased in the conditioned medium of macrophages co‐cultured with CREB1 overexpression supernatant, and this elevation was suppressed by CCL5 neutralizing antibody. Conversely, CREB1 knockdown supernatant reduced TNF‐*α* secretion by macrophages, and the addition of recombinant CCL5 protein increased the TNF‐*α* concentration (Figure 8M). These results indicated that CCL5 was responsible for the increased secretion of TNF‐*α* by macrophages. We then questioned whether such TNF‐*α* could also act on lymphatic endothelial cells to cause changes in lymphatic permeability. To test this hypothesis, we constructed the in vivo permeability detection model shown in (Figure 7A). We began by subcutaneously injecting CRIP1 overexpression cells or CREB1 overexpression cells into nude mice. When the subcutaneously transplanted tumors formed, we performed intratumoral injection with CCL5 neutralizing antibody or TNF‐*α* neutralizing antibody once every 2 days for five cycles. We found a significant increase in Evans blue reflux in the CRIP1 or the CREB1 overexpression group compared with the vector group, and intratumoral injection of CCL5 neutralizing antibody or TNF‐*α* neutralizing antibody both significantly reduced the amount of Evans blue in the lymph nodes (Figure 8N,O). As the vessel permeability was correlated with VE‐cadherin,^[^ ^30^ ^]^ we then explored the in vitro permeability via detecting VE‐cadherin. The results showed that the staining of VE‐cadherin was decreased in HLEC after coculture with CREB1 overexpression supernatant compared with control supernatant, and this downtrend was rescued by CCL5 neutralizing antibody and TNF‐*α* neutralizing antibody (Figure S8L, Supporting Information). Conversely, the staining of VE‐cadherin was increased in HLEC after co‐culture with CREB1 knockdown supernatant, which could be reversed by recombinant human CCL5 and TNF‐*α* (Figure S8M, Supporting Information). Taken together, these results indicated that CRIP1‐mediated CCL5 secretion promoted M2 macrophage recruitment, which subsequently induced TNF‐*α* secretion, ultimately increasing lymphatic vessel permeability and possibly allowing for easier lymph node metastasis. We also performed IHC staining of CRIP1, p‐CREB1, VEGFC, and CCL5 in clinical GC tissues. The results showed that there is positive association between CRIP1 and p‐CREB1 and the expression of p‐CREB1 is also positively associated with VEGFC and CCL5. These results further support the regulatory axis of CRIP1‐CREB1‐VEGFC/CCL5 in lymphangiogenesis and lymph vessel permeability in GC (Figure S9, Supporting Information). In conclusion, CRIP1 could interact with the CREB1 to increase its transcriptional activity through elevating its phosphorylation level. CREB1 functioned as a transcription factor for VEGFC and CCL5. Enrichment of VEGFC in the GC microenvironment could induce lymphangiogenesis, while increased CCL5 in tumor microenvironment could form a concentration gradient to recruit M2 TAM, then promote TNF‐*α* secretion to increase lymphatic permeability. Through the reciprocal effects of VEGFC and CCL5, CRIP1 could promote lymphangiogenesis and LM development in GC (Figure 8P).

## 3 Discussion

In this study, we identified an important LM‐associated oncogene, CRIP1, and confirmed that CRIP1 effectively promoted the development of LM in GC through enhancing lymphangiogenesis and lymphatic permeability. Mechanistically, CRIP1 could function as a protein scaffold to interact with both CREB1 and PKA*α*, which enhance phosphorylation level of CREB1 catalyzed by PKA*α*. Such action could significantly increase the transcriptional activity of CREB1 which served as a common transcription factor for *VEGFC* and *CCL5*. CRIP1 and CREB1 mediated secretion of VEGFC and CCL5 could then reshape the tumor microenvironment into a suitable “soil” which favor lymphangiogenesis and LM in GC. Enrichment of VEGFC in the GC microenvironment could induce lymphangiogenesis, while increased CCL5 in tumor microenvironment could form a concentration gradient to recruit M2 type TAM, then promote TNF‐*α* secretion to increase lymphatic permeability. Through the reciprocal effects of VEGFC and CCL5, CRIP1 could promote lymphangiogenesis and LM development in GC, making it an ideal target for controlling lymphangiogenesis and LM in GC.

CRIP1 is an evolutionarily conserved LIM family protein containing the LIM domain, a major functional domain that mediates protein interactions in eukaryotes.^[^ ^15^ ^]^ Because of the capacity of the LIM domain to carry zinc, it was first revealed that CRIP1 functioned in physiological zinc absorption in the gastrointestinal tract. In our paper, we revealed that CRIP1 could work through a nonclassical zinc transport role to regulate lymphangiogenesis and LM in GC. CRIP1 functioned as a molecular chaperon to facilitate interactions between CREB1 and PKA*α*, thus increasing the phosphorylation and transcriptional activity of CREB1, which may be summarized as a transcription coactivator‐like function. Previous studies have also reported that CRIP1 interacts with other proteins to exert its regulatory function.^[^ ^17^, ^18^, ^31^ ^]^ Based on the LC‐MS/MS results and experimental confirmation, we identified an unreported interaction between CRIP1, CREB1, and PKA*α*, which may present an effective target route for enhancing GC therapeutic efficacy in the future.

VEGFC is an essential factor for lymphangiogenesis in tumors. VEGFC binding to its receptor VEGFR3 can activate VEGFC/VEGFR3 signaling and enhance formation of new lymphatic vessels, leading to LM development.^[^ ^32^, ^33^, ^34^ ^]^ Here, we first reported that VEGFC expression could be markedly increased by CRIP1. Meanwhile, inhibition of VEGFC expression and blocking the VEGFC/VEGFR3 signaling pathway both rescued the effect of CRIP1 in inducing lymphangiogenesis and LM in GC. Mechanistically, we further revealed CREB1 as a bona fide transcription factor for VEGFC. CRIP1 functions as a protein chaperone to interact with CREB1 and enhances its transcriptional activity to promote VEGFC expression. VEGFC was reported to be regulated by various transcription factors, such as TBL1XR1,^[^ ^35^ ^]^ the NF‐kB pathway downstream transcription factor p65,^[^ ^36^ ^]^ the YAP/TEAD complex,^[^ ^37^ ^]^ FOXK1,^[^ ^38^ ^]^ etc. CREB1 was revealed as a new transcription factor for VEGFC in GC which may expand the transcriptional regulation of VEGFC in other disease. Furthermore, our research may provide a new direction for targeting VEGFC/VEGFR3 signaling in GC.

The lymphangiogenesis together with increased lymphatic permeability facilitated tumor cell intravasation into lymph vessels and promoted metastatic dissemination in lymph nodes.^[^ ^39^, ^40^, ^41^ ^]^ In our study, we found that CRIP1 not only promoted lymphangiogenesis in GC, but also increased the lymphatic permeability of the lymph vessels. Previous studies showed that VEGFC‐VEGFR3 pathway activation increased lymphangiogenesis, meanwhile, enhanced lymphatic permeability through disrupting the lymphatic endothelial barrier.^[^ ^40^ ^]^ Consistent with this finding, we showed that blocking of VEGFC‐VEGFR3 pathway could partially rescue the increased permeability caused by CRIP1 overexpression. Interestingly, we found that inhibition of CCL5 could effectively and largely reverse the lymphatic permeability brought by CRIP1 overexpression through Luminex multifactor screening. Enhanced CCL5 could promote M2 type TAM recruitment to GC microenvironment and subsequent secretion of TNF‐*α* from TAM, thus increasing lymphatic permeability. Previous studies reported that TAM could influence cancer metastasis and formation of blood or lymphatic vessels.^[^ ^12^, ^42^, ^43^ ^]^ TNF‐*α* has been shown to increase lymphatic vessel permeability by modulating the cytoskeleton of endothelial cells.^[^ ^44^ ^]^ Here, we posit that TAM recruitment caused by CRIP1 overexpression promote LM by increasing the permeability of lymphatic vessels via secreting TNF‐*α*, which may enrich the understanding of TAM in promoting LM formation.

In conclusion, we identified CRIP1 as an important LM‐associated oncogene. CRIP1 significantly increased the proliferative and metastatic capacities of GC cells and induced lymphangiogenesis and LM in GC. Mechanistically, CRIP1 interacted with CREB1 via its LIM domain and promoted the serine 133 phosphorylation on CREB1 through enhancing the interaction between PKA*α* and CREB1. CREB1 served as bona fide transcription factor for VEGFC and CCL5. Secretion of VEGFC and CCL5 mediated by CRIP1 and CREB1 could reshape the tumor microenvironment into a suitable “soil” which could favor lymphangiogenesis and LM. An in‐depth understanding of the function of CRIP1 in GC may accelerate the development of therapeutic strategies for GC patients with LM.

## 4 Experimental Section

### Tissues

Eighty‐one GC tissue samples and matched NATs were obtained from patients who were newly diagnosed and received surgical resection at the First Hospital of China Medical University. Informed consent was obtained from all patients enrolled in this study. Pathological stage was staged according to the eighth TNM staging of the International Union against Cancer (UICC)/American Joint Committee on Cancer (AJCC) system. All research complied with the principles of the Declaration of Helsinki, and approval was acquired from the Research Ethics Committee of the First Hospital of China Medical University (AF‐S0P‐07‐1.1‐01).

### Microarray Analysis

Total RNA was extracted from paired GC and NATs using TRIzol Reagent (#15 596 026, Invitrogen, CA) according to the manufacturer's instructions. After extraction, RNA integrity and concentration were examined prior to labeling. RNA integrity was assessed using denaturing agarose gel electrophoresis. RNA concentration and purity were assessed by a NanoDrop ND‐1000 (Thermo Fisher Scientific, CA). RNA labeling was performed using a Quick Amp Labeling Kit (Agilent Technologies, CA), and hybridization to Arraystar_Human_ mRNA_8×60 k v4.01 microarrays was performed in Agilent Sure Hyb Hybridization Chambers. Hybridized microarrays were scanned using an Agilent DNA Microarray Scanner, with data extracted using Agilent Feature Extraction software. Differently expressed mRNAs with statistical significance were identified using Box plot and Scatter plot filtering. The thresholds used to screen upregulated or downregulated mRNAs were fold change > = 2.0, *p*‐value < = 0.05.

### Cell Culture

MGC‐803, HGC‐27 (purchased from the Institute of Biochemistry and Cell Biology at the Chinese Academy of Sciences, Shanghai, China), and THP1 (iCell Bioscience Inc, Shanghai, China) were cultured in RPMI 1640 medium (Invitrogen, Carlsbad, CA) supplemented with 10% fetal bovine serum, 1% penicillin, and streptomycin. HLECs were obtained from ScienCell Research Laboratories (ScienCell, San Diego, CA). HLECs were cultured in endothelial cell medium (ScienCell, San Diego, CA) supplemented with 10% fetal bovine serum, 1% endothelial cell growth supplement, and 1% antibiotic solution. All cells were cultured in a humidified incubator at 37 °C with 5% CO_2_ (Thermo, Waltham, MA). None of the cell lines were listed in the database of commonly misidentified cell lines maintained by the International Cell Line Authentication Committee (ICLAC). All cell lines were routinely tested for mycoplasma contamination and were all free of contamination.

### Luminex Assay

The human Luminex kit (R&D Systems, MN) was used to measure the expression levels of 45 cytokines in the samples. Conditioned cell culture medium samples were added to a mixture of luminex beads coded by two fluorochromes in different proportions, precoated with analyte‐specific capture antibodies. Biotinylated detection antibodies and phycoerythrin (PE) conjugated streptavidin were added to bind to the antigen‐antibody complex. Concentration values were obtained from fluorescence intensity using a Luminex 200 System detector (R&D systems, MN).

### GST Pull Down

A recombinant technique was used to fuse the probe protein with GST. The recombinant plasmid was transformed into *E.coli* BL21 (#9216, Takara, Dalian, China). Cultured bacteria were broken by ultrasonication for 30 min. Recombinant protein was incubated with total proteins extracted from the MGC‐803 cells then the complex was purified using Glutathione Purification Resin (Beyotime Biotechnology, Shanghai, China) at 4 °C overnight in rotation. After eluting, protein interactions were examined via western blot analysis.

### ELISA

Cell supernatants were collected to quantify the secretion of VEGFC (DVEC00, R&D systems, MN), VEGFD (DVED00, R&D systems, MN), CCL5 (mlbio, Shanghai, China), and TNF‐*α* (mlbio, Shanghai, China) via ELISA assay. Supernatants were diluted at 1:4 then added into wells coated with specific antibodies. After incubation with enzyme‐labeled antibodies at 37 °C for 60 min and chromogen solution at 37 °C for 15 min, the reaction was stopped and the absorbance of each well at 450 nm was measured using a Tecan Infinite 200 Pro microplate reader (Tecan, Shanghai, China). Marker concentrations in the samples were then calculated based on the standard curve drawn using standard samples.

### HLECs Tube Formation Assay

Conditioned medium was collected from cultured GC cell groups. Before coating, the HLECs, pipette tips, Ibidi angiogenesis slides (Ibidi, German), and Matrigel (Corning, NY) were all precooled at 4 °C overnight. 5×10^3^ HLECs were seeded together with conditioned medium in Ibidi angiogenesis slides pre‐coated with Matrigel. Six hours later, the capillary‐like structures formed by HLECs were stained by Calcein‐AM (Biolegend, CA) and visualized via fluorescence microscope (DM4500B, Leica, German). Image J was used to measure the length of tubes.

### Dual‐Luciferase Reporter Assay

VEGFC promoter and CCL5 promoter luciferase reporter plasmids were synthesized and inserted into the pGL3‐basic vector between Mlul and Xhol sites (Genomeditech Biotech, Shanghai, China). These promoter luciferase reporters were then cotransfected with renilla luciferase expression plasmid using lipofectamine 2000 reagent (Invitrogen, CA). Forty‐eight hours later, the relative luciferase activity of cell lysates was measured using the Dual‐Luciferase Reporter Assay System (E1910, Promega, Beijing, China) kit according to the manufacturer's instruction, and signal was detected via Infinate M200 PRO microplate reader (Tecan, Shanghai, China).

### ChIP

The ChIP assay was performed using a ChIP kit (Cell signaling Technology, Boston, MA) following the provided instructions. Transfected cells were treated with 1% formaldehyde for 10 min to cross‐link proteins and DNA. After ultrasonication, equal aliquots of chromatin sample were immunoprecipitated with anti‐CREB1 (#9197, Cell signaling Technology, Boston, USA), anti‐p‐CREB1 (#9198, Cell signaling Technology, Boston, MA), or IgG at 4 °C overnight in rotation. The protein A/G bead‐antibody‐chromatin complexes were then washed with a low‐salt, high‐salt buffer. NaCl buffer and Proteinase K were used to reverse cross‐link the protein‐DNA interaction. Purified DNA was examined via RT‐qPCR and the relative expression of 2% input was calculated as the enrichment of DNA in the immunocomplex. Primers were listed in the Supporting Information.

### PLA

For the PLA, the Duolink In Situ Red Starter Kit Mouse/Rabbit (DUO92101, Sigma, MA) was used following the manufacturer's instruction. Briefly, MGC‐803 cells were grown on glass coverslips and fixed using 4% paraformaldehyde. The cells were washed with PBS three times and permeabilized using 0.1% Triton X‐100 in PBS for 15 min. After blocking, cells were incubated with primary antibodies from different species (rabbit or mouse) diluted in blocking solution overnight at 4 °C. The next day, slides were incubated with prediluted antirabbit (plus) and antimouse (minus) probes for 1 h at 37 °C. After that, cells were incubated with 1× ligase for 30 min and 1× polymerase for 100 min at 37 °C. Finally, coverslips were mounted onto slides with Duolink In situ Mounting Medium with DAPI. Pictures were obtained via a Niko A1R confocal microscope system (Nikon Corporation, Tokyo, Japan).

### Animal Experiments

2×10^6^ MGC‐803 cells in 0.2 mL PBS were injected into the right armpit region of BALB/c nude mice randomly divided into groups (*n* = 5 for each group). Tumor sizes were measured every 2 days with vernier calipers, and the volume of subcutaneous tumor was calculated using the following formula: Tumor volume = length × width^2^ /2. At the end of experiment, the mice were sacrificed and tumors were isolated from the subcutaneous tissue to measure volume and weight.

For the popliteal lymph node metastasis model, 1×10^5^ MGC‐803 cells in 0.05 mL PBS were injected into the footpads of BALB/c nude mice which were randomly divided into groups (*n* = 5 for each group). Four weeks after injection, primary footpad tumors, and popliteal lymph nodes were enucleated for measurement.

For lung metastasis experiments, 2×10^6^ MGC‐803 cells in 0.2 mL PBS were injected into the tail vein of BALB/c nude mice randomly divided into groups (*n* = 5 for each group). Seven weeks later, a positron emission tomography (PET) scanner (Madic Technology, Shandong, China) was used to perform 18‐fluorodeoxyglucose PET (18F‐FDG PET) scans. 7–12 MBq of 18F‐FDG was administered via tail vein into mice fasted for at least 4 h. PET images were evaluated by two experienced nuclear medicine physicians. For semiquantitative analysis, the maximum standardized uptake value (SUVmax) was measured and calculated according to the formula: SUV = The radioactive concentration in the tumor lesion (MBq/g) × Body weight of mice (g). The injected dose of 18F‐FDG (MBq).

After scanning, intact lung tissues were isolated and stained with hematoxylin and eosin (H&E). H&E‐stained lung sections were scanned via Pannoramic 250 FLASH Slide scanner, and number of metastatic cancer nodules in each section was counted.

For all animal experiments, the operators and investigators were blinded to group allocation. All experimental procedures involving animals were done in accordance with the Guide for the Care and Use of Laboratory Animals (NIH publication no. 80‐23, revised 1996) and the institutional ethical guidelines in the Institutional Animal Care and Use Committee (IACUC) of China Medical University (KT2019017).

### In Vivo Lymphatic Vessel Permeability Detection

2×10^6^ MGC803 cells in 0.2 mL PBS were subcutaneously injected into the right armpit region of BALB/c nude mice randomly divided into eight groups (*n* = 5 for each group). Two weeks later, PBS, CCL5 neutralization antibody (R&D Systems, MN), or TNF‐*α* neutralization antibody (R&D Systems, MN) were intratumorally injected every 2 days for 10 days. The mice were then injected intratumorally with 5% Evans Blue (Sigma, MA), then sacrificed 20 min later. Subcutaneous tumors were isolated, washed in PBS, then digested in 1 mL formamide at 60 °C for 48 h, while using 0.3 mL formamide to extract Evans Blue from the isolated axillary lymph nodes. A Tecan Infinite 200 Pro microplate reader (Tecan, Shanghai, China) was used to quantify the amount of extravasated Evans Blue dye at 630 nm.

### Plasmids and Transfection

The open reading frames (ORF) of CRIP1 and CREB1 were cloned into pCDNA3.1 vectors. Short hairpin RNAs (shRNA) were cloned into a lentivirus vector. All vectors were purchased from GenePharma (Shanghai, China). Transient transfections were performed using Lipofectamine 2000 Reagent (Invitrogen, CA) following the manufacturer's instruction. The concentrations for each transfection were 0.75 µg mL^−1^ plasmids with 2 mL culture medium in a six well cell culture plate. Cells were transfected with 5 × 10^6^ transducing units of lentivirus. G418 (100 µg mL^−1^) or puromycin (5 µg mL^−1^) were used to establish stable cell lines.

### CCK‐8 Proliferation Assay

The CCK‐8 (Dojindo Laboratories, Kumamoto, Japan) was used to measure capacity for cellular proliferation. After adding CCK‐8 to the medium, optical density was determined 1 h later by a SpectraMax Absorbance Reader (Molecular Devices, San Jose, CA) at a wavelength of 450 nm.

### Transwell Migration and Invasion Assays

For the transwell migration assay, cells were incubated in the upper compartment of the chamber (Corning, NY) for 24 h. For the invasion assay, Matrigel (BD Biosciences, San Jose, CA) was added into the upper compartment before introducing cells. After 24 h incubation at 37 °C with 5% CO_2_, the number of cells invading through the Matrigel was counted in 10 random regions from the central and peripheral sections of the filter using a Leica DM3000 microscope (Lecia, Wetzlar, Germany).

### EdU Assay

The EdU Assay was used to detect newly synthesized DNA to measure cell proliferation. After incubating cells in culture medium with EdU (Beyotime Biotechnology, Shanghai, China) for 1/10‐1/5 cell cycle, cells were fixed then stained with the BeyoClick EdU‐594 Kit (Beyotime Biotechnology, Shanghai, China) and DAPI. A Cytation 5 was used to obtain pictures.

### Colony‐Forming Assay

Cells at the exponential growth stage were dissociated and dispersed into a single cell suspension. After counting and adjusting cell concentrations, cells were inoculated in petri dishes for 2–3 weeks. When colonies were visible, the cells were fixed with methanol and stained with Giemsa Stain solution (G1015, Solarbio Science & Technology, Beijing, China) for 10 min. Colony‐forming units were counted to assess the proliferation ability of cells.

### RNA Isolation and RT‐qPCR

Total RNA was extracted by Trizol reagent (Invitrogen). Using the PrimeScript RT Reagent Kit with gDNA Eraser (Takara, Dalian, China) and gene‐specific primers, cDNA was generated via reverse transcription. RT‐qPCR was performed in a Light Cycler 480 II Real‐time PCR system (Roche Diagnostics, Basel, Switzerland) using TB Green Premix Ex Taq II (Takara, Dalian, China). *β*‐actin (ACTB) was used as an endogenous control for mRNA. The comparative Ct method was applied to calculate relative RNA expression. Primer sequences are displayed in Table S7, Supporting Information.

### Immunohistochemistry Staining

GC tissue microarray sections (4 µm) were heated at 60 °C for 2 h. After dewaxing, rehydration and antigen retrieval, tissue sections were blocked with 5% BSA and incubated with corresponding primary antibodies overnight at 4 °C then biotinylated secondary antibody for 30 min at 37 °C. Vector DAB Substrate Kit (Vector Laboratories, CA) was used to control the degree of chromogenic reaction. Tissue sections were then counterstained with hematoxylin and colored with lithium carbonate. Stained sections were scanned using a Pannoramic 250 FLASH Slide scanner. Antibodies used are indicated in Table S8, Supporting Information.

### Co‐IP

Cells lysates were prepared using NP‐40 lysis buffer (Keygen, Nanjing, China). Total proteins were incubated with primary antibody in rotation at 4 °C overnight. Magnetic protein A&G beads (Sigma, MA) were mixed with samples for 2 h at room temperature. After washing three times, the binding complex was eluted from the beads and SDS‐PAGE was used to separate proteins for western blot analysis.

### Western Blot Analysis

Total proteins were extracted via the total Protein Extraction Kit (KeyGen Biotech, Nanjing, China). Protein concentrations were quantified using the BCA Protein Assay Kit (Takara, Dalian, China). Proteins were separated via SDS‐polyacrylamide gel electrophoresis (SDS‐PAGE) and transferred to PVDF membranes (Millipore, MA). Membranes were incubated with primary antibodies overnight at 4 °C then secondary antibody for 1 h. Blots were detected using GelCapture software (DNR Bio‐Imaging Systems, Jerusalem, Israel). Primary antibodies, secondary antibodies, and their catalog numbers are listed in Table S8, Supporting Information.

### IF

Formalin‐fixed paraffin‐embedded (FFPE) sections were first dewaxed in dimethylbenzene then hydrated in ethanol along a concentration gradient. Antigen retrieval was performed in citric acid buffer under high pressure. Cultured cells inoculated in plates were fixed with methanol at −20 °C for 20 min. The sections or cells were blocked with 5% Bovine Serum Albumin (Sigma, MA) and incubated with primary antibodies overnight at 4 °C then secondary antibodies for 1 h. The primary antibodies, secondary antibodies, and their catalog numbers are listed in Table S8, Supporting Information. Finally, pictures were obtained via a Niko A1R confocal microscope system (Nikon Corporation, Tokyo, Japan).

### Flow Cytometry Analysis

Tumors were collected from tumor‐bearing nude mice and then were digested by Trypsin (Solarbio), Collagenase (Yeasen), Hyaluronidase (Yeasen), and DNase I (Beyotime). After digestion, the suspension was filtered through a 70 µm strainer to prepare the single‐cell suspension. Cells were stained with APC‐Cy7 Anti‐Mouse CD45 (BD Biosciences), PerCP/Cyanine5.5 antimouse/human CD11b (Biolegend), APC antimouse CD68 (Biolegend), and PE antimouse CD163 (Biolegend) for 30 min at 4 °C, protect from light. Cells were then washed and analyzed in a BD FACSCelesta Multicolor Flow Cytometer (BD Biosciences). Data were analyzed by FlowJo Software 10.8.1.

### Statistics

All statistical analysis was performed in the Statistical Package for the Social Sciences (SPSS) software 20.0 (IBM Corp, Armonk, NY) and GraphPad Prism software 8.0.0 (San Diego, CA). Student's *t‐*test was used to analyze variance between similar groups, while the Wilcoxon signed rank test was used in nonsimilar groups. Differences were considered statistically significant at *p*‐value < 0.05.

### Ethical Statement

All research complied with the principles of the Declaration of Helsinki, and approval was acquired from the Research Ethics Committee of the First Hospital of China Medical University.

## Conflict of Interest

The authors declare no conflict of interest.

## Author Contributions

Z.W., B.Q., M.Y., and J.L. contributed equally to this work. Conceptualization: Z.N.W., Y.X.S., Z.H.W., B.C.Q., M.X.Y., and J.J.L.; Methodology: Z.H.W., B.C.Q., M.X.Y., J.J.L., C.Z., and M.W.S.; Investigation: Y.Q.Z., Z.X.G., C.Z., and M.W.S.; Visualization: Y.Q.Z., Z.X.G., C.Z., and M.W.S.; Supervision: Z.N.W., Y.X.S., Z.H.W., B.C.Q.; Writing—original draft: Z.H.W., B.C.Q., M.X.Y., J.J.L.; Writing—review & editing: Z.N.W., Y.X.S., Z.H.W., B.C.Q., M.X.Y., and J.J.L.; All authors discussed the results and commented on the manuscript.

## Supporting information

Supporting Information

## Data Availability

The data that support the findings of this study are available in the supplementary material of this article.
