# Supplementary material for: CRIP1 Reshapes the Gastric Cancer Microenvironment to Facilitate Development of Lymphatic Metastasis
Source: Adv Sci (Weinh). 2023 Jul 6;10(26):2303246. doi: 10.1002/advs.202303246 (PMC10502640; doi:10.1002/advs.202303246)
Supplement: Supplementary file 1 — Supporting Information [file ADVS-10-2303246-s001.pdf]

ADVANCED SCIENCE
Open Access

Supporting Information

for *Adv. Sci.*, DOI 10.1002/advs.202303246

CRIP1 Reshapes the Gastric Cancer Microenvironment to Facilitate Development of Lymphatic Metastasis

Zhonghua Wu, Bicheng Qu, Minxian Yuan, Jingjing Liu, Cen Zhou, Mingwei Sun, Zhexu Guo, Yaqing Zhang, Yongxi Song and Zhenning Wang**

Supporting Information

CRIP1 reshapes the gastric cancer microenvironment to facilitate development of lymphatic metastasis

Authors: *Zhonghua Wu[1,†], Bicheng Qu[1,†], Minxian Yuan[1,†], Jingjing Liu[2,†], Cen Zhou[1], Mingwei Sun[1], Zhexu Guo[1], Yaqing Zhang[1], Yongxi Song[1,2,*], Zhenning Wang[1,*]*

This PDF file includes:

Figures S1-S9

Table S1- S8

Figure S1

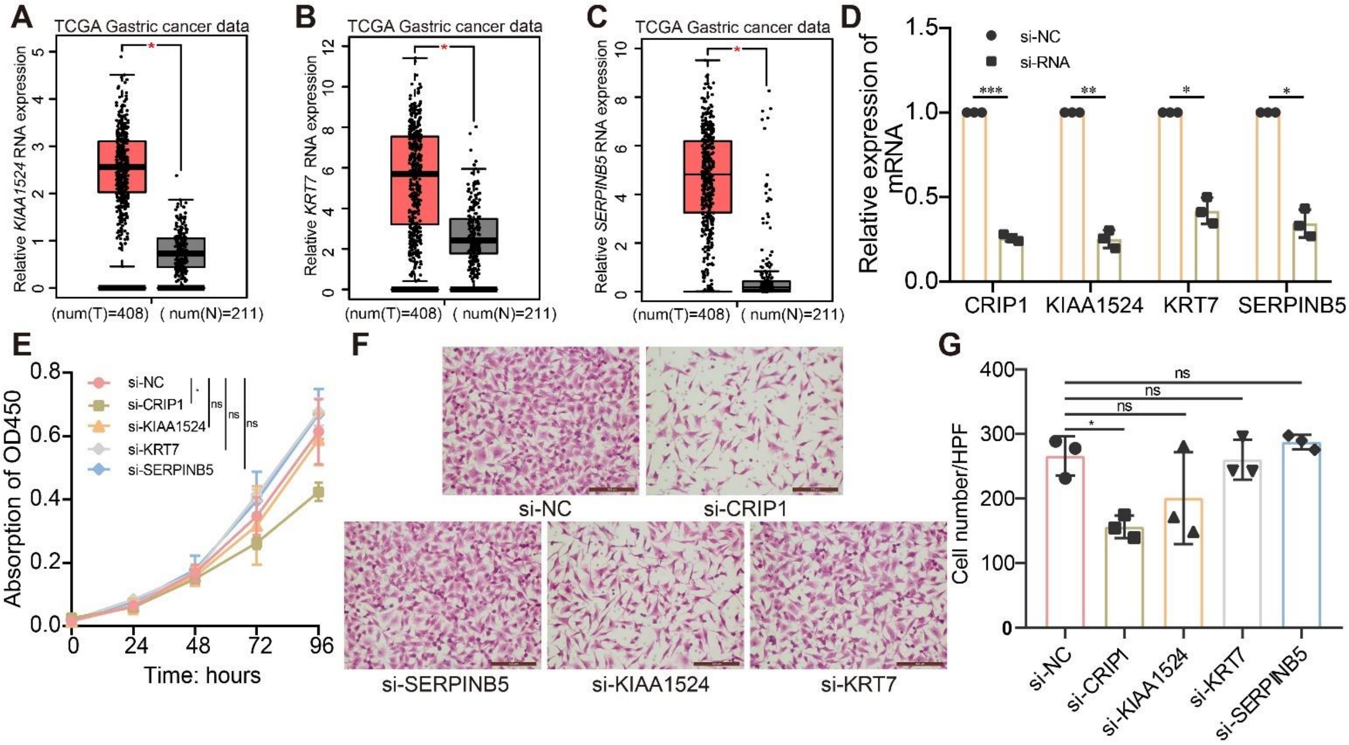

Figure S1. Knock down of CRIP1 inhibited GC cell proliferation and invasion. (A-C) The expression of *KIAA1524, KRT7* and *SERPINB5* in GC patients compared with controls were analyzed from TCGA database by GEPIA (http://gepia.cancerpku.cn/index. html). **(D)** RT-qPCR showing the effect of siRNA on CRIP1, KIAA1524, KRT7 and SERPINB5 mRNA expression. **(E)** CCK-8 assay showing the effect of CRIP1, KIAA1524, KRT7 or SERPINB5 knockdown on GC cells proliferation. **(F** and **G)** Transwell assay showing the effect of CRIP1, KIAA1524, KRT7 or SERPINB5 knockdown on GC cells migration. Error bars represent the mean±SD of three independent experiments. ns not significant, *P<0.05, **P<0.01, ***P<0.001.

Figure S2

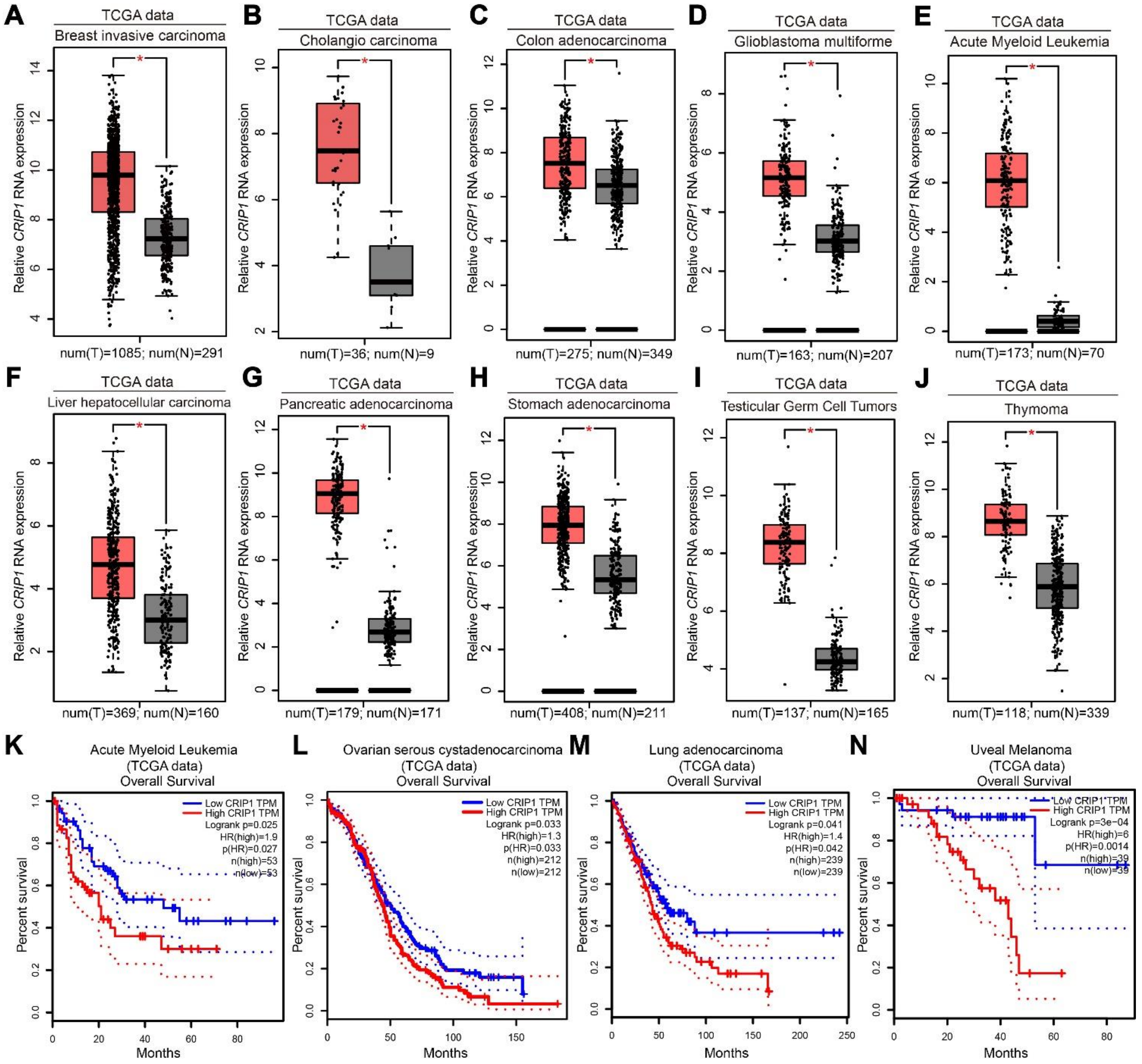

Figure S2. CRIP1 is upregulated in various types of human cancers and correlates with poor prognosis. (A-J) Analysis of TCGA data showed the expression of CRIP1 in various types of human cancers compared with corresponding control. **(K-N)** The overall survival rate of patients with low or high CRIP1 expression in various types of human cancers was analyzed with the Kaplan-Meier method. *P<0.05.

Figure S3

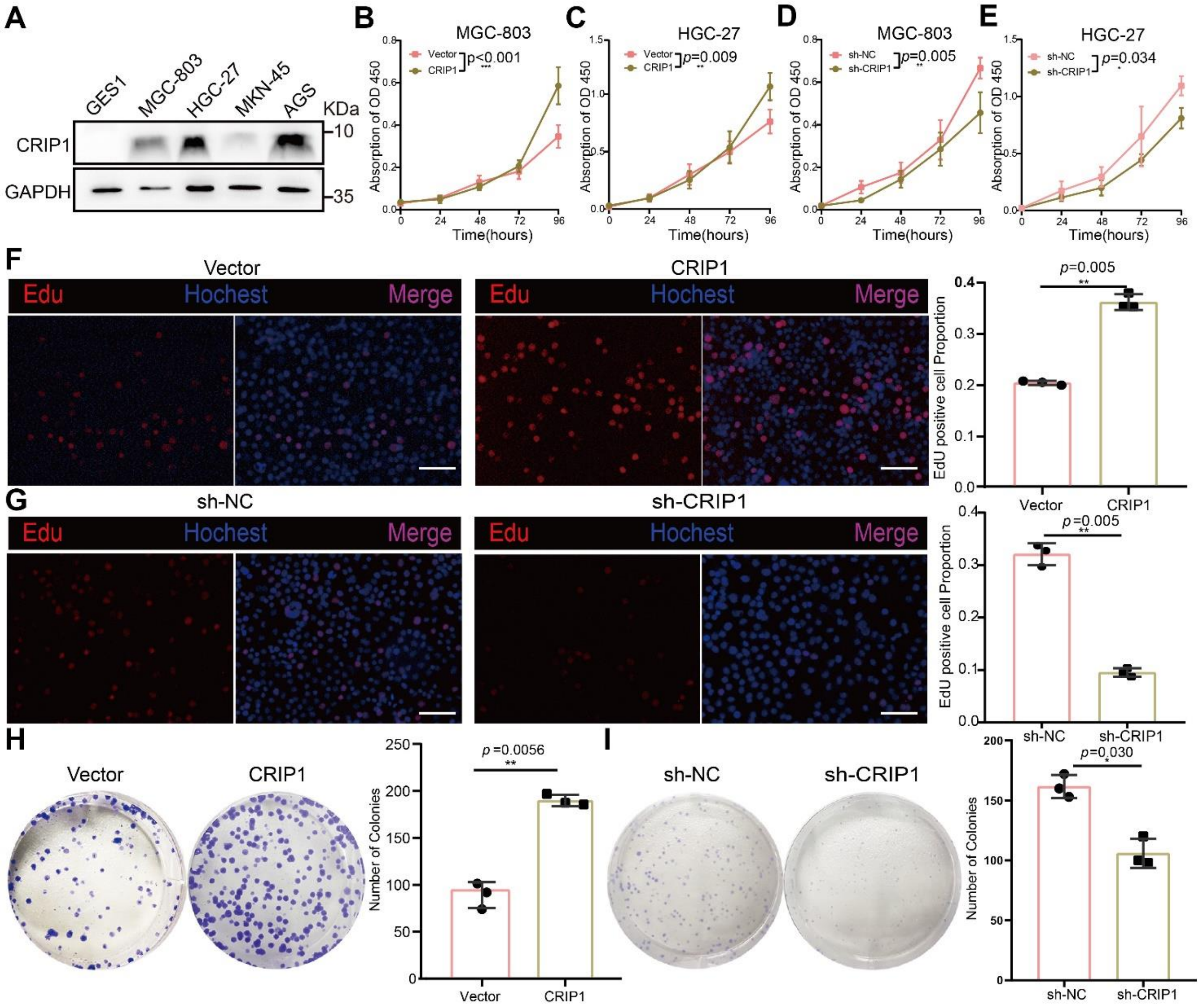

Figure S3. CRIP1 promoted GC cell proliferation *in vitro.* **(A)** Expression of CRIP1 in GES1 and GC cell lines. **(B** and **C)** CCK-8 assay showing the effect of CRIP1 overexpression on MGC-803 (B) and HGC-27 (C) cells proliferation. **(D** and **E)** CCK-8 assay showing the effect of CRIP1 knockdown on MGC-803 (D) and HGC-27 (E) cells proliferation. **(F** and **G)** Edu assay showing the effect of CRIP1 overexpression (F) and knockdown (G) on MGC-803 cells proliferation. The representative images of Edu assay were showed in left panels and the histogram showed the proportion of Edu positive cells. **(H** and **I)** Colony formation assay showing the effect of CRIP1 overexpression (H) and knockdown (I) on MGC-803 cells growth. Error bars represent the mean±SD of three independent experiments. *P<0.05, **P<0.01.

Figure S4

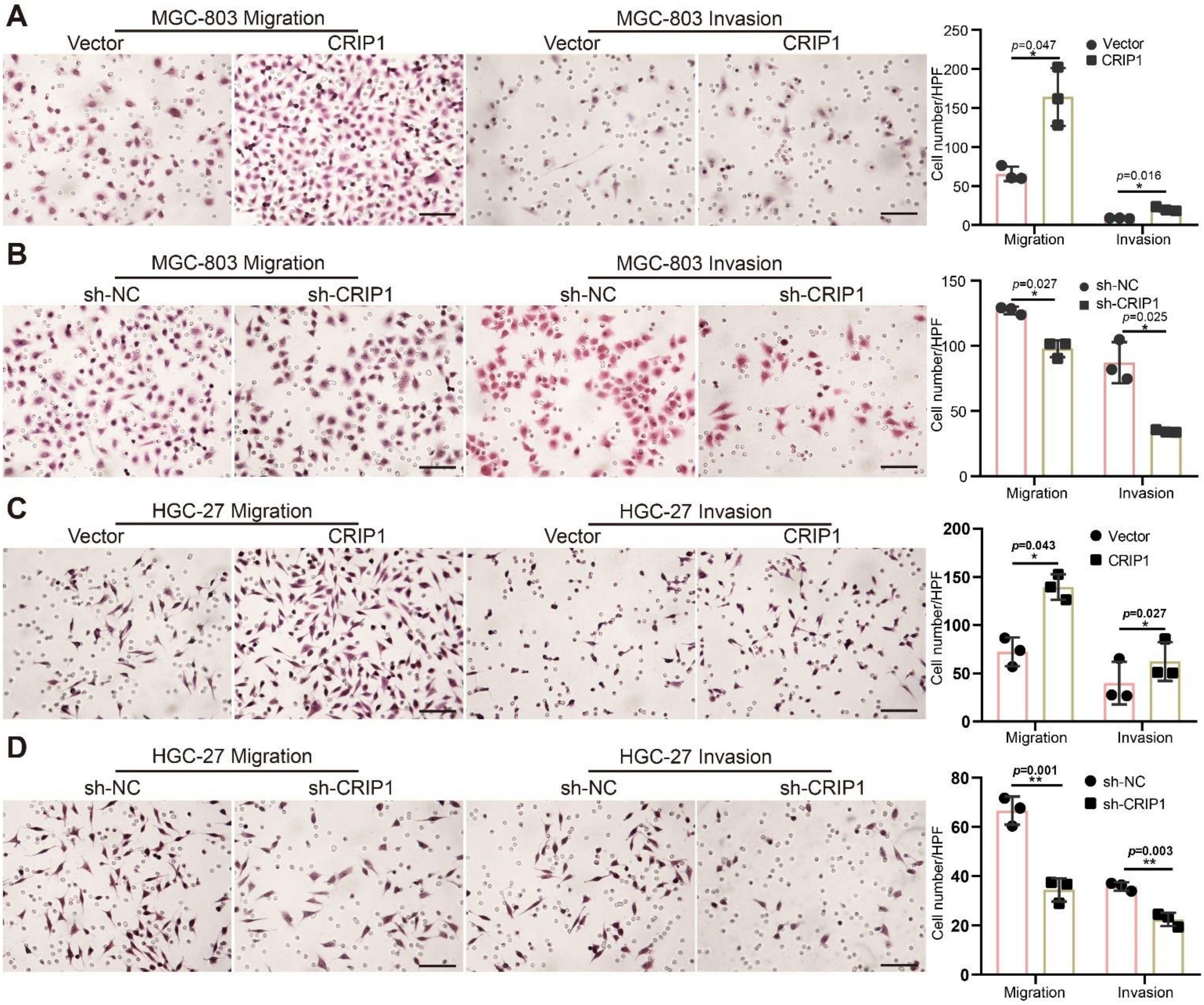

Figure S4. CRIP1 promoted GC cell migration and invasion *in vitro.* (**A**) Transwell migration and invasion assays showing the effect of CRIP1 overexpression on MGC-803 cells migrative and invasive capacity. (**B**) Transwell migration and invasion assays showing the effect of CRIP1 knockdown on MGC-803 cells migrative and invasive capacity. (**C**) Transwell migration and invasion assays showing the effect of CRIP1 overexpression on HGC-27 cells migrative and invasive capacity. (**D**) Transwell migration and invasion assays showing the effect of CRIP1 knockdown on HGC-27 cells migrative and invasive capacity. Scale bars=100μm. Error bars represent the mean±SD of three independent experiments. *P<0.05, **P<0.01.

Figure S5

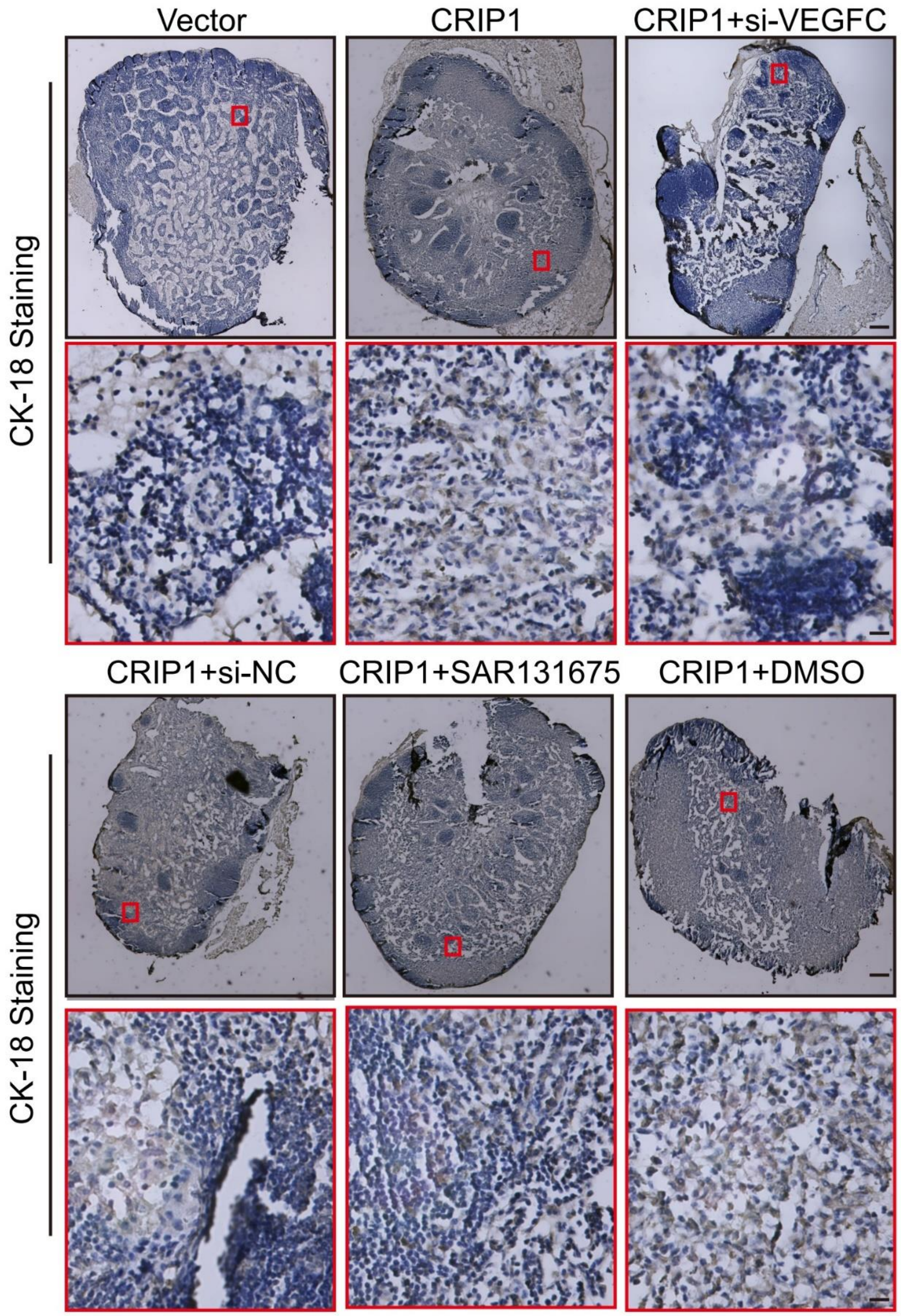

Figure S5. The representative immunohistochemistry images for lymph nodes isolated from popliteal LM model stained with cytokeratin (CK)-18. Scale bars=50μm.

Figure S6

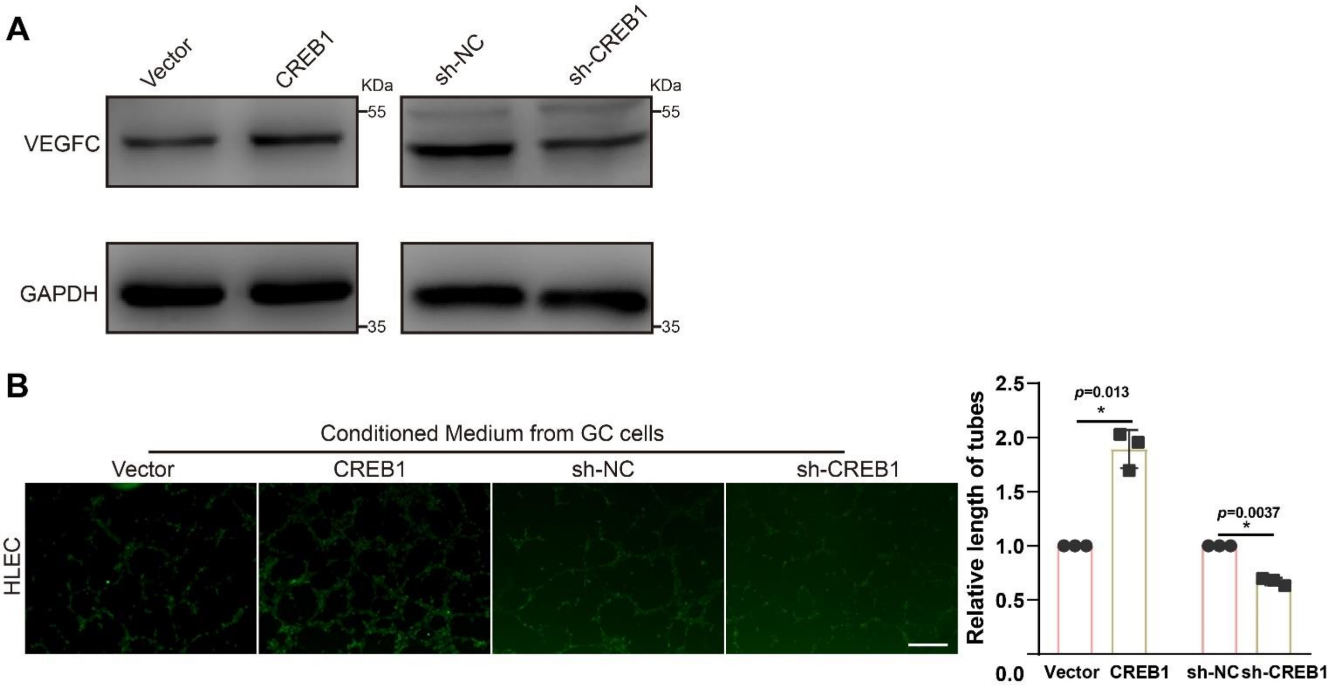

C

Groups	No. of Total LNs	No. of Metastatic LNs	Metastatic ratio (%)
Vector	15	3	20
CREB1	15	12	80

$p<0.001$ **

D

Groups	No. of Total LNs	No. of Metastatic LNs	Metastatic ratio (%)
sh-NC	15	6	40
sh-CREB1	15	1	7

$p=0.031$ *

Figure S6. CREB1 enhanced VEGFC secretion and promoted lymphangiogenesis and lymphatic metastasis. (A) Western blot revealed different expression of CCL5 in CREB1 overexpression or knockdown groups. **(B)** Representative images (left panel) and histogram quantification (right panel) of the Matrigel tube formation assay with human lymphatic endothelial cells (HLECs). HLECs were cultured with conditioned medium derived from GC cells that were treated as indicated. Scale bars=200μm. (**C** and **D)** The metastatic ratio of popliteal lymph nodes from mice inoculated with the indicated cells. Error bars represent the mean±SD of three independent experiments. *P<0.05.

Figure S7

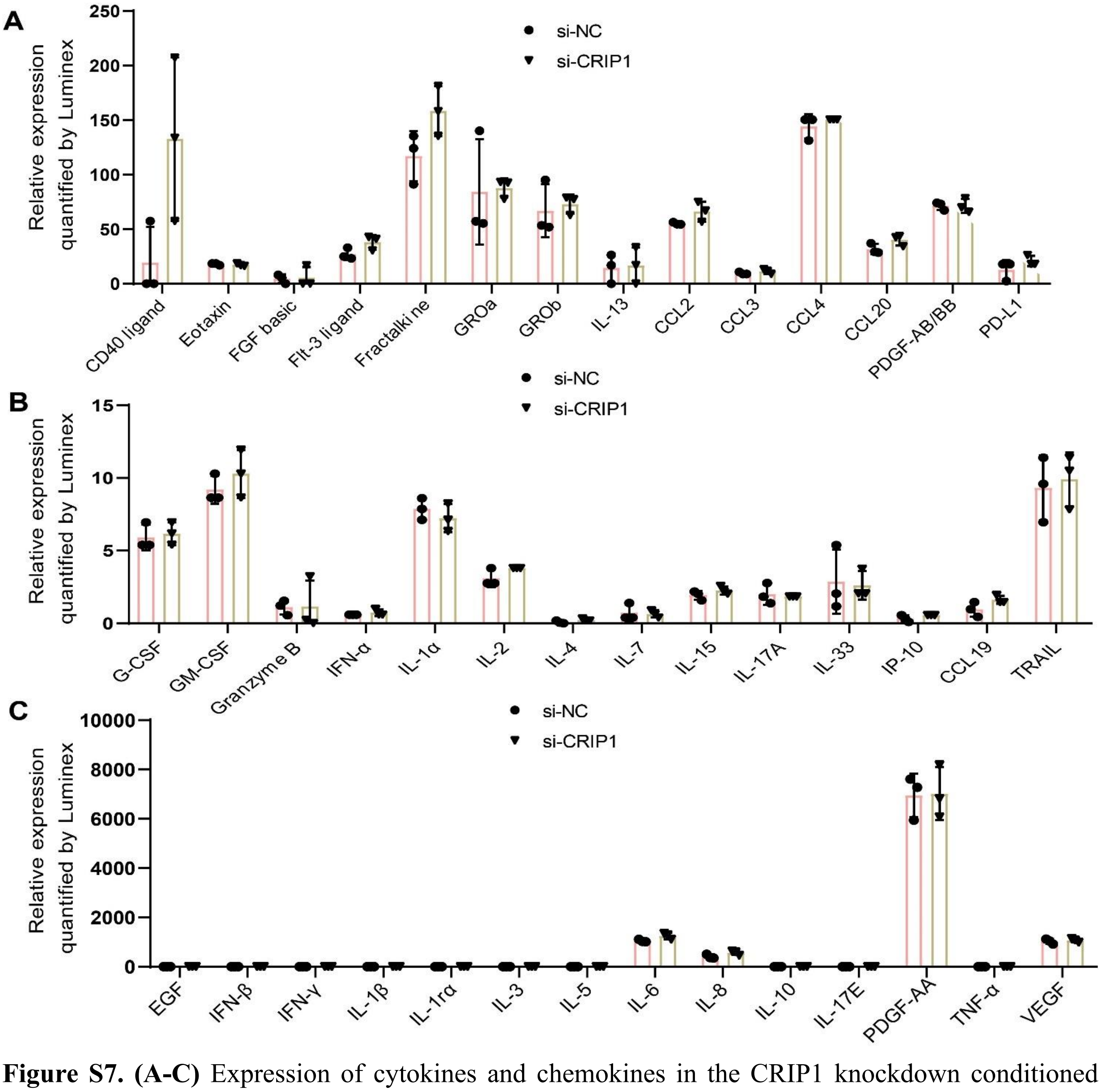

Figure S7. (A-C) Expression of cytokines and chemokines in the CRIP1 knockdown conditioned medium detected by Luminex. Error bars represent the mean±SD of three independent experiments.

Figure S8

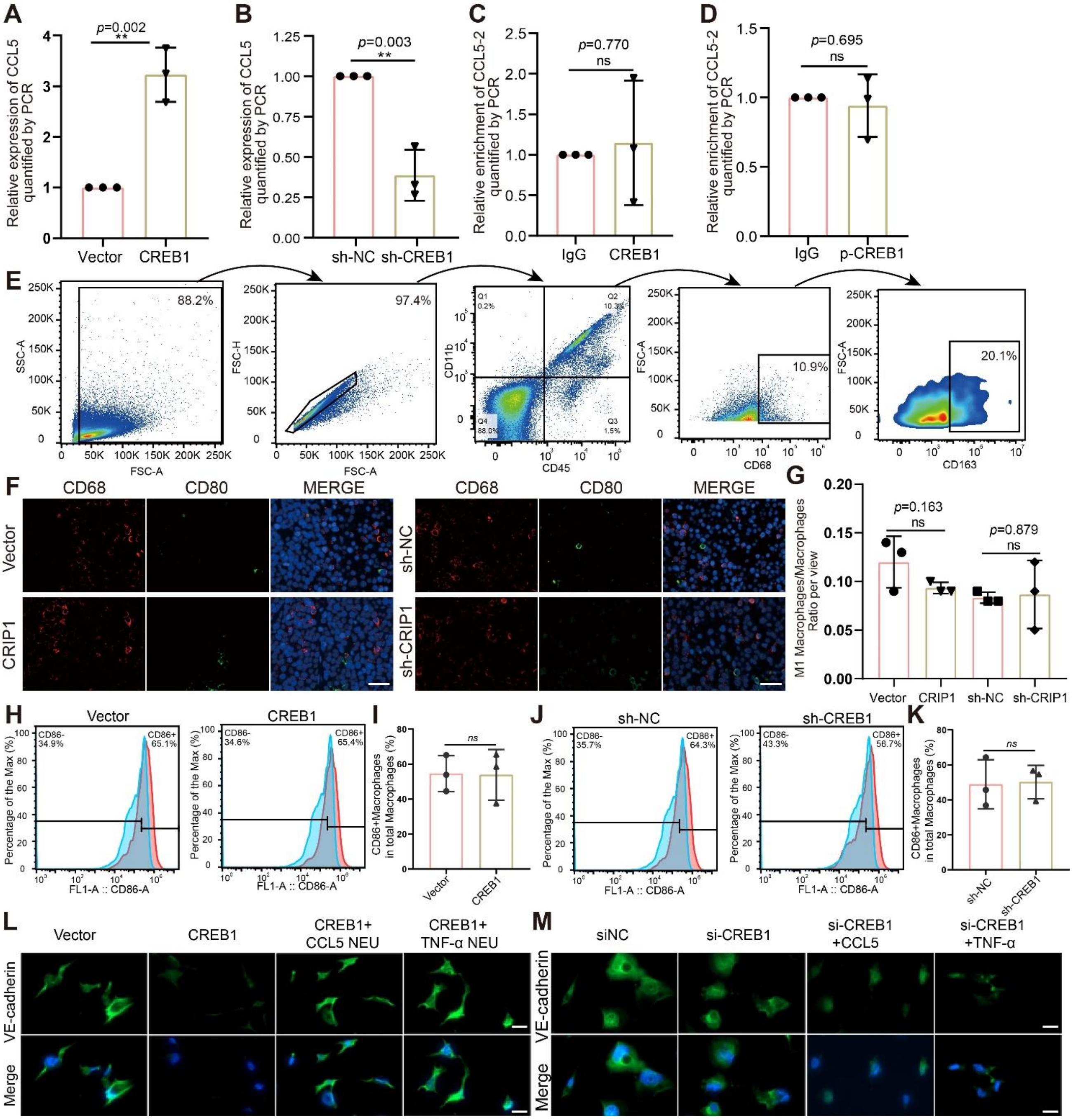

Figure S8. (A and B) Relative expression of CCL5 detected by RT-qPCR in CREB1 overexpression or knockdown groups. (**C** and **D**) ChIP was used to detect the interaction between CCL5-2 DNA and CREB1 or p-CREB1. (**E**) Gating strategies used for macrophage analysis in subcutaneous xenograft. **(F** and **G)** The recruitment of TAM was detected respectively by IF staining of CD68 and CD80 in CRIP1 overexpression or knockdown group. Scale bars=50μm. **(H-K)** Flow cytometry analysis showed percentage of M1 macrophages after co-cultured with CREB1 overexpression (H and I) or knockdown (J and K) supernatant. **(L** and **M)** The staining of VE-cadherin was detected by Immunofluorescence in HLEC co-culture with different supernatant. Scale bars=50μm. Error bars represent the mean±SD of three independent experiments, ns not significant.

Figure S9

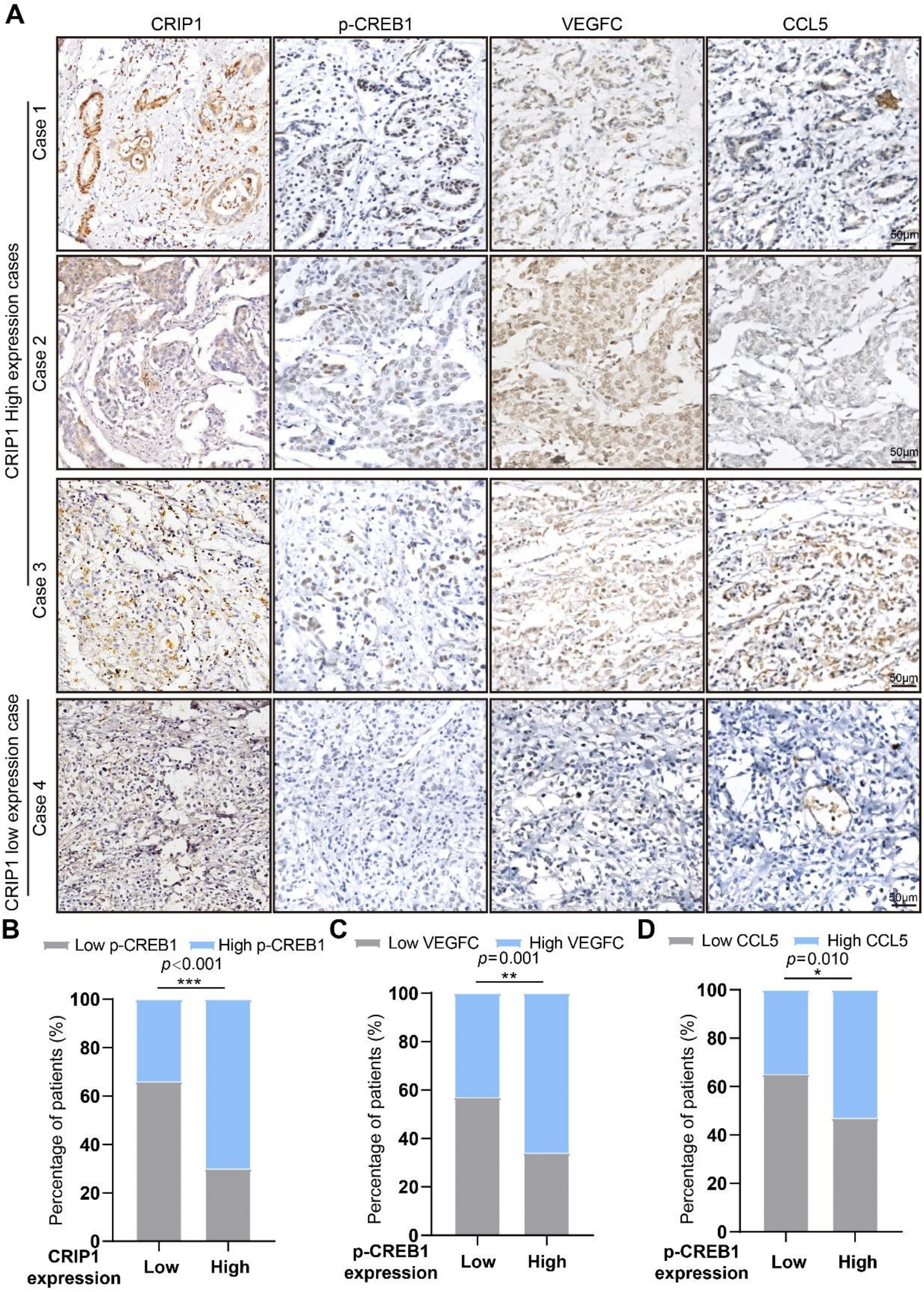

Figure S9. Verification of CRIP1-CREB1-VEGFC/CCL5 regulatory axis in clinical tissues. (**A**) The representative images of CRIP1, p-CREB1, VEGFC and CCL5 in human gastric cancer tissues. (**B**) Analysis of p-CREB1 expression relative to CRIP1 expression across gastric cancer patients. (**C**) Analysis of VEGFC expression relative to p-CREB1 expression across gastric cancer patients. (**D**) Analysis of CCL5 expression relative to p-CREB1 expression across gastric cancer patients. Statistical significance was assessed by χ-square test. *P<0.05, **p<0.01, ***P<0.001.

Table S1. clinicopathological characteristics of 10 gastric cancer patients

Specimen NO.	Age (years)	Gender (male/female)	Borrmann type	T stage	N stage	M stage	TNM stage
**NO.1**	67	F	Borrmann 3	T4a	N3b	M0	IIIC
**NO.2**	65	F	Borrmann 3	T4a	N2	M0	IIIB
**NO.3**	54	M	Borrmann 3	T4a	N2	M0	IIIB
**NO.4**	47	M	Borrmann 3	T3	N1	M0	IIB
**NO.5**	59	M	Borrmann 4	T3	N1	M0	IIB
**NO.6**	52	F	Borrmann 3	T4a	N0	M0	IIB
**NO.7**	56	M	Borrmann 3	T4a	N0	M0	IIB
**NO.8**	52	M	Borrmann 3	T4a	N0	M0	IIB
**NO.9**	47	F	Borrmann 3	T3	N0	M0	IIA
**NO.10**	53	M	Borrmann 2	T2	N0	M0	IB

Note: the TNM stage were staged according to the eighth TNM staging of the International Union against Cancer (UICC)/American Joint Committee on Cancer (AJCC) system.

Table S2 Differentially expressed mRNAs between gastric cancer and paired non-tumorous adjacent tissues

ProbeName	P-value	FDR	Fold Change	Regulation	type	seqname	GeneSymbol
ASHGV40018476	0.009489597	0.097753962	2.1025617	down	protein_coding	NM_001003938	HBM
ASHGV40030259	0.004486198	0.071810531	12.3583347	down	protein_coding	NM_001632	ALPP
ASHGV40046224	0.010751644	0.103400812	2.6616245	down	protein_coding	NM_152774	TMEM196
ASHGV40040097	0.011047527	0.104460239	2.2454351	down	protein_coding	NM_030945	C1QTNF3
ASHGV40006160	0.002414844	0.059482588	2.6627091	down	protein_coding	NM_001008723	CFAP58
ASHGV40044581	0.010509107	0.102456293	2.8609282	down	protein_coding	NM_001491	GCNT2
ASHGV40057746	0.027891237	0.161162524	2.1499355	down	protein_coding	NM_001190702	MTRNR2L8
ASHGV40018484	0.002937363	0.061986011	12.3700601	down	protein_coding	NM_006849	PDIA2
ASHGV40004889	0.004044138	0.069284044	3.7385332	down	protein_coding	NM_052918	SORCS1
ASHGV40048385	0.000371685	0.039736903	9.3173849	down	protein_coding	NM_002851	PTPRZ1
ASHGV40027791	0.000901492	0.049204994	5.0847986	down	protein_coding	NM_002500	NEUROD1
ASHGV40033814	0.003794674	0.067881807	2.2152191	down	protein_coding	NM_025225	PNPLA3
ASHGV40018455	0.03993111	0.193433788	2.1790376	down	protein_coding	NM_138795	ARL8A
ASHGV40013879	0.003806057	0.067881807	2.3166521	down	protein_coding	NM_001836	CMA1
ASHGV40033459	0.042357702	0.199891315	3.5969989	down	protein_coding	NM_182948	PRKACB
ASHGV40030524	6.81381E-05	0.028718686	8.6705888	down	protein_coding	NM_020341	PAK7
ASHGV40030791	0.008139862	0.090920324	2.6290885	down	protein_coding	NM_178134	CYP4Z1
ASHGV40035755	0.001056249	0.049811075	5.7781833	down	protein_coding	NM_015141	GPD1L
ASHGV40021758	0.008856119	0.094675221	2.6561874	down	protein_coding	NM_009590	AOC2
ASHGV40038549	0.037606403	0.186916924	2.5558874	down	protein_coding	NM_020773	TBC1D14
ASHGV40033282	0.031196689	0.170170948	2.1502797	down	protein_coding	NM_006071	PKDREJ
ASHGV40005423	0.001788866	0.055586762	2.5824902	down	protein_coding	NM_000818	GAD2
ASHGV40016083	0.000170033	0.030140601	12.5907075	down	protein_coding	NM_001102658	CT62
ASHGV40054729	0.001396149	0.052512256	2.1525326	down	protein_coding	NM_004726	REPS2
ASHGV40035722	0.00291455	0.061986011	389.1344373	down	protein_coding	NM_201653	CHIA
ASHGV40015141	0.002483683	0.060063095	2.1491763	down	protein_coding	NM_020692	GALNT16
ASHGV40036198	0.003326655	0.064680656	16.1457924	down	protein_coding	NM_001167674	CADM2
ASHGV40028658	0.003807548	0.067881807	3.6152024	down	protein_coding	ENST0000044716	AC109829.1
ASHGV40032129	0.013032576	0.112548783	12.5431722	down	protein_coding	NM_014495	ANGPTL3
ASHGV40044102	0.003733424	0.067544862	17.904718	down	protein_coding	NM_145176	SLC2A12
ASHGV40041045	0.024451856	0.151723334	2.0940046	down	protein_coding	NM_032385	FAXDC2
ASHGV40049900	0.013205683	0.11335758	2.4295446	down	protein_coding	NM_014751	MTSS1
ASHGV40026034	0.021379631	0.144598619	2.4542131	down	protein_coding	NM_145814	CACNG6
ASHGV40026080	0.00101713	0.049811075	8.0124037	down	protein_coding	NM_020378	NAT14
ASHGV40016254	0.004352806	0.071084094	2.6376832	down	protein_coding	NM_004644	AP3B2
ASHGV40039043	0.009681789	0.098547388	10.6939829	down	protein_coding	NM_000477	ALB
ASHGV40007032	0.002279738	0.058369326	83.3742013	down	protein_coding	NM_005142	GIF
ASHGV40038720	0.00137495	0.052512256	2.3202369	down	protein_coding	NM_005349	RBPJ
ASHGV40044747	0.004074945	0.069284044	5.0064442	down	protein_coding	NM_006995	BTN2A2
ASHGV40055152	0.000193109	0.030890283	3.3652658	down	protein_coding	NM_053281	DACH2
ASHGV40037525	0.003404001	0.064805109	5.2232557	down	protein_coding	NM_021114	SPINK2
ASHGV40029529	0.004532969	0.071864614	2.1137647	down	protein_coding	NM_001304449	ZNF806
ASHGV40008714	0.003527061	0.065507718	50.4029074	down	protein_coding	NM_020811	CARNS1
ASHGV40009123	0.015592344	0.123222273	2.8714364	down	protein_coding	NM_015191	SIK2
ASHGV40010911	0.032841255	0.17502601	2.5461014	down	protein_coding	NM_018463	ITFG2
ASHGV40052165	0.004664204	0.07280668	3.3206322	down	protein_coding	NM_019114	EPB41L4B
ASHGV40016348	0.002734828	0.061986011	2.4775416	down	protein_coding	ENST0000041279	GABARAPL3
ASHGV40030698	0.026267275	0.156959315	2.1497695	down	protein_coding	NM_032501	ACSS1
ASHGV40035092	0.001532455	0.053801741	4.8222189	down	protein_coding	NM_174878	CLRN1
ASHGV40022552	0.014595299	0.118973184	2.4845566	down	protein_coding	NM_145287	ZNF519
ASHGV40025980	0.000806367	0.048770305	7.7644905	down	protein_coding	uc001ayv.2	AX747988
ASHGV40030110	0.011091027	0.104667783	5.5954061	down	protein_coding	NM_000597	IGFBP2
ASHGV40008692	0.000875734	0.049097415	3.0879028	down	protein_coding	NM_005125	CCS
ASHGV40005952	0.035372316	0.181042365	3.2357544	down	protein_coding	NM_001102469	LIPN
ASHGV40025260	0.029064684	0.164407506	2.2756666	down	protein_coding	NM_002741	PKN1
ASHGV40000107	0.006326722	0.082097034	2.1310862	down	protein_coding	uc010oyb.2	LOC100288142
ASHGV40048965	0.001146919	0.050016538	2.3192477	down	protein_coding	NM_015310	PSD3
ASHGV40014293	0.001396849	0.052512256	3.6117221	down	protein_coding	NM_005589	ALDH6A1

ASHGV40024482	0.002288578	0.058473339	2.1790315	down	protein_coding	NM_001033719	ZNF404
ASHGV40012456	0.001428822	0.052558935	2.7348784	down	protein_coding	NM_001144981	CCDC169
ASHGV40011885	0.00404128	0.069284044	158.0468334	down	protein_coding	NM_139319	SLC17A8
ASHGV40045336	0.000384263	0.039736903	4.4135474	down	protein_coding	NM_001009994	RIPPLY2
ASHGV40036575	0.019366162	0.137096387	6.087853	down	protein_coding	NM_016369	CLDN18
ASHGV40029080	0.039389483	0.19221803	5.3729056	down	protein_coding	NM_002909	REG1A
ASHGV40029873	0.001480484	0.053005575	5.7795879	down	protein_coding	NM_173651	FSIP2
ASHGV40007685	0.002464105	0.059755395	5.8340136	down	protein_coding	NM_138971	BACE1
ASHGV40042662	8.11754E-07	0.008015752	7.2618561	down	protein_coding	NM_000806	GABRA1
ASHGV40006641	0.001725381	0.055333849	5.1350864	down	protein_coding	NM_020974	SCUBE2
ASHGV40037205	0.00908225	0.096149426	19.0058992	down	protein_coding	NM_001085382	PSAPL1
ASHGV40022190	0.011572447	0.106869002	3.4662382	down	protein_coding	NM_018653	GPRC5C
ASHGV40040815	0.038033066	0.18823291	2.5100293	down	protein_coding	NM_015082	FSTL4
ASHGV40024749	0.002152177	0.056552005	2.5796517	down	protein_coding	NM_018355	ZNF415
ASHGV40038276	0.005469947	0.077372027	4.3810929	down	protein_coding	NM_006818	MLLT11
ASHGV40038500	0.008797118	0.09433367	4.4461148	down	protein_coding	NM_001528	HGFAC
ASHGV40048232	0.004392802	0.071448595	3.2570957	down	protein_coding	NM_033506	FBXO24
ASHGV40015221	0.012351734	0.109722298	3.5172409	down	protein_coding	NM_004452	ESRRB
ASHGV40010346	0.0009481	0.049736021	2.7542014	down	protein_coding	NM_013244	MGAT4C
ASHGV40047387	0.001918233	0.055772948	3.6515841	down	protein_coding	NM_002847	PTPRN2
ASHGV40057166	0.001914908	0.055772948	2.1428105	down	protein_coding	NM_030613	ZFP2
ASHGV40036104	0.002641299	0.061734161	2.867999	down	protein_coding	NM_000248	MITF
ASHGV40051624	0.000184283	0.030140601	11.5078218	down	protein_coding	NM_152570	LINGO2
ASHGV40006196	0.001121787	0.049896617	2.2727166	down	protein_coding	NM_014456	PDCD4
ASHGV40056122	0.001441088	0.052724506	4.1102078	down	protein_coding	NM_001271507	CCDC177
ASHGV40054481	0.01984402	0.138920246	3.4933021	down	protein_coding	NM_005369	MCF2
ASHGV40029885	0.029868717	0.166580805	2.5405286	down	protein_coding	NM_016315	GULP1
ASHGV40043201	0.002913081	0.061986011	2.733148	down	protein_coding	NM_001010877	ZNF311
ASHGV40025883	0.044714988	0.205153987	3.0377931	down	protein_coding	NM_000085	CLCNKB
ASHGV40012711	0.028358718	0.162598688	2.1361239	down	protein_coding	NM_007249	KLF12
ASHGV40013839	0.002019746	0.055772948	2.8465389	down	protein_coding	NM_012244	SLC7A8
ASHGV40048402	0.004580619	0.072278436	5.5866352	down	protein_coding	NM_000740	CHRM3
ASHGV40040117	0.035686781	0.181859659	2.1014547	down	protein_coding	uc010iux.3	NADKD1
ASHGV40041689	0.031151951	0.170111314	3.0505648	down	protein_coding	NM_012343	NNT
ASHGV40039490	0.002815916	0.061986011	4.0758277	down	protein_coding	NM_181885	RXFP4
ASHGV40020284	0.002860823	0.061986011	2.6321197	down	protein_coding	NM_152349	KRT222
ASHGV40024443	0.046769474	0.209075944	3.132082	down	protein_coding	NM_032488	CNFN
ASHGV40008893	0.02588868	0.155887709	3.1635614	down	protein_coding	NM_006189	OMP
ASHGV40022230	0.0182988	0.133293579	2.6743415	down	protein_coding	NM_001162997	SMIM6
ASHGV40023269	0.024376866	0.151714606	2.0464968	down	protein_coding	NM_000371	TTR
ASHGV40019861	0.037029594	0.185191074	2.1895868	down	protein_coding	NM_003802	MYH13
ASHGV40047318	0.009428777	0.097468969	3.9170977	down	protein_coding	NM_198285	WDR86
ASHGV40035980	0.012056388	0.108852935	2.1664121	down	protein_coding	NM_000839	GRM2
ASHGV40025742	0.010797231	0.103530481	2.6986303	down	protein_coding	NM_022752	ZNF574
ASHGV40007958	0.005390001	0.076906366	2.5182542	down	protein_coding	uc001luy.2	AK126380
ASHGV40008059	0.011990156	0.108703513	2.2913421	down	protein_coding	NM_003621	PPFIBP2
ASHGV40009126	0.003964025	0.068946524	4.2157602	down	protein_coding	NM_001541	HSPB2
ASHGV40037315	0.00274857	0.061986011	2.917315	down	protein_coding	NM_001278141	NBPF12
ASHGV40053537	0.005750858	0.079177486	16.3768267	down	protein_coding	NM_001291815	HMCN2
ASHGV40015213	0.001306739	0.051782513	5.8238385	down	protein_coding	NM_031281	FCRL5
ASHGV40052021	0.000713912	0.046533174	5.1261507	down	protein_coding	NM_003837	FBP2
ASHGV40016367	0.000753338	0.047440361	2.2021715	down	protein_coding	NM_207446	FAM174B
ASHGV40031696	0.034310433	0.178694547	4.1592199	down	protein_coding	NM_006252	PRKAA2
ASHGV40014267	0.029907716	0.166580805	2.2620642	down	protein_coding	NM_001014342	FLG2
ASHGV40021934	0.01347553	0.114242293	2.6941547	down	protein_coding	NM_022469	GREM2
ASHGV40044386	0.009429933	0.097468969	2.1704862	down	protein_coding	NM_016098	MPC1
ASHGV40035290	0.02660443	0.15791087	2.3941597	down	protein_coding	NM_002885	RAP1GAP
ASHGV40017707	0.005899193	0.080032877	2.2463069	down	protein_coding	NM_182617	ACSM2B
ASHGV40057615	0.007689377	0.088966144	4.7454914	down	protein_coding	NM_002565	P2RY4
ASHGV40040297	0.003234004	0.064150273	3.1796231	down	protein_coding	NM_004983	KCNJ9
ASHGV40009100	0.004946396	0.074157667	2.6316738	down	protein_coding	NM_000019	ACAT1

ASHGV40037462	0.003664827	0.067051334	2.4946309	down	protein_coding	NM_173536	GABRG1
ASHGV40040354	0.001170391	0.050017015	5.5181353	down	protein_coding	NM_144699	ATP1A4
ASHGV40044643	0.013426126	0.114197014	2.7334242	down	protein_coding	NM_006877	GMPR
ASHGV40032149	0.002247923	0.058034571	3.7555289	down	protein_coding	NM_001128598	KRTAP25-1
ASHGV40007174	0.017728779	0.130981013	5.878594	down	protein_coding	NM_138368	AP5B1
ASHGV40050028	0.000761832	0.047440361	16.613746	down	protein_coding	NM_001160372	TRAPPC9
ASHGV40041296	0.003819399	0.067888222	2.1274858	down	protein_coding	NM_001258283	OR2V1
ASHGV40043020	0.009664894	0.09843288	2.3189442	down	protein_coding	NM_032744	ADTRP
ASHGV40036067	0.005060816	0.07484092	2.1863263	down	protein_coding	NM_001080537	SNTN
ASHGV40017334	0.002559529	0.060670246	3.453791	down	protein_coding	ENST0000060079	AC087477.1
ASHGV40020510	0.001911531	0.055772948	2.7713629	down	protein_coding	NM_032391	PRAC1
ASHGV40041293	0.017923735	0.131913417	2.4014385	down	protein_coding	NM_017542	POGK
ASHGV40015131	0.016248143	0.125739087	5.3125712	down	protein_coding	NM_152443	RDH12
ASHGV40051423	0.001660835	0.054739049	4.371813	down	protein_coding	NM_014665	LRRC14
ASHGV40048119	0.003954049	0.068946524	2.1615083	down	protein_coding	NM_001287135	CDK14
ASHGV40049072	0.004575738	0.072266824	2.7735034	down	protein_coding	NM_001394	DUSP4
ASHGV40055273	0.025933154	0.155887709	3.0354943	down	protein_coding	NM_182607	VSIG1
ASHGV40011946	0.008827174	0.09446094	3.5787275	down	protein_coding	NM_203436	ASCL4
ASHGV40005770	0.020082086	0.139836761	2.9953163	down	protein_coding	NM_018344	SLC29A3
ASHGV40046175	0.000399026	0.03988564	7.0607889	down	protein_coding	NM_001017425	KCNK2
ASHGV40022235	0.014078252	0.11697786	2.7061123	down	protein_coding	NM_001080419	UNK
ASHGV40021927	0.002058303	0.055772948	2.1412544	down	protein_coding	NM_025149	ACSF2
ASHGV40035280	0.001478056	0.053005575	2.4649201	down	protein_coding	NM_001040709	SYPL2
ASHGV40026559	0.003566086	0.065866822	3.7850126	down	protein_coding	NM_144575	CAPN13
ASHGV40051527	0.005895793	0.080032877	2.0032959	down	protein_coding	NM_020824	ARHGAP21
ASHGV40009146	0.002153621	0.056552005	3.7485691	down	protein_coding	NM_181351	NCAM1
ASHGV40025241	0.005174853	0.075886235	3.9951991	down	protein_coding	NM_014047	C19orf53
ASHGV40028975	0.021738916	0.145767282	9.2455773	down	protein_coding	NM_019617	GKN1
ASHGV40013288	0.007875419	0.089971932	2.2761702	down	protein_coding	uc021oww.1	DQ786323
ASHGV40053883	0.010342882	0.101599149	2.7315769	down	protein_coding	NM_004015	DMD
ASHGV40031299	0.034659024	0.179356685	2.965722	down	protein_coding	NM_182797	PLCB4
ASHGV40048396	0.023457494	0.149727141	4.5471768	down	protein_coding	NM_207163	LMOD2
ASHGV40020674	0.003702715	0.067298943	11.8088263	down	protein_coding	NM_152598	MARCH10
ASHGV40030922	0.012590924	0.11054147	3.1157182	down	protein_coding	NM_018102	ZNF334
ASHGV40029662	0.024536179	0.152054281	6.602422	down	protein_coding	NM_007102	GUCA2B
ASHGV40053062	0.002436159	0.059482588	3.9678973	down	protein_coding	NM_001001709	C9orf170
ASHGV40014451	0.012091824	0.108901362	2.198582	down	protein_coding	NM_004755	RPS6KA5
ASHGV40038156	0.008708696	0.093650627	16.5446326	down	protein_coding	NM_021870	FGG
ASHGV40025273	0.000807544	0.048770305	4.7050719	down	protein_coding	NM_012114	CASP14
ASHGV40007591	0.009603283	0.098103745	2.0146438	down	protein_coding	NM_152587	C11orf65
ASHGV40014967	0.010947227	0.104057649	2.0752697	down	protein_coding	NM_172193	KLHDC1
ASHGV40038594	0.007274107	0.08639736	36.7507674	down	protein_coding	NM_000798	DRD5
ASHGV40021251	0.000620942	0.045111426	9.1354619	down	protein_coding	ENST0000039941	AC129492.6
ASHGV40013189	0.002797715	0.061986011	2.7301237	down	protein_coding	NM_004795	KL
ASHGV40048676	0.003001159	0.062593553	7.2952871	down	protein_coding	NM_000603	NOS3
ASHGV40014816	0.007045346	0.085147057	2.0238644	down	protein_coding	NM_174944	TSSK4
ASHGV40050811	0.000196304	0.031004988	2.6912619	down	protein_coding	NM_004770	KCNB2
ASHGV40011120	0.006740739	0.083835464	15.279364	down	protein_coding	NM_004570	PIK3C2G
ASHGV40044967	0.001168967	0.050017015	2.9171372	down	protein_coding	NM_182548	LHFPL5
ASHGV40038370	0.000244665	0.03332801	5.964847	down	protein_coding	NM_152775	CCDC110
ASHGV40010277	0.026906139	0.158931032	2.1307757	down	protein_coding	NM_032606	CAPS2
ASHGV40047616	0.000316128	0.03643396	8.0845178	down	protein_coding	NM_000905	NPY
ASHGV40018700	0.005230064	0.076092721	5.7860356	down	protein_coding	NM_152308	RMI2
ASHGV40020761	0.004803952	0.073532246	3.1267348	down	protein_coding	NM_017565	FAM20A
ASHGV40021502	0.002095013	0.055911749	6.6958481	down	protein_coding	NM_178170	NEK8
ASHGV40022141	0.000590434	0.044687591	4.7776583	down	protein_coding	NM_001270422	KCNJ16
ASHGV40016128	0.001465631	0.052900205	3.723252	down	protein_coding	NM_006917	RXRG
ASHGV40036651	0.030901369	0.169417437	2.5195869	down	protein_coding	NM_001871	CPB1
ASHGV40050364	0.002591818	0.061068834	6.6963393	down	protein_coding	NM_001013842	C8orf58
ASHGV40051142	0.00473812	0.073434545	2.620242	down	protein_coding	NM_022783	DEPTOR
ASHGV40032623	0.010921871	0.104057649	7.8383028	down	protein_coding	NM_053277	CLIC6

ASHGV40055807	0.01115955	0.104993631	3.8479143	down	protein_coding	uc001kgl.3	SLC16A12
ASHGV40017015	0.000106605	0.028718686	5.0830225	down	protein_coding	NM_014249	NR2E3
ASHGV40055219	0.000403551	0.039979068	6.8248737	down	protein_coding	NM_080390	TCEAL2
ASHGV40025351	0.015013047	0.121046845	3.2549387	down	protein_coding	NM_021933	MIIP
ASHGV40002601	0.000733847	0.046846037	4.8870555	down	protein_coding	ENST0000059466	TD-2521M24.1
ASHGV40031645	0.007248325	0.086267434	2.0429284	down	protein_coding	NM_001098798	TOX2
ASHGV40036561	0.00071083	0.046533174	4.4904231	down	protein_coding	NM_002718	PPP2R3A
ASHGV40024518	0.00064229	0.045710101	26.049751	down	protein_coding	NM_001824	CKM
ASHGV40014138	0.001047263	0.049811075	2.0921177	down	protein_coding	NM_021136	RTN1
ASHGV40023810	0.002943589	0.061986011	3.543748	down	protein_coding	NM_174895	PCP2
ASHGV40001585	0.030634444	0.168658722	2.9882363	down	protein_coding	ENST0000051060	CTD-2228K2.5
ASHGV40025651	0.002811607	0.061986011	6.7749641	down	protein_coding	NM_021185	CATSPERG
ASHGV40030185	0.000110854	0.028718686	4.5900556	down	protein_coding	NM_058165	MOGAT1
ASHGV40056612	0.00915432	0.096506793	2.2856986	down	protein_coding	NM_024584	CCDC121
ASHGV40018137	0.04261619	0.200285477	3.198706	down	protein_coding	NM_178818	CMTM4
ASHGV40018784	0.005218517	0.076078653	3.2194169	down	protein_coding	NM_005622	ACSM3
ASHGV40026829	0.002898413	0.061986011	2.8340033	down	protein_coding	NM_015910	WDPCP
ASHGV40045002	0.002866894	0.061986011	26.8311216	down	protein_coding	NM_181644	MFSD4
ASHGV40045936	0.000890126	0.049204994	3.9096032	down	protein_coding	NM_152410	PACRG
ASHGV40007551	0.006355115	0.082201623	5.5788179	down	protein_coding	NM_025208	PDGFD
ASHGV40021952	0.003187201	0.063583557	6.0278704	down	protein_coding	NM_002924	RGS7
ASHGV40011759	0.016892601	0.12782262	2.2760846	down	protein_coding	NM_152588	TMTC2
ASHGV40015149	0.03025123	0.167607385	2.0392405	down	protein_coding	NM_014215	INSRR
ASHGV40024125	0.003819351	0.067888222	3.3666219	down	protein_coding	NM_001076675	ZNF626
ASHGV40042529	0.017468367	0.12972491	5.3001863	down	protein_coding	NM_001040129	SPINK13
ASHGV40050667	0.023509219	0.149834056	3.4363922	down	protein_coding	NM_006269	RP1
ASHGV40036595	0.002174512	0.056932681	2.0012611	down	protein_coding	NM_001104647	SLC25A36
ASHGV40027740	0.038962125	0.190820817	2.166667	down	protein_coding	NM_001193308	SYTL1
ASHGV40036898	0.005373111	0.076791441	6.2255589	down	protein_coding	NM_130770	HTR3C
ASHGV40030278	0.003943685	0.068946524	2.2055123	down	protein_coding	NM_152879	DGKD
ASHGV40028638	0.023555484	0.149839682	2.1462664	down	protein_coding	NM_012326	MAPRE3
ASHGV40014815	0.009758491	0.098981412	2.5481963	down	protein_coding	NM_005132	REC8
ASHGV40027727	0.00303647	0.062716466	4.0604182	down	protein_coding	NM_152529	GPR155
ASHGV40047930	0.008819078	0.094452974	2.9896159	down	protein_coding	NM_001159522	ZNF727
ASHGV40016318	0.004092556	0.069304474	13.3203667	down	protein_coding	NM_178232	HAPLN3
ASHGV40048473	0.00235503	0.059122906	13.9615082	down	protein_coding	NM_145268	SSMEM1
ASHGV40046196	0.029683298	0.166310408	2.768102	down	protein_coding	NM_001101426	ISPD
ASHGV40023982	0.005058137	0.07484092	3.0144358	down	protein_coding	NM_001277378	C19orf67
ASHGV40053504	0.001982973	0.055772948	4.2453831	down	protein_coding	NM_018201	TBC1D13
ASHGV40046800	0.000165715	0.030140601	2.6236442	down	protein_coding	NM_002612	PDK4
ASHGV40050639	0.04583947	0.207378474	2.0116369	down	protein_coding	NM_152751	BEND7
ASHGV40039559	2.23071E-05	0.025539914	6.2505249	down	protein_coding	NM_000826	GRIA2
ASHGV40031307	0.000345905	0.038034032	4.0677414	down	protein_coding	NM_130811	SNAP25
ASHGV40009594	0.024681032	0.152291693	2.0018874	down	protein_coding	NM_018423	STYK1
ASHGV40015635	0.008381464	0.092027211	3.5732013	down	protein_coding	NM_015307	FAM189A1
ASHGV40014730	0.034324129	0.178694547	2.0408492	down	protein_coding	NM_002937	RNASE4
ASHGV40015333	0.004877445	0.073630419	9.0759438	down	protein_coding	NM_001275	CHGA
ASHGV40005949	0.047082503	0.209580703	9.4474475	down	protein_coding	NM_004190	LIPF
ASHGV40033293	0.012618448	0.110671657	2.0693152	down	protein_coding	ENST0000040536	22NC03-75H12
ASHGV40019999	0.006175539	0.081388289	4.1657556	down	protein_coding	NM_000691	ALDH3A1
ASHGV40001260	0.004557363	0.072041872	2.1726394	down	protein_coding	ENST0000046053	FAM115A
ASHGV40007717	0.023845652	0.150424307	6.8447012	down	protein_coding	NM_006389	HYOU1
ASHGV40052677	0.014790363	0.119946404	2.660966	down	protein_coding	NM_003026	SH3GL2
ASHGV40043524	0.00999011	0.099667779	3.075763	down	protein_coding	NM_018135	MRPS18A
ASHGV40007033	0.033892137	0.17767387	3.6674377	down	protein_coding	NM_001062	TCN1
ASHGV40012635	0.022107907	0.146790385	2.7744977	down	protein_coding	NM_001042517	DIAPH3
ASHGV40013179	0.018666996	0.134660997	2.4029217	down	protein_coding	NM_023037	FRY
ASHGV40044816	0.007872639	0.089971932	2.2278721	down	protein_coding	NM_206809	MOG
ASHGV40003075	0.00801791	0.090230383	2.3875631	down	protein_coding	NM_001010978	LDLRAD1
ASHGV40024326	0.034495765	0.179233539	2.1725797	down	protein_coding	NM_152484	ZNF569
ASHGV40039129	0.001703396	0.055102814	3.1001294	down	protein_coding	NM_001025616	ARHGAP24

ASHGV40028649	0.011522635	0.106699757	9.0904581	down	protein_coding	NM_004341	CAD
ASHGV40006447	0.024852475	0.152493483	3.1354604	down	protein_coding	NM_000773	CYP2E1
ASHGV40037314	0.028105592	0.161785771	3.0634422	down	protein_coding	NM_013261	PPARGC1A
ASHGV40019167	0.024982186	0.152784775	2.4193505	down	protein_coding	NM_014685	HERPUD1
ASHGV40020991	0.024088181	0.150987108	2.3838313	down	protein_coding	ENST0000042589	C17orf70
ASHGV40008596	0.014841716	0.120195153	3.7048102	down	protein_coding	NM_033310	KCNK4
ASHGV40009896	0.001925334	0.055772948	34.3221204	down	protein_coding	NM_001844	COL2A1
ASHGV40017705	1.5883E-06	0.008015752	5.7426218	down	protein_coding	NM_003361	UMOD
ASHGV40032293	0.002676271	0.061839224	12.6362806	down	protein_coding	NM_152489	UBE2U
ASHGV40043901	0.003994272	0.068983335	3.5114859	down	protein_coding	NM_007073	BVES
ASHGV40017127	0.005657292	0.078517713	2.1153643	down	protein_coding	NM_206839	MORF4L1
ASHGV40033285	0.003696429	0.067298943	2.0450083	down	protein_coding	NM_014246	CELSR1
ASHGV40053510	0.002655575	0.061839224	23.6139144	down	protein_coding	NM_032809	FAM73B
ASHGV40025724	0.002356643	0.059122906	2.7252178	down	protein_coding	NM_001098821	TMEM91
ASHGV40029510	0.010076128	0.100163837	2.7796349	down	protein_coding	NM_001079530	CFC1B
ASHGV40049437	0.000715302	0.046533174	2.1381894	down	protein_coding	NM_024504	PRDM14
ASHGV40051832	0.000276319	0.034634372	4.8980002	down	protein_coding	NM_206948	TRPM3
ASHGV40050930	0.001903533	0.055772948	17.2459536	down	protein_coding	NM_001282356	SLC26A7
ASHGV40008536	0.010555183	0.102668445	2.4229165	down	protein_coding	NM_002407	SCGB2A1
ASHGV40005745	0.041363221	0.197429269	2.0532091	down	protein_coding	NM_001852	COL9A2
ASHGV40002225	0.007912954	0.089971932	3.1276905	down	protein_coding	NM_005953	MT2A
ASHGV40047784	0.016074132	0.125064061	2.8362878	down	protein_coding	NM_031449	ZMIZ2
ASHGV40055090	0.001745447	0.055333849	31.6725586	down	protein_coding	NM_001042506	PABPC1L2B
ASHGV40008189	0.003103588	0.062996703	3.5245228	down	protein_coding	NM_213599	ANO5
ASHGV40009133	0.01731711	0.129328049	2.4020554	down	protein_coding	NM_024686	TTLL7
ASHGV40033206	0.001936349	0.055772948	2.4823222	down	protein_coding	NM_002676	PMM1
ASHGV40054190	0.002657174	0.061839224	3.8539388	down	protein_coding	NM_014496	RPS6KA6
ASHGV40052254	0.004061382	0.069284044	3.1841541	down	protein_coding	NM_001735	C5
ASHGV40007090	0.012297852	0.109697039	6.4542382	down	protein_coding	ENST0000043100	C11orf48
ASHGV40053799	6.65183E-05	0.028718686	5.3213301	down	protein_coding	ENST0000038034	TMEM27
ASHGV40053304	0.0048646	0.073623294	2.5139856	down	protein_coding	NM_133464	ZNF483
ASHGV40035217	0.000483283	0.041928091	2.0527806	down	protein_coding	NM_005241	MECOM
ASHGV40002522	0.003351082	0.064680656	2.4583504	down	protein_coding	ENST0000058751	CTB-102L5.4
ASHGV40023304	0.012746534	0.111291221	3.8228343	down	protein_coding	NM_001281739	FHOD3
ASHGV40032588	0.013839093	0.116064657	2.4860965	down	protein_coding	NM_058187	EVA1C
ASHGV40029727	0.027606418	0.16063555	2.3944824	down	protein_coding	NM_021007	SCN2A
ASHGV40046729	0.007450576	0.087598278	2.0941882	down	protein_coding	NM_014510	PCLO
ASHGV40043480	0.002026287	0.055772948	30.532674	down	protein_coding	NM_002630	PGC
ASHGV40024368	0.011439268	0.10626269	2.0472266	down	protein_coding	NM_178820	FBXO27
ASHGV40008122	0.03340464	0.176391066	2.6274709	down	protein_coding	NM_001080521	RASSF10
ASHGV40017190	0.023113035	0.148817902	2.2473403	down	protein_coding	NM_003027	SH3GL3
ASHGV40008513	0.006278633	0.081636279	294.4749533	down	protein_coding	NM_014224	PGA5
ASHGV40006990	0.003322641	0.064680656	2.6745807	down	protein_coding	NM_024603	BEND5
ASHGV40049284	0.005524414	0.077743087	3.2770357	down	protein_coding	NM_014682	ST18
ASHGV40011891	0.036562739	0.183984971	2.4355283	down	protein_coding	NM_001253849	VTCN1
ASHGV40040866	0.026581629	0.157860108	3.1068591	down	protein_coding	NM_001496	GFRA3
ASHGV40051077	0.017263972	0.129292086	3.570984	down	protein_coding	NM_014241	HACD1
ASHGV40054143	0.042624716	0.200285477	2.0837909	down	protein_coding	NM_021963	NAP1L2
ASHGV40036573	0.011887922	0.108471894	4.5221235	down	protein_coding	NM_006623	PHGDH
ASHGV40036754	0.022536261	0.147611664	2.0246804	down	protein_coding	NM_015938	NMD3
ASHGV40040429	0.004151494	0.069802755	2.8175334	down	protein_coding	NM_013391	DMGDH
ASHGV40003205	0.005068732	0.07484092	2.1066413	down	protein_coding	NM_001278267	NBPF20
ASHGV40010227	0.022946777	0.148477601	2.1039729	down	protein_coding	NM_001874	CPM
ASHGV40056430	0.000164224	0.030140601	4.1933199	down	protein_coding	NM_001270945	LOC643355
ASHGV40027760	0.000457006	0.041719107	2.246565	down	protein_coding	NM_152517	TTC30B
ASHGV40025420	0.00423858	0.070518974	2.0161436	down	protein_coding	NM_001001415	ZNF429
ASHGV40001382	0.012612315	0.110671657	4.0761732	down	protein_coding	NM_002980	SCTR
ASHGV40026407	0.000262097	0.033851258	2.1790713	down	protein_coding	NM_001099218	RAD51AP2
ASHGV40021756	0.016323112	0.126020119	14.8629114	down	protein_coding	NM_173478	CNTD1
ASHGV40030970	0.009570563	0.0980628	3.4884123	down	protein_coding	NM_004975	KCNB1
ASHGV40043629	0.012081908	0.108868292	2.9958105	down	protein_coding	NM_019036	HMGCLL1

ASHGV40044815	0.044661268	0.20513496	3.0982571	down	protein_coding	NM_007160	OR2H2
ASHGV40027653	0.002024543	0.055772948	3.5475804	down	protein_coding	NM_014900	COBLL1
ASHGV40037794	0.04834327	0.212427834	3.9051636	down	protein_coding	NM_000668	ADH1B
ASHGV40027066	0.039311931	0.191947024	2.3913031	down	protein_coding	NM_001010980	NCMAP
ASHGV40006843	0.000659224	0.046153893	13.3345219	down	protein_coding	NM_004171	SLC1A2
ASHGV40055112	0.003257016	0.064458674	3.0574697	down	protein_coding	uc004ecw.2	ATP7A
ASHGV40054902	0.009869993	0.099354989	3.4903581	down	protein_coding	NM_001129898	KRBOX4
ASHGV40015827	0.028356371	0.162598688	2.6221009	down	protein_coding	ENST0000032432	MYEF2
ASHGV40035760	0.004918206	0.073925721	55.3332025	down	protein_coding	NM_138410	CMTM7
ASHGV40041977	0.022437091	0.147541072	11.0196097	down	protein_coding	NM_001825	CKMT2
ASHGV40013977	0.002081427	0.055772948	2.0395981	down	protein_coding	NM_003944	SELENBP1
ASHGV40019013	0.027291901	0.15979234	3.1576184	down	protein_coding	NM_133443	GPT2
ASHGV40036538	0.012314291	0.109713394	9.1146061	down	protein_coding	NM_001099	ACPP
ASHGV40043008	0.004830099	0.073552499	2.176935	down	protein_coding	NM_001145020	C6orf52
ASHGV40053507	0.00674588	0.083835464	3.5277289	down	protein_coding	NM_001100876	PHYHD1
ASHGV40006008	0.000720151	0.046533174	4.8441397	down	protein_coding	NM_005097	LGI1
ASHGV40038868	0.005005917	0.074655993	16.1192591	down	protein_coding	NM_025087	CWH43
ASHGV40011164	0.023254923	0.149119145	2.4920293	down	protein_coding	NM_001001660	LYRM5
ASHGV40007226	0.003445198	0.064941046	3.3438772	down	protein_coding	NM_206997	GPR152
ASHGV40003168	0.0492454	0.214018641	2.0756168	down	protein_coding	NM_016282	AK3
ASHGV40033227	0.015223155	0.121970651	2.0496745	down	protein_coding	NM_032311	POLDIP3
ASHGV40019938	0.01181949	0.108237724	2.3485407	down	protein_coding	NM_020653	ZNF287
ASHGV40048011	0.007115965	0.08550928	3.4656883	down	protein_coding	uc011kfh.1	NCF1
ASHGV40011584	0.003524578	0.065507718	7.1342298	down	protein_coding	NM_004731	SLC16A7
ASHGV40008050	0.000535091	0.043394606	4.8637695	down	protein_coding	NM_207186	OR10A4
ASHGV40020172	0.00903349	0.095807742	2.0906558	down	protein_coding	NM_006584	CCT6B
ASHGV40036419	0.0114522	0.10632618	2.8906118	down	protein_coding	ENST0000027339	MAATS1
ASHGV40026020	0.001922849	0.055772948	2.8945799	down	protein_coding	NM_001012728	DPRX
ASHGV40030187	0.023674405	0.149889765	2.1112795	down	protein_coding	NM_004457	ACSL3
ASHGV40007116	0.029134416	0.164610392	3.2059653	down	protein_coding	NM_173587	RCOR2
ASHGV40041583	0.049997424	0.215711352	3.7936882	down	protein_coding	ENST0000032695	AC026703.1
ASHGV40056694	0.004468759	0.071681034	3.4665949	down	protein_coding	NM_001099771	POTEF
ASHGV40037618	0.000274635	0.034634372	12.9785623	down	protein_coding	NM_000583	GC
ASHGV40037572	0.003076523	0.062812953	4.4540756	down	protein_coding	NM_004439	EPHA5
ASHGV40043603	0.01153403	0.106699757	5.2311493	down	protein_coding	NM_145740	GSTA1
ASHGV40007211	0.004626906	0.072674639	2.6447691	down	protein_coding	NM_003793	CTSF
ASHGV40040165	0.012549771	0.11054147	3.4105545	down	protein_coding	NM_000065	C6
ASHGV40008486	0.023708221	0.149991486	2.0691383	down	protein_coding	NM_024848	MORN1
ASHGV40022184	0.000295685	0.035312118	3.8803287	down	protein_coding	NM_032646	TTYH2
ASHGV40020425	0.019599281	0.138353468	3.6723446	down	protein_coding	NM_001264573	KIF18B
ASHGV40008433	0.002549153	0.06066831	55.9153258	down	protein_coding	NM_001005204	OR8U1
ASHGV40000124	0.004501556	0.071864614	3.5873745	down	protein_coding	NM_006615	CAPN9
ASHGV40016257	0.004142092	0.06971188	9.2685318	down	protein_coding	NM_199330	HOMER2
ASHGV40012401	3.05312E-05	0.026561918	2.6687116	down	protein_coding	NM_007106	UBL3
ASHGV40053317	0.019206752	0.136825495	3.0307958	down	protein_coding	NM_133465	KIAA1958
ASHGV40055988	0.031388673	0.170604662	2.0306019	down	protein_coding	NM_004316	ASCL1
ASHGV40026079	0.002487355	0.060068593	7.816769	down	protein_coding	NM_033113	ZNF628
ASHGV40015147	0.00397008	0.068946524	3.2684325	down	protein_coding	NM_022137	SMOC1
ASHGV40047428	0.00524347	0.076092721	3.2627338	down	protein_coding	NM_001505	GPER1
ASHGV40025172	0.032791413	0.174880478	2.0229876	down	protein_coding	NM_020428	SLC44A2
ASHGV40037335	0.000671004	0.046153893	14.5145177	down	protein_coding	NM_000730	CCKAR
ASHGV40017411	0.00671103	0.083754019	2.8209493	down	protein_coding	NM_183337	RGS11
ASHGV40033714	0.035242116	0.18064501	2.9785449	down	protein_coding	NM_033386	MICALL1
ASHGV40027862	0.033638177	0.177029899	2.1686199	down	protein_coding	NM_016192	TMEFF2
ASHGV40023724	0.007127957	0.085567393	2.1390043	down	protein_coding	ENST0000058784	C19orf77
ASHGV40041211	0.010496677	0.102456293	2.2215772	down	protein_coding	NM_020444	KIAA1191
ASHGV40019927	0.004357619	0.071084094	151.7943613	down	protein_coding	ENST0000045558	RP11-385D13.1
ASHGV40001274	0.039438861	0.192243775	2.2476639	down	protein_coding	uc001jtv.4	TTC18
ASHGV40025873	0.007095709	0.085506036	12.1908993	down	protein_coding	NM_004070	CLCNKA
ASHGV40030288	0.015051861	0.121112485	3.8455706	down	protein_coding	NM_001287395	MROH2A
ASHGV40038212	0.01440612	0.118252561	2.0807742	down	protein_coding	NM_000909	NPY1R

ASHGV40006707	0.001764979	0.055403878	5.7788566	down	protein_coding	NM_000352	ABCC8
ASHGV40016593	0.000252816	0.033394702	28.6314065	down	protein_coding	NM_000261	MYOC
ASHGV40033467	0.014235817	0.117526372	2.5064837	down	protein_coding	NM_001010971	SAMD13
ASHGV40054402	0.009956788	0.099667779	4.2078739	down	protein_coding	NM_178470	DCAF12L1
ASHGV40008819	0.019851827	0.138920246	3.0401158	down	protein_coding	NM_000802	FOLR1
ASHGV40024615	0.015108449	0.121452705	2.6505624	down	protein_coding	NM_001190	BCAT2
ASHGV40034420	0.00828511	0.091649926	2.5702311	down	protein_coding	NM_198563	TMEM110
ASHGV40043399	0.003442775	0.064941046	6.3108447	down	protein_coding	NM_001832	CLPS
ASHGV40023428	0.031817974	0.172291363	2.1519296	down	protein_coding	NM_001144967	NEDD4L
ASHGV40021773	5.55282E-05	0.028718686	6.3964332	down	protein_coding	NM_019891	ERO1B
ASHGV40028616	0.002851712	0.061986011	4.2163852	down	protein_coding	NM_014971	EFR3B
ASHGV40025558	0.001895834	0.055772948	11.8814575	down	protein_coding	NM_002151	HPN
ASHGV40038813	0.005807731	0.079484774	2.4040605	down	protein_coding	ENST0000038178	NSUN7
ASHGV40029729	0.005048965	0.07484092	2.0635085	down	protein_coding	NM_024969	CSRNP3
ASHGV40042088	0.00440234	0.071459267	2.3551281	down	protein_coding	NM_001012761	RGMB
ASHGV40038461	0.009365176	0.097468969	4.4459745	down	protein_coding	NM_004091	E2F2
ASHGV40013014	0.001024469	0.049811075	425.2288969	down	protein_coding	NM_000705	ATP4B
ASHGV40047665	0.009380309	0.097468969	3.2649713	down	protein_coding	NM_001080529	WIPF3
ASHGV40043788	0.001957415	0.055772948	4.882284	down	protein_coding	NM_000735	CGA
ASHGV40020766	0.048289118	0.212381007	2.5394328	down	protein_coding	NM_007168	ABCA8
ASHGV40010466	0.022116216	0.146790385	2.1456675	down	protein_coding	NM_152317	DEPDC4
ASHGV40022860	0.014907517	0.120480163	3.5208887	down	protein_coding	NM_173557	RNF152
ASHGV40041220	0.031968763	0.172465145	2.8244098	down	protein_coding	NM_002115	HK3
ASHGV40049457	0.004291341	0.070721944	4.7489876	down	protein_coding	NM_153225	SBSPON
ASHGV40000050	0.006410287	0.082486913	2.9027803	down	protein_coding	ENST0000031290	CCDC147
ASHGV40010416	0.021270721	0.144232877	3.3033408	down	protein_coding	NM_001918	DBT
ASHGV40052510	0.012048133	0.108852935	2.3448034	down	protein_coding	NM_198946	LCN6
ASHGV40042401	0.00225335	0.058034571	2.8689432	down	protein_coding	NM_032412	CYSTM1
ASHGV40034275	0.000478077	0.041888197	3.9656616	down	protein_coding	NM_024512	LRRC2
ASHGV40060884	0.000104188	0.028718686	7.9593029	down	protein_coding	uc010zse.2	C20orf26
ASHGV40005788	0.004040103	0.069284044	3.6591506	down	protein_coding	NM_019058	DDIT4
ASHGV40034724	0.003451467	0.064944871	29.3858157	down	protein_coding	NM_005459	GUCA1C
ASHGV40032953	0.000130541	0.029433159	4.5883835	down	protein_coding	NM_001001663	TMEM211
ASHGV40021574	0.001141316	0.050016538	3.5868827	down	protein_coding	NM_173847	SPACA3
ASHGV40009160	0.005902861	0.080032877	4.0552795	down	protein_coding	NM_006006	ZBTB16
ASHGV40036529	0.038171571	0.188597196	2.1299272	down	protein_coding	NM_153264	COL6A5
ASHGV40033993	0.010501807	0.102456293	20.419112	down	protein_coding	NM_016362	GHRL
ASHGV40026918	0.000962079	0.049736021	6.7495731	down	protein_coding	NM_020459	PAIP2B
ASHGV40033450	0.004942931	0.074157667	14.703766	down	protein_coding	NM_005446	P2RX6
ASHGV40054135	0.018091806	0.132541486	3.2145803	down	protein_coding	NM_002637	PHKA1
ASHGV40045807	0.007030193	0.085147057	8.9046526	down	protein_coding	NM_015093	TAB2
ASHGV40033241	0.00138113	0.052512256	3.4775326	down	protein_coding	NM_022785	EFCAB6
ASHGV40049531	0.000843825	0.048843614	3.5630808	down	protein_coding	NM_002677	PMP2
ASHGV40043605	0.015979671	0.124774536	5.3681949	down	protein_coding	NM_000847	GSTA3
ASHGV40001345	0.004354096	0.071084094	2.0306682	down	protein_coding	NM_005090	MJD7-PLA2G4I
ASHGV40027146	0.046866774	0.209208674	2.7908592	down	protein_coding	NM_198461	LONRF2
ASHGV40045597	0.002683669	0.061839224	3.6854866	down	protein_coding	NM_001029858	SLC35F1
ASHGV40053902	0.026493697	0.157559292	2.0018764	down	protein_coding	NM_006307	SRPX
ASHGV40057061	0.003075812	0.062812953	2.9884524	down	protein_coding	NM_006174	NPY5R
ASHGV40018559	0.001864813	0.055772948	3.7592726	down	protein_coding	NM_016243	CYB5R1
ASHGV40022613	0.041759531	0.198614072	5.1029662	down	protein_coding	NM_031422	CHST9
ASHGV40019958	0.046959843	0.209283925	2.4695612	down	protein_coding	NM_016084	RASD1
ASHGV40013969	0.002685968	0.061839224	2.2253535	down	protein_coding	NM_016586	MBIP
ASHGV40051042	0.015780351	0.123828175	3.2746388	down	protein_coding	NM_024812	BAALC
ASHGV40011123	0.000808363	0.048770305	11.2717453	down	protein_coding	NM_033328	CAPZA3
ASHGV40033531	0.015343474	0.122207594	2.8165377	down	protein_coding	NM_001039948	SGSM1
ASHGV40034517	0.011184661	0.104993761	2.1743488	down	protein_coding	NM_004742	MAGI1
ASHGV40020144	0.023083126	0.148735174	7.2071441	down	protein_coding	ENST0000039883	AC090616.2
ASHGV40038491	0.003508924	0.065507718	2.3598479	down	protein_coding	NM_182982	GRK4
ASHGV40035734	0.032193534	0.173142031	2.6624838	down	protein_coding	NM_181643	PIFO
ASHGV40030142	0.000157334	0.030140601	6.9625816	down	protein_coding	NM_025216	WNT10A

ASHGV40015556	0.00189016	0.055772948	10.5734253	down	protein_coding	NM_033438	SLAMF9
ASHGV40027166	0.049836595	0.215352653	2.7526616	down	protein_coding	NM_153836	CREG2
ASHGV40013208	0.011664582	0.107326462	2.5678328	down	protein_coding	NM_015678	NBEA
ASHGV40027037	0.022720701	0.147893728	2.1068018	down	protein_coding	NM_022912	REEP1
ASHGV40046903	0.001226506	0.050556396	7.9570049	down	protein_coding	NM_145032	FBXL13
ASHGV40010512	0.002021176	0.055772948	2.6599852	down	protein_coding	NM_001034173	ALDH1L2
ASHGV40056653	0.011344863	0.105610805	3.7535228	down	protein_coding	NM_031288	INO80B
ASHGV40022612	0.002938239	0.061986011	61.5245617	down	protein_coding	NM_001650	AQP4
ASHGV40027776	0.002290508	0.058473339	10.5488846	down	protein_coding	NM_152520	ZNF385B
ASHGV40032335	0.037091208	0.185307252	2.9482719	down	protein_coding	NM_000071	CBS
ASHGV40054095	0.00821783	0.091307649	73.4823658	down	protein_coding	NM_021783	EDA2R
ASHGV40039254	0.001512287	0.053485251	2.811362	down	protein_coding	uc021pao.2	AX746485
ASHGV40047903	0.006688396	0.083657725	13.8285806	down	protein_coding	NM_001146333	SUMF2
ASHGV40052182	0.001075424	0.049811075	5.895819	down	protein_coding	ENST0000031873	C9orf84
ASHGV40011398	0.034815381	0.179597926	2.0048375	down	protein_coding	NM_004302	ACVR1B
ASHGV40031579	0.00370682	0.067298943	3.8185317	down	protein_coding	NM_004139	LBP
ASHGV40039046	0.000720576	0.046533174	8.5324601	down	protein_coding	NM_001133	AFM
ASHGV40055501	0.029803998	0.166580805	2.2951209	down	protein_coding	NM_001164415	HSFX2
ASHGV40054673	0.00756045	0.087925399	2.2855648	down	protein_coding	NM_005647	TBL1X
ASHGV40033546	0.021200222	0.144037554	4.4714783	down	protein_coding	NM_032608	MYO18B
ASHGV40015996	0.014412681	0.118252561	2.1278061	down	protein_coding	NM_194272	RBPMS2
ASHGV40018045	0.006836518	0.084300935	5.5196428	down	protein_coding	NM_001301267	MT1G
ASHGV40050897	0.032144392	0.172965747	3.2696555	down	protein_coding	NM_000067	CA2
ASHGV40028214	0.012373984	0.109722298	8.6635758	down	protein_coding	NM_002242	KCNJ13
ASHGV40038250	0.011269983	0.105338938	2.3889365	down	protein_coding	NM_012224	NEK1
ASHGV40024434	0.005763482	0.079252423	2.9996537	down	protein_coding	NM_152296	ATP1A3
ASHGV40025598	0.048187832	0.212226079	2.083462	down	protein_coding	NM_005166	APLP1
ASHGV40017265	0.002389183	0.059482588	6.7806186	down	protein_coding	NM_022769	CRTC3
ASHGV40021794	0.002985599	0.06249329	3.3496822	down	protein_coding	NM_080863	ASB16
ASHGV40009590	0.002044002	0.055772948	3.2070968	down	protein_coding	NM_002260	KLRC2
ASHGV40030081	6.97115E-05	0.028718686	3.0048286	down	protein_coding	NM_024532	SPAG16
ASHGV40017832	0.001400586	0.052512256	4.1301872	down	protein_coding	NM_001145524	YPEL3
ASHGV40031162	0.02672684	0.158398769	5.000263	down	protein_coding	NM_001958	EEF1A2
ASHGV40009084	0.001113791	0.049811075	31.2405795	down	protein_coding	NM_001077244	GRIA4
ASHGV40020463	0.000449134	0.041719107	4.2201477	down	protein_coding	NM_203400	RPRML
ASHGV40017706	0.000981404	0.049743466	22.793116	down	protein_coding	NM_174924	PDILT
ASHGV40031136	0.045639933	0.207080587	3.1651645	down	protein_coding	NM_080473	GATA5
ASHGV40032921	0.021872574	0.146006966	2.0413025	down	protein_coding	NM_020070	IGLL1
ASHGV40013072	0.024348221	0.151674021	2.4163502	down	protein_coding	NM_002010	FGF9
ASHGV40041915	6.56305E-05	0.028718686	3.5376896	down	protein_coding	NM_001177693	ARHGEF28
ASHGV40023753	0.000140142	0.029433159	5.2891112	down	protein_coding	NM_001080400	PLIN4
ASHGV40020364	0.00034873	0.038034032	2.2949349	down	protein_coding	ENST0000059102	PLEKHH3
ASHGV40041720	0.002734236	0.061986011	3.5942943	down	protein_coding	NM_002202	ISL1
ASHGV40034519	0.006378476	0.082378819	3.472183	down	protein_coding	NM_015541	LRIG1
ASHGV40027759	0.00315019	0.063464592	2.1372283	down	protein_coding	NM_006164	NFE2L2
ASHGV40024582	0.003483476	0.065379863	15.9809504	down	protein_coding	NM_015063	SLC8A2
ASHGV40044257	0.005856106	0.079836199	5.901346	down	protein_coding	NM_017909	RMND1
ASHGV40041160	0.007751838	0.089215209	3.8811968	down	protein_coding	NM_012300	FBXW11
ASHGV40028192	0.036505624	0.183870023	4.3551498	down	protein_coding	NM_178865	SERINC2
ASHGV40025003	0.008337999	0.09183913	2.5820932	down	protein_coding	NM_173480	ZNF57
ASHGV40000139	5.02487E-05	0.028718686	3.6666048	down	protein_coding	NM_001291332	MROH7
ASHGV40031279	0.017054161	0.12858334	2.2259595	down	protein_coding	NM_001819	CHGB
ASHGV40021228	0.001503333	0.05332898	4.250584	down	protein_coding	NM_001256615	LOC149373
ASHGV40023175	0.004542028	0.071864614	2.3175776	down	protein_coding	NM_005913	MC5R
ASHGV40040119	0.00933629	0.097468969	13.2498484	down	protein_coding	NM_145000	RANBP3L
ASHGV40054911	0.003701637	0.067298943	4.8766232	down	protein_coding	NM_004683	RGN
ASHGV40035173	0.043936121	0.203850507	2.0139734	down	protein_coding	NM_003781	B3GALNT1
ASHGV40041354	0.001622881	0.054621802	3.9568691	down	protein_coding	NM_052862	RCSD1
ASHGV40024282	0.001011666	0.049811075	351.7559654	down	protein_coding	NM_000704	ATP4A
ASHGV40050264	0.027972071	0.161445259	4.5803218	down	protein_coding	ENST0000028448	C8orf12
ASHGV40024429	0.023860217	0.150449728	2.6582936	down	protein_coding	NM_001288583	SMIM1

ASHGV40033006	0.049064273	0.213699077	2.0852904	down	protein_coding	NM_015370	C22orf31
ASHGV40011488	0.003995938	0.068983335	4.1039077	down	protein_coding	NM_001005243	OR9K2
ASHGV40017482	0.012303045	0.109697039	3.5811714	down	protein_coding	NM_178167	ZNF598
ASHGV40039329	0.017609679	0.130323586	2.0812061	down	protein_coding	NM_019050	USP53
ASHGV40030338	0.037620531	0.186916924	2.042202	down	protein_coding	NM_024101	MLPH
ASHGV40012604	0.003957352	0.068946524	4.3440429	down	protein_coding	NM_002590	PCDH8
ASHGV40045027	0.003166661	0.063464592	12.8939091	down	protein_coding	NM_006789	APOBEC2
ASHGV40034429	0.034677426	0.179356685	2.0600041	down	protein_coding	NM_018397	CHDH
ASHGV40022594	0.000225266	0.032731164	3.7311511	down	protein_coding	NM_173505	ANKRD29
ASHGV40016079	0.022448858	0.147541072	2.4369351	down	protein_coding	NM_018357	LARP6
ASHGV40037497	0.019862823	0.138920246	2.1804535	down	protein_coding	NM_001126328	LNX1
ASHGV40022749	0.03002202	0.16676137	3.6883893	down	protein_coding	NM_013305	ST8SIA5
ASHGV40025369	0.00408257	0.069284044	14.1831351	down	protein_coding	NM_012109	TMEM59L
ASHGV40054479	0.007286549	0.086427388	3.9972979	down	protein_coding	NM_004114	FGF13
ASHGV40034915	0.00115064	0.050016538	4.2354534	down	protein_coding	NM_130808	CPNE4
ASHGV40009137	0.016092275	0.125093583	2.0229493	down	protein_coding	ENST0000059505	AP002884.2
ASHGV40019159	0.008043363	0.090305271	2.9713571	down	protein_coding	uc010ccj.1	MT1IP
ASHGV40020397	0.000865315	0.048985823	4.9620735	down	protein_coding	NM_001278374	MPP2
ASHGV40050272	0.005787273	0.079404247	2.5762734	down	protein_coding	ENST0000052504	C8orf49
ASHGV40032405	0.004701444	0.073060937	3.4546175	down	protein_coding	ENST0000060106	PRED60
ASHGV40057865	0.007109389	0.08550928	3.5566769	down	protein_coding	NM_014033	METTL7A
ASHGV40003291	0.01668675	0.127466323	2.9394046	down	protein_coding	NM_018476	BEX1
ASHGV40051079	0.0429165	0.201116194	3.0589938	down	protein_coding	NM_177531	PKHD1L1
ASHGV40018194	0.000449496	0.041719107	17.3750465	down	protein_coding	NM_144676	TMED6
ASHGV40000061	0.000323227	0.036835164	4.3683815	down	protein_coding	ENST0000032733	ERO1LB
ASHGV40018018	0.009615193	0.098156036	2.3215721	down	protein_coding	NM_001308963	CRNDE
ASHGV40027495	0.000547994	0.043740162	7.634266	down	protein_coding	NM_018557	LRP1B
ASHGV40037593	0.003441242	0.064941046	7.2127313	down	protein_coding	NM_001076	UGT2B15
ASHGV40034980	0.001494865	0.053309279	2.8515152	down	protein_coding	NM_001134659	PRR23A
ASHGV40026121	0.002456016	0.059725368	3.4211638	down	protein_coding	NM_021216	ZNF71
ASHGV40027425	0.001381713	0.052512256	10.871926	down	protein_coding	NM_032545	CFC1
ASHGV40038155	0.009801111	0.099240515	19.4475517	down	protein_coding	NM_000508	FGA
ASHGV40006785	0.002461149	0.059755395	2.7338314	down	protein_coding	NM_001709	BDNF
ASHGV40055963	0.003403349	0.064805109	4.8514593	down	protein_coding	NM_007264	GPR182
ASHGV40036913	0.004801657	0.073532246	4.2949233	down	protein_coding	NM_001304472	CHRD
ASHGV40017433	0.000210046	0.031004988	4.2718724	down	protein_coding	NM_022773	LMF1
ASHGV40014317	0.001188194	0.050120601	9.165508	down	protein_coding	ENST0000055351	RP11-293M10.1
ASHGV40025985	0.001770008	0.055403878	2.9616166	down	protein_coding	NM_001310155	FAM231B
ASHGV40035823	0.010562188	0.102668445	3.1566049	down	protein_coding	NM_015460	MYRIP
ASHGV40034349	0.002896022	0.061986011	2.5986953	down	protein_coding	NM_000481	AMT
ASHGV40038234	0.010341466	0.101599149	2.1492385	down	protein_coding	NM_016950	SPOCK3
ASHGV40017791	0.006976139	0.085038143	28.4507682	down	protein_coding	ENST0000045231	NPIPB7
ASHGV40054328	0.00651315	0.082892908	3.4367886	down	protein_coding	NM_000399	EGR2
ASHGV40028127	0.043002083	0.201446621	2.1705016	down	protein_coding	NM_003469	SCG2
ASHGV40045072	0.000136817	0.029433159	35.655201	down	protein_coding	NM_000707	AVPR1B
ASHGV40013980	0.012970728	0.112332125	2.3504467	down	protein_coding	NM_004496	FOXA1
ASHGV40056819	0.001212278	0.0505256	84.1025507	down	protein_coding	NM_172201	KCNE2
ASHGV40021387	0.033155631	0.175924118	2.7692573	down	protein_coding	NM_030665	RAI1
ASHGV40007228	0.011750586	0.107782941	3.6909084	down	protein_coding	NM_025124	TMEM134
ASHGV40001419	0.01900806	0.136164558	2.7102079	down	protein_coding	NM_032517	LYZL1
ASHGV40055198	0.004124585	0.069649315	3.6852748	down	protein_coding	NM_014467	SRPX2
ASHGV40045602	0.002619762	0.061427953	2.7137126	down	protein_coding	NM_014034	ASF1A
ASHGV40032838	0.039856316	0.193289207	2.229007	down	protein_coding	NM_005984	SLC25A1
ASHGV40009952	0.000554403	0.043740162	6.8436553	down	protein_coding	NM_001031628	SMAGP
ASHGV40049351	0.000506687	0.042546158	3.3267746	down	protein_coding	NM_014729	TOX
ASHGV40006468	0.033381602	0.176322816	2.1661647	down	protein_coding	NM_173573	LMNTD2
ASHGV40006478	0.007560611	0.087925399	2.1774807	down	protein_coding	NM_021008	DEAF1
ASHGV40018428	0.001261608	0.05083886	4.3191091	down	protein_coding	NM_178841	RNF166
ASHGV40055213	0.004621329	0.072674639	2.0831438	down	protein_coding	NM_016608	ARMCX1
ASHGV40006633	0.014220769	0.117526372	2.1618082	down	protein_coding	NM_001289058	STK33
ASHGV40019186	0.004747145	0.073457793	2.8622775	down	protein_coding	uc021pik.2	MIR29C

ASHGV40041766	0.035145671	0.180447561	2.3331281	down	protein_coding	NM_002022	FMO4
ASHGV40057359	0.031389066	0.170604662	2.1430668	down	protein_coding	NM_203397	MBLAC1
ASHGV40042537	0.032500726	0.174096054	2.2479495	down	protein_coding	NM_000024	ADRB2
ASHGV40016843	0.000184521	0.030140601	2.5711455	down	protein_coding	NM_001198784	C15orf65
ASHGV40003316	0.010785909	0.103530481	2.5003219	down	protein_coding	NM_032435	KIAA1804
ASHGV40031102	0.003314118	0.064680656	2.4198625	down	protein_coding	NM_014258	SYCP2
ASHGV40049279	0.034685262	0.179356685	3.1209222	down	protein_coding	NM_144651	PXDNL
ASHGV40037755	0.006820437	0.084221776	2.175326	down	protein_coding	NM_014883	FAM13A
ASHGV40053483	0.020823389	0.142877377	2.0021932	down	protein_coding	:NST0000044349	AL590708.2
ASHGV40035761	0.001535197	0.053801741	4.2522703	down	protein_coding	NM_174896	C1orf162
ASHGV40027161	0.008806056	0.094371472	2.124577	down	protein_coding	NM_001102426	TBC1D8
ASHGV40030466	0.015701192	0.12360564	2.8172071	down	protein_coding	NM_001282533	LZTS3
ASHGV40053310	0.021412546	0.144652908	2.0737952	down	protein_coding	NM_003358	UGCG
ASHGV40054269	0.000883038	0.049190557	5.1471814	down	protein_coding	NM_032621	BEX2
ASHGV40053339	0.033295425	0.176134416	3.4401721	down	protein_coding	NM_000607	ORM1
ASHGV40046438	0.011285972	0.105338938	2.0379001	down	protein_coding	NM_013284	POLM
ASHGV40053855	0.043616391	0.202997913	2.5705965	down	protein_coding	NM_139058	ARX
ASHGV40041933	0.000516863	0.042710987	2.2039602	down	protein_coding	NM_014979	SV2C
ASHGV40032343	0.002522142	0.060406687	5.2976223	down	protein_coding	NM_173354	SIK1
ASHGV40014617	0.009808083	0.099253472	7.7127949	down	protein_coding	NM_001823	CKB
ASHGV40043182	0.024417334	0.151714606	2.6947111	down	protein_coding	NM_030899	ZSCAN31
ASHGV40026818	0.008242668	0.091435455	3.2165056	down	protein_coding	NM_032180	FAM161A
ASHGV40045893	0.027895647	0.161162524	2.528197	down	protein_coding	NM_001242384	SYTL3
ASHGV40050770	0.002070959	0.055772948	2.0945665	down	protein_coding	NM_001029954	CDNF
ASHGV40028900	0.039099312	0.191207612	2.0349179	down	protein_coding	NM_001143959	C2orf74
ASHGV40025399	0.026255492	0.156959315	2.136561	down	protein_coding	NM_007138	ZNF90
ASHGV40048901	0.009375614	0.097468969	3.2309284	down	protein_coding	NM_053279	FAM167A
ASHGV40053225	0.013325025	0.113987801	5.0146845	down	protein_coding	NM_003692	TMEFF1
ASHGV40024613	0.000143076	0.029433159	6.8204903	down	protein_coding	NM_182575	IZUMO1
ASHGV40011912	0.015350462	0.122207594	6.080195	down	protein_coding	NM_017564	STAB2
ASHGV40041233	0.013224049	0.113360136	4.1129854	down	protein_coding	NM_024872	DOK3
ASHGV40005905	0.022413194	0.147541072	2.5194025	down	protein_coding	NM_033100	CDHR1
ASHGV40014048	0.003024896	0.062622161	2.090186	down	protein_coding	NM_007185	CELF3
ASHGV40046566	0.001263846	0.05083886	18.3972772	down	protein_coding	NM_001145712	NUPR1L
ASHGV40019161	0.019304978	0.136829914	3.0497178	down	protein_coding	NM_005952	MT1X
ASHGV40024445	0.019403392	0.137248499	12.0655427	down	protein_coding	NM_198477	CXCL17
ASHGV40007440	0.012199078	0.109304951	2.2819502	down	protein_coding	NM_012193	FZD4
ASHGV40056860	0.002393685	0.059482588	4.5768249	down	protein_coding	uc011ant.3	LOC400927
ASHGV40016642	0.00452259	0.071864614	3.1227223	down	protein_coding	NM_207444	C15orf53
ASHGV40048258	0.008456713	0.09250392	2.0319096	down	protein_coding	NM_001278563	COL26A1
ASHGV40025737	0.02129768	0.144232877	2.0128804	down	protein_coding	NM_001040283	DMRTC2
ASHGV40008093	0.000153917	0.029818933	9.1906441	down	protein_coding	NM_181712	KANK4
ASHGV40051328	0.033108538	0.175924118	2.1551085	down	protein_coding	NM_207371	SKIDA1
ASHGV40012895	8.35824E-05	0.028718686	6.0173055	down	protein_coding	NM_004115	FGF14
ASHGV40012276	0.005491361	0.077465505	5.1616712	down	protein_coding	NM_018663	PXMP2
ASHGV40057604	0.00898092	0.095540769	2.0380601	down	protein_coding	NM_181532	ERAS
ASHGV40029372	0.003416421	0.064819067	5.5348639	down	protein_coding	NM_020868	DPP10
ASHGV40022247	0.029655468	0.166261331	2.1102765	down	protein_coding	NM_182565	UBALD2
ASHGV40008537	3.65615E-05	0.026561918	4.2941976	down	protein_coding	NM_006551	SCGB1D2
ASHGV40037793	0.04677171	0.209075944	2.6864901	down	protein_coding	NM_000667	ADH1A
ASHGV40032667	0.001745372	0.055333849	3.4260253	down	protein_coding	NM_002243	KCNJ15
ASHGV40007535	0.006667914	0.083521372	2.0665392	down	protein_coding	NM_022122	MMP27
ASHGV40046866	0.003709239	0.067298943	8.6718149	down	protein_coding	NM_003227	TFR2
ASHGV40027689	0.020044555	0.139631184	2.8224908	down	protein_coding	:NST0000040978	LINC01124
ASHGV40006829	0.003213562	0.063963086	3.611948	down	protein_coding	NM_145804	ABTB2
ASHGV40046197	0.025339127	0.154326364	8.8782264	down	protein_coding	NM_015464	SOSTDC1
ASHGV40027455	0.009834245	0.099354989	2.1529518	down	protein_coding	NM_207363	NCKAP5
ASHGV40028084	0.000854399	0.048843614	2.3249993	down	protein_coding	NM_002846	PTPRN
ASHGV40002349	0.005664591	0.078517713	3.4052354	down	protein_coding	NM_005951	MT1H
ASHGV40052433	0.04281831	0.200846244	3.2500236	down	protein_coding	NM_021996	GBGT1
ASHGV40015435	0.036102632	0.182734528	2.2563397	down	protein_coding	NM_207117	SLC25A47

ASHGV40013119	0.008289015	0.091649926	3.5013927	down	protein_coding	NM_006646	WASF3
ASHGV40045634	0.020579977	0.141774982	3.0794431	down	protein_coding	NM_003287	TPD52L1
ASHGV40038864	0.001977599	0.055772948	2.7277973	down	protein_coding	NM_152679	SLC10A4
ASHGV40049260	0.000271828	0.034595557	2.2115424	down	protein_coding	NM_024593	EFCAB1
ASHGV40034232	0.014991586	0.120959414	2.0302112	down	protein_coding	NM_144719	CCDC13
ASHGV40030646	0.010554439	0.102668445	2.9648311	down	protein_coding	NM_153675	FOXA2
ASHGV40024870	0.00416664	0.069855329	2.9941998	down	protein_coding	ENST0000059683	AC004076.9
ASHGV40019458	0.010929004	0.104057649	2.1093729	down	protein_coding	NM_001305017	DYNLRB2
ASHGV40000883	0.010848291	0.103757983	2.7971589	down	protein_coding	NM_145861	EDARADD
ASHGV40023338	0.015964116	0.124708926	2.4216766	down	protein_coding	NM_015559	SETBP1
ASHGV40039012	0.010750797	0.103400812	5.1368596	down	protein_coding	NM_017855	ODAM
ASHGV40001357	0.009648326	0.098337216	3.5734532	down	protein_coding	NM_173160	FXYD4
ASHGV40008512	0.008006048	0.090176651	80.7608394	down	protein_coding	NM_001079808	PGA4
ASHGV40007113	0.010728467	0.103340808	5.4991227	down	protein_coding	NM_015459	ATL3
ASHGV40016381	0.002322551	0.058860464	6.1894294	down	protein_coding	NM_001937	DPT
ASHGV40018859	0.001601361	0.054536631	4.3862146	down	protein_coding	NM_024773	KDM8
ASHGV40005075	0.002606999	0.061260953	3.5297024	down	protein_coding	NM_001039762	FAM196A
ASHGV40030856	0.020718272	0.14239014	3.3147281	down	protein_coding	NM_007050	PTPRT
ASHGV40011829	0.005332129	0.076519346	2.537335	down	protein_coding	NM_001007237	IGSF3
ASHGV40006630	0.016705043	0.127466323	2.1826679	down	protein_coding	NM_001206671	RIC3
ASHGV40038431	0.000184845	0.030140601	2.594379	down	protein_coding	uc001bhd.4	BC038455
ASHGV40023925	8.23987E-05	0.028718686	2.7994313	down	protein_coding	NM_001080411	ZNF433
ASHGV40039751	0.028451068	0.162806966	2.826544	down	protein_coding	NM_001151	SLC25A4
ASHGV40047883	0.009948364	0.099667779	2.6349862	down	protein_coding	NM_182546	VSTM2A
ASHGV40012832	0.026891483	0.158931032	9.788996	down	protein_coding	NM_007084	SOX21
ASHGV40043549	0.017170883	0.128881411	5.3973712	down	protein_coding	NM_021572	ENPP5
ASHGV40051122	0.001829697	0.055772948	2.3652198	down	protein_coding	NM_004421	DVL1
ASHGV40040095	0.011584217	0.106869002	3.1727572	down	protein_coding	NM_016180	SLC45A2
ASHGV40000791	0.005263123	0.076092721	2.0107913	down	protein_coding	NM_001172651	ZNF177
ASHGV40010033	0.026808287	0.15866575	2.7408431	down	protein_coding	NM_002205	ITGA5
ASHGV40021821	0.021913052	0.14610936	3.9740446	down	protein_coding	NM_001135707	ACBD4
ASHGV40008158	0.02545228	0.154632382	4.3216583	down	protein_coding	NM_054032	MRGPRX4
ASHGV40007108	0.000802035	0.048770305	5.7897784	down	protein_coding	NM_199352	SLC22A25
ASHGV40027604	0.027727918	0.161047297	2.9073858	down	protein_coding	NM_017837	PIGV
ASHGV40050393	0.007992007	0.090176651	2.7959619	down	protein_coding	NM_014265	ADAM28
ASHGV40031380	0.022296956	0.14730461	2.1909663	down	protein_coding	NM_002196	INSM1
ASHGV40022615	0.012421363	0.109882744	6.2080756	down	protein_coding	NM_001792	CDH2
ASHGV40039766	8.66549E-05	0.028718686	2.4414225	down	protein_coding	NM_015398	FAM149A
ASHGV40028763	0.00604011	0.080593472	2.7602868	down	protein_coding	NM_152390	TMEM178A
ASHGV40009333	0.003758848	0.067705865	4.4391764	down	protein_coding	NM_013264	DDX25
ASHGV40009115	0.000965579	0.049736021	7.2281103	down	protein_coding	NM_001136105	COLCA2
ASHGV40027666	0.021700362	0.145694076	3.8654269	down	protein_coding	NM_002976	SCN7A
ASHGV40039423	0.018266012	0.133146401	2.0820442	down	protein_coding	NM_031296	RAB33B
ASHGV40037863	0.002883475	0.061986011	98.4011922	down	protein_coding	NM_031279	ETNPPL
ASHGV40006677	0.000184054	0.030140601	12.1955987	down	protein_coding	NM_000315	PTH
ASHGV40056869	0.004820335	0.073532246	2.9367301	down	protein_coding	NM_032287	LDOC1L
ASHGV40014909	0.016734447	0.12752702	2.2066337	down	protein_coding	NM_138731	MIPOL1
ASHGV40002367	0.00340876	0.064811383	3.6418279	down	protein_coding	NM_176870	MT1M
ASHGV40014912	0.001070886	0.049811075	6.8416642	down	protein_coding	NM_001310135	TTC6
ASHGV40057588	0.019101714	0.136364064	2.4950056	down	protein_coding	NM_001195081	CLDN34
ASHGV40042069	0.009316036	0.09741267	2.0439552	down	protein_coding	NM_173362	RFESD
ASHGV40015894	0.001055663	0.049811075	2.2089037	down	protein_coding	NM_004580	RAB27A
ASHGV40007886	0.002147201	0.056552005	18.6692167	down	protein_coding	NM_053280	ODF3
ASHGV40053136	0.006423941	0.082540776	3.7293156	down	protein_coding	NM_001282394	WNK2
ASHGV40042757	0.000382733	0.039736903	10.0414593	down	protein_coding	NM_015980	HMP19
ASHGV40005568	0.03788036	0.187690239	2.093876	down	protein_coding	NM_003819	PABPC4
ASHGV40017339	0.003149426	0.063464592	6.6511011	down	protein_coding	NM_002065	GLUL
ASHGV40025311	0.01851375	0.133944296	2.9558774	down	protein_coding	NM_001007525	NWD1
ASHGV40003272	0.006616823	0.083328272	6.4488179	down	protein_coding	NM_005672	PSCA
ASHGV40035213	0.019298761	0.136829914	2.8561503	down	protein_coding	NM_020775	KIAA1324
ASHGV40028074	0.003368575	0.064680656	11.5103468	down	protein_coding	NM_194302	CCDC108

ASHGV40015622	0.006632131	0.08340168	5.4603866	down	protein_coding	NM_000814	GABRB3
ASHGV40032416	0.031558914	0.171367555	2.1490306	down	protein_coding	uc021wka.2	BC031638
ASHGV40044883	0.014155069	0.117255292	2.2407814	down	protein_coding	NM_005076	CNTN2
ASHGV40012453	0.015441859	0.122372976	2.675164	down	protein_coding	NM_005584	MAB21L1
ASHGV40027655	0.002063068	0.055772948	2.1142602	down	protein_coding	NM_006922	SCN3A
ASHGV40003120	0.012541905	0.11054147	5.3174833	down	protein_coding	NM_001136002	TMEM229A
ASHGV40041824	0.012569888	0.11054147	2.4826497	down	protein_coding	NM_001113561	RNF180
ASHGV40025004	0.029352182	0.165305117	2.1723398	down	protein_coding	NM_052960	RBP7
ASHGV40008648	0.01458296	0.118943234	7.1773852	down	protein_coding	NM_198714	PTGER3
ASHGV40015844	0.033850488	0.177564383	4.0134971	down	protein_coding	NM_002112	HDC
ASHGV40014125	0.001065421	0.049811075	3.1421573	down	protein_coding	NM_005060	RORC
ASHGV40019070	0.024938017	0.152621715	2.6202959	down	protein_coding	NM_001114	ADCY7
ASHGV40040651	0.000146235	0.029433159	4.0956263	down	protein_coding	NM_001039763	TMEM232
ASHGV40046371	0.00343221	0.064941046	3.7802698	down	protein_coding	NM_001100425	KIAA0895
ASHGV40055377	0.018256685	0.133146401	2.435495	down	protein_coding	NM_003399	XPNPEP2
ASHGV40019651	0.025514583	0.154953771	2.1590599	down	protein_coding	NM_016080	GLOD4
ASHGV40054229	0.006090637	0.080880694	2.2598637	down	protein_coding	NM_001184880	PCDH19
ASHGV40014824	0.007112127	0.08550928	2.0289179	down	protein_coding	NM_004554	NFATC4
ASHGV40036847	7.39932E-05	0.028718686	5.7369651	down	protein_coding	NM_005832	KCNMB2
ASHGV40019863	0.008289283	0.091649926	2.0336032	down	protein_coding	NM_002472	MYH8
ASHGV40045061	0.000185367	0.030140601	6.0782943	down	protein_coding	NM_018960	GNMT
ASHGV40021485	0.004162659	0.069855329	2.3787722	down	protein_coding	NM_014573	TMEM97
ASHGV40016334	0.001738596	0.055333849	2.3157067	down	protein_coding	NM_002666	PLIN1
ASHGV40028428	0.0006363	0.0456565	27.6389455	down	protein_coding	NST0000039166	KFZP761K232
ASHGV40051684	0.023469554	0.149731116	2.467689	down	protein_coding	NM_001017363	ARID3C
ASHGV40056219	0.002494862	0.060086644	3.3973714	down	protein_coding	uc001dcb.1	AK123450
ASHGV40052448	0.040457359	0.194704531	2.2324968	down	protein_coding	NM_001134398	VAV2
ASHGV40048025	0.001365689	0.052512256	12.945611	down	protein_coding	NM_198853	TRIM74
ASHGV40037318	0.001414056	0.052512256	2.5753843	down	protein_coding	NM_173463	CCDC149
ASHGV40031969	4.64551E-05	0.028718686	2.4260205	down	protein_coding	NM_003195	TCEA2
ASHGV40005781	0.017948419	0.131971998	6.4695175	down	protein_coding	NM_004273	CHST3
ASHGV40046891	0.001725345	0.055333849	13.032272	down	protein_coding	NM_138403	MYL10
ASHGV40025940	0.0074875	0.087778347	2.3311053	down	protein_coding	NM_001195076	C19orf81
ASHGV40019784	0.002772041	0.061986011	43.9304444	down	protein_coding	NM_001438	ESRRG
ASHGV40018807	0.001013378	0.049811075	10.9778936	down	protein_coding	NM_006338	LRRN2
ASHGV40011861	0.007349388	0.086936176	2.5481772	down	protein_coding	NM_207356	C1orf174
ASHGV40033437	0.020132308	0.139962887	2.0307124	down	protein_coding	NM_003426	ZNF74
ASHGV40052549	0.00076178	0.047440361	2.640625	down	protein_coding	uc004cnd.1	PNPLA7
ASHGV40024589	0.024015837	0.150734389	22.4058006	down	protein_coding	NM_003167	SULT2A1
ASHGV40037798	0.004432482	0.071459267	14.5169668	down	protein_coding	NM_000673	ADH7
ASHGV40010724	0.001223693	0.050556396	2.5993148	down	protein_coding	NM_032554	HCAR1
ASHGV40012893	0.041029995	0.196484206	3.2449032	down	protein_coding	NM_052867	NALCN
ASHGV40050735	0.021840531	0.145952783	3.1128374	down	protein_coding	NM_001282695	FAM107B
ASHGV40030783	0.022652516	0.147893728	3.6629753	down	protein_coding	NM_178033	CYP4X1
ASHGV40055403	0.003362841	0.064680656	7.3636302	down	protein_coding	NM_001101357	CCDC160
ASHGV40032618	0.00305781	0.062716466	2.9868907	down	protein_coding	NM_058182	SMIM11
ASHGV40018953	0.012384347	0.109722298	3.4048999	down	protein_coding	NM_152491	PM20D1
ASHGV40012084	0.008758357	0.093975821	2.1437982	down	protein_coding	NM_012240	SIRT4
ASHGV40037910	0.002872575	0.061986011	3.810988	down	protein_coding	NM_152402	TRAM1L1
ASHGV40044262	0.046633466	0.209075944	2.0194908	down	protein_coding	NM_182961	SYNE1
ASHGV40003248	0.006115316	0.081084905	3.6540995	down	protein_coding	NM_002371	MAL
ASHGV40037183	0.008111821	0.090723357	3.1322194	down	protein_coding	NM_018659	CYTL1
ASHGV40025808	0.002822383	0.061986011	11.1205127	down	protein_coding	NST0000059835	L47234.1
ASHGV40003194	0.005794365	0.07942653	2.0479248	down	protein_coding	NM_001256141	FSBP
ASHGV40050948	0.035546978	0.181386509	3.9551735	down	protein_coding	NM_004102	FABP3
ASHGV40054259	0.002888463	0.061986011	3.968376	down	protein_coding	NM_001012978	BEX5
ASHGV40021937	0.000385154	0.039736903	18.8791224	down	protein_coding	uc002ism.3	BC131755
ASHGV40008447	0.044991656	0.205686042	2.1661243	down	protein_coding	NM_001105565	SMTNL1
ASHGV40049361	0.019947242	0.139231431	4.0782285	down	protein_coding	NM_004056	CA8
ASHGV40057239	0.000210757	0.031004988	20.6592456	down	protein_coding	NM_000865	HTR1E
ASHGV40033338	0.002260928	0.058058229	21.7140578	down	protein_coding	NM_001001694	IL17REL

ASHGV40038372	0.003404535	0.064805109	3.4211544	down	protein_coding	NM_021069	SORBS2
ASHGV40009653	0.044992347	0.205686042	2.2421033	down	protein_coding	NM_152321	ERP27
ASHGV40008282	0.045456332	0.20669613	2.1539614	down	protein_coding	NM_003477	PDHX
ASHGV40043052	0.040245828	0.194333379	2.1134169	down	protein_coding	NM_017673	SWT1
ASHGV40017535	0.010003751	0.099671657	17.4818367	down	protein_coding	NM_000243	MEFV
ASHGV40005872	0.033546547	0.176836053	2.2780674	down	protein_coding	NM_005411	SFTPA1
ASHGV40024100	0.032961783	0.175433959	2.6049706	down	protein_coding	NM_007263	COPE
ASHGV40039558	0.013991092	0.11674283	2.874879	down	protein_coding	NM_000824	GLRB
ASHGV40035345	0.005034445	0.074834253	2.80751	down	protein_coding	NM_005688	ABCC5
ASHGV40007613	0.032712759	0.17477812	3.5552841	down	protein_coding	NM_006235	POU2AF1
ASHGV40057806	0.00595991	0.080368903	10.0154428	down	protein_coding	ENST0000044096	FOLR4
ASHGV40021019	0.03232126	0.173507846	2.1129318	down	protein_coding	NM_016286	DCXR
ASHGV40040661	0.024617911	0.152212021	2.6686114	down	protein_coding	NM_022140	EPB41L4A
ASHGV40017710	0.001469074	0.052900205	4.0270921	down	protein_coding	NM_052956	ACSM1
ASHGV40043104	0.00174376	0.055333849	12.2782029	down	protein_coding	NM_000948	PRL
ASHGV40035985	0.002429357	0.059482588	2.5718523	down	protein_coding	NM_203424	IQCF2
ASHGV40052188	0.0016283	0.054621802	22.7670135	down	protein_coding	ENST0000045768	C9orf147
ASHGV40019818	0.01540362	0.122358778	2.0291203	down	protein_coding	NM_002616	PER1
ASHGV40043608	0.017319539	0.129328049	3.150225	down	protein_coding	uc003pbg.3	AK125212
ASHGV40007896	7.8117E-05	0.028718686	2.0047636	down	protein_coding	NM_002228	JUN
ASHGV40019151	0.024441955	0.151715886	2.7941651	down	protein_coding	ENST0000021916	MT4
ASHGV40043455	0.005879307	0.079962247	2.7858087	down	protein_coding	NM_020737	LRFN2
ASHGV40029257	0.002541314	0.060616064	8.584758	down	protein_coding	NM_001011552	SLC9A4
ASHGV40055825	0.007045675	0.085147057	3.1874807	down	protein_coding	NM_005398	PPP1R3C
ASHGV40022186	0.001813093	0.05576279	2.1273773	down	protein_coding	NM_023036	DNAI2
ASHGV40043939	0.00152307	0.053757592	3.7535267	down	protein_coding	NM_001145128	AK9
ASHGV40028518	0.025198575	0.153806697	2.2406314	down	protein_coding	NM_021643	TRIB2
ASHGV40049007	0.003498523	0.065507718	2.3001427	down	protein_coding	NM_144962	PEBP4
ASHGV40048948	0.020928589	0.14321463	3.397543	down	protein_coding	NM_019851	FGF20
ASHGV40008034	0.011318892	0.105441158	9.2418741	down	protein_coding	NM_176875	CCKBR
ASHGV40027119	0.019974634	0.139311089	3.0709065	down	protein_coding	NM_005735	ACTR1B
ASHGV40043601	0.013991174	0.11674283	5.4196413	down	protein_coding	NM_000846	GSTA2
ASHGV40054156	8.86961E-05	0.028718686	8.618786	down	protein_coding	NM_144969	ZDHHC15
ASHGV40018427	0.016518282	0.126710411	2.6363117	down	protein_coding	NM_178310	SNAI3
ASHGV40034383	6.91265E-05	0.028718686	6.0382857	down	protein_coding	NM_001174051	CACNA2D2
ASHGV40038153	0.044425125	0.204594947	4.361676	down	protein_coding	NM_017639	DCHS2
ASHGV40038435	0.002744365	0.061986011	2.2521286	down	protein_coding	NM_182524	ZNF595
ASHGV40056941	0.003288958	0.064600323	3.4894474	down	protein_coding	NM_000685	AGTR1
ASHGV40021303	0.036778148	0.18437717	2.9244568	down	protein_coding	NM_001372	DNAH9
ASHGV40052587	0.002396612	0.059482588	3.3572103	down	protein_coding	NM_003383	VLDLR
ASHGV40031435	0.002059566	0.055772948	2.8015167	down	protein_coding	NM_024893	SYNDIG1
ASHGV40031589	0.005256076	0.076092721	2.1462839	down	protein_coding	NM_001164431	ARHGAP40
ASHGV40034375	0.022576674	0.147683655	2.3747595	down	protein_coding	NM_033159	HYAL1
ASHGV40014151	0.012897766	0.112043699	3.0533778	down	protein_coding	NM_005982	SIX1
ASHGV40032046	0.006457496	0.082800205	2.5295255	down	protein_coding	NM_003689	AKR7A2
ASHGV40020254	0.006154045	0.081250308	3.4046998	down	protein_coding	NM_017982	SUSD4
ASHGV40024656	0.004517273	0.071864614	12.8323338	down	protein_coding	NM_052884	SIGLEC11
ASHGV40031660	0.005913482	0.080044754	3.7347955	down	protein_coding	NM_022358	KCNK15
ASHGV40023200	0.001953656	0.055772948	3.260656	down	protein_coding	ENST0000057983	RP11-595B24.2
ASHGV40008587	0.010174777	0.100685249	2.0303996	down	protein_coding	NM_004470	FKBP2
ASHGV40010558	0.002062533	0.055772948	5.6161201	down	protein_coding	NM_018711	SVOP
ASHGV40055553	0.004061711	0.069284044	21.6493229	down	protein_coding	NM_005629	SLC6A8
ASHGV40032292	0.013206297	0.11335758	2.3985269	down	protein_coding	NM_005656	TMPRSS2
ASHGV40036374	0.007816052	0.089717373	2.5798995	down	protein_coding	NM_017699	SIDT1
ASHGV40024473	0.002065738	0.055772948	4.852977	down	protein_coding	NM_145296	CADM4
ASHGV40046654	0.010621677	0.102908278	8.399303	down	protein_coding	uc003txz.1	TRIM50
ASHGV40056171	0.002874254	0.061986011	2.2058788	down	protein_coding	NM_001301268	INAFM2
ASHGV40024397	0.007031718	0.085147057	2.3180283	down	protein_coding	NM_181882	PRX
ASHGV40047539	0.00924085	0.096945528	2.0004352	down	protein_coding	NM_006542	SPHAR
ASHGV40014152	0.049287094	0.214093116	2.0806317	down	protein_coding	NM_017420	SIX4
ASHGV40011565	0.009409069	0.097468969	2.9498733	down	protein_coding	NM_005981	TSPAN31

ASHGV40006892	0.009343624	0.097468969	4.8671848	down	protein_coding	NM_006034	TP53I11
ASHGV40019155	0.027783073	0.16112465	3.0839809	down	protein_coding	NM_005946	MT1A
ASHGV40056231	0.002983294	0.06249329	2.1700574	down	protein_coding	NM_004675	DIRAS3
ASHGV40031031	0.049449911	0.214479761	2.6101219	down	protein_coding	NM_003657	BCAS1
ASHGV40053791	0.032214159	0.173146141	2.5915124	down	protein_coding	NM_001031739	ASB9
ASHGV40017818	0.001495081	0.053309279	12.071717	down	protein_coding	NM_175900	C16orf54
ASHGV40052661	0.017292335	0.129292086	2.0239596	down	protein_coding	NM_144569	SPOCD1
ASHGV40009906	0.010920777	0.104057649	3.3004995	down	protein_coding	NM_002289	LALBA
ASHGV40003302	0.028471404	0.16286988	2.6105939	down	protein_coding	NM_022804	SNURF
ASHGV40010600	0.001957675	0.055772948	2.9716374	down	protein_coding	NM_138341	TMEM116
ASHGV40030562	0.034995109	0.180045654	4.4203349	down	protein_coding	NM_013281	FLRT3
ASHGV40021355	0.037594109	0.186916924	2.902727	down	protein_coding	NM_000676	ADORA2B
ASHGV40027781	0.023765485	0.150081902	3.0065103	down	protein_coding	NM_177424	STX12
ASHGV40019825	0.02621301	0.156953997	2.093592	down	protein_coding	NM_201520	SLC25A35
ASHGV40033751	0.014034759	0.1168205	4.7446983	down	protein_coding	NM_019008	MIEF1
ASHGV40045721	0.046462798	0.209061114	2.7815596	down	protein_coding	NM_000288	PEX7
ASHGV40003215	0.009986463	0.099667779	8.9483884	down	protein_coding	NM_212557	AMTN
ASHGV40038645	0.001794133	0.055586762	3.4412917	down	protein_coding	uc003gox.1	BC010030
ASHGV40014017	0.00014776	0.029433159	2.4987977	down	protein_coding	NM_001113498	MDGA2
ASHGV40055153	0.012839081	0.111707691	2.0866548	down	protein_coding	NM_019117	KLHL4
ASHGV40002020	0.02537255	0.154462892	2.0285836	down	protein_coding	NM_198181	GOLGA6L9
ASHGV40037953	0.004217049	0.07035008	2.1592284	down	protein_coding	NM_012113	CA14
ASHGV40031550	0.018147133	0.13277944	2.3014828	down	protein_coding	NM_012156	EPB41L1
ASHGV40034455	0.013775763	0.115923846	2.5022781	down	protein_coding	NM_003865	HESX1
ASHGV40012434	0.006477154	0.082800205	3.4813496	down	protein_coding	NM_001136571	ZAR1L
ASHGV40039668	0.003675432	0.067174869	2.3403566	down	protein_coding	NM_170710	WDR17
ASHGV40008463	0.016621911	0.127281349	5.5104613	down	protein_coding	NM_001220494	GLYATL1
ASHGV40020701	5.13628E-06	0.017911235	3.6929449	down	protein_coding	NM_000334	SCN4A
ASHGV40041667	0.006637872	0.08340168	5.1532372	down	protein_coding	NM_000163	GHR
ASHGV40042610	0.022016939	0.146410124	2.6934694	down	protein_coding	NM_001291722	CYFIP2
ASHGV40040519	0.02431179	0.151674021	4.0218675	down	protein_coding	NM_001289007	TMEM161B
ASHGV40035882	0.038819124	0.190411825	2.1078891	down	protein_coding	NM_144638	TMEM42
ASHGV40022309	0.000530942	0.043259351	3.3674781	down	protein_coding	NM_030968	C1QTNF1
ASHGV40055199	0.027660563	0.160763192	2.9500462	down	protein_coding	NM_145170	CFAP70
ASHGV40034474	0.03285502	0.17502601	2.7589329	down	protein_coding	NM_007177	FAM107A
ASHGV40039149	0.049673454	0.214914727	2.0099945	down	protein_coding	NM_014606	HERC3
ASHGV40034755	0.047426775	0.210367748	2.162649	down	protein_coding	NM_001008784	CD200R1L
ASHGV40008511	0.00904921	0.095857856	43.0807792	down	protein_coding	NM_001079807	PGA3
ASHGV40055353	0.006252189	0.081506727	2.5884819	down	protein_coding	NM_000828	GRIA3
ASHGV40043607	0.003363486	0.064680656	3.3387925	down	protein_coding	NM_001512	GSTA4
ASHGV40026045	0.002071903	0.055772948	2.3695673	down	protein_coding	NM_052925	LENG8
ASHGV40031376	0.003951337	0.068946524	4.0356824	down	protein_coding	NM_020689	SLC24A3
ASHGV40042362	0.006705743	0.083754019	3.7232875	down	protein_coding	NM_001135940	MYOT
ASHGV40006293	0.022468973	0.147541072	2.3264978	down	protein_coding	NM_001030059	PPAPDC1A
ASHGV40056485	0.003748809	0.067664841	24.229717	down	protein_coding	NM_018083	ZNF358
ASHGV40037450	0.011312683	0.105441158	4.4859536	down	protein_coding	NM_198353	KCTD8
ASHGV40029626	0.016891149	0.12782262	3.8920684	down	protein_coding	NM_177964	LYPD6B
ASHGV40010003	0.018505376	0.133939281	2.0307338	down	protein_coding	NM_001244705	CSAD
ASHGV40035856	0.003304344	0.064680656	2.3595934	down	protein_coding	NM_207404	ZNF662
ASHGV40005948	0.004537851	0.071864614	29.1068845	down	protein_coding	NM_001010939	LIPJ
ASHGV40019157	0.0061498	0.081250308	3.9339044	down	protein_coding	NM_005949	MT1F
ASHGV40043932	0.010800754	0.103530481	2.2004708	down	protein_coding	NM_001199933	SESN1
ASHGV40024939	0.025981617	0.1560508	2.4097473	down	protein_coding	NM_002777	PRTN3
ASHGV40022264	0.00970515	0.098669967	2.0395201	down	protein_coding	NM_001199172	MGAT5B
ASHGV40048474	0.002402664	0.059482588	54.7106826	down	protein_coding	NM_001869	CPA2
ASHGV40055684	0.004414941	0.071459267	2.9483018	down	protein_coding	NM_033284	TBL1Y
ASHGV40041713	9.43597E-05	0.028718686	20.5265035	down	protein_coding	ENST0000023616	FMO6P
ASHGV40032900	0.006353407	0.082201623	2.700258	down	protein_coding	NM_013313	YPEL1
ASHGV40030273	0.00184107	0.055772948	2.3478292	down	protein_coding	ENST0000040990	AC106876.2
ASHGV40045622	0.00819529	0.091174268	5.4001267	down	protein_coding	NM_032471	PKIB
ASHGV40046845	0.002788898	0.061986011	2.3415545	down	protein_coding	NM_181538	GJC3

ASHGV40055841	0.006476068	0.082800205	5.9967139	down	protein_coding	NM_000770	CYP2C8
ASHGV40050305	0.021107004	0.143756832	2.6248075	down	protein_coding	NM_181723	MICU3
ASHGV40051984	0.002778027	0.061986011	2.2361161	down	protein_coding	NM_001698	AUH
ASHGV40029754	0.00549603	0.077468694	6.145565	down	protein_coding	NM_001289947	ERICH2
ASHGV40038065	0.002736531	0.061986011	3.0089044	down	protein_coding	NM_004362	CLGN
ASHGV40055798	0.000317054	0.03643396	3.1979107	down	protein_coding	NM_144590	ANKRD22
ASHGV40034526	0.00770319	0.088976529	2.0119566	down	protein_coding	NM_003848	SUCLG2
ASHGV40022662	0.00963685	0.098319557	2.72585	down	protein_coding	NM_003787	NOL4
ASHGV40034848	0.013426467	0.114197014	6.7045881	down	protein_coding	NM_012190	ALDH1L1
ASHGV40014908	0.005564099	0.077963502	2.3455126	down	protein_coding	ENST0000055666	SLC25A21-AS1
ASHGV40051050	0.005189497	0.075909447	4.3193479	down	protein_coding	NM_014677	RIMS2
ASHGV40054675	0.01579478	0.123829936	2.3934017	down	protein_coding	ENST0000044530	AC002365.1
ASHGV40024686	0.002338012	0.058995057	5.7381099	down	protein_coding	NM_144947	KLK11
ASHGV40005753	0.002025905	0.055772948	9.2543658	down	protein_coding	NM_145306	C10orf35
ASHGV40054212	0.000776562	0.047935729	2.5219278	down	protein_coding	NM_014836	RHOBTB1
ASHGV40050262	2.51241E-05	0.025539914	23.944233	down	protein_coding	NM_054028	SLC35G5
ASHGV40054753	0.004094036	0.069304474	4.6281299	down	protein_coding	NM_014927	CNKSR2
ASHGV40055276	0.04582863	0.207378474	2.7666145	down	protein_coding	NM_000495	COL4A5
ASHGV40040398	0.003535139	0.065507718	4.635601	down	protein_coding	NM_016591	GCNT4
ASHGV40018816	0.001049876	0.049811075	2.2640481	down	protein_coding	NM_001039	SCNN1G
ASHGV40010145	0.024201509	0.151340367	3.575196	down	protein_coding	NM_178539	FAM19A2
ASHGV40015882	0.002204111	0.057359512	2.1117653	down	protein_coding	NM_001286495	FAM214A
ASHGV40003293	0.002534074	0.060555796	47.685304	down	protein_coding	NM_019845	RPRM
ASHGV40005840	0.002441903	0.059482588	3.0404195	down	protein_coding	NM_032024	C10orf11
ASHGV40001384	0.029778507	0.166580805	4.9746554	down	protein_coding	NM_001216	CA9
ASHGV40041416	0.001072918	0.049811075	8.4403919	down	protein_coding	NM_139056	ADAMTS16
ASHGV40029770	0.024709406	0.152291693	2.9891064	down	protein_coding	NM_007023	RAPGEF4
ASHGV40021391	0.003054699	0.062716466	2.5103547	down	protein_coding	NM_001388	DRG2
ASHGV40033476	0.026051646	0.156363685	3.0592362	down	protein_coding	NM_021233	DNASE2B
ASHGV40053579	0.000416405	0.040349735	2.6159862	down	protein_coding	NM_139026	ADAMTS13
ASHGV40035392	0.016047708	0.124970003	11.2644199	down	protein_coding	NM_001048	SST
ASHGV40015109	0.00019864	0.031004988	2.493405	down	protein_coding	ENST0000029870	PPP1R36
ASHGV40006725	0.039099871	0.191207612	2.0900857	down	protein_coding	NM_001243728	ATPAF1
ASHGV40014210	0.001768903	0.055403878	7.7259227	down	protein_coding	NM_001122965	RPTN
ASHGV40035435	0.010696945	0.103322146	6.5767385	down	protein_coding	NM_032279	ATP13A4
ASHGV40054024	0.03581291	0.182103792	2.3673025	down	protein_coding	NM_173358	SSX7
ASHGV40015365	0.019256163	0.136825495	4.5203823	down	protein_coding	NM_006215	SERPINA4
ASHGV40057337	0.001999168	0.055772948	7.9210433	down	protein_coding	ENST0000043779	TRIM73
ASHGV40006925	0.000114944	0.028925399	15.2118235	down	protein_coding	NM_001302489	ACP2
ASHGV40041324	0.002879442	0.061986011	11.0554291	down	protein_coding	NM_018140	CEP72
ASHGV40048326	0.007746482	0.089215209	2.4357714	down	protein_coding	NM_001035	RYR2
ASHGV40011844	0.011176527	0.104993761	15.3286084	down	protein_coding	ENST0000055309	RP11-536G4.1
ASHGV40004937	0.023642157	0.149889765	2.1399051	down	protein_coding	NM_198060	NRAP
ASHGV40033712	0.000656562	0.046153893	3.5008381	down	protein_coding	NM_003614	GALR3
ASHGV40047684	0.009553392	0.097984082	3.8427953	down	protein_coding	NM_194300	CCDC129
ASHGV40011714	0.000456309	0.041719107	2.9463149	down	protein_coding	NM_001270396	GLIPR1L2
ASHGV40036780	0.005005588	0.074655993	2.9623846	down	protein_coding	NM_005025	SERPINI1
ASHGV40046428	0.015336255	0.122207594	2.2058176	down	protein_coding	NM_001029882	AHDC1
ASHGV40013486	0.048901756	0.213203874	2.2361141	down	protein_coding	NM_144595	SLAIN1
ASHGV40033614	0.00106854	0.049811075	2.86617	down	protein_coding	NM_006932	SMTN
ASHGV40042943	0.012512733	0.11041094	3.1136073	down	protein_coding	NM_206836	ECI2
ASHGV40048154	0.007170064	0.085745706	4.6415623	down	protein_coding	NM_017650	PPP1R9A
ASHGV40020582	0.003985717	0.068983335	2.1926983	down	protein_coding	NM_018286	TMEM100
ASHGV40025537	0.002959237	0.06224035	2.7746156	down	protein_coding	NM_001129994	KCTD15
ASHGV40035175	0.048767434	0.213003249	5.0387202	down	protein_coding	NM_001040100	SPTSSB
ASHGV40024784	0.003543951	0.065596959	2.5029051	down	protein_coding	NM_016831	PER3
ASHGV40013731	0.001634009	0.054621802	11.7309206	down	protein_coding	NM_024979	MCF2L
ASHGV40015811	0.006754562	0.083875749	4.2563328	down	protein_coding	NM_001276266	DUOXA1
ASHGV40003358	0.014208766	0.117525634	2.5495806	down	protein_coding	NM_183058	LYZL2
ASHGV40006490	0.036380628	0.183586631	6.4682122	down	protein_coding	NM_005961	MUC6
ASHGV40008553	0.000290065	0.035122006	5.9006707	down	protein_coding	NM_002394	SLC3A2

ASHGV40044019	0.004178784	0.069991624	2.5351714	down	protein_coding	NM_001042475	CEP85L
ASHGV40022947	0.011033201	0.104381383	3.8818672	down	protein_coding	NM_001044369	FAM69C
ASHGV40054654	0.000137477	0.029433159	3.8663991	down	protein_coding	NM_000351	STS
ASHGV40032280	0.012111941	0.10897528	2.2228848	down	protein_coding	NM_005012	ROR1
ASHGV40025625	0.002518911	0.060406687	2.1274452	down	protein_coding	NM_198539	ZNF568
ASHGV40018632	0.027185637	0.159437861	2.8190247	down	protein_coding	NM_002023	FMOD
ASHGV40026881	0.003150443	0.063464592	22.3694585	down	protein_coding	ENST0000033564	C1orf213
ASHGV40021911	0.008650245	0.093622391	8.2718257	down	protein_coding	NM_138281	DLX4
ASHGV40016526	0.007824508	0.089754791	2.9634084	down	protein_coding	uc010ayj.3	HBT8
ASHGV40043902	0.013814031	0.116063883	8.3404421	down	protein_coding	NM_022361	POPDC3
ASHGV40019152	0.006249398	0.081506727	3.2443687	down	protein_coding	NM_005954	MT3
ASHGV40025899	0.00496542	0.074201092	2.0759761	down	protein_coding	NM_017636	TRPM4
ASHGV40028489	0.031289705	0.170356921	2.2757851	down	protein_coding	NM_002149	HPCAL1
ASHGV40054499	0.015631511	0.123437965	2.1691353	down	protein_coding	NM_173078	SLITRK4
ASHGV40056238	0.007367786	0.087035721	2.2971677	down	protein_coding	NM_021195	CLDN6
ASHGV40044527	0.010192935	0.100807724	3.693242	down	protein_coding	NM_001085401	C6orf201
ASHGV40008561	0.026159385	0.156740565	2.088448	down	protein_coding	NM_033101	LGALS12
ASHGV40033419	0.00802229	0.090230383	7.9235687	down	protein_coding	NM_080646	TBX1
ASHGV40038905	0.018226908	0.133118163	3.5381143	down	protein_coding	NM_000222	KIT
ASHGV40049059	0.034977936	0.180012924	2.1051266	down	protein_coding	NM_001010906	NUGGC
ASHGV40057279	0.012230662	0.109374724	4.6623328	down	protein_coding	NM_001276687	MT1HL1
ASHGV40028270	0.020230182	0.140307658	2.4524859	down	protein_coding	NM_022449	RAB17
ASHGV40018817	0.010095242	0.100190822	2.5661168	down	protein_coding	NM_000336	SCNN1B
ASHGV40018012	0.033346392	0.176201594	2.027554	down	protein_coding	NM_024336	IRX3
ASHGV40048579	0.003027672	0.062622161	5.3537857	down	protein_coding	NM_004445	EPHB6
ASHGV40021250	0.049779165	0.215211882	4.2920743	down	protein_coding	uc002gjx.1	AX747630
ASHGV40003164	0.025961211	0.155981967	3.2014076	down	protein_coding	NM_152487	TMEM56
ASHGV40032135	0.016711861	0.127466323	6.1733332	down	protein_coding	ENST0000059371	AL138847.1
ASHGV40037300	0.026428526	0.157433474	2.6538663	down	protein_coding	NM_025221	KCNIP4
ASHGV40032392	0.002720273	0.061986011	5.9528719	down	protein_coding	ENST0000059620	PRED58
ASHGV40023754	0.000677644	0.046153893	16.912458	down	protein_coding	NM_001013706	PLIN5
ASHGV40036379	0.011731646	0.107772913	3.1784532	down	protein_coding	NM_017577	GRAMD1C
ASHGV40012569	0.000121927	0.029122276	2.0584012	down	protein_coding	NM_001079670	CAB39L
ASHGV40041234	0.011620235	0.107039132	3.9423098	down	protein_coding	NM_016222	DDX41
ASHGV40017702	0.006566443	0.083034206	2.3951953	down	protein_coding	NM_016235	GPRC5B
ASHGV40049199	1.8389E-06	0.008015752	5.4571073	down	protein_coding	NM_024645	ZMAT4
ASHGV40043604	0.012774584	0.111313165	4.7630639	down	protein_coding	NM_153699	GSTA5
ASHGV40004977	0.005261211	0.076092721	4.1164851	down	protein_coding	NM_014904	RAB11FIP2
ASHGV40014126	0.013417813	0.114197014	3.6138645	down	protein_coding	NM_001001872	C14orf37
ASHGV40030217	0.046344788	0.208856996	2.3771911	down	protein_coding	NM_000091	COL4A3
ASHGV40035896	0.003806393	0.067881807	7.6508056	down	protein_coding	NM_001123041	CCR2
ASHGV40037097	0.019106298	0.136364064	2.2503771	down	protein_coding	NM_006472	TXNIP
ASHGV40036581	0.033274842	0.17607895	2.8014348	down	protein_coding	NM_031913	ESYT3
ASHGV40032368	0.003040968	0.062716466	119.6030442	down	protein_coding	NM_198693	KRTAP10-2
ASHGV40022051	0.029341173	0.16529651	3.8815644	down	protein_coding	NM_000717	CA4
ASHGV40038070	0.01620437	0.125579029	2.2936796	down	protein_coding	NM_015130	TBC1D9
ASHGV40011187	0.015289632	0.122176915	4.000931	down	protein_coding	NM_001029874	REP15
ASHGV40025927	0.003254717	0.064458674	3.5642891	down	protein_coding	NM_012068	ATF5
ASHGV40046954	0.004426774	0.071459267	7.0297053	down	protein_coding	NM_007356	LAMB4
ASHGV40016812	0.000483834	0.041928091	9.5431689	down	protein_coding	NM_013243	SCG3
ASHGV40056868	0.004774106	0.073532246	2.1204362	down	protein_coding	NM_001284334	SERHL2
ASHGV40038223	0.034683426	0.179356685	2.254345	down	protein_coding	NM_001100389	TMEM192
ASHGV40024634	0.006056318	0.08067071	2.3880695	down	protein_coding	NM_014037	SLC6A16
ASHGV40000454	0.006993846	0.085038143	3.5781991	down	protein_coding	NM_001271641	MTCH1
ASHGV40021963	0.000476617	0.041888197	3.553572	down	protein_coding	ENST0000041236	AC102948.2
ASHGV40032417	0.000746174	0.047310137	7.1905973	down	protein_coding	NM_002303	LEPR
ASHGV40044971	0.03429258	0.178694547	3.4801098	down	protein_coding	NM_001145717	PNPLA1
ASHGV40046740	0.003314551	0.064680656	2.8962958	down	protein_coding	NM_152754	SEMA3D
ASHGV40027832	0.003290037	0.064600323	13.1996247	down	protein_coding	NM_018053	XKR8
ASHGV40022229	0.004809616	0.073532246	2.1171013	down	protein_coding	NM_001162995	SMIM5
ASHGV40016163	0.005974141	0.080436393	6.7508322	down	protein_coding	NM_138573	NRG4

ASHGV40055099	0.035265083	0.180688213	2.0590597	down	protein_coding	NM_145052	UPRT
ASHGV40025233	0.000880982	0.049190557	2.6274151	down	protein_coding	NM_002501	NFIX
ASHGV40029862	0.010373912	0.10184658	4.3687766	down	protein_coding	NM_080876	DUSP19
ASHGV40019256	0.035906222	0.182365538	3.7380952	down	protein_coding	NM_005182	CA7
ASHGV40032412	0.003022445	0.062622161	11.4479009	down	protein_coding	NM_002340	LSS
ASHGV40039570	0.006541398	0.083034206	2.5155864	down	protein_coding	NM_021634	RXFP1
ASHGV40013103	0.011244572	0.105295574	2.3449528	down	protein_coding	NM_178540	C1QTNF9
ASHGV40023708	0.024667065	0.152291693	2.4800162	down	protein_coding	NM_052847	GNG7
ASHGV40030834	0.003734414	0.067544862	3.1901186	down	protein_coding	NM_004474	FOXD2
ASHGV40035415	0.004063252	0.069284044	2.7333503	down	protein_coding	ENST0000042600	LEPREL1
ASHGV40030239	0.009899521	0.099393971	3.7156529	down	protein_coding	NM_030926	ITM2C
ASHGV40011546	9.61474E-05	0.028718686	2.9284709	down	protein_coding	ENST0000059800	AC126614.1
ASHGV40049533	0.02230352	0.14730461	2.9285616	down	protein_coding	NM_001442	FABP4
ASHGV40006711	0.004275044	0.070586813	3.9015855	down	protein_coding	NM_004179	TPH1
ASHGV40043700	0.015735813	0.123701371	2.0049219	down	protein_coding	NM_001080507	OOEP
ASHGV40009733	0.012065157	0.108852935	3.3673647	down	protein_coding	NM_001242672	TTC34
ASHGV40015538	0.008909943	0.095133965	2.184742	down	protein_coding	NM_001135050	IGSF9
ASHGV40036992	0.00379655	0.067881807	4.9782286	down	protein_coding	NM_020386	HRASLS
ASHGV40007785	0.010797609	0.103530481	6.0704035	down	protein_coding	NM_014312	VSIG2
ASHGV40037886	0.006967219	0.085010804	2.4776599	down	protein_coding	NM_052864	TIFA
ASHGV40009477	0.001636554	0.054621802	2.155976	down	protein_coding	NM_001278309	AKAP3
ASHGV40049655	0.016264602	0.125739087	2.2710847	down	protein_coding	NM_033285	TP53INP1
ASHGV40013622	0.00927761	0.0971558	2.1511906	down	protein_coding	NM_000282	PCCA
ASHGV40019154	0.002794107	0.061986011	3.8765055	down	protein_coding	NM_175617	MT1E
ASHGV40049759	0.003357889	0.064680656	2.1166012	down	protein_coding	NM_001146	ANGPT1
ASHGV40040138	0.002413734	0.059482588	9.1156732	down	protein_coding	NM_002310	LIFR
ASHGV40026736	0.009547149	0.097977685	6.0805313	down	protein_coding	NM_004801	NRXN1
ASHGV40048132	0.024705458	0.152291693	2.1533726	down	protein_coding	uc001hxp.1	AX747246
ASHGV40051831	0.001253031	0.05083886	2.042873	down	protein_coding	NM_001206	KLF9
ASHGV40057432	0.004266711	0.070586813	2.4091655	down	protein_coding	uc003xki.3	GPR124
ASHGV40026188	0.005457949	0.07736975	6.9658726	down	protein_coding	NM_001002919	FAM150B
ASHGV40011465	0.001600099	0.054536631	2.9730385	down	protein_coding	NM_018953	HOXC5
ASHGV40050765	0.001193833	0.050120601	4.7798972	down	protein_coding	NM_144650	ADHFE1
ASHGV40048273	0.002820525	0.061986011	5.821055	down	protein_coding	NM_001031692	LRRC17
ASHGV40018964	0.002078637	0.055772948	3.8305688	down	protein_coding	NM_052934	SLC26A9
ASHGV40018596	0.007385642	0.08718758	5.0004687	down	protein_coding	NM_001135086	PRSS41
ASHGV40054934	0.000399534	0.03988564	2.1220691	down	protein_coding	NM_001079900	SPACA5B
ASHGV40053340	0.008186682	0.091174268	10.5690237	down	protein_coding	NM_000608	ORM2
ASHGV40027040	0.006332904	0.082097034	2.6814912	down	protein_coding	NM_001768	CD8A
ASHGV40013101	0.007711077	0.088981032	2.529874	down	protein_coding	uc001eqa.3	NBPF24
ASHGV40018319	0.014066389	0.116958299	3.1927627	down	protein_coding	NM_145168	SDR42E1
ASHGV40052941	0.00243061	0.059482588	3.8727558	down	protein_coding	NM_153267	MAMDC2
ASHGV40033473	0.003116212	0.063152685	4.1586974	down	protein_coding	NM_007128	VPREB1
ASHGV40034534	0.003260642	0.064458674	3.4696457	down	protein_coding	NM_017734	PALMD
ASHGV40007437	0.000202222	0.031004988	3.8698387	down	protein_coding	NM_006680	ME3
ASHGV40024808	9.61221E-05	0.028718686	40.4356122	down	protein_coding	NM_003180	SYT5
ASHGV40031321	0.024381985	0.151714606	2.6892331	down	protein_coding	NM_001282550	BTBD3
ASHGV40046846	0.000908695	0.049204994	4.3954842	down	protein_coding	NM_001185	AZGP1
ASHGV40032745	0.020899503	0.143184179	3.1684987	down	protein_coding	uc002zey.1	TRPM2
ASHGV40054583	0.005736292	0.079128149	2.9966114	down	protein_coding	NM_145178	ATOH7
ASHGV40022445	0.009219092	0.096775488	2.5076883	down	protein_coding	NM_173464	L3MBTL4
ASHGV40023034	0.000776218	0.047935729	19.1853886	down	protein_coding	NM_014410	CLUL1
ASHGV40053770	0.001342415	0.052312433	2.9407481	down	protein_coding	NM_013427	ARHGAP6
ASHGV40019773	0.00413438	0.069649315	3.181455	down	protein_coding	NM_007123	USH2A
ASHGV40037628	0.003006923	0.062622161	2.5487115	down	protein_coding	NM_201431	RASSF6
ASHGV40054117	0.003284131	0.064600323	5.5317911	down	protein_coding	NM_032803	SLC7A3
ASHGV40011143	0.007424543	0.087410087	2.9211084	down	protein_coding	NM_030572	SPX
ASHGV40016859	0.002837912	0.061986011	2.1604253	down	protein_coding	NM_032866	CGNL1
ASHGV40055253	0.000630999	0.045463199	9.2792944	down	protein_coding	NM_152423	MUM1L1
ASHGV40035434	0.000919303	0.049228681	4.7140324	down	protein_coding	NM_198505	ATP13A5
ASHGV40060858	0.002125729	0.056243113	4.3511782	down	protein_coding	uc010aiv.1	AV2S1A1

ASHGV40028005	0.00046394	0.041888197	16.3553242	down	protein_coding	NM_001608	ACADL
ASHGV40037213	0.015671568	0.123530497	2.1623732	down	protein_coding	uc003glb.1	AX746755
ASHGV40034564	0.001214168	0.0505256	8.9620743	down	protein_coding	NM_020872	CNTN3
ASHGV40037068	0.004786001	0.073532246	2.7187281	down	protein_coding	NM_001039703	NBPF10
ASHGV40012277	0.00350515	0.065507718	3.4806933	down	protein_coding	ENST0000053726	RP13-672B3.2
ASHGV40049083	0.002494995	0.060086644	5.9967746	down	protein_coding	NM_001100916	MBOAT4
ASHGV40026670	0.000477205	0.041888197	14.7635229	down	protein_coding	NM_007352	CELA3B
ASHGV40005137	0.000456874	0.041719107	3.4991159	down	protein_coding	ENST0000054015	BNIP3
ASHGV40040181	0.002572066	0.060767674	2.5911668	down	protein_coding	NM_001639	APCS
ASHGV40047612	0.043851741	0.203567349	2.2732528	down	protein_coding	NM_001127364	FAM221A
ASHGV40005317	0.026823129	0.158699722	3.5016857	down	protein_coding	NM_024693	ECHDC3
ASHGV40025411	0.001504809	0.05332898	2.4803045	down	protein_coding	NM_003429	ZNF85
ASHGV40009211	0.002694404	0.061839224	3.1763146	down	protein_coding	NM_032780	TMEM25
ASHGV40000100	0.00787913	0.089971932	2.3433335	down	protein_coding	NM_003097	SNRPN
ASHGV40001902	0.003183215	0.063576796	2.1695055	down	protein_coding	NM_001024678	LRRC24
ASHGV40010686	0.001002576	0.049811075	7.4485321	down	protein_coding	NM_000928	PLA2G1B
ASHGV40047101	0.003938046	0.068946524	2.6841317	down	protein_coding	NM_022143	LRRC4
ASHGV40049848	0.000170472	0.030140601	3.1995733	down	protein_coding	NM_021021	SNTB1
ASHGV40053829	0.003340344	0.064680656	6.6838214	down	protein_coding	NM_152780	MAP7D2
ASHGV40042841	0.009373672	0.097468969	2.0993613	down	protein_coding	NM_025158	RUFY1
ASHGV40044719	0.005009607	0.074655993	4.0694405	down	protein_coding	NM_006998	SCGN
ASHGV40039726	0.000504025	0.042546158	2.1692078	down	protein_coding	NM_020225	STOX2
ASHGV40040171	0.022730983	0.147893728	2.8250439	down	protein_coding	NM_000436	OXCT1
ASHGV40040168	0.000945584	0.049736021	11.7134794	down	protein_coding	NM_001005473	PLCXD3
ASHGV40009591	0.003982873	0.068983335	3.2220864	down	protein_coding	NM_002259	KLRC1
ASHGV40029248	0.044274375	0.204236449	2.6515585	down	protein_coding	ENST0000041400	FLJ20373
ASHGV40053970	0.005634801	0.078517713	2.3266882	down	protein_coding	NM_006950	SYN1
ASHGV40030413	0.000293692	0.035312118	6.3338741	down	protein_coding	NM_001167600	NEU4
ASHGV40010426	0.006802785	0.08406333	2.8743509	down	protein_coding	NM_021229	NTN4
ASHGV40015360	0.021597116	0.1452806	3.651057	down	protein_coding	NM_058237	PPP4R4
ASHGV40029659	0.001653197	0.054739049	2.3572506	down	protein_coding	NM_052917	GALNT13
ASHGV40024122	0.030521001	0.168406383	2.5732992	down	protein_coding	NM_033196	ZNF682
ASHGV40022330	0.002737951	0.061986011	2.9580598	down	protein_coding	NM_173627	ENDOV
ASHGV40042236	0.003382035	0.064680656	2.2599533	down	protein_coding	NM_014035	SNX24
ASHGV40026676	0.000466811	0.041888197	15.9828485	down	protein_coding	NM_005747	CELA3A
ASHGV40014820	0.004847691	0.073566912	2.3188798	up	protein_coding	NM_174913	NOP9
ASHGV40030997	0.019326453	0.136926467	3.1258621	up	protein_coding	NM_002237	KCNG1
ASHGV40053297	0.0093266	0.097434749	2.4505722	up	protein_coding	NM_007203	PALM2-AKAP2
ASHGV40050832	0.000927132	0.049284996	4.3976044	up	protein_coding	NM_031461	CRISPLD1
ASHGV40006199	0.034862173	0.179662193	3.3595278	up	protein_coding	NM_000681	ADRA2A
ASHGV40032790	0.000171443	0.030140601	2.3223489	up	protein_coding	NM_001849	COL6A2
ASHGV40057004	0.021139741	0.143756832	2.719704	up	protein_coding	NM_006607	PTTG2
ASHGV40052980	0.027150313	0.159391535	7.6027999	up	protein_coding	NM_001190482	PCSK5
ASHGV40049066	0.02876921	0.16376753	2.7333454	up	protein_coding	NM_004895	NLRP3
ASHGV40053068	0.00040032	0.03988564	2.6936552	up	protein_coding	NM_001912	CTSL
ASHGV40005970	0.020695763	0.142347659	2.2396138	up	protein_coding	NM_001284259	KIF20B
ASHGV40024683	0.004630737	0.072674639	8.0170662	up	protein_coding	NM_007196	KLK8
ASHGV40046663	0.00010586	0.028718686	73.2459309	up	protein_coding	NM_001306	CLDN3
ASHGV40054927	0.000652159	0.046153893	3.7189638	up	protein_coding	NM_003254	TIMP1
ASHGV40020293	0.009161434	0.096506793	7.1745096	up	protein_coding	NM_015515	KRT23
ASHGV40045579	0.016475346	0.126548075	2.2912703	up	protein_coding	NM_153711	FAM26E
ASHGV40040832	0.013790235	0.115943689	2.2500029	up	protein_coding	NM_138610	H2AFY
ASHGV40035881	0.027792892	0.16112465	2.9105815	up	protein_coding	NM_020242	KIF15
ASHGV40010916	0.000303882	0.036044172	2.0525099	up	protein_coding	NM_003213	TEAD4
ASHGV40025848	0.002890999	0.061986011	2.9679393	up	protein_coding	NM_018485	C5AR2
ASHGV40008896	0.003636017	0.066664143	2.0089033	up	protein_coding	NM_000260	MYO7A
ASHGV40008705	0.002554264	0.060670246	3.350223	up	protein_coding	NM_177963	SYT12
ASHGV40013689	0.018926438	0.135904775	2.1082641	up	protein_coding	NM_001846	COL4A2
ASHGV40046203	0.028782868	0.163791806	2.8572903	up	protein_coding	NM_176813	AGR3
ASHGV40041987	0.040506896	0.194835377	2.0288436	up	protein_coding	NM_022406	XRCC4
ASHGV40009920	0.006213638	0.081506727	2.3973383	up	protein_coding	NM_144593	RHEBL1

ASHGV40029684	0.002764109	0.061986011	2.7063761	up	protein_coding	NM_173355	UPP2
ASHGV40049441	0.035187953	0.180545761	2.1685178	up	protein_coding	NM_016027	LACTB2
ASHGV40040884	0.038518682	0.189707175	2.0078808	up	protein_coding	NM_001077693	ECSCR
ASHGV40043423	0.004868528	0.073623294	2.5719923	up	protein_coding	NM_145316	TMEM217
ASHGV40047719	0.030593483	0.168599574	3.5239942	up	protein_coding	NM_018685	ANLN
ASHGV40020324	0.043027672	0.201512355	2.6168087	up	protein_coding	NM_003771	KRT36
ASHGV40052545	0.001076462	0.049811075	3.077069	up	protein_coding	NM_001004354	NRARP
ASHGV40057498	0.007915894	0.089971932	3.5953599	up	protein_coding	NM_021057	IFNA7
ASHGV40011525	0.000173856	0.030140601	23.3101278	up	protein_coding	NM_173596	SLC39A5
ASHGV40052527	0.025708793	0.155406605	2.0928427	up	protein_coding	NM_203468	ENTPD2
ASHGV40030939	0.006073008	0.080769619	2.12423	up	protein_coding	NM_018837	SULF2
ASHGV40052755	0.000120699	0.029122276	88.3940288	up	protein_coding	NM_014471	SPINK4
ASHGV40009369	0.012112531	0.10897528	2.1620868	up	protein_coding	NM_138788	TMEM45B
ASHGV40014067	0.001670175	0.054739049	2.8947957	up	protein_coding	NM_007361	NID2
ASHGV40015888	0.005553619	0.077963502	19.6293842	up	protein_coding	NM_182758	WDR72
ASHGV40015836	0.005943764	0.080213215	2.3014826	up	protein_coding	NM_203349	SHC4
ASHGV40023035	0.037719813	0.187160691	2.8296617	up	protein_coding	NM_001071	TYMS
ASHGV40044128	0.013376737	0.114052217	2.7400843	up	protein_coding	NM_138419	MTFR2
ASHGV40015800	0.006028493	0.080593472	3.4873454	up	protein_coding	NM_005099	ADAMTS4
ASHGV40011670	0.014533486	0.118802563	3.707926	up	protein_coding	NM_182530	MYRFL
ASHGV40039547	0.010603817	0.102829895	5.7980319	up	protein_coding	NM_005651	TDO2
ASHGV40046589	0.007357083	0.086968201	2.0228824	up	protein_coding	ENST0000059363	AC073188.1
ASHGV40052374	0.008529845	0.092938508	2.099861	up	protein_coding	NM_203434	IER5L
ASHGV40057195	0.016527063	0.126722017	3.0298644	up	protein_coding	NM_003509	HIST1H2AI
ASHGV40040723	0.000150238	0.029433159	2.3088307	up	protein_coding	NM_004106	FCER1G
ASHGV40035961	0.017535407	0.129884177	2.1140259	up	protein_coding	NM_002070	GNAI2
ASHGV40031698	0.005966728	0.080398659	3.8255374	up	protein_coding	NM_004994	MMP9
ASHGV40037261	0.043244709	0.202094545	3.0678013	up	protein_coding	NM_005130	FGFBP1
ASHGV40027417	0.026245345	0.156959315	2.1213092	up	protein_coding	ENST0000031298	TUBA3E
ASHGV40040992	0.022770982	0.147948462	2.6507144	up	protein_coding	NM_000440	PDE6A
ASHGV40014873	0.040283894	0.194333379	2.0733809	up	protein_coding	ENST0000055333	RP11-187E13.1
ASHGV40045837	0.031188651	0.170170948	2.1209695	up	protein_coding	NM_001029884	PLEKHG1
ASHGV40035417	0.001195812	0.050120601	8.7763558	up	protein_coding	NM_021101	CLDN1
ASHGV40041316	0.038936307	0.190820817	2.1625053	up	protein_coding	NM_001242412	AHRR
ASHGV40046226	0.023193375	0.148895321	2.4224325	up	protein_coding	NM_182762	MACC1
ASHGV40003206	0.002724595	0.061986011	3.9249359	up	protein_coding	NM_001278655	LINC01272
ASHGV40017509	0.027279938	0.15979234	2.7187011	up	protein_coding	NM_031948	PRSS27
ASHGV40032331	0.045897771	0.207378474	2.7220911	up	protein_coding	NM_001308491	ERVH48-1
ASHGV40055205	0.00357198	0.065905867	4.1529801	up	protein_coding	NM_006733	CENPI
ASHGV40026217	0.001099876	0.049811075	2.4251138	up	protein_coding	NM_012293	PXDN
ASHGV40040833	0.00987168	0.099354989	2.4241751	up	protein_coding	NM_004001	FCGR2B
ASHGV40030100	0.048350078	0.212427834	2.3064944	up	protein_coding	NM_138390	TMEM169
ASHGV40047452	0.003841699	0.068142284	2.0333736	up	protein_coding	NM_003751	EIF3B
ASHGV40025961	0.00110043	0.049811075	2.8486465	up	protein_coding	NM_014385	SIGLEC7
ASHGV40025782	0.026494829	0.157559292	2.9896137	up	protein_coding	NM_001205280	IGSF23
ASHGV40042642	0.006036127	0.080593472	5.021794	up	protein_coding	NM_001445	FABP6
ASHGV40010029	0.000593978	0.044687591	3.3251168	up	protein_coding	NM_020370	GPR84
ASHGV40020830	0.047308015	0.210102535	2.0206153	up	protein_coding	NM_006678	CD300C
ASHGV40045705	0.03540664	0.181042365	4.3101193	up	protein_coding	NM_005375	MYB
ASHGV40042800	0.041279636	0.197300366	2.3142124	up	protein_coding	NM_003052	SLC34A1
ASHGV40026052	3.50524E-05	0.026561918	3.5259228	up	protein_coding	NM_001278428	LILRB4
ASHGV40049285	0.000579704	0.044687591	6.3220558	up	protein_coding	NM_207413	FAM150A
ASHGV40050035	0.00396803	0.068946524	8.9979777	up	protein_coding	NM_024803	TUBAL3
ASHGV40043143	0.001757899	0.055403878	3.2339924	up	protein_coding	NM_021018	HIST1H3F
ASHGV40039838	0.003369254	0.064680656	2.39851	up	protein_coding	NM_024830	LPCAT1
ASHGV40056384	0.041474092	0.19776566	2.6358534	up	protein_coding	NM_031961	KRTAP9-2
ASHGV40023483	0.017468394	0.12972491	17.3504107	up	protein_coding	NM_002639	SERPINB5
ASHGV40016882	0.022496524	0.147541072	5.1736349	up	protein_coding	NM_004751	GCNT3
ASHGV40050134	0.003755163	0.067705865	2.322157	up	protein_coding	NM_130849	SLC39A4
ASHGV40018610	0.000340422	0.037940966	5.608396	up	protein_coding	NM_016639	TNFRSF12A
ASHGV40006523	0.003379625	0.064680656	4.7607608	up	protein_coding	NM_007105	SLC22A18AS

ASHGV40017290	0.025705614	0.155406605	2.0114981	up	protein_coding	NM_013272	SLCO3A1
ASHGV40044095	0.006560698	0.083034206	7.4059384	up	protein_coding	NM_004666	VNN1
ASHGV40024777	0.01766946	0.130599709	2.9427318	up	protein_coding	NM_006864	LILRB3
ASHGV40050543	0.010162745	0.100623292	5.6928212	up	protein_coding	NM_153692	HTRA4
ASHGV40056552	0.046552236	0.209075944	2.6364588	up	protein_coding	NM_005267	GJA8
ASHGV40013159	0.033648887	0.177029899	2.2382701	up	protein_coding	NM_001629	ALOX5AP
ASHGV40011504	0.006640188	0.08340168	2.526057	up	protein_coding	NM_002905	RDH5
ASHGV40041212	0.006572209	0.083038437	2.0771663	up	protein_coding	NM_016391	NOP16
ASHGV40014452	0.027571201	0.160592595	2.2157787	up	protein_coding	NM_005979	S100A13
ASHGV40053398	0.001079765	0.049811075	2.1628448	up	protein_coding	NM_138777	MRRF
ASHGV40009998	0.002070869	0.055772948	2.0992674	up	protein_coding	NM_175834	KRT79
ASHGV40037929	0.017736073	0.130981013	3.1591167	up	protein_coding	NM_002358	MAD2L1
ASHGV40013251	0.024132894	0.151196959	2.4812464	up	protein_coding	NM_178009	DGKH
ASHGV40036492	0.033580189	0.176836053	2.4045237	up	protein_coding	NM_004526	MCM2
ASHGV40054949	0.003270085	0.064499089	3.417751	up	protein_coding	NM_001080489	GLOD5
ASHGV40011356	0.028557004	0.163252434	2.3458761	up	protein_coding	NM_175744	RHOC
ASHGV40005376	0.004848178	0.073566912	8.7348043	up	protein_coding	uc010qcr.1	AK297683
ASHGV40043638	0.032361575	0.1736708	2.3134415	up	protein_coding	NM_001723	DST
ASHGV40034298	0.006922959	0.084906668	2.3662465	up	protein_coding	NM_001789	CDC25A
ASHGV40052499	0.010147563	0.100530059	2.0200314	up	protein_coding	NM_017617	NOTCH1
ASHGV40003250	0.023779879	0.150118018	3.1068404	up	protein_coding	NM_002952	RPS2
ASHGV40048909	0.000843647	0.048843614	2.821595	up	protein_coding	NM_001908	CTSB
ASHGV40031953	0.006784647	0.083910159	2.2306242	up	protein_coding	NM_016434	RTEL1
ASHGV40052077	0.002174648	0.056932681	2.2210549	up	protein_coding	NM_001267571	TBC1D2
ASHGV40047828	0.002732762	0.061986011	2.4722493	up	protein_coding	uc022acp.1	AX746840
ASHGV40033138	0.024665113	0.152291693	2.8800922	up	protein_coding	NM_006498	LGALS2
ASHGV40013163	0.001105733	0.049811075	5.137388	up	protein_coding	NM_032849	MEDAG
ASHGV40047598	0.011763392	0.107782941	6.83487	up	protein_coding	NM_000600	IL6
ASHGV40040350	0.010871948	0.103870293	2.0884817	up	protein_coding	uc003kae.3	DQ591060
ASHGV40051414	0.005262301	0.076092721	2.1794351	up	protein_coding	uc003zci.3	ADCK5
ASHGV40038641	0.034686324	0.179356685	3.8325299	up	protein_coding	NM_002965	S100A9
ASHGV40053219	0.000969863	0.049736021	2.0797666	up	protein_coding	NM_006981	NR4A3
ASHGV40055584	0.002739006	0.061986011	2.5556099	up	protein_coding	NM_001288747	DKC1
ASHGV40021475	0.003170318	0.063464592	2.7818908	up	protein_coding	NM_021170	HES4
ASHGV40050573	0.011105494	0.104667783	2.9992564	up	protein_coding	NM_032336	GINS4
ASHGV40016479	0.017997608	0.132128964	4.2025609	up	protein_coding	NM_000450	SELE
ASHGV40002351	0.007475525	0.087778347	2.1524142	up	protein_coding	ENST0000056931	ATP6C
ASHGV40007359	0.000905848	0.049204994	3.8572421	up	protein_coding	NM_030792	GDPD5
ASHGV40000083	0.02211594	0.146790385	2.0224916	up	protein_coding	NM_198216	SNRPB
ASHGV40003122	0.01256633	0.11054147	2.2242233	up	protein_coding	NM_001137549	FAM25G
ASHGV40030618	0.003086908	0.062854503	2.3215846	up	protein_coding	NM_003579	RAD54L
ASHGV40019786	0.033142464	0.175924118	2.291781	up	protein_coding	NM_015982	YBX2
ASHGV40045535	0.009447875	0.097544009	14.8776724	up	protein_coding	NM_003880	WISP3
ASHGV40043001	0.001427069	0.052558935	5.9219764	up	protein_coding	NM_003220	TFAP2A
ASHGV40015501	0.001541687	0.053801741	2.2313218	up	protein_coding	uc001ymf.1	AX746522
ASHGV40019503	0.0436743	0.203016945	2.9520053	up	protein_coding	NM_001291454	ATP2C2
ASHGV40048005	0.022695069	0.147893728	3.7234801	up	protein_coding	NM_014146	LAT2
ASHGV40004948	0.00633141	0.082097034	2.8927059	up	protein_coding	NM_001001936	AFAP1L2
ASHGV40047331	0.018477936	0.133907436	2.1945943	up	protein_coding	NM_005431	XRCC2
ASHGV40028798	0.014869325	0.120362833	13.131071	up	protein_coding	NM_000341	SLC3A1
ASHGV40014362	0.048961692	0.213317355	3.6105214	up	protein_coding	NM_002964	S100A8
ASHGV40048834	1.78583E-05	0.025539914	4.1724934	up	protein_coding	NM_001147	ANGPT2
ASHGV40007348	0.000107784	0.028718686	8.5070144	up	protein_coding	NM_001278473	CHRDL2
ASHGV40042558	0.001989288	0.055772948	12.3956489	up	protein_coding	NM_001804	CDX1
ASHGV40048253	0.000407058	0.040031539	2.5145205	up	protein_coding	NM_000602	SERPINE1
ASHGV40017481	0.013338703	0.113987801	3.9092005	up	protein_coding	NM_172168	NOXO1
ASHGV40007933	0.005816428	0.079541368	11.5698065	up	protein_coding	NM_002457	MUC2
ASHGV40025793	6.18696E-05	0.028718686	4.3407501	up	protein_coding	NM_000041	APOE
ASHGV40042542	0.015878434	0.124180314	2.160408	up	protein_coding	NM_014945	ABLIM3
ASHGV40034549	0.012712779	0.111052115	5.3034282	up	protein_coding	NM_001126128	PROK2
ASHGV40049970	0.013696884	0.115315724	2.3600215	up	protein_coding	NM_012472	LRRC6

ASHGV40022327	0.000181965	0.030140601	2.9994687	up	protein_coding	NM_024110	CARD14
ASHGV40024681	0.000453532	0.041719107	76.574463	up	protein_coding	NM_005046	KLK7
ASHGV40006310	0.038224678	0.188645764	3.7400717	up	protein_coding	NM_144587	BTBD16
ASHGV40008835	0.012120591	0.108991551	3.1639695	up	protein_coding	NM_002564	P2RY2
ASHGV40020540	0.014036961	0.1168205	2.9952605	up	protein_coding	NM_001257359	SAMD14
ASHGV40037857	0.006483911	0.082800205	2.3140568	up	protein_coding	NM_016269	LEF1
ASHGV40039053	0.008466421	0.092532472	3.9512985	up	protein_coding	NM_001511	CXCL1
ASHGV40003099	0.043904414	0.203757615	2.3928592	up	protein_coding	NM_004233	CD83
ASHGV40057384	0.003701886	0.067298943	6.160027	up	protein_coding	ENST0000055046	RP11-1220K2.2
ASHGV40044764	0.002666405	0.061839224	2.5078134	up	protein_coding	NM_003495	HIST1H4I
ASHGV40057583	0.004663564	0.07280668	2.048889	up	protein_coding	NM_004192	ASMTL
ASHGV40021850	0.002994328	0.06255259	3.8036788	up	protein_coding	NM_002476	MYL4
ASHGV40016682	0.019902417	0.139020527	2.586308	up	protein_coding	NM_001130448	C15orf62
ASHGV40027823	0.019889079	0.138992374	2.1687183	up	protein_coding	NM_006287	TFPI
ASHGV40045989	0.027613707	0.16063555	2.8432877	up	protein_coding	ENST0000059727	AL009178.1
ASHGV40021647	0.001779758	0.055414038	6.7376647	up	protein_coding	NM_173625	C17orf78
ASHGV40034719	0.015173488	0.121863165	3.5471657	up	protein_coding	NM_020890	KIAA1524
ASHGV40036285	0.001975703	0.055772948	6.6212336	up	protein_coding	NM_032787	ADGRG7
ASHGV40020073	0.018801054	0.135181513	2.8790683	up	protein_coding	NM_006461	SPAG5
ASHGV40014806	0.027629388	0.16063555	2.2194279	up	protein_coding	NM_001291556	PCK2
ASHGV40013688	0.009172968	0.096523757	2.2732361	up	protein_coding	NM_030920	ANP32E
ASHGV40014681	0.001878306	0.055772948	2.5597805	up	protein_coding	NM_181864	ACOT7
ASHGV40030141	0.006780826	0.083910159	2.1213823	up	protein_coding	NM_006522	WNT6
ASHGV40007199	0.016327108	0.126020119	2.0711996	up	protein_coding	NM_020404	CD248
ASHGV40047613	0.03744129	0.186308886	2.339971	up	protein_coding	NM_031414	STK31
ASHGV40026827	0.002759363	0.061986011	3.6397452	up	protein_coding	NM_001309193	EPHB2
ASHGV40038473	0.00866635	0.093650627	2.428998	up	protein_coding	NM_006342	TACC3
ASHGV40018424	0.006251947	0.081506727	2.4991692	up	protein_coding	uc002fle.1	BC033739
ASHGV40049885	2.33016E-05	0.025539914	106.893981	up	protein_coding	NM_004306	ANXA13
ASHGV40011369	0.009577939	0.0980628	3.9950581	up	protein_coding	NM_005276	GPD1
ASHGV40033857	0.011168251	0.104993631	2.9298077	up	protein_coding	NM_016426	GTSE1
ASHGV40017121	0.045227796	0.205952428	3.3658792	up	protein_coding	NM_000745	CHRNA5
ASHGV40053271	0.024905084	0.152588216	2.7007254	up	protein_coding	NM_021224	ZNF462
ASHGV40002036	0.029922636	0.166580805	2.09059	up	protein_coding	NM_032704	TUBA1C
ASHGV40020278	0.03913644	0.191250833	3.6390516	up	protein_coding	NM_001067	TOP2A
ASHGV40014644	0.038385124	0.189180431	2.5156014	up	protein_coding	NM_138420	AHNAK2
ASHGV40005769	0.014162632	0.117255292	2.5780647	up	protein_coding	NM_170744	UNC5B
ASHGV40033097	0.016397212	0.126248248	2.501323	up	protein_coding	NM_030965	ST6GALNAC5
ASHGV40011078	0.000132766	0.029433159	3.2628742	up	protein_coding	NM_003979	GPRC5A
ASHGV40014054	0.017026588	0.128497186	2.0559008	up	protein_coding	NM_002863	PYGL
ASHGV40040973	0.00443928	0.071471184	2.8691805	up	protein_coding	NM_206966	C5orf46
ASHGV40016736	0.045641815	0.207080587	2.5773671	up	protein_coding	NM_138423	CASC4
ASHGV40029528	0.015264937	0.122135704	2.3564105	up	protein_coding	NM_013310	C2orf27A
ASHGV40008150	0.020972947	0.143379212	2.9462452	up	protein_coding	NM_001202439	NCR3LG1
ASHGV40050997	0.003182167	0.063576796	6.5160705	up	protein_coding	NM_001142462	OSR2
ASHGV40003145	0.02030976	0.140635815	4.2199869	up	protein_coding	NM_001195597	SMLR1
ASHGV40031070	0.001692792	0.055003756	3.4008059	up	protein_coding	NM_020182	PMEPA1
ASHGV40012361	0.007581968	0.088015441	3.7714352	up	protein_coding	NM_207418	FAM72D
ASHGV40010418	0.030469939	0.168313289	2.3674259	up	protein_coding	NM_018351	FGD6
ASHGV40007240	0.007519548	0.087872873	2.1086517	up	protein_coding	ENST0000022747	UNC93B1
ASHGV40046951	0.021446946	0.144717084	15.5678555	up	protein_coding	NM_000111	SLC26A3
ASHGV40027011	0.001069302	0.049811075	9.6692468	up	protein_coding	NM_001277053	TRABD2A
ASHGV40015391	0.00106715	0.049811075	2.635752	up	protein_coding	NM_000710	BDKRB1
ASHGV40046723	0.000819705	0.048785893	2.6727588	up	protein_coding	NM_000601	HGF
ASHGV40013770	0.009522683	0.097841781	2.686787	up	protein_coding	NM_032132	HORMAD1
ASHGV40026046	0.005273553	0.076180338	5.6133185	up	protein_coding	NM_002288	LAIR2
ASHGV40015004	0.013050518	0.112592198	4.1252255	up	protein_coding	NM_005192	CDKN3
ASHGV40010563	0.004784199	0.073532246	9.3428483	up	protein_coding	NM_001854	COL11A1
ASHGV40011542	0.013902257	0.116482338	2.2150997	up	protein_coding	NM_002332	LRP1
ASHGV40010554	0.012757861	0.111313165	2.7639598	up	protein_coding	NM_014325	CORO1C
ASHGV40013323	0.000819217	0.048785893	2.3811492	up	protein_coding	NM_138450	ARL11

ASHGV40049968	0.008295694	0.091662687	3.7984237	up	protein_coding	NM_004519	KCNQ3
ASHGV40006524	0.000237255	0.03332801	5.1840405	up	protein_coding	NM_003311	PHLDA2
ASHGV40021322	0.01301827	0.112530032	2.1928583	up	protein_coding	NM_020808	SIPA1L2
ASHGV40029917	0.001766473	0.055403878	2.9430495	up	protein_coding	NM_001031716	NABP1
ASHGV40029886	0.0093189	0.09741267	2.3638175	up	protein_coding	NM_000090	COL3A1
ASHGV40023679	0.026577663	0.157860108	2.3576909	up	protein_coding	NM_213604	ADAMTSL5
ASHGV40011054	0.017277125	0.129292086	3.3147226	up	protein_coding	NM_004696	SLC16A4
ASHGV40028642	0.007579349	0.088015441	3.7451005	up	protein_coding	NM_000221	KHK
ASHGV40041640	0.004962123	0.074201092	2.1624666	up	protein_coding	NM_003999	OSMR
ASHGV40048363	0.010492325	0.102456293	4.3804425	up	protein_coding	NM_000492	CFTR
ASHGV40041227	0.000381693	0.039736903	9.6993681	up	protein_coding	NM_000505	F12
ASHGV40007343	0.009799863	0.099240515	2.9338773	up	protein_coding	NM_182904	P4HA3
ASHGV40039545	0.026381558	0.15734077	2.2000212	up	protein_coding	NM_000856	GUCY1A3
ASHGV40016215	0.001375976	0.052512256	2.2268032	up	protein_coding	NM_001100879	ST20
ASHGV40039143	0.000759065	0.047440361	2.4510638	up	protein_coding	NM_000297	PKD2
ASHGV40045681	0.008737198	0.093806518	2.3865712	up	protein_coding	uc003qdy.1	SNORA33
ASHGV40047489	0.001660326	0.054739049	2.5235463	up	protein_coding	NM_003088	FSCN1
ASHGV40048670	1.72179E-05	0.025539914	2.8461997	up	protein_coding	NM_018487	TMEM176A
ASHGV40021913	0.001187202	0.050120601	3.101608	up	protein_coding	NM_002204	ITGA3
ASHGV40027635	0.002053449	0.055772948	5.7155998	up	protein_coding	NM_001935	DPP4
ASHGV40026972	0.004717271	0.07324162	3.1046344	up	protein_coding	NM_032181	EVA1A
ASHGV40002682	0.010440089	0.102226677	4.3513048	up	protein_coding	ENST0000059999	AC187652.1
ASHGV40042599	0.039863959	0.193289207	2.057777	up	protein_coding	NM_001099293	KIF4B
ASHGV40019247	4.61553E-05	0.028718686	2.7860261	up	protein_coding	NM_001178020	BEAN1
ASHGV40030138	0.026343838	0.157197522	2.4026084	up	protein_coding	NM_000784	CYP27A1
ASHGV40029476	0.003550039	0.06563995	8.361562	up	protein_coding	NM_001080527	MYO7B
ASHGV40043173	0.004084664	0.069284044	2.9891536	up	protein_coding	NM_003546	HIST1H4L
ASHGV40027905	0.018415949	0.133680469	2.6138201	up	protein_coding	uc002uvb.1	AK025127
ASHGV40001318	0.001158103	0.050016538	4.104148	up	protein_coding	NM_002983	CCL3
ASHGV40000130	0.000203469	0.031004988	4.3469075	up	protein_coding	NM_002966	S100A10
ASHGV40018763	0.035702255	0.181859659	2.6742383	up	protein_coding	NM_024847	TMC7
ASHGV40015124	0.002550463	0.06066831	2.4331641	up	protein_coding	NM_004494	HDGF
ASHGV40056480	0.005856216	0.079836199	2.693307	up	protein_coding	NM_001100910	FAM72B
ASHGV40018114	0.019273939	0.136825495	3.6798707	up	protein_coding	NM_003039	SLC2A5
ASHGV40031257	0.028004567	0.161495561	2.9666188	up	protein_coding	NM_021873	CDC25B
ASHGV40037828	0.022481914	0.147541072	3.1768893	up	protein_coding	NM_001813	CENPE
ASHGV40019011	0.010258518	0.101132463	2.5311659	up	protein_coding	NM_014321	ORC6
ASHGV40023112	0.000640694	0.045710101	3.0058205	up	protein_coding	NM_015210	MTCL1
ASHGV40013504	0.007175088	0.08574697	3.8718017	up	protein_coding	ENST0000036917	FAM72C
ASHGV40042924	0.048834153	0.213071006	2.2023496	up	protein_coding	NM_004155	SERPINB9
ASHGV40005374	0.007051539	0.085147057	3.9578486	up	protein_coding	NM_001142308	MALRD1
ASHGV40040309	0.001615165	0.054621802	2.939822	up	protein_coding	NM_197941	ADAMTS6
ASHGV40007751	0.031619028	0.171533717	2.1248676	up	protein_coding	NM_001098169	BSX
ASHGV40014100	0.018620783	0.134412027	3.1828012	up	protein_coding	NM_014750	DLGAP5
ASHGV40015992	0.042159562	0.199320534	2.5016057	up	protein_coding	NM_014736	KIAA0101
ASHGV40039923	0.030459305	0.168313289	2.0816668	up	protein_coding	NM_003966	SEMA5A
ASHGV40039369	0.000211431	0.031004988	4.5369862	up	protein_coding	NM_014278	HSPA4L
ASHGV40050156	0.01602221	0.124882995	2.7470698	up	protein_coding	NM_000417	IL2RA
ASHGV40048855	0.026427938	0.157433474	2.389243	up	protein_coding	NM_001256872	USP17L8
ASHGV40009281	0.02107402	0.143756832	2.6629879	up	protein_coding	NM_032873	UBASH3B
ASHGV40007541	0.000893821	0.049204994	18.0665772	up	protein_coding	NM_002422	MMP3
ASHGV40002432	0.03821362	0.188644584	2.0236022	up	protein_coding	NM_006470	TRIM16
ASHGV40023054	0.03135664	0.170587948	3.0222224	up	protein_coding	NM_006101	NDC80
ASHGV40028028	0.002777743	0.061986011	3.0059081	up	protein_coding	NM_054034	FN1
ASHGV40031595	0.024340372	0.151674021	4.2840615	up	protein_coding	NM_030919	FAM83D
ASHGV40000097	0.017499024	0.12972491	2.5294061	up	protein_coding	ENST0000034462	SULT1A4
ASHGV40008704	0.037427576	0.186308886	3.834137	up	protein_coding	NM_001136485	C11orf86
ASHGV40003251	0.023431286	0.149727141	2.2030428	up	protein_coding	NM_002984	CCL4
ASHGV40007630	0.025745317	0.155491458	2.4460722	up	protein_coding	NM_001562	IL18
ASHGV40003231	0.028990453	0.164383305	2.690877	up	protein_coding	NM_001304433	LOC101928841
ASHGV40011420	0.027551709	0.160592595	2.0530372	up	protein_coding	NM_000224	KRT18

ASHGV40020835	0.006733414	0.083835464	2.9658529	up	protein_coding	NM_139018	CD300LF
ASHGV40030774	0.000588969	0.044687591	2.4394225	up	protein_coding	NM_080476	PIGU
ASHGV40048014	0.019266645	0.136825495	2.2867324	up	protein_coding	ENST0000036182	GATSL1
ASHGV40018611	0.023033887	0.148637619	2.1178957	up	protein_coding	NM_024339	THOC6
ASHGV40024805	0.003462973	0.065065084	3.4269865	up	protein_coding	NM_003283	TNNT1
ASHGV40031447	0.030881319	0.169376119	2.0761214	up	protein_coding	NM_021067	GINS1
ASHGV40033636	0.002147222	0.056552005	10.1728216	up	protein_coding	NM_000343	SLC5A1
ASHGV40024998	0.027500181	0.160419258	3.1781162	up	protein_coding	NM_003249	THOP1
ASHGV40007573	0.007956379	0.090141279	3.1765068	up	protein_coding	NM_000855	GUCY1A2
ASHGV40025098	0.025340467	0.154326364	2.6182499	up	protein_coding	NM_198534	C19orf45
ASHGV40020504	0.001244017	0.05083886	3.3796907	up	protein_coding	NM_018952	HOXB6
ASHGV40024356	0.048949186	0.213316173	3.746035	up	protein_coding	NM_002307	LGALS7
ASHGV40007727	9.84426E-05	0.028718686	4.7344238	up	protein_coding	NM_006288	THY1
ASHGV40021541	0.035526681	0.181386509	2.2172077	up	protein_coding	NM_024857	ATAD5
ASHGV40007540	0.009379833	0.097468969	3.8180373	up	protein_coding	NM_002421	MMP1
ASHGV40006208	0.016415623	0.126311915	2.1681777	up	protein_coding	NM_203379	ACSL5
ASHGV40035492	8.46835E-05	0.028718686	2.0088354	up	protein_coding	NM_005017	PCYT1A
ASHGV40006788	0.047249372	0.209949045	2.7621111	up	protein_coding	NM_031217	KIF18A
ASHGV40042782	0.015662566	0.123530497	2.7042574	up	protein_coding	NM_017675	CDHR2
ASHGV40024703	0.000566084	0.044261189	3.2639406	up	protein_coding	NM_053003	SIGLEC12
ASHGV40051365	0.004357264	0.071084094	4.6568084	up	protein_coding	ENST0000039517	C8orf31
ASHGV40057751	0.010512406	0.102456293	2.3404595	up	protein_coding	NM_005566	LDHA
ASHGV40030271	0.021113252	0.143756832	2.2442744	up	protein_coding	NM_206895	C2orf82
ASHGV40028711	0.016853484	0.12782262	4.2099443	up	protein_coding	NM_153212	GJB4
ASHGV40007951	0.039470265	0.192343079	2.2958946	up	protein_coding	NM_001290332	SYT8
ASHGV40030287	0.046962465	0.209283925	2.0465538	up	protein_coding	NM_001145636	C1orf228
ASHGV40037633	0.027017175	0.159100475	2.0459206	up	protein_coding	NM_002090	CXCL3
ASHGV40051852	0.006929467	0.084906668	3.7315274	up	protein_coding	NM_017662	TRPM6
ASHGV40044922	0.008596885	0.093276474	3.781963	up	protein_coding	NM_002263	KIFC1
ASHGV40011020	0.007024109	0.085147057	2.2690446	up	protein_coding	NM_080387	CLEC4D
ASHGV40051706	3.57831E-05	0.026561918	2.432843	up	protein_coding	NM_003289	TPM2
ASHGV40024932	0.000598751	0.044791154	4.0189127	up	protein_coding	NM_173481	MISP
ASHGV40034311	0.00360106	0.066209231	4.4723255	up	protein_coding	NM_000094	COL7A1
ASHGV40046512	0.012572563	0.11054147	2.0645449	up	protein_coding	NM_022116	FIGNL1
ASHGV40046840	0.007705416	0.088976529	9.2684273	up	protein_coding	ENST0000033637	CYP3A7
ASHGV40048519	0.011864223	0.108471894	2.2101215	up	protein_coding	ENST0000041650	AC015987.2
ASHGV40042270	0.022687218	0.147893728	2.8343115	up	protein_coding	NM_005573	LMNB1
ASHGV40019615	0.00484626	0.073566912	14.4866591	up	protein_coding	NM_004413	DPEP1
ASHGV40029326	0.000499505	0.042538419	4.0192629	up	protein_coding	NM_001136493	MFSD2A
ASHGV40036058	0.017081181	0.128595629	2.0262552	up	protein_coding	NM_002841	PTPRG
ASHGV40043168	0.003089042	0.062854503	3.9411122	up	protein_coding	NM_021066	HIST1H2AJ
ASHGV40018224	0.000179926	0.030140601	4.327261	up	protein_coding	NM_015020	PHLPP2
ASHGV40034072	0.012364885	0.109722298	2.531652	up	protein_coding	NM_001012410	SGOL1
ASHGV40018483	0.001803163	0.05564593	2.8493733	up	protein_coding	NM_014176	UBE2T
ASHGV40034360	0.001249473	0.05083886	2.361953	up	protein_coding	NM_005879	TRAIP
ASHGV40050612	0.042511057	0.20004572	2.0509896	up	protein_coding	NM_005914	MCM4
ASHGV40037597	0.001262491	0.05083886	6.1460703	up	protein_coding	NM_001073	UGT2B11
ASHGV40046418	2.37625E-05	0.025539914	30.0573289	up	protein_coding	NM_002192	INHBA
ASHGV40042544	0.015880099	0.124180314	2.4051385	up	protein_coding	NM_152406	AFAP1L1
ASHGV40014424	0.016063213	0.125034905	5.2862493	up	protein_coding	NM_005978	S100A2
ASHGV40047302	0.001156942	0.050016538	2.256849	up	protein_coding	NM_014020	TMEM176B
ASHGV40051368	0.0464956	0.209075944	2.2118839	up	protein_coding	NM_001301772	GPIHBP1
ASHGV40022370	0.036409874	0.183586631	2.5647438	up	protein_coding	ENST0000059822	AC132872.2
ASHGV40038815	0.027864298	0.161155167	3.0354262	up	protein_coding	NM_004181	UCHL1
ASHGV40055038	0.001415247	0.052512256	7.9437581	up	protein_coding	NM_014799	HEPH
ASHGV40035351	0.011539655	0.106699757	2.5095271	up	protein_coding	NM_004366	CLCN2
ASHGV40003365	0.01025728	0.101132463	2.7669172	up	protein_coding	NM_207007	CCL4L1
ASHGV40000244	0.002602809	0.061245041	3.4866884	up	protein_coding	ENST0000040368	HIST2H3A
ASHGV40047502	0.03106188	0.169938797	2.0133974	up	protein_coding	NM_017560	ZNF853
ASHGV40039049	0.004605769	0.072543977	8.345225	up	protein_coding	ENST0000040193	IL8
ASHGV40029646	0.042157429	0.199320534	2.4975771	up	protein_coding	NM_007115	TNFAIP6

ASHGV40046251	0.00173513	0.055333849	11.2115934	up	protein_coding	NM_006547	IGF2BP3
ASHGV40006076	0.018605123	0.134382322	3.2977677	up	protein_coding	ENST0000037044	ABCC2
ASHGV40033194	0.015776354	0.123828175	2.1061933	up	protein_coding	NM_145174	DNAJB7
ASHGV40005692	0.013830063	0.116063883	2.8638017	up	protein_coding	NM_001786	CDK1
ASHGV40056001	0.003285056	0.064600323	2.1640903	up	protein_coding	uc001txn.1	AK130486
ASHGV40050440	0.003017766	0.062622161	2.0530616	up	protein_coding	NM_001440	EXTL3
ASHGV40030903	0.00412654	0.069649315	4.8061328	up	protein_coding	NM_003279	TNNC2
ASHGV40050126	0.001137716	0.050016538	3.119263	up	protein_coding	NM_012079	DGAT1
ASHGV40019488	0.00037583	0.039736903	2.7781469	up	protein_coding	NM_001257	CDH13
ASHGV40006002	0.00615575	0.081250308	2.8448777	up	protein_coding	NM_000783	CYP26A1
ASHGV40033353	0.02756977	0.160592595	2.3742086	up	protein_coding	NM_001953	TYMP
ASHGV40007866	0.027461316	0.160353487	2.2569595	up	protein_coding	NM_001277285	IGSF9B
ASHGV40028314	0.011621076	0.107039132	2.6635048	up	protein_coding	ENST0000039136	AL136115.1
ASHGV40020379	0.013977683	0.11674283	2.3718695	up	protein_coding	NM_001142653	PTGES3L
ASHGV40035157	0.002560983	0.060670246	7.7746873	up	protein_coding	NM_206963	RARRES1
ASHGV40020082	0.006018809	0.080593472	2.5461426	up	protein_coding	NM_031934	RAB34
ASHGV40043468	0.000853293	0.048843614	6.9072255	up	protein_coding	NM_018643	TREM1
ASHGV40015749	0.006009721	0.080593472	9.2780121	up	protein_coding	NM_017726	PPP1R14D
ASHGV40026135	0.01258695	0.11054147	2.3710264	up	protein_coding	NM_012387	PADI4
ASHGV40012549	0.006947857	0.085006048	3.6510267	up	protein_coding	NM_025113	KIAA0226L
ASHGV40046146	0.019246332	0.136825495	3.6436858	up	protein_coding	NM_016343	CENPF
ASHGV40053863	0.029386615	0.165386939	3.703409	up	protein_coding	NM_138932	A1CF
ASHGV40020209	0.001813346	0.05576279	2.3263597	up	protein_coding	uc010cum.2	DQ580766
ASHGV40020327	0.044064308	0.204031307	2.3329594	up	protein_coding	NM_002276	KRT19
ASHGV40024936	0.001881463	0.055772948	2.743941	up	protein_coding	NM_025106	SPSB1
ASHGV40050139	0.012904618	0.112043699	2.2952568	up	protein_coding	ENST0000042855	RECQL4
ASHGV40057085	0.000341156	0.037940966	2.1128438	up	protein_coding	NM_138348	OTULIN
ASHGV40005329	0.014082159	0.11697786	4.5676179	up	protein_coding	NM_182751	MCM10
ASHGV40044907	0.040358733	0.194468865	2.1348904	up	protein_coding	NM_002800	PSMB9
ASHGV40014484	0.017271758	0.129292086	3.413227	up	protein_coding	NM_000295	SERPINA1
ASHGV40053787	0.02003398	0.139613302	2.0996811	up	protein_coding	NM_152633	FANCB
ASHGV40045470	0.038910941	0.190790541	2.4772509	up	protein_coding	NM_001624	AIM1
ASHGV40003192	0.020012166	0.139517045	2.5453724	up	protein_coding	NM_001243523	LOC100130880
ASHGV40014372	0.035093821	0.180316786	2.9372577	up	protein_coding	NM_001045479	S100A7L2
ASHGV40053088	0.002535314	0.060555796	4.1744337	up	protein_coding	ENST0000031435	CKS2
ASHGV40019832	0.007491063	0.087778347	3.5238262	up	protein_coding	NM_001146684	RNF222
ASHGV40054624	0.024610194	0.152212021	3.5771992	up	protein_coding	NM_175569	XG
ASHGV40000815	0.006934527	0.084908993	3.9242181	up	protein_coding	ENST0000043583	CEA
ASHGV40052816	0.001265432	0.05083886	3.0688186	up	protein_coding	NM_005893	CCIN
ASHGV40009578	0.045687297	0.207147534	2.0955998	up	protein_coding	NM_016511	CLEC1A
ASHGV40054757	0.002975142	0.06249329	2.3864382	up	protein_coding	NM_032797	AIFM2
ASHGV40033785	0.024658751	0.152291693	2.3897763	up	protein_coding	NM_024821	CCDC134
ASHGV40051808	0.006551319	0.083034206	2.0219956	up	protein_coding	NM_199244	FOXD4L4
ASHGV40034833	0.002746223	0.061986011	8.4916818	up	protein_coding	NM_033049	MUC13
ASHGV40032853	0.000693851	0.046533174	61.7284239	up	protein_coding	NM_000300	PLA2G2A
ASHGV40037947	0.031643446	0.171559428	2.8203233	up	protein_coding	NM_001237	CCNA2
ASHGV40033936	0.021574821	0.145208242	2.5099503	up	protein_coding	NM_003503	CDC7
ASHGV40038077	0.044243537	0.204236449	2.7292019	up	protein_coding	NM_003866	INPP4B
ASHGV40021852	0.012175926	0.109304951	2.2094548	up	protein_coding	NM_000212	ITGB3
ASHGV40044229	0.000109606	0.028718686	2.8800406	up	protein_coding	NM_207360	ZC3H12D
ASHGV40050356	0.033146854	0.175924118	2.0962141	up	protein_coding	NM_001722	POLR3D
ASHGV40008284	0.021441497	0.144717084	2.5191216	up	protein_coding	NM_000610	CD44
ASHGV40021896	0.035163847	0.180487733	2.089228	up	protein_coding	NM_006546	IGF2BP1
ASHGV40026047	0.000864462	0.048985823	2.0959363	up	protein_coding	uc010erm.2	KIR3DX1
ASHGV40021362	0.00564571	0.078517713	2.276484	up	protein_coding	NM_016113	TRPV2
ASHGV40035923	0.01245148	0.110037506	3.039256	up	protein_coding	NM_004345	CAMP
ASHGV40032186	0.047730064	0.211170109	2.5779072	up	protein_coding	NM_144659	TCP10L
ASHGV40052641	0.001034414	0.049811075	4.5429125	up	protein_coding	NM_000550	TYRP1
ASHGV40024281	0.020604096	0.141885078	6.9180966	up	protein_coding	NM_001166034	SBSN
ASHGV40046328	0.001082888	0.049811075	2.9630654	up	protein_coding	NM_003238	TGFB2
ASHGV40012046	0.031909665	0.172378528	2.0894594	up	protein_coding	ENST0000054794	RP11-116D17.1

ASHGV40046881	0.01612329	0.125111565	3.1100757	up	protein_coding	NM_178176	MOGAT3
ASHGV40054545	0.007832158	0.089754791	3.3763735	up	protein_coding	NM_004961	GABRE
ASHGV40025798	0.000594604	0.044687591	11.7158467	up	protein_coding	NM_000483	APOC2
ASHGV40035238	0.031215806	0.170193495	2.6953164	up	protein_coding	NM_015028	TNIK
ASHGV40040722	0.000352497	0.038174731	2.4049393	up	protein_coding	NM_002317	LOX
ASHGV40051588	0.032273272	0.173303593	3.9273111	up	protein_coding	NM_000077	CDKN2A
ASHGV40054100	0.007432831	0.087448614	2.0148606	up	protein_coding	NM_002547	OPHN1
ASHGV40017870	0.006507155	0.08287711	2.1878207	up	protein_coding	NM_013258	PYCARD
ASHGV40019136	0.001552598	0.053863419	2.3113279	up	protein_coding	NM_004530	MMP2
ASHGV40024700	0.034009184	0.177949164	2.3964564	up	protein_coding	NM_033130	SIGLEC10
ASHGV40021950	0.001888712	0.055772948	2.4159796	up	protein_coding	NM_000269	NME1
ASHGV40009518	0.011971154	0.108703513	3.6125276	up	protein_coding	NM_031299	CDCA3
ASHGV40008815	0.008716134	0.093650627	2.2435188	up	protein_coding	ENST0000044294	FOLR3
ASHGV40048593	0.016651327	0.127450629	2.8043821	up	protein_coding	NM_003461	ZYX
ASHGV40033603	0.002407751	0.059482588	2.4443272	up	protein_coding	NM_012429	SEC14L2
ASHGV40023619	0.04761189	0.210861292	2.1317601	up	protein_coding	NM_171999	SALL3
ASHGV40015503	0.011143054	0.104965039	3.2953462	up	protein_coding	NM_030943	AMN
ASHGV40005068	0.017117743	0.128648692	2.4948081	up	protein_coding	NM_003474	ADAM12
ASHGV40024293	0.002080321	0.055772948	2.21564	up	protein_coding	NM_003332	TYROBP
ASHGV40011461	0.007412556	0.087327927	20.1907965	up	protein_coding	NM_006897	HOXC9
ASHGV40008863	0.005607347	0.07834111	2.458821	up	protein_coding	NM_001195528	TPBGL
ASHGV40030000	0.015942164	0.124593263	2.2093069	up	protein_coding	NM_006139	CD28
ASHGV40006126	0.000629684	0.045463199	2.3034897	up	protein_coding	NM_001077494	NFKB2
ASHGV40039849	0.009997645	0.099667779	2.4439014	up	protein_coding	NM_002529	NTRK1
ASHGV40041642	0.000586535	0.044687591	5.6696049	up	protein_coding	NM_022716	PRRX1
ASHGV40046842	0.015356552	0.122207594	5.8771758	up	protein_coding	NM_017460	CYP3A4
ASHGV40008253	0.024765262	0.15232004	2.6273873	up	protein_coding	NM_002901	RCN1
ASHGV40026987	0.023987073	0.150731954	10.2668271	up	protein_coding	NM_006507	REG1B
ASHGV40000010	0.033664085	0.177029899	2.0566802	up	protein_coding	NM_001164257	PRR29
ASHGV40007145	0.011099665	0.104667783	3.0457699	up	protein_coding	uc009ypx.3	AB429224
ASHGV40054037	0.005185202	0.075909447	2.126489	up	protein_coding	NM_004493	HSD17B10
ASHGV40007225	0.033565417	0.176836053	3.163303	up	protein_coding	NM_004153	ORC1
ASHGV40032144	0.040931837	0.196175787	2.1645628	up	protein_coding	NM_012131	CLDN17
ASHGV40056369	0.001447407	0.052761522	20.2742573	up	protein_coding	ENST0000035405	CCL15
ASHGV40052821	0.049075139	0.213699077	3.4346035	up	protein_coding	NM_014791	MELK
ASHGV40012107	6.62815E-05	0.028718686	120.0832518	up	protein_coding	NM_032044	REG4
ASHGV40027050	0.000256232	0.033591455	207.8181343	up	protein_coding	NM_001443	FABP1
ASHGV40028498	0.000729523	0.046846037	7.0223344	up	protein_coding	NM_001039362	ATP6V1C2
ASHGV40003178	0.001110771	0.049811075	2.8579416	up	protein_coding	NM_005118	TNFSF15
ASHGV40005964	0.014357161	0.118025204	2.4662213	up	protein_coding	NM_001010987	IFIT1B
ASHGV40048096	0.010831391	0.10371012	2.0912807	up	protein_coding	NM_021723	ADAM22
ASHGV40039915	0.000611396	0.044791154	10.9419144	up	protein_coding	NM_152372	MYOM3
ASHGV40024451	0.00729647	0.08648623	3.9210606	up	protein_coding	NM_001184825	PSG1
ASHGV40044778	0.003308888	0.064680656	2.8342013	up	protein_coding	NM_003536	HIST1H3H
ASHGV40050122	0.007140431	0.085567393	2.0251726	up	protein_coding	ENST0000037741	KM-PA-2
ASHGV40017638	0.000147641	0.029433159	2.6101281	up	protein_coding	NM_018340	CPPED1
ASHGV40045117	0.000852613	0.048843614	18.0217764	up	protein_coding	NM_005588	MEP1A
ASHGV40010837	0.04479037	0.205292231	2.4154968	up	protein_coding	NM_144584	HENMT1
ASHGV40017681	0.001152133	0.050016538	6.8766982	up	protein_coding	NM_000963	PTGS2
ASHGV40029399	0.009989424	0.099667779	4.1307933	up	protein_coding	NM_006770	MARCO
ASHGV40054248	0.002929065	0.061986011	3.7625493	up	protein_coding	NM_178505	TMEM26
ASHGV40020500	0.000986714	0.049788318	2.5442465	up	protein_coding	NM_002146	HOXB3
ASHGV40033601	0.004456269	0.071546504	4.4997184	up	protein_coding	uc003aha.3	MGC20647
ASHGV40056018	0.013356107	0.113987801	2.8534222	up	protein_coding	NM_004004	GJB2
ASHGV40044945	0.02967293	0.166305754	2.4321625	up	protein_coding	NM_145899	HMGA1
ASHGV40036158	0.033742126	0.177367414	2.1102149	up	protein_coding	NM_002942	ROBO2
ASHGV40025875	0.031415176	0.170640188	2.5223717	up	protein_coding	NM_133498	SPACA4
ASHGV40007583	0.030908286	0.169417437	2.8084774	up	protein_coding	NM_003063	SLN
ASHGV40007677	0.000666653	0.046153893	242.5544689	up	protein_coding	NM_000482	APOA4
ASHGV40016877	0.03208576	0.172893897	3.1044913	up	protein_coding	NM_004701	CCNB2
ASHGV40008636	0.008450719	0.092498753	2.1689706	up	protein_coding	NM_006779	CDC42EP2

ASHGV40031688	0.002091793	0.055911749	3.5257629	up	protein_coding	NM_007019	UBE2C
ASHGV40021702	0.024298884	0.151674021	2.6561948	up	protein_coding	NM_001254	CDC6
ASHGV40053649	0.000896924	0.049204994	2.3225462	up	protein_coding	NM_016215	EGFL7
ASHGV40034869	0.002844066	0.061986011	2.3894693	up	protein_coding	NM_003707	RUVBL1
ASHGV40017492	0.001936905	0.055772948	2.0004859	up	protein_coding	NM_001042371	PGP
ASHGV40019355	0.002002941	0.055772948	6.0303412	up	protein_coding	NM_052858	MARVELD3
ASHGV40040295	0.048170801	0.212214493	2.3900136	up	protein_coding	NM_021258	IL22RA1
ASHGV40031671	0.015811242	0.12390329	7.430119	up	protein_coding	NM_003007	SEMG1
ASHGV40041266	0.006310724	0.081992381	2.952043	up	protein_coding	NM_014244	ADAMTS2
ASHGV40003354	0.022915333	0.148477052	3.1593993	up	protein_coding	NM_181809	BMP8A
ASHGV40041328	0.033831903	0.177564383	2.9602724	up	protein_coding	NM_004237	TRIP13
ASHGV40051163	0.020391454	0.140986613	2.0192338	up	protein_coding	NM_014943	ZHX2
ASHGV40023997	0.031923048	0.172378528	2.3227591	up	protein_coding	NM_032571	ADGRE3
ASHGV40047997	0.000616279	0.044960019	11.3090999	up	protein_coding	NM_001305	CLDN4
ASHGV40044781	0.004431504	0.071459267	2.2512889	up	protein_coding	NM_003511	HIST1H2AL
ASHGV40014295	0.021856086	0.145952783	2.2317029	up	protein_coding	NM_005050	ABCD4
ASHGV40016652	0.001381082	0.052512256	4.8786579	up	protein_coding	NM_003246	THBS1
ASHGV40055549	0.002236653	0.058034571	3.0053889	up	protein_coding	NM_001711	BGN
ASHGV40017937	0.030851593	0.169319601	3.8974173	up	protein_coding	NM_024745	SHCBP1
ASHGV40007543	0.001337527	0.052312433	19.2277752	up	protein_coding	NM_002426	MMP12
ASHGV40056421	0.001109041	0.049811075	3.5887534	up	protein_coding	NM_003955	SOCS3
ASHGV40022099	0.002676712	0.061839224	2.351178	up	protein_coding	NM_014812	CEP170
ASHGV40045271	0.007136468	0.085567393	2.6458837	up	protein_coding	NM_133493	CD109
ASHGV40020511	0.00637399	0.082378819	2.2168956	up	protein_coding	NM_006361	HOXB13
ASHGV40030290	0.035530004	0.181386509	2.3873995	up	protein_coding	NM_006944	SPP2
ASHGV40060830	0.007705584	0.088976529	2.6270816	up	protein_coding	uc003afp.3	KIAA0845
ASHGV40037697	0.018445044	0.133835953	2.3326662	up	protein_coding	NM_001145794	ANTXR2
ASHGV40009161	0.002884436	0.061986011	3.590827	up	protein_coding	NM_006169	NNMT
ASHGV40023382	0.044142756	0.204031307	3.0937673	up	protein_coding	NM_145060	SKA1
ASHGV40045975	0.006964212	0.085010804	5.3563899	up	protein_coding	NM_018974	UNC93A
ASHGV40025481	0.019553974	0.138089546	4.0851806	up	protein_coding	NM_001238	CCNE1
ASHGV40032638	0.009421414	0.097468969	2.3958929	up	protein_coding	NM_001236	CBR3
ASHGV40000640	0.000550888	0.043740162	3.9963637	up	protein_coding	NM_001205019	GK
ASHGV40023978	0.001827041	0.055772948	4.0966097	up	protein_coding	NM_024825	PODNL1
ASHGV40027923	0.00475599	0.073457793	2.2128731	up	protein_coding	NM_001127391	ALS2CR12
ASHGV40015516	0.034721332	0.17943745	2.3896019	up	protein_coding	NM_152307	TRMT61A
ASHGV40015891	0.002693873	0.061839224	2.1248111	up	protein_coding	NM_000570	FCGR3B
ASHGV40036069	0.036847078	0.184616568	2.5504874	up	protein_coding	NM_000549	TSHB
ASHGV40057724	0.007748003	0.089215209	3.7678083	up	protein_coding	NM_145202	PRAP1
ASHGV40012867	0.006245731	0.081506727	5.0981381	up	protein_coding	NM_005073	SLC15A1
ASHGV40008671	0.00495179	0.074174755	7.0296438	up	protein_coding	NM_001323	CST6
ASHGV40017434	0.029741923	0.166478388	2.6751248	up	protein_coding	NM_000433	NCF2
ASHGV40057194	0.000338032	0.037940966	2.7011024	up	protein_coding	NM_003519	HIST1H2BL
ASHGV40010723	0.024676225	0.152291693	4.2300076	up	protein_coding	NM_006018	HCAR3
ASHGV40024349	0.043191887	0.201902251	2.0356897	up	protein_coding	NM_170604	RASGRP4
ASHGV40048839	0.017497358	0.12972491	26.7361819	up	protein_coding	NM_001926	DEFA6
ASHGV40034265	0.009005826	0.095630687	7.478465	up	protein_coding	NM_020208	SLC6A20
ASHGV40020545	0.000149618	0.029433159	3.9704169	up	protein_coding	NM_000088	COL1A1
ASHGV40036434	0.03704923	0.185191074	2.4062144	up	protein_coding	NM_006889	CD86
ASHGV40013252	0.021753067	0.145767282	2.1643536	up	protein_coding	uc001uyq.2	AK054970
ASHGV40032731	0.005369284	0.076791441	2.7228427	up	protein_coding	NM_003683	RRP1
ASHGV40008441	0.036147838	0.182900088	2.2706906	up	protein_coding	NM_001005210	LRRC55
ASHGV40010939	0.02040085	0.140986613	5.0110962	up	protein_coding	NM_017417	GALNT8
ASHGV40026044	0.024086307	0.150987108	2.0636418	up	protein_coding	NM_004431	EPHA2
ASHGV40028276	0.006007077	0.080593472	2.3398046	up	protein_coding	NM_018645	HES6
ASHGV40042866	0.043647578	0.202997913	2.5340355	up	protein_coding	NM_001040462	BTNL8
ASHGV40036008	0.0004917	0.042232917	4.3090453	up	protein_coding	NM_002217	ITIH3
ASHGV40031955	0.029820049	0.166580805	2.3024827	up	protein_coding	uc002yfy.3	TEL1-TNFRSF6
ASHGV40047606	0.006480736	0.082800205	3.499607	up	protein_coding	NM_001005340	GPNMB
ASHGV40037876	0.011928783	0.108520715	8.4579459	up	protein_coding	NM_153426	PITX2
ASHGV40050787	0.001760271	0.055403878	5.733197	up	protein_coding	NM_015170	SULF1

ASHGV40052607	0.042627862	0.200285477	2.3776736	up	protein_coding	NM_002195	INSL4
ASHGV40017045	0.000553961	0.043740162	2.5694378	up	protein_coding	NM_005576	LOXL1
ASHGV40038099	0.008026343	0.090230383	2.9374115	up	protein_coding	NM_004425	ECM1
ASHGV40036607	0.004684277	0.072989317	3.5091793	up	protein_coding	NM_001679	ATP1B3
ASHGV40020938	0.036861277	0.18463465	2.068427	up	protein_coding	NM_033445	HIST3H2A
ASHGV40028133	0.00289313	0.061986011	3.2405669	up	protein_coding	NM_006216	SERPINE2
ASHGV40007890	0.014945555	0.120643838	2.0335633	up	protein_coding	NM_025092	ATHL1
ASHGV40002653	0.020192407	0.140212987	2.3350965	up	protein_coding	ENST0000059795	TD-3214H19.1
ASHGV40049906	0.024855843	0.152493483	2.2803037	up	protein_coding	NM_006762	LAPTM5
ASHGV40003306	0.020130295	0.139962887	2.6986595	up	protein_coding	NM_024114	TRIM48
ASHGV40005432	0.021953366	0.146163095	2.2423416	up	protein_coding	NM_014317	PDSS1
ASHGV40040823	0.000104221	0.028718686	2.6873935	up	protein_coding	NM_201563	FCGR2C
ASHGV40042217	0.010625288	0.102908278	2.3832762	up	protein_coding	NM_182761	FAM170A
ASHGV40030063	0.0029226	0.061986011	11.6362311	up	protein_coding	NM_001875	CPS1
ASHGV40047158	0.011020526	0.104318076	2.0512872	up	protein_coding	NM_001018111	PODXL
ASHGV40011408	0.027865813	0.161155167	2.7835304	up	protein_coding	NM_005556	KRT7
ASHGV40043715	0.032051465	0.172804372	2.0199013	up	protein_coding	NM_015687	FILIP1
ASHGV40041054	0.018356285	0.13353737	3.2628446	up	protein_coding	NM_031423	NUF2
ASHGV40048148	0.001911566	0.055772948	2.1661604	up	protein_coding	NM_000089	COL1A2
ASHGV40026933	0.005753491	0.079177486	2.2898473	up	protein_coding	NM_144579	SFXN5
ASHGV40042905	0.004536159	0.071864614	4.5462306	up	protein_coding	NM_005562	LAMC2
ASHGV40007892	0.000134715	0.029433159	2.496189	up	protein_coding	NM_003641	IFITM1
ASHGV40015563	0.028008865	0.161495561	2.7824661	up	protein_coding	NM_001311	CRIP1
ASHGV40010384	0.002372348	0.059390042	2.301406	up	protein_coding	NM_002345	LUM
ASHGV40000177	0.04562337	0.207080587	2.8129881	up	protein_coding	NM_001256829	TMEM265
ASHGV40037790	0.000974113	0.049736021	19.2301107	up	protein_coding	NM_000670	ADH4
ASHGV40024579	0.004131712	0.069649315	2.1979892	up	protein_coding	NM_001301059	MEIS3
ASHGV40046786	0.001342937	0.052312433	5.3929315	up	protein_coding	NM_006528	TFPI2
ASHGV40030484	0.001190913	0.050120601	3.7399428	up	protein_coding	NM_000678	ADRA1D
ASHGV40005375	0.028098482	0.161785771	7.8061998	up	protein_coding	uc010qcq.1	C10orf112
ASHGV40047993	0.031968471	0.172465145	2.0309536	up	protein_coding	NM_017528	WBSCR22
ASHGV40014230	0.00225229	0.058034571	2.1732428	up	protein_coding	NM_001102	ACTN1
ASHGV40043016	0.002921343	0.061986011	4.8231069	up	protein_coding	NM_017770	ELOVL2
ASHGV40025270	0.001133362	0.050016538	5.635461	up	protein_coding	NM_024758	AGMAT
ASHGV40054336	0.003323118	0.064680656	6.9292193	up	protein_coding	NM_000640	IL13RA2
ASHGV40025035	0.031291124	0.170356921	2.2463389	up	protein_coding	NM_020209	SHD
ASHGV40012232	0.003535373	0.065507718	4.8709977	up	protein_coding	NM_004764	PIWIL1
ASHGV40013623	0.008596116	0.093276474	3.3964037	up	protein_coding	NM_001145862	MTMR11
ASHGV40045331	9.68612E-05	0.028718686	5.8319189	up	protein_coding	NM_000574	CD55
ASHGV40040829	0.000117785	0.028925399	2.8085754	up	protein_coding	NM_002653	PITX1
ASHGV40025083	0.00017364	0.030140601	2.2302373	up	protein_coding	NM_001288962	TRIP10
ASHGV40009183	0.017070137	0.12858334	4.5086299	up	protein_coding	NM_000040	APOC3
ASHGV40050113	0.015227581	0.121970651	2.1839957	up	protein_coding	NM_031308	EPPK1
ASHGV40031078	0.019676284	0.138497762	2.1560079	up	protein_coding	NM_153360	APCDD1L
ASHGV40034809	0.034409348	0.178986096	2.5367182	up	protein_coding	NM_199420	POLQ
ASHGV40030258	0.005415121	0.07701309	2.2477296	up	protein_coding	NM_152383	DIS3L2
ASHGV40056386	0.008066105	0.090502316	2.5547345	up	protein_coding	NM_000422	KRT17
ASHGV40024602	0.013555925	0.114725783	2.0165505	up	protein_coding	NM_001080434	LMTK3
ASHGV40046380	0.005133569	0.075534938	6.3069872	up	protein_coding	NM_003014	SFRP4
ASHGV40052960	0.044982222	0.205686042	2.1071739	up	protein_coding	NM_000700	ANXA1
ASHGV40052769	0.016804305	0.12782262	2.6359916	up	protein_coding	NM_002771	PRSS3
ASHGV40026672	0.00101298	0.049811075	29.970986	up	protein_coding	NM_022436	ABCG5
ASHGV40020386	0.000667181	0.046153893	6.4904762	up	protein_coding	NM_001986	ETV4
ASHGV40051664	0.001691244	0.055003756	2.0913367	up	protein_coding	NM_022917	NOL6
ASHGV40008129	0.018907499	0.135834839	2.3924872	up	protein_coding	NM_000922	PDE3B
ASHGV40054829	0.014373586	0.118104543	10.7964383	up	protein_coding	NM_000531	OTC
ASHGV40003052	0.001744106	0.055333849	19.5195101	up	protein_coding	NM_000584	CXCL8
ASHGV40039270	0.002995608	0.06255259	5.0654605	up	protein_coding	NM_001977	ENPEP
ASHGV40046049	0.017846369	0.131554	2.7221589	up	protein_coding	ENST0000051498	AC145676.2
ASHGV40023303	0.046012665	0.20757486	2.7182539	up	protein_coding	NM_017947	MOCOS
ASHGV40018994	0.003851319	0.068149113	3.1862346	up	protein_coding	uc010vgb.2	BC068290

ASHGV40027275	0.002334577	0.058995057	3.6846083	up	protein_coding	NM_152515	CKAP2L
ASHGV40033706	7.86203E-05	0.028718686	2.1358171	up	protein_coding	NM_002305	LGALS1
ASHGV40006453	0.000542021	0.043740162	2.0668539	up	protein_coding	NM_021034	IFITM3
ASHGV40044432	0.000873994	0.049097415	7.6675395	up	protein_coding	NM_003247	THBS2
ASHGV40046662	0.009901774	0.099393971	2.4222617	up	protein_coding	NM_148912	ABHD11
ASHGV40009523	0.005455194	0.07736975	2.2015543	up	protein_coding	NM_001733	C1R
ASHGV40007539	0.005207081	0.076039081	9.023434	up	protein_coding	NM_002425	MMP10
ASHGV40050141	0.001449459	0.052761522	2.7022691	up	protein_coding	NM_001308207	ARHGAP39
ASHGV40037121	0.003836834	0.0681253	2.1486205	up	protein_coding	NM_012445	SPON2
ASHGV40049829	0.001306265	0.051782513	9.3040793	up	protein_coding	NM_002546	TNFRSF11B
ASHGV40010886	0.007614413	0.088305167	2.4489306	up	protein_coding	NM_032636	PSRC1
ASHGV40020099	0.004966315	0.074201092	2.3593683	up	protein_coding	NM_007207	DUSP10
ASHGV40011965	0.033317188	0.176142659	2.1373543	up	protein_coding	NM_001917	DAO
ASHGV40053660	0.044049868	0.204031307	3.7782279	up	protein_coding	NM_000606	C8G
ASHGV40040286	0.018568168	0.134170978	4.1259954	up	protein_coding	NM_018369	DEPDC1B
ASHGV40035601	0.007908623	0.089971932	2.3826831	up	protein_coding	NM_001018115	FANCD2
ASHGV40024289	0.018783247	0.135176582	2.5224064	up	protein_coding	NM_021232	PRODH2
ASHGV40007189	0.031612822	0.171533717	2.503077	up	protein_coding	NM_053054	CATSPER1
ASHGV40010110	0.009764278	0.098982529	2.0090277	up	protein_coding	NM_014770	AGAP2
ASHGV40025167	0.016758582	0.127599404	3.0212911	up	protein_coding	NM_000201	ICAM1
ASHGV40025662	0.016203839	0.125579029	4.3828713	up	protein_coding	NM_001042507	LGALS7B
ASHGV40035344	0.00196993	0.055772948	2.4017148	up	protein_coding	NM_018622	PARL
ASHGV40005162	0.004782605	0.073532246	3.885718	up	protein_coding	NM_198472	FUOM
ASHGV40044737	0.009935605	0.099618864	2.9923047	up	protein_coding	NM_003522	HIST1H2BF
ASHGV40056073	0.016091856	0.125093583	2.2305715	up	protein_coding	NM_001145442	POTEM
ASHGV40025518	0.00369608	0.067298943	2.7411126	up	protein_coding	NM_004708	PDCD5
ASHGV40055098	0.004493035	0.071810531	9.8792745	up	protein_coding	NM_032562	PLA2G12B
ASHGV40014713	0.010935908	0.104057649	2.5202286	up	protein_coding	NM_001004715	OR4K17
ASHGV40041744	0.001337011	0.052312433	2.9920062	up	protein_coding	NM_006308	HSPB3
ASHGV40034059	0.022169885	0.146945582	2.3692773	up	protein_coding	NM_001351	DAZL
ASHGV40016942	0.029820298	0.166580805	3.5795356	up	protein_coding	NM_178859	SLC51B
ASHGV40007144	0.000248059	0.033353656	2.5605454	up	protein_coding	NM_006795	EHD1
ASHGV40043276	0.003382226	0.064680656	2.2092644	up	protein_coding	NM_013974	DDAH2
ASHGV40047450	0.010280129	0.101210803	2.3227403	up	protein_coding	NM_002452	NUDT1
ASHGV40054773	0.029228648	0.16492903	3.0800366	up	protein_coding	NM_005391	PDK3
ASHGV40007180	0.004904174	0.073905938	3.5382167	up	protein_coding	NM_005507	CFL1
ASHGV40040006	0.011910219	0.108498735	2.3930614	up	protein_coding	NM_018240	KIRREL
ASHGV40022457	0.004736036	0.073434545	2.4971375	up	protein_coding	ENST0000031786	HNRNPCL1
ASHGV40031639	0.002822228	0.061986011	3.8523201	up	protein_coding	NM_002466	MYBL2
ASHGV40039591	0.029009373	0.164383305	4.2621949	up	protein_coding	NM_001297550	APELA
ASHGV40044327	0.013498097	0.114359968	2.7263376	up	protein_coding	NM_000636	SOD2
ASHGV40044742	0.007051637	0.085147057	3.7898812	up	protein_coding	NM_003524	HIST1H2BH
ASHGV40044375	0.042920014	0.201116194	2.6734231	up	protein_coding	ENST0000054586	SDIM1
ASHGV40020849	0.032033587	0.172761403	2.9053506	up	protein_coding	NM_016185	HN1
ASHGV40027831	0.006251362	0.081506727	2.1097426	up	protein_coding	NM_000393	COL5A2
ASHGV40016579	0.021174906	0.143939828	2.7653615	up	protein_coding	NM_001039841	ARHGAP11B
ASHGV40055904	0.013049897	0.112592198	2.1300793	up	protein_coding	NM_016112	PKD2L1
ASHGV40047581	0.028991242	0.164383305	2.542607	up	protein_coding	NM_002214	ITGB8
ASHGV40057057	0.007068021	0.085285818	2.0654378	up	protein_coding	NM_004564	GATB
ASHGV40025885	0.02756813	0.160592595	2.5959705	up	protein_coding	NM_014475	DHDH
ASHGV40024446	0.003338753	0.064680656	3.501476	up	protein_coding	NM_001712	CEACAM1
ASHGV40027676	0.017144677	0.128795604	3.9170018	up	protein_coding	NM_020675	SPC25
ASHGV40011198	0.00426519	0.070586813	3.9472499	up	protein_coding	NM_018099	FAR2
ASHGV40016217	0.002513066	0.060406687	3.1803448	up	protein_coding	NM_004049	BCL2A1
ASHGV40025794	0.015443765	0.122372976	3.4365186	up	protein_coding	NM_001645	APOC1
ASHGV40026662	0.046460807	0.209061114	2.1324566	up	protein_coding	NM_133329	KCNG3
ASHGV40011529	0.016390851	0.126248248	3.0935963	up	protein_coding	NM_016584	IL23A
ASHGV40055556	0.024947825	0.15262817	2.4052394	up	protein_coding	NM_005393	PLXNB3
ASHGV40051398	0.004075406	0.069284044	2.1444631	up	protein_coding	NM_000837	GRINA
ASHGV40015240	0.02483317	0.152493483	3.1907572	up	protein_coding	NM_052939	FCRL3
ASHGV40038626	0.034267339	0.178673839	2.1009133	up	protein_coding	NM_001010857	LELP1

ASHGV40043554	0.005654427	0.078517713	5.3922473	up	protein_coding	NM_005084	PLA2G7
ASHGV40006123	0.002433132	0.059482588	3.4840083	up	protein_coding	NM_004741	NOLC1
ASHGV40048246	0.015238768	0.121994108	2.2307404	up	protein_coding	NM_001164462	MUC12
ASHGV40042807	0.000104443	0.028718686	2.5684587	up	protein_coding	NM_007255	B4GALT7
ASHGV40009580	0.017397091	0.129519935	3.181104	up	protein_coding	NM_002543	OLR1
ASHGV40024426	0.024778089	0.15232004	6.9761596	up	protein_coding	ENST0000000672	CEACAM7
ASHGV40038151	0.022657711	0.147893728	2.900046	up	protein_coding	NM_173662	RNF175
ASHGV40020420	0.006197026	0.081506727	2.1207208	up	protein_coding	NM_005497	GJC1
ASHGV40005747	0.000288693	0.035122006	19.1379198	up	protein_coding	uc010qje.2	HKDC1
ASHGV40037634	0.045462242	0.20669613	2.9787325	up	protein_coding	NM_002089	CXCL2
ASHGV40040767	0.017812901	0.131381448	2.4580855	up	protein_coding	NM_178450	MARCH3
ASHGV40046327	0.016995459	0.128374093	2.0859024	up	protein_coding	NM_006092	NOD1
ASHGV40031421	0.004051972	0.069284044	3.0678805	up	protein_coding	NM_138283	CSTL1
ASHGV40023798	0.004212706	0.07035008	3.8656995	up	protein_coding	NM_000064	C3
ASHGV40024767	0.046801067	0.209075944	2.3987888	up	protein_coding	NM_198481	VSTM1
ASHGV40023129	0.00083096	0.048843614	3.1235136	up	protein_coding	NM_006868	RAB31
ASHGV40044782	0.047669792	0.211010535	2.350573	up	protein_coding	NM_012367	OR2B6
ASHGV40022442	0.002752376	0.061986011	2.0049408	up	protein_coding	NM_012307	EPB41L3
ASHGV40024465	0.037167472	0.185554194	2.9506634	up	protein_coding	NM_014400	LYPD3
ASHGV40003266	0.025745804	0.155491458	5.2343707	up	protein_coding	NM_005101	ISG15
ASHGV40031545	0.046369695	0.208861278	2.3041978	up	protein_coding	NM_016436	PHF20
ASHGV40019361	0.002442308	0.059482588	2.4809432	up	protein_coding	NM_001361	DHODH
ASHGV40056536	0.006420775	0.082540776	5.9511224	up	protein_coding	uc010ozf.2	PDZK1P1
ASHGV40046292	0.040896092	0.196112282	2.044955	up	protein_coding	NM_003930	SKAP2
ASHGV40015216	0.003505012	0.065507718	3.0485872	up	protein_coding	NM_017791	FLVCR2
ASHGV40041730	0.001193695	0.050120601	3.3402558	up	protein_coding	NM_002203	ITGA2
ASHGV40021543	0.006382988	0.082378819	2.2628362	up	protein_coding	NM_018404	ADAP2
ASHGV40048620	0.012352274	0.109722298	3.6756691	up	protein_coding	NM_014141	CNTNAP2
ASHGV40030460	0.024176767	0.151313517	4.4467927	up	protein_coding	NM_019609	CPXM1
ASHGV40018536	0.012444779	0.110034059	3.95233	up	protein_coding	NM_016111	TELO2
ASHGV40018616	0.002937525	0.061986011	2.6941595	up	protein_coding	NM_001308078	IL32
ASHGV40043464	0.0081992	0.091174268	2.7300897	up	protein_coding	NM_178174	TREML1
ASHGV40013801	0.007549288	0.087925399	2.2978872	up	protein_coding	NM_000396	CTSK
ASHGV40056188	0.004829127	0.073552499	2.457453	up	protein_coding	NM_004039	ANXA2
ASHGV40028956	0.005299663	0.076304639	2.9391107	up	protein_coding	ENST0000037313	RP11-268J15.5
ASHGV40018143	0.000925041	0.049284996	3.1914276	up	protein_coding	NM_004165	RRAD
ASHGV40046546	0.034084306	0.178222837	2.5352553	up	protein_coding	NM_005248	FGR
ASHGV40025564	0.000228858	0.032853377	3.7620725	up	protein_coding	NM_006474	PDPN
ASHGV40025109	0.001817582	0.055772948	2.9207385	up	protein_coding	NM_014257	CLEC4M
ASHGV40016237	0.009742875	0.098938133	8.3924522	up	protein_coding	NM_005814	GPA33
ASHGV40010572	0.009001746	0.095630687	2.1856957	up	protein_coding	NM_021625	TRPV4
ASHGV40003274	0.01666169	0.127466323	2.8843693	up	protein_coding	NM_006398	UBD
ASHGV40029316	0.02660829	0.15791087	2.3231556	up	protein_coding	NM_018715	RCC2
ASHGV40036712	0.011989779	0.108703513	5.278085	up	protein_coding	NM_000902	MME
ASHGV40053538	0.031945462	0.172446155	2.3698534	up	protein_coding	NM_000050	ASS1
ASHGV40044881	0.000166528	0.030140601	2.380526	up	protein_coding	NM_000063	C2
ASHGV40046856	0.002705165	0.061980622	2.6499417	up	protein_coding	ENST0000029237	GPC2
ASHGV40016661	0.033273837	0.17607895	3.8416182	up	protein_coding	NM_001211	BUB1B
ASHGV40008939	0.040324186	0.194439299	2.402558	up	protein_coding	ENST0000043032	C11orf82
ASHGV40002245	0.009528521	0.097844105	4.113929	up	protein_coding	ENST0000056276	RP11-315D16.2
ASHGV40055077	0.010127199	0.100442456	2.2921732	up	protein_coding	NM_012278	ITGB1BP2
ASHGV40036659	0.027050062	0.159100475	5.6089983	up	protein_coding	NM_004617	TM4SF4
ASHGV40038256	0.004806947	0.073532246	2.4681566	up	protein_coding	NM_021647	MFAP3L
ASHGV40012957	0.000209397	0.031004988	2.6132128	up	protein_coding	NM_001303110	COL4A1
ASHGV40018509	0.012306	0.109697039	4.2764843	up	protein_coding	NM_013404	MSLN
ASHGV40021733	0.006006921	0.080593472	2.3528872	up	protein_coding	NM_033133	CNP
ASHGV40003341	0.047083017	0.209580703	2.9296487	up	protein_coding	NM_172089	NFSF12-TNFSF
ASHGV40053946	0.049327311	0.214161104	2.1070149	up	protein_coding	NM_000266	NDP
ASHGV40051391	0.012180031	0.109304951	2.5511425	up	protein_coding	uc022bcj.1	AX746851
ASHGV40054240	0.001879507	0.055772948	2.1160592	up	protein_coding	NM_000169	GLA
ASHGV40032721	0.013415169	0.114197014	2.1304799	up	protein_coding	NM_004571	PKNOX1

ASHGV40014414	0.012397228	0.109724903	2.1353918	up	protein_coding	NM_002961	S100A4
ASHGV40034277	0.00210763	0.056104777	2.4512912	up	protein_coding	NM_147129	ALS2CL
ASHGV40049644	2.86408E-05	0.026283201	91.2186864	up	protein_coding	NM_004063	CDH17
ASHGV40008868	0.001876041	0.055772948	2.5539473	up	protein_coding	NM_001235	SERPINH1
ASHGV40041620	0.027489656	0.160419258	2.210089	up	protein_coding	NM_004172	SLC1A3
ASHGV40046839	0.014273174	0.11755648	7.5843	up	protein_coding	uc031syj.1	YP3A7-CYP3AI
ASHGV40030448	0.02217331	0.146945582	2.2599405	up	protein_coding	NM_006065	SIRPB1
ASHGV40052323	0.035557415	0.181386509	2.2358839	up	protein_coding	NM_012098	ANGPTL2
ASHGV40043535	0.003025251	0.062622161	2.2019081	up	protein_coding	NM_004556	NFKBIE
ASHGV40032410	0.014008909	0.116758763	2.003567	up	protein_coding	NM_006657	FTCD
ASHGV40040967	0.000978404	0.049736021	3.1149782	up	protein_coding	NM_001387	DPYSL3
ASHGV40025797	0.049566946	0.214773676	2.1067897	up	protein_coding	NM_001646	APOC4
ASHGV40025734	0.028993977	0.164383305	8.7588711	up	protein_coding	uc002orj.1	CEACAM5
ASHGV40042368	0.023554992	0.149839682	3.7702822	up	protein_coding	NM_005733	KIF20A
ASHGV40014435	0.003779284	0.067881807	2.5436621	up	protein_coding	NM_080388	S100A16
ASHGV40020173	0.024540075	0.152054281	2.2993282	up	protein_coding	NM_001017368	RFFL
ASHGV40057499	0.020210891	0.140229646	2.753693	up	protein_coding	NM_002172	IFNA14
ASHGV40007452	0.007931925	0.089981161	2.8160248	up	protein_coding	NM_001300995	NOX4
ASHGV40003131	0.023445486	0.149727141	2.2371247	up	protein_coding	NM_001146157	FAM25A
ASHGV40003610	0.01464262	0.119247416	2.108558	up	protein_coding	uc001lgu.3	LOC399815
ASHGV40008164	0.007937831	0.089989605	3.7634075	up	protein_coding	NM_002301	LDHC
ASHGV40024675	0.000654921	0.046153893	11.7900643	up	protein_coding	NM_002257	KLK1
ASHGV40011435	0.005069232	0.07484092	3.5027773	up	protein_coding	NM_012291	ESPL1
ASHGV40048244	0.016700335	0.127466323	3.2301763	up	protein_coding	NM_005960	MUC3A
ASHGV40043144	0.007260163	0.086349392	3.9605196	up	protein_coding	NM_003534	HIST1H3G
ASHGV40029945	0.032748857	0.17477812	2.9558943	up	protein_coding	NM_001080539	CCDC150
ASHGV40046514	0.003369919	0.064680656	7.0364607	up	protein_coding	NM_000790	DDC
ASHGV40032379	0.02798428	0.16146059	2.2621339	up	protein_coding	NM_001303238	ITGB2
ASHGV40034802	0.000112002	0.028718686	3.0018655	up	protein_coding	NM_007085	FSTL1
ASHGV40002602	0.00110079	0.049811075	2.5628234	up	protein_coding	ENST0000059466	AC006486.9
ASHGV40010094	0.038626362	0.190036469	2.315295	up	protein_coding	NM_005379	MYO1A
ASHGV40024929	0.00409854	0.069313435	2.648699	up	protein_coding	NM_005860	FSTL3
ASHGV40041856	0.019615793	0.138407297	2.0409651	up	protein_coding	NM_022909	CENPH
ASHGV40006475	0.002122857	0.056243113	7.4396667	up	protein_coding	NM_031264	CDHR5
ASHGV40042644	0.009875754	0.099354989	3.822276	up	protein_coding	NM_004219	PTTG1
ASHGV40034780	0.041852331	0.198701165	2.2027297	up	protein_coding	NM_002338	LSAMP
ASHGV40045057	0.028419332	0.162785637	3.1687243	up	protein_coding	NM_001123168	FAM72A
ASHGV40057672	0.019269708	0.136825495	2.6990474	up	protein_coding	NM_139214	TGIF2LY
ASHGV40051046	0.001553871	0.053863419	4.231299	up	protein_coding	NM_138455	CTHRC1
ASHGV40029543	0.003893778	0.068716517	2.4372357	up	protein_coding	NM_001301237	CTPS1
ASHGV40046682	0.040218353	0.194311342	3.9783193	up	protein_coding	NM_006072	CCL26
ASHGV40011431	0.000607183	0.044791154	2.0130249	up	protein_coding	NM_001004304	ZNF740
ASHGV40024389	0.033913884	0.177681032	2.662866	up	protein_coding	NM_152479	TTC9B
ASHGV40016027	0.041540364	0.197949655	3.0942433	up	protein_coding	NM_207338	LCTL
ASHGV40022116	0.002858446	0.061986011	2.6293744	up	protein_coding	NM_002266	KPNA2
ASHGV40053492	0.018812756	0.135209899	2.4199995	up	protein_coding	NM_005094	SLC27A4
ASHGV40016036	0.015789508	0.123829936	4.3215524	up	protein_coding	NM_033429	CALML4
ASHGV40048034	0.008533755	0.092938508	2.0981646	up	protein_coding	uc022agm.2	BC063788
ASHGV40008370	0.015139477	0.121646051	3.5806651	up	protein_coding	NM_002391	MDK
ASHGV40034792	0.005258781	0.076092721	3.4755904	up	protein_coding	NM_005191	CD80
ASHGV40016787	0.015228843	0.121970651	2.5858833	up	protein_coding	NM_002009	FGF7
ASHGV40052955	0.017961089	0.131971998	2.2828782	up	protein_coding	NM_004293	GDA
ASHGV40044488	0.00378108	0.067881807	2.2632509	up	protein_coding	NM_001453	FOXC1
ASHGV40043174	0.00345285	0.064944871	4.5576046	up	protein_coding	NM_003535	HIST1H3J
ASHGV40024685	0.011547977	0.106704044	2.0077505	up	protein_coding	NM_002776	KLK10
ASHGV40006314	0.001442394	0.052724506	2.6112571	up	protein_coding	NM_002775	HTRA1
ASHGV40020503	0.002070188	0.055772948	2.9861547	up	protein_coding	NM_002147	HOXB5
ASHGV40011464	0.005799509	0.079434591	3.3959066	up	protein_coding	NM_004503	HOXC6
ASHGV40020199	0.020865584	0.143007986	2.2890375	up	protein_coding	NM_145654	RDM1
ASHGV40057233	0.012505853	0.1104061	2.0034286	up	protein_coding	NM_001271675	LOC441155
ASHGV40009201	0.036292414	0.183365557	3.3658942	up	protein_coding	NM_019894	TMPRSS4

ASHGV40007184	0.00571624	0.079101877	2.4819337	up	protein_coding	NM_005438	FOSL1
ASHGV40051533	0.029646684	0.166261331	3.8629741	up	protein_coding	NM_144966	FREM1
ASHGV40033988	0.001945306	0.055772948	2.7443765	up	protein_coding	NM_173567	EPHX4
ASHGV40007152	0.02762568	0.16063555	2.6434471	up	protein_coding	NM_001282448	TMEM262
ASHGV40018887	0.005859067	0.079836199	2.359949	up	protein_coding	NM_177552	SULT1A3
ASHGV40011135	0.043334809	0.202197868	2.2899417	up	protein_coding	NM_000921	PDE3A
ASHGV40052528	0.0044773	0.071752025	2.7691187	up	protein_coding	NM_178448	SAPCD2
ASHGV40041855	0.012191461	0.109304951	3.0352602	up	protein_coding	NM_031966	CCNB1
ASHGV40013362	0.000920426	0.049228681	56.4755523	up	protein_coding	NM_006418	OLFM4
ASHGV40044876	0.009649861	0.098337216	2.0215113	up	protein_coding	NM_001039651	SAPCD1
ASHGV40045645	0.016357964	0.126202417	3.0502199	up	protein_coding	NM_001012507	CENPW
ASHGV40039684	0.001593056	0.054536631	2.9834177	up	protein_coding	NM_018248	NEIL3
ASHGV40030676	0.000108306	0.028718686	5.7666601	up	protein_coding	NM_001898	CST1
ASHGV40007731	0.014810758	0.120055964	4.082631	up	protein_coding	NM_012101	TRIM29
ASHGV40005997	0.027289883	0.15979234	2.7710886	up	protein_coding	NM_004523	KIF11
ASHGV40009550	0.043268418	0.202151164	2.633616	up	protein_coding	NM_014358	CLEC4E
ASHGV40026423	0.004270943	0.070586813	2.4856096	up	protein_coding	NM_000929	PLA2G5
ASHGV40056975	0.000250592	0.033353656	12.3367716	up	protein_coding	NM_138461	TM4SF19
ASHGV40048562	0.027207555	0.159512754	3.3497486	up	protein_coding	NM_006027	EXO1
ASHGV40040797	0.025843041	0.155755019	2.2786271	up	protein_coding	NM_001136219	FCGR2A
ASHGV40022736	0.0126942	0.111052115	11.229966	up	protein_coding	NM_001085474	LYPD8
ASHGV40057052	5.14513E-05	0.028718686	3.2120117	up	protein_coding	NM_012118	NOCT
ASHGV40027255	0.029079648	0.164407506	3.5666692	up	protein_coding	NM_004336	BUB1
ASHGV40055516	0.008703865	0.093650627	2.5074253	up	protein_coding	NM_005342	HMGB3
ASHGV40048248	0.000507606	0.042546158	15.0648183	up	protein_coding	NM_001040105	MUC17
ASHGV40020459	0.006045897	0.080593472	3.2285563	up	protein_coding	NM_030753	WNT3
ASHGV40031476	0.009182763	0.09656855	4.5738203	up	protein_coding	NM_012112	TPX2
ASHGV40022084	0.023653267	0.149889765	2.3560509	up	protein_coding	NM_001085423	MILR1
ASHGV40037720	0.023514789	0.149834056	2.3519194	up	protein_coding	NM_006665	HPSE
ASHGV40056429	0.015052234	0.121112485	2.0241338	up	protein_coding	NM_002949	MRPL12
ASHGV40027928	0.001164485	0.050017015	3.7919997	up	protein_coding	NM_033066	MPP4
ASHGV40021289	0.038722189	0.190293148	3.0372116	up	protein_coding	ENST0000058104	AC000003.2
ASHGV40005219	0.00649162	0.082800205	2.7797294	up	protein_coding	NM_002627	PFKP
ASHGV40024227	0.016887197	0.12782262	4.5782089	up	protein_coding	NM_014270	SLC7A9
ASHGV40044411	0.008968334	0.095468999	4.5827575	up	protein_coding	ENST0000036682	C6orf123
ASHGV40024007	0.00128501	0.051153952	2.4683629	up	protein_coding	NM_000435	NOTCH3
ASHGV40019310	0.021348274	0.144442569	3.6982667	up	protein_coding	NM_001793	CDH3
ASHGV40006708	0.027972363	0.161445259	3.4760589	up	protein_coding	NM_005709	USH1C
ASHGV40018285	0.013601692	0.114847021	4.0170186	up	protein_coding	NM_199355	ADAMTS18
ASHGV40008165	0.0286002	0.163392232	2.5967856	up	protein_coding	NM_144972	LDHAL6A
ASHGV40019732	0.001688257	0.055003756	2.2360045	up	protein_coding	NM_182566	VMO1
ASHGV40039201	0.004317087	0.071012005	45.1558951	up	protein_coding	NM_000253	MTTP
ASHGV40015832	0.025225976	0.15384404	2.0044757	up	protein_coding	NM_000138	FBN1
ASHGV40042867	0.0066471	0.08340168	2.5409498	up	protein_coding	NM_197975	BTNL3
ASHGV40049465	0.001157823	0.050016538	5.5943509	up	protein_coding	NM_020647	JPH1
ASHGV40018962	0.001421529	0.052512256	2.9688308	up	protein_coding	NM_000887	ITGAX
ASHGV40016477	0.006858748	0.084455602	3.2499246	up	protein_coding	NM_000655	SELL
ASHGV40038684	0.000932114	0.049399233	16.1386641	up	protein_coding	NM_020973	GBA3
ASHGV40026445	0.013058461	0.112605006	3.1553459	up	protein_coding	NM_152376	UBXN10
ASHGV40023419	0.000420782	0.040349735	32.5886368	up	protein_coding	NM_004852	ONECUT2
ASHGV40053971	0.03598866	0.182518404	2.0517226	up	protein_coding	NM_002621	CFP
ASHGV40043369	0.031862808	0.172378528	6.6157414	up	protein_coding	NM_002418	MLN
ASHGV40008700	0.00914755	0.096506793	2.7505885	up	protein_coding	NM_024036	LRFN4
ASHGV40029971	0.008673947	0.093650627	2.530535	up	protein_coding	NM_152524	SGOL2
ASHGV40049321	0.02520221	0.153806697	3.3097357	up	protein_coding	NM_138969	SDR16C5
ASHGV40013255	0.005560698	0.077963502	6.369679	up	protein_coding	NM_003701	TNFSF11
ASHGV40014421	0.001799341	0.055626431	6.3403075	up	protein_coding	NM_002960	S100A3
ASHGV40007532	0.028502809	0.162996057	13.3796014	up	protein_coding	NM_002423	MMP7
ASHGV40021730	0.044517385	0.204930456	2.4738827	up	protein_coding	NM_021939	FKBP10
ASHGV40022314	0.004024061	0.069284044	3.4374886	up	protein_coding	ENST0000032831	ENPP7
ASHGV40014866	0.033667765	0.177029899	3.4599516	up	protein_coding	NM_004086	COCH

ASHGV40025011	0.001601443	0.054536631	2.0786988	up	protein_coding	NM_020170	NCLN
ASHGV40018184	0.00949171	0.097753962	4.5207166	up	protein_coding	NM_014875	KIF14
ASHGV40025989	0.035282532	0.180724509	2.1594911	up	protein_coding	NM_014225	PPP2R1A
ASHGV40024448	0.033810477	0.177512642	8.1048174	up	protein_coding	NM_001816	CEACAM8
ASHGV40024778	0.001225434	0.050556396	2.4319717	up	protein_coding	NM_024318	LILRA6
ASHGV40028248	0.031642079	0.171559428	2.0597278	up	protein_coding	NM_024726	IQCA1
ASHGV40028777	0.004801482	0.073532246	4.0527767	up	protein_coding	NM_138370	PKDCC
ASHGV40024706	0.019776973	0.138849219	2.8978519	up	protein_coding	NM_003830	SIGLEC5
ASHGV40034987	0.0016597	0.054739049	18.6757608	up	protein_coding	NM_004164	RBP2
ASHGV40046087	0.001652581	0.054739049	2.1236482	up	protein_coding	ENST0000022299	SNX8
ASHGV40042355	0.001391252	0.052512256	2.7421061	up	protein_coding	NM_000358	TGFBI
ASHGV40033996	0.032454984	0.174064615	2.0283284	up	protein_coding	NM_001001331	ATP2B2
ASHGV40009579	0.004932589	0.074078062	2.4961407	up	protein_coding	NM_197947	CLEC7A
ASHGV40043465	0.001318826	0.05210508	6.8881158	up	protein_coding	NM_018965	TREM2
ASHGV40044828	0.000525955	0.043246784	6.8685479	up	protein_coding	NM_033229	TRIM15
ASHGV40020336	0.022488484	0.147541072	2.5275961	up	protein_coding	NM_002230	JUP
ASHGV40016187	0.012326711	0.109713394	3.3264321	up	protein_coding	NM_006383	CIB2
ASHGV40045066	0.022835919	0.148127635	3.0116129	up	protein_coding	NM_002821	PTK7
ASHGV40035332	0.011007372	0.104272803	3.6660932	up	protein_coding	NM_014398	LAMP3
ASHGV40005815	0.0001118	0.028718686	4.220013	up	protein_coding	NM_002658	PLAU
ASHGV40014679	0.016620453	0.127281349	2.8410686	up	protein_coding	NM_207370	GPR153
ASHGV40054592	0.00158594	0.054536631	2.8091411	up	protein_coding	NM_001042351	G6PD
ASHGV40017424	0.000701509	0.046533174	2.7702857	up	protein_coding	NM_001031737	CCDC78
ASHGV40020149	0.002670412	0.061839224	2.3251217	up	protein_coding	NM_015194	MYO1D
ASHGV40015523	0.011539679	0.106699757	2.2506455	up	protein_coding	ENST0000042916	AL049840.1
ASHGV40038783	0.001116264	0.049811075	2.4605267	up	protein_coding	NM_015173	TBC1D1
ASHGV40032431	0.001012059	0.049811075	3.4747225	up	protein_coding	NM_002600	PDE4B
ASHGV40022311	0.043337722	0.202197868	2.0572933	up	protein_coding	NM_001042573	ENGASE
ASHGV40023965	0.001638851	0.054621802	2.7102894	up	protein_coding	NM_017722	TRMT1
ASHGV40048198	0.004222898	0.07035008	2.6953291	up	protein_coding	NM_005720	ARPC1B
ASHGV40019785	2.28135E-05	0.025539914	15.6299765	up	protein_coding	NM_001307	CLDN7
ASHGV40041093	0.022517022	0.147541072	3.6911941	up	protein_coding	NM_025153	ATP10B
ASHGV40028065	0.038614808	0.190035503	2.1006677	up	protein_coding	NM_022453	RNF25
ASHGV40048217	0.00723089	0.086258773	3.7041947	up	protein_coding	NM_012447	STAG3
ASHGV40024109	0.005464582	0.077372027	4.3233903	up	protein_coding	NM_001001524	TM6SF2
ASHGV40056741	0.005014253	0.074661416	2.7704474	up	protein_coding	NM_005301	GPR35
ASHGV40054582	6.69592E-06	0.018057943	2.3058445	up	protein_coding	NM_001110556	FLNA
ASHGV40025339	0.004812239	0.073532246	2.9079085	up	protein_coding	NM_015122	FCHO1
ASHGV40029943	0.006715308	0.083754019	2.363615	up	protein_coding	NM_020342	SLC39A10
ASHGV40016690	0.010228158	0.101068491	2.5158643	up	protein_coding	NM_019074	DLL4
ASHGV40033425	0.000609986	0.044791154	6.3323274	up	protein_coding	NM_018584	CAMK2N1
ASHGV40008274	0.01620514	0.125579029	2.2611119	up	protein_coding	NM_001282670	FAAP20
ASHGV40005726	0.016905194	0.12782262	3.1280955	up	protein_coding	NM_178011	LRRTM3
ASHGV40001343	0.017374944	0.129410305	2.6827319	up	protein_coding	NM_002664	PLEK
ASHGV40019635	0.03782361	0.187462325	2.1893566	up	protein_coding	ENST0000056796	TUBB8P7
ASHGV40016338	6.99387E-05	0.028718686	21.637745	up	protein_coding	NM_001150	ANPEP
ASHGV40005229	0.01225755	0.109442525	2.8774165	up	protein_coding	NM_024700	SNIP1
ASHGV40041179	0.033348547	0.176201594	2.0851739	up	protein_coding	NM_003714	STC2
ASHGV40013357	0.027444522	0.160309106	2.3395084	up	protein_coding	NM_018204	CKAP2
ASHGV40012883	0.01549231	0.122672081	9.0269779	up	protein_coding	NM_033132	ZIC5
ASHGV40054950	0.013998762	0.11674283	2.9097535	up	protein_coding	NM_002049	GATA1
ASHGV40023662	0.034722872	0.17943745	2.9139815	up	protein_coding	NM_138774	R3HDM4
ASHGV40023400	0.043100166	0.201635228	2.5012439	up	protein_coding	NM_173529	C18orf54
ASHGV40003112	0.014234504	0.117526372	2.0167073	up	protein_coding	NM_001099285	PTMA
ASHGV40033609	0.0273223	0.15986296	4.373416	up	protein_coding	NM_012128	CLCA4
ASHGV40009643	0.000186693	0.030140601	13.2111924	up	protein_coding	NM_004963	GUCY2C
ASHGV40052000	0.0111653	0.104993631	3.0107544	up	protein_coding	NM_017680	ASPN
ASHGV40021793	0.03251518	0.174104439	2.1152116	up	protein_coding	NM_024032	C17orf53
ASHGV40029902	0.03505547	0.180196689	2.0933813	up	protein_coding	NM_002194	INPP1
ASHGV40043141	0.002259342	0.058058229	2.2760993	up	protein_coding	NM_005320	HIST1H1D
ASHGV40007720	0.010465222	0.102397086	2.6701102	up	protein_coding	NM_002105	H2AFX

ASHGV40015923	0.004918104	0.073925721	3.5029919	up	protein_coding	NM_001297713	OLFML2B
ASHGV40056255	0.004644926	0.072766338	2.0451182	up	protein_coding	uc002dey.2	AK310228
ASHGV40043701	0.001742506	0.055333849	2.2709456	up	protein_coding	NM_138441	MB21D1
ASHGV40041283	0.027166809	0.159394295	2.4642413	up	protein_coding	NM_005110	GFPT2
ASHGV40016646	0.035585501	0.181476686	2.5174058	up	protein_coding	NM_006786	UTS2
ASHGV40010952	0.006398594	0.082458161	3.1561652	up	protein_coding	NM_002527	NTF3
ASHGV40033214	0.042128765	0.199283002	2.2464116	up	protein_coding	NM_024053	CENPM
ASHGV40049448	0.007983381	0.090176651	2.6137753	up	protein_coding	NM_005098	MSC
ASHGV40031834	0.042743065	0.20057805	2.144946	up	protein_coding	NM_002591	PCK1
ASHGV40033226	0.032797612	0.174880478	2.0572308	up	protein_coding	NM_015703	RRP7A
ASHGV40033448	0.027082485	0.159100475	3.1330308	up	protein_coding	NM_144704	AIFM3
ASHGV40028265	2.6366E-05	0.025539914	6.1587713	up	protein_coding	NM_004369	COL6A3
ASHGV40023999	1.80144E-05	0.025539914	3.0625944	up	protein_coding	NM_013447	ADGRE2
ASHGV40014215	0.022242345	0.14730461	2.7687143	up	protein_coding	NM_016445	PLEK2
ASHGV40043712	0.000375829	0.039736903	4.8494725	up	protein_coding	NM_004370	COL12A1
ASHGV40040247	0.000134522	0.029433159	7.0482768	up	protein_coding	NM_007036	ESM1
ASHGV40043140	0.000861931	0.048985823	2.174235	up	protein_coding	NM_003518	HIST1H2BG
ASHGV40030868	0.00566502	0.078517713	2.0824952	up	protein_coding	NM_001080472	FITM2
ASHGV40027628	0.0212772	0.144232877	2.4274855	up	protein_coding	NM_006142	SFN
ASHGV40007378	0.002076118	0.055772948	2.0136028	up	protein_coding	NM_004631	LRP8
ASHGV40018837	0.002320421	0.058860464	3.549421	up	protein_coding	NM_052944	SLC5A11
ASHGV40009399	0.013609286	0.114855524	3.1382226	up	protein_coding	NM_001080407	GLB1L3
ASHGV40028611	0.045482102	0.206732514	2.1502166	up	protein_coding	NM_024322	CENPO
ASHGV40019822	0.004434442	0.071459267	2.3293623	up	protein_coding	NM_004217	AURKB
ASHGV40047825	0.000579824	0.044687591	3.8794591	up	protein_coding	NM_003364	UPP1
ASHGV40048943	0.016012376	0.124881034	2.4839308	up	protein_coding	NM_138715	MSR1
ASHGV40024699	0.004422561	0.071459267	2.4166957	up	protein_coding	NM_001193623	C19orf84
ASHGV40009708	0.000528307	0.043246784	3.0216563	up	protein_coding	NM_005504	BCAT1
ASHGV40006666	0.004273599	0.070586813	2.1315687	up	protein_coding	NM_013253	DKK3
ASHGV40014554	0.027267067	0.15979234	2.3470543	up	protein_coding	NM_138576	BCL11B
ASHGV40021233	0.005403206	0.076906366	2.0192604	up	protein_coding	NM_152379	C1orf131
ASHGV40011330	0.02924558	0.164971187	2.9274516	up	protein_coding	NM_152319	C12orf54
ASHGV40049564	0.04562988	0.207080587	6.0017979	up	protein_coding	NM_001738	CA1
ASHGV40027562	0.000445309	0.041719107	2.0687299	up	protein_coding	NM_002953	RPS6KA1
ASHGV40027276	0.000485746	0.041928091	21.3584958	up	protein_coding	NM_000575	IL1A
ASHGV40046797	0.000390096	0.03988564	2.3035103	up	protein_coding	NM_000305	PON2
ASHGV40040886	0.024207399	0.151340367	2.3568432	up	protein_coding	NM_198282	TMEM173
ASHGV40006982	0.002517724	0.060406687	2.0652269	up	protein_coding	NM_005161	APLNR
ASHGV40006868	0.037435278	0.186308886	2.6103622	up	protein_coding	NM_003035	STIL
ASHGV40031919	0.000202423	0.031004988	2.6144167	up	protein_coding	NM_007346	OGFR
ASHGV40028941	0.001209143	0.0505256	5.142901	up	protein_coding	NM_024676	SH3D21
ASHGV40019742	0.042310455	0.199817198	2.4695514	up	protein_coding	NM_017986	SLC52A1
ASHGV40050900	0.001036754	0.049811075	6.3594615	up	protein_coding	NM_152565	ATP6V0D2
ASHGV40032314	0.00044092	0.041719107	17.0626456	up	protein_coding	NM_003226	TFF3
ASHGV40049013	0.013462557	0.114242293	2.0011092	up	protein_coding	NM_003844	TNFRSF10A
ASHGV40018973	0.040601449	0.195128683	2.0412332	up	protein_coding	NM_003414	ZNF267
ASHGV40026091	0.000903357	0.049204994	2.6616664	up	protein_coding	NM_007279	U2AF2
ASHGV40028674	0.019229764	0.136825495	2.4600563	up	protein_coding	NM_015131	WDR43
ASHGV40037850	0.022937535	0.148477601	3.4637911	up	protein_coding	NM_014421	DKK2
ASHGV40015008	0.006581397	0.08309431	3.3324197	up	protein_coding	NM_015589	SAMD4A
ASHGV40023551	0.032523389	0.174104439	6.4742979	up	protein_coding	NM_032649	CNDP1
ASHGV40046061	0.010260572	0.101132463	2.1601319	up	protein_coding	NM_001284309	ADAP1
ASHGV40018895	0.006267455	0.081594928	7.2256868	up	protein_coding	NM_152338	ZG16
ASHGV40003074	0.000799401	0.048770305	2.5082049	up	protein_coding	NM_014984	CEP131
ASHGV40025735	0.000824459	0.048843614	16.1677495	up	protein_coding	ENST0000019976	CEACAM6
ASHGV40039142	0.005181095	0.075909447	13.1700685	up	protein_coding	NM_001040058	SPP1
ASHGV40021922	0.002078597	0.055772948	2.7912091	up	protein_coding	NM_152463	EME1
ASHGV40038931	0.008552911	0.093017817	4.0563764	up	protein_coding	NM_206919	ARL9
ASHGV40033151	8.35773E-05	0.028718686	3.3465256	up	protein_coding	NM_025045	BAIAP2L2
ASHGV40016686	0.009387582	0.097468969	2.1375576	up	protein_coding	NM_181642	SPINT1
ASHGV40013825	0.024695509	0.152291693	2.5066517	up	protein_coding	NM_001126105	SLC7A7

ASHGV40045062	0.000987999	0.049788318	2.3319503	up	protein_coding	NM_006245	PPP2R5D
ASHGV40037050	0.000918018	0.049228681	32.8318794	up	protein_coding	NM_152672	SLC51A
ASHGV40023745	0.045073116	0.205778077	2.0358427	up	protein_coding	NM_001013841	STAP2
ASHGV40005084	0.031804429	0.172271523	3.8507235	up	protein_coding	NM_002417	MKI67
ASHGV40044080	0.000707515	0.046533174	2.3876638	up	protein_coding	NM_001431	EPB41L2
ASHGV40030739	0.00069876	0.046533174	4.492087	up	protein_coding	NM_004118	FOXS1
ASHGV40023267	0.037413137	0.186308886	4.0690119	up	protein_coding	NM_001944	DSG3
ASHGV40052219	0.00179487	0.055586762	3.2951914	up	protein_coding	NM_002160	TNC
ASHGV40016096	0.033178541	0.175943138	2.1065447	up	protein_coding	NM_002654	PKM
ASHGV40053882	0.002194407	0.057192359	3.1113952	up	protein_coding	NM_031894	FTHL17
ASHGV40000740	0.014191716	0.117440322	2.0907592	up	protein_coding	NM_003748	ALDH4A1
ASHGV40016679	0.002018866	0.055772948	2.9283282	up	protein_coding	NM_170589	CASC5
ASHGV40020326	0.013576685	0.114747006	5.6239273	up	protein_coding	NM_002275	KRT15
ASHGV40053720	0.008078755	0.090554534	2.5279779	up	protein_coding	NM_000047	ARSE
ASHGV40035065	0.048125748	0.212214493	3.6029938	up	protein_coding	NM_000096	CP
ASHGV40033665	0.000349016	0.038034032	3.7920522	up	protein_coding	NM_002133	HMOX1
ASHGV40028271	0.022736553	0.147893728	2.8767053	up	protein_coding	NM_001525	HCRTR1
ASHGV40011213	0.010733226	0.103340808	2.5070836	up	protein_coding	NM_152438	DDX11
ASHGV40038663	0.027169067	0.159394295	3.6079279	up	protein_coding	NM_022346	NCAPG
ASHGV40033028	0.000839813	0.048843614	7.5196862	up	protein_coding	NM_002309	LIF
ASHGV40047398	0.025913153	0.155887709	2.1111504	up	protein_coding	NM_001010867	IBA57
ASHGV40028154	0.001263806	0.05083886	27.5030446	up	protein_coding	NM_024795	TM4SF20
ASHGV40036381	0.007051459	0.085147057	3.4489828	up	protein_coding	NM_001014980	FAM132A
ASHGV40027908	0.036529531	0.183870928	2.0668905	up	protein_coding	NM_001269	RCC1
ASHGV40033160	0.037561732	0.186854881	2.2287152	up	protein_coding	NM_007068	DMC1
ASHGV40018234	0.022620737	0.147831772	2.395766	up	protein_coding	NM_031293	PMFBP1
ASHGV40005069	0.041332444	0.197429269	2.1512886	up	protein_coding	NM_005202	COL8A2
ASHGV40023778	0.019962337	0.139281033	4.8392144	up	protein_coding	NM_000149	FUT3
ASHGV40008784	0.002185714	0.057051057	2.116362	up	protein_coding	NM_005231	CTTN
ASHGV40049261	0.003358205	0.064680656	2.1543334	up	protein_coding	NM_177987	TUBB8
ASHGV40045646	0.029210063	0.164877522	2.0265548	up	protein_coding	NM_005525	HSD11B1
ASHGV40044655	0.00090387	0.049204994	2.4378523	up	protein_coding	NM_006366	CAP2
ASHGV40025282	0.024216542	0.151340367	2.3961298	up	protein_coding	NM_000896	CYP4F3
ASHGV40029911	0.010943798	0.104057649	3.9589945	up	protein_coding	NM_001255	CDC20
ASHGV40010708	0.018940617	0.135904775	2.0002958	up	protein_coding	NM_019034	RHOF
ASHGV40046307	8.28536E-06	0.018057943	98.4153243	up	protein_coding	NM_000522	HOXA13
ASHGV40017485	0.010432316	0.102226677	2.6805624	up	protein_coding	NM_002528	NTHL1
ASHGV40052493	0.000976156	0.049736021	2.076771	up	protein_coding	NM_052813	CARD9
ASHGV40047631	0.026643117	0.158063758	2.8402464	up	protein_coding	NM_013322	SNX10
ASHGV40007352	0.037037284	0.185191074	2.2726777	up	protein_coding	NM_001005285	OR2AT4
ASHGV40010807	0.034362691	0.178796741	2.8783395	up	protein_coding	NM_001143989	NBPF4
ASHGV40027638	0.000211608	0.031004988	9.0604844	up	protein_coding	NM_001291807	FAP
ASHGV40050681	0.001399187	0.052512256	2.0708046	up	protein_coding	NM_001011671	CHCHD7
ASHGV40050374	0.001626894	0.054621802	2.0583908	up	protein_coding	NM_003841	TNFRSF10C
ASHGV40055519	0.006065472	0.080730965	2.1474991	up	protein_coding	NM_001017980	VMA21
ASHGV40010932	0.027491371	0.160419258	2.5804889	up	protein_coding	NM_001759	CCND2
ASHGV40030321	0.002621151	0.061427953	4.1074406	up	protein_coding	NM_006845	KIF2C
ASHGV40042647	0.017341815	0.129328049	2.3170027	up	protein_coding	NM_016545	IER5
ASHGV40039098	0.01645738	0.126465792	2.2212499	up	protein_coding	NM_025074	FRAS1
ASHGV40033170	0.036331947	0.183498527	2.0906422	up	protein_coding	NM_014292	CBX6
ASHGV40005377	0.008517511	0.092877627	2.3973535	up	protein_coding	NM_032812	PLXDC2
ASHGV40034445	0.004697677	0.073060937	3.8933741	up	protein_coding	NM_003392	WNT5A
ASHGV40030010	0.00789824	0.089971932	2.3773563	up	protein_coding	NM_003872	NRP2
ASHGV40022681	0.030137299	0.16715585	2.2598482	up	protein_coding	NM_015476	TPGS2
ASHGV40013585	0.000829352	0.048843614	2.9188487	up	protein_coding	NM_021059	HIST2H3C
ASHGV40046240	0.023922671	0.150585114	2.5901156	up	protein_coding	NM_207342	STEAP1B
ASHGV40006316	4.02414E-05	0.027456531	54.3863454	up	protein_coding	NM_007329	DMBT1
ASHGV40021173	0.014726377	0.11953869	3.6587982	up	protein_coding	NM_000029	AGT
ASHGV40021449	0.022984219	0.148477601	2.5282968	up	protein_coding	NM_145109	MAP2K3
ASHGV40046884	0.005900213	0.080032877	4.2572487	up	protein_coding	NM_014343	CLDN15
ASHGV40032778	0.001584478	0.054536631	3.1706358	up	protein_coding	NM_030582	COL18A1

ASHGV40035958	0.043812677	0.203440168	2.1540469	up	protein_coding	NM_000172	GNAT1
ASHGV40030729	0.036615503	0.184062726	2.0835091	up	protein_coding	NM_153289	DEFB119
ASHGV40032722	0.001694025	0.055003756	6.9953276	up	protein_coding	NM_019062	RNF186
ASHGV40051675	0.001281156	0.051153952	2.2494631	up	protein_coding	NM_194313	KIF24
ASHGV40052430	0.001253208	0.05083886	2.2530207	up	protein_coding	NM_006266	RALGDS
ASHGV40036273	0.000674547	0.046153893	7.0137218	up	protein_coding	NM_001850	COL8A1
ASHGV40038639	0.001014831	0.049811075	3.1492956	up	protein_coding	NM_004334	BST1
ASHGV40006222	0.001943184	0.055772948	3.6981065	up	protein_coding	NM_024889	PLEKHS1

Table S3 Differentially expressed mRNAs between lymph node metastasis positive gastric cancer and negative gastric cancer tissues

ProbeName	P-value	FDR	Fold Change	Regulation	type	seqname	GeneSymbol
ASHGV40028329	0.033997189	0.838838466	2.2925512	up	protein_coding	NM_001085437	C2orf54
ASHGV40038258	0.04748993	0.838838466	2.1083973	up	protein_coding	NM_016228	AADAT
ASHGV40049900	0.045981503	0.838838466	2.0706491	up	protein_coding	NM_014751	MTSS1
ASHGV40039043	0.034884362	0.838838466	7.7337045	up	protein_coding	NM_000477	ALB
ASHGV40038744	0.02500711	0.838838466	2.6785282	up	protein_coding	NM_002589	PCDH7
ASHGV40019128	0.010064533	0.838838466	2.2668403	up	protein_coding	NM_005853	IRX5
ASHGV40046463	0.020183197	0.838838466	2.006965	up	protein_coding	NM_018650	MARK1
ASHGV40031298	0.018428976	0.838838466	2.1435225	up	protein_coding	NM_015192	PLCB1
ASHGV40047091	0.016986326	0.838838466	2.3348011	up	protein_coding	NM_000845	GRM8
ASHGV40023269	0.020072683	0.838838466	2.4411653	up	protein_coding	NM_000371	TTR
ASHGV40018735	0.033622684	0.838838466	2.3523333	up	protein_coding	NM_001128423	MPV17L
ASHGV40014267	0.025288932	0.838838466	3.6049109	up	protein_coding	NM_001014342	FLG2
ASHGV40053219	0.045547979	0.838838466	2.0838604	up	protein_coding	NM_006981	NR4A3
ASHGV40036670	0.014143525	0.838838466	2.4985175	up	protein_coding	NM_014779	TSC22D2
ASHGV40023044	0.040857514	0.838838466	3.4309997	up	protein_coding	NM_001117	ADCYAP1
ASHGV40006570	0.025184374	0.838838466	2.3570809	up	protein_coding	NM_000184	HBG2
ASHGV40012122	0.044746668	0.838838466	2.2077707	up	protein_coding	NM_144668	WDR66
ASHGV40029324	0.030197574	0.838838466	2.3338228	up	protein_coding	NM_138621	BCL2L11
ASHGV40049691	0.015068592	0.838838466	2.174624	up	protein_coding	NM_001029860	FBXO43
ASHGV40011775	0.022655013	0.838838466	2.0343157	up	protein_coding	NM_001009894	C12orf29
ASHGV40008189	0.04790329	0.838838466	2.453386	up	protein_coding	NM_213599	ANO5
ASHGV40007109	0.032691441	0.838838466	2.8072611	up	protein_coding	NM_054108	HRASLS5
ASHGV40031764	0.010693758	0.838838466	2.3081824	up	protein_coding	NM_017843	BCAS4
ASHGV40001274	0.046750419	0.838838466	2.2034883	up	protein_coding	uc001jtv.4	TTC18
ASHGV40057429	0.000879941	0.838838466	3.364602	up	protein_coding	NM_001206847	SMIM18
ASHGV40041168	0.011396484	0.838838466	2.7821068	up	protein_coding	uc021yhu.1	AK316321
ASHGV40038491	0.027460442	0.838838466	2.0453996	up	protein_coding	NM_182982	GRK4
ASHGV40043739	0.023708573	0.838838466	2.0720058	up	protein_coding	NM_181714	LCA5
ASHGV40042429	0.010170002	0.838838466	2.7272131	up	protein_coding	NM_018899	PCDHAC2
ASHGV40015458	0.02030956	0.838838466	2.0577119	up	protein_coding	ENST00000599197	AL117190.3
ASHGV40034109	0.03284106	0.838838466	2.009707	up	protein_coding	NM_005442	EOMES
ASHGV40055272	0.000207345	0.837047987	2.1813973	up	protein_coding	NM_012216	MID2
ASHGV40027561	0.039043243	0.838838466	3.488147	up	protein_coding	NM_004543	NEB
ASHGV40030033	0.004782325	0.838838466	2.0922046	up	protein_coding	NM_173077	CPO
ASHGV40011408	0.013208561	0.838838466	3.3666509	up	protein_coding	NM_005556	KRT7
ASHGV40033544	0.027409722	0.838838466	2.9178972	up	protein_coding	NM_001554	CYR61
ASHGV40006565	0.038804529	0.838838466	2.0223019	up	protein_coding	NM_000518	HBB
ASHGV40052960	0.028477363	0.838838466	2.0837338	up	protein_coding	NM_000700	ANXA1
ASHGV40000157	0.016657065	0.838838466	2.107002	up	protein_coding	NM_021246	LY6G6D
ASHGV40055954	0.004285033	0.838838466	3.1404637	up	protein_coding	NM_001129742	CALHM3
ASHGV40003261	0.034708774	0.838838466	2.8797019	up	protein_coding	NM_004417	DUSP1
ASHGV40015184	0.026136494	0.838838466	2.1714456	up	protein_coding	NM_025057	CCDC176
ASHGV40053946	0.046156665	0.838838466	2.0781561	up	protein_coding	NM_000266	NDP
ASHGV40019151	0.02966665	0.838838466	2.5789595	up	protein_coding	ENST00000219162	MT4
ASHGV40042525	0.045429746	0.838838466	2.8572784	up	protein_coding	NM_006846	SPINK5
ASHGV40028518	0.025167248	0.838838466	2.2335497	up	protein_coding	NM_021643	TRIB2
ASHGV40052885	0.012124696	0.838838466	2.0156145	up	protein_coding	uc004ady.3	AL953854.2-002
ASHGV40011030	0.028682559	0.838838466	2.0250377	up	protein_coding	NM_005810	KLRG1
ASHGV40028103	0.015235586	0.838838466	2.2715264	up	protein_coding	NM_004438	EPHA4
ASHGV40005650	0.002363978	0.838838466	9.2186301	up	protein_coding	NM_012242	DKK1
ASHGV40035094	0.025010336	0.838838466	4.6531192	up	protein_coding	NM_023915	GPR87

ASHGV40043936	0.011470148	0.838838466	2.1764047	up	protein_coding	NM_173672	PPIL6
ASHGV40003215	0.004847055	0.838838466	14.9104483	up	protein_coding	NM_212557	AMTN
ASHGV40017369	0.010482049	0.838838466	2.1671177	up	protein_coding	ENST00000558188	CTD-2054N24.2
ASHGV40016814	0.019448341	0.838838466	2.0001387	up	protein_coding	NM_014548	TMOD2
ASHGV40001985	0.022433532	0.838838466	2.0459236	up	protein_coding	NM_004282	BAG2
ASHGV40045622	0.02234046	0.838838466	4.6485068	up	protein_coding	NM_032471	PKIB
ASHGV40050195	0.015408729	0.838838466	2.2938314	up	protein_coding	NM_003970	MYOM2
ASHGV40042883	1.29722E-05	0.226183546	2.2905325	up	protein_coding	NM_001005221	OR4F29
ASHGV40009687	0.01436583	0.838838466	2.0704831	up	protein_coding	NM_021957	GYS2
ASHGV40043141	0.026381265	0.838838466	2.5571749	up	protein_coding	NM_005320	HIST1H1D
ASHGV40057279	0.049188623	0.838838466	3.4598843	up	protein_coding	NM_001276687	MT1HL1
ASHGV40050929	0.017096754	0.838838466	2.6842785	up	protein_coding	NM_001129890	LRRC69
ASHGV40043140	0.023795147	0.838838466	2.660415	up	protein_coding	NM_003518	HIST1H2BG
ASHGV40001048	0.005768715	0.838838466	2.01017	up	protein_coding	NM_181506	LRRC70
ASHGV40052615	0.031092148	0.838838466	2.6208182	up	protein_coding	NM_033439	IL33
ASHGV40019154	0.042566329	0.838838466	2.5874956	up	protein_coding	NM_175617	MT1E
ASHGV40000238	0.044367491	0.838838466	2.5683152	up	protein_coding	ENST00000401851	MGC10955
ASHGV40045115	0.004364406	0.838838466	2.2325562	up	protein_coding	NM_001010870	TDRD6
ASHGV40042440	0.019736502	0.838838466	2.9307109	up	protein_coding	ENST00000239444	PCDHB8
ASHGV40015210	0.032291672	0.838838466	2.5409491	up	protein_coding	NM_005252	FOS
ASHGV40001212	0.002400378	0.838838466	2.4519389	up	protein_coding	ENST00000456475	OR4F3
ASHGV40024533	0.004994739	0.838838466	2.5849956	down	protein_coding	NM_175875	SIX5
ASHGV40037165	0.000863638	0.838838466	4.1050656	down	protein_coding	NM_006099	PIAS3
ASHGV40045161	0.031198758	0.838838466	2.6543267	down	protein_coding	NM_002190	IL17A
ASHGV40051412	0.045820643	0.838838466	2.4964214	down	protein_coding	NM_005526	HSF1
ASHGV40038498	0.018067547	0.838838466	2.0230425	down	protein_coding	NM_178438	LCE5A
ASHGV40035757	0.017776102	0.838838466	2.07942	down	protein_coding	NM_178868	CMTM8
ASHGV40038487	0.033709355	0.838838466	2.8810574	down	protein_coding	NM_001119	ADD1
ASHGV40057245	0.008740225	0.838838466	2.9435385	down	protein_coding	NM_005284	GPR6
ASHGV40017825	0.025801574	0.838838466	2.2069083	down	protein_coding	NM_178863	KCTD13
ASHGV40023727	0.029507598	0.838838466	2.9230495	down	protein_coding	NM_001060	TBXA2R
ASHGV40024669	0.005829619	0.838838466	2.90951	down	protein_coding	NM_001114598	ASPDH
ASHGV40043404	0.016451093	0.838838466	2.7138759	down	protein_coding	NM_001010903	C6orf222
ASHGV40042451	0.023941549	0.838838466	2.1659864	down	protein_coding	NM_018915	PCDHGA2
ASHGV40022446	0.032033587	0.838838466	2.1365885	down	protein_coding	uc009vno.2	LOC649330
ASHGV40055499	0.009819855	0.838838466	3.730226	down	protein_coding	NM_001171909	CXorf40A
ASHGV40027111	0.022571452	0.838838466	3.3778712	down	protein_coding	NM_016490	FAM178B
ASHGV40024625	0.007993464	0.838838466	3.0186891	down	protein_coding	NM_033377	CGB1
ASHGV40011044	0.009571621	0.838838466	2.0659563	down	protein_coding	NM_001291823	KLRF1
ASHGV40006446	0.000869774	0.838838466	2.7412597	down	protein_coding	uc001lnh.1	LOC619207
ASHGV40057643	0.01868029	0.838838466	2.205348	down	protein_coding	NM_012084	GLUD2
ASHGV40022292	0.031973109	0.838838466	3.080581	down	protein_coding	NM_152468	TMC8
ASHGV40042599	0.015888787	0.838838466	3.0672277	down	protein_coding	NM_001099293	KIF4B
ASHGV40007945	0.037940705	0.838838466	3.1671157	down	protein_coding	NM_001001480	KRTAP5-5
ASHGV40007383	0.018601177	0.838838466	2.2649234	down	protein_coding	NM_182833	GDPD4
ASHGV40026150	0.005904072	0.838838466	2.4876256	down	protein_coding	NM_017652	ZNF586
ASHGV40054594	0.012094168	0.838838466	9.0955435	down	protein_coding	NM_172377	CTAG2
ASHGV40001636	0.036109535	0.838838466	2.9706963	down	protein_coding	NM_016340	RAPGEF6
ASHGV40030143	0.040237232	0.838838466	2.6387709	down	protein_coding	NM_003936	CDK5R2
ASHGV40025803	0.003634045	0.838838466	2.8863637	down	protein_coding	NM_212550	BLOC1S3
ASHGV40048303	0.039311789	0.838838466	2.3146498	down	protein_coding	NM_005295	GPR22
ASHGV40034785	0.015451339	0.838838466	2.1356929	down	protein_coding	uc011biu.1	EU250752
ASHGV40043346	0.011345242	0.838838466	2.5882547	down	protein_coding	NM_001163771	COL11A2
ASHGV40006591	0.006228521	0.838838466	2.3346231	down	protein_coding	uc021qcy.1	OLFR690

ASHGV40007145	0.011566094	0.838838466	3.5920809	down	protein_coding	uc009ypx.3	AB429224
ASHGV40006931	0.019765522	0.838838466	2.0069666	down	protein_coding	NM_005055	RAPSN
ASHGV40018967	0.017592503	0.838838466	2.292998	down	protein_coding	NM_003041	SLC5A2
ASHGV40032570	0.002121343	0.838838466	3.7286711	down	protein_coding	NM_181605	KRTAP6-3
ASHGV40024131	0.01543495	0.838838466	2.2432449	down	protein_coding	NM_002744	PRKCZ
ASHGV40057386	0.013929624	0.838838466	3.6046761	down	protein_coding	uc022anf.1	TCRBV6S6A2T
ASHGV40005908	0.041214731	0.838838466	2.8031504	down	protein_coding	NM_018999	CCSER2
ASHGV40005532	0.001897275	0.838838466	5.8673895	down	protein_coding	NM_052997	ANKRD30A
ASHGV40017447	0.010553248	0.838838466	2.4655438	down	protein_coding	NM_012467	TPSG1
ASHGV40002661	0.047954348	0.838838466	2.701569	down	protein_coding	ENST00000598644	PCDP1
ASHGV40010935	0.0017702	0.838838466	3.3472383	down	protein_coding	NM_001130862	RAD51AP1
ASHGV40000569	0.00405414	0.838838466	4.3190712	down	protein_coding	NM_020787	ZNF624
ASHGV40005942	0.002859914	0.838838466	3.8034847	down	protein_coding	NM_004670	PAPSS2
ASHGV40025949	0.024844113	0.838838466	2.0062086	down	protein_coding	NM_001648	KLK3
ASHGV40016942	0.04341536	0.838838466	3.681726	down	protein_coding	NM_178859	SLC51B
ASHGV40000172	0.049409761	0.838838466	2.1358103	down	protein_coding	ENST00000378590	C1orf222
ASHGV40011462	0.004788202	0.838838466	2.8461625	down	protein_coding	NM_022658	HOXC8
ASHGV40014141	0.008228432	0.838838466	3.0758985	down	protein_coding	NM_182578	THEM5
ASHGV40012645	0.010849174	0.838838466	3.1122677	down	protein_coding	NM_022843	PCDH20
ASHGV40042228	0.019223281	0.838838466	3.2085414	down	protein_coding	NM_177478	FTMT
ASHGV40041951	0.048571252	0.838838466	3.9125788	down	protein_coding	NM_001195190	LOC730159
ASHGV40047591	0.04096518	0.838838466	2.0785824	down	protein_coding	NM_003112	SP4
ASHGV40025882	0.010852729	0.838838466	2.9815992	down	protein_coding	NM_006184	NUCB1
ASHGV40021219	0.014364087	0.838838466	3.7801526	down	protein_coding	NM_000747	CHRNB1
ASHGV40040558	0.017676099	0.838838466	2.2249397	down	protein_coding	NM_032042	FAM172A
ASHGV40016612	0.017711757	0.838838466	2.7744611	down	protein_coding	NM_175741	NUTM1
ASHGV40049105	0.02884388	0.838838466	4.3501747	down	protein_coding	NM_001001957	OR2W3
ASHGV40019331	0.025912734	0.838838466	3.181104	down	protein_coding	uc002eyu.3	AK125701
ASHGV40028318	0.006214718	0.838838466	2.5164245	down	protein_coding	NM_016552	ANKMY1
ASHGV40016668	9.1836E-05	0.80062633	2.9265746	down	protein_coding	NM_001145643	PHGR1
ASHGV40043573	0.015655948	0.838838466	3.5072935	down	protein_coding	NM_003296	CRISP2
ASHGV40022314	0.032628626	0.838838466	2.7224545	down	protein_coding	ENST00000328313	ENPP7
ASHGV40048535	0.009586489	0.838838466	2.4558359	down	protein_coding	NM_144625	WDR64
ASHGV40007857	0.00294419	0.838838466	2.4955215	down	protein_coding	NM_002545	OPCML
ASHGV40011066	0.013083824	0.838838466	2.3977891	down	protein_coding	NM_001987	ETV6
ASHGV40024109	0.009016604	0.838838466	4.1867555	down	protein_coding	NM_001001524	TM6SF2
ASHGV40018450	0.002382855	0.838838466	3.7818914	down	protein_coding	NM_002768	CHMP1A
ASHGV40057285	0.046555021	0.838838466	2.4725533	down	protein_coding	ENST00000403226	AC091801.1
ASHGV40031834	0.036202953	0.838838466	2.0482696	down	protein_coding	NM_002591	PCK1
ASHGV40019740	0.004775604	0.838838466	2.3476742	down	protein_coding	NM_001167986	INCA1
ASHGV40023759	0.003373352	0.838838466	3.8218909	down	protein_coding	NM_139159	DPP9
ASHGV40001601	0.02406217	0.838838466	2.5303108	down	protein_coding	ENST00000511936	RP11-661C8.3
ASHGV40032633	0.035374965	0.838838466	2.0928063	down	protein_coding	ENST00000600312	AP000688.1
ASHGV40018557	0.025544329	0.838838466	3.0201026	down	protein_coding	NM_001099456	NPW
ASHGV40057612	0.011434177	0.838838466	2.1933688	down	protein_coding	NM_130388	ASB12
ASHGV40057648	0.016825999	0.838838466	2.3128637	down	protein_coding	ENST00000448053	CT45A6
ASHGV40021199	0.028024931	0.838838466	2.4680766	down	protein_coding	NM_001004333	RNASEK

Table S4. The relationship between CRIP1 expression and clinicopathological characteristics

	CRIP1		
	low expression	high expression	P value[a]
**Gender**			0.429
male	27	194	
female	13	70	
**Age**			0.384
≤64	24	173	
> 64	17	91	
**Borrmann**			0.003
I	2	2	
II	6	12	
III	24	219	
IV	3	21	
**Differentiation**			0.914
well	3	20	
moderately	10	56	
poorly	28	185	
**T stage**			<0.001
T1	5	0	
T2	12	12	
T3	14	50	
T4	10	202	
**N stage**			<0.001
N0	31	58	
N1	4	58	
N2	2	60	
N3	4	88	
**M stage**			0.229
M0	41	250	
M1	0	14	
**TNM stage**			<0.001
I	15	4	
II	17	70	
III	9	176	
IV	0	14	
**Lymphatic metastasis**			<0.001
Yes	31	58	
No	10	206	

Note: a, p value less than 0.05 regarded as statistically significant.

Table S5 Abundance of protein in immunocomplex immunoprecipitated by CRIP1 antibody

gene	Accessions	MW [kDa]	Unique Peptides _CRIP1	Unique Peptides _igG	Score _CRIP1	Score _igG	Abundances (Normalized) _CRIP1	Abundances Normalized _igG
UCHL1	D6R956	26.8	1	-	0	-	10660183	-
SMARCA5	O60264	121.8	7	-	9.35	-	17525308.25	-
CLTA	F8WF69	27.8	2	-	2.26	-	7955533.5	-
FKBP10	Q96AY3	64.2	2	-	4.37	-	4122122	-
PAK1IP1	Q9NWT1	43.9	1	-	2.6	-	2263648.25	-
SF3B2	Q13435	100.2	12	2	16.17	1.88	34995544.63	
-	B4DNB9	52.3	5	-	6.62	-	22352410.25	-
ATAD3B	Q5T9A4	72.5	1	-	9.86	-	14473301	-
LRCH1	Q9Y2L9	80.8	1	2	4.92	5.09	9131237	
ZNF622	Q969S3	54.2	2	-	2.85	-	8389213	-
-	Q59EH3	18.7	1	3	2.06	3.33	684632.5	
ZNF326	Q5BKZ1	65.6	2	-	3.79	-	7900970	-
LSM7	Q9UK45	11.6	1	-	1.74	-	3031527	-
TRA2A	Q13595	32.7	3	2	7.36	2.05	4701993.75	
DHX36	Q9H2U1	114.7	3	-	4.64	-	2859006.375	-
KPNA1	P52294	60.2	5	-	10.67	-	16977499.75	-
IPO5	H0Y8C6	123.8	2	2	2.34	3.89	2903972	
-	Q53F62	44.6	1	-	1.85	-	2590854	-
SMC1L1	A0A384MR33	143.1	4	-	4.02	-	7571849.25	-
WDR18	Q9BV38	47.4	1	-	0	-	725641.9375	-
RPL10	P27635	24.6	6	-	11.6	-	142545092	-
PURB	Q96QR8	33.2	1	-	2.47	-	11784199	-
-	B2RDP6	49.4	1	-	2.73	-	3220602.25	-
EIF4G1	Q4LE58	178	8	3	13.21	3.76	10853558.75	
MRPS34	C9JJ19	26.3	3	-	5.36	-	13148031.25	-
THRAP3	Q9Y2W1	108.6	16	-	37.1	-	238035514.5	-
TARS2	Q9BW92	81	1	-	0	-	7844112.5	-
RHOF	Q9HBH0	23.6	3	-	1.83	-	18425559.5	-
TCF25	Q9BQ70	76.6	1	-	2.28	-	1019542.688	-
-	B4E0L0	48.1	2	1	5.8	2.49	14921488	
CAT	P04040	59.7	6	2	10.96	2.45	6997356.75	
-	B2R7C7	57.8	3	-	1.64	-	9879532.813	-
-	A8K6Q4	128.5	5	-	4.71	-	7035505.375	-
NUFIP2	Q7Z417	76.1	8	-	23.48	-	44816625.5	-
GRSF1	Q12849	53.1	1	-	1.85	-	1627317.625	-
CDK11B	P21127	92.6	1	-	1.71	-	2386048.75	-
CRIP1	P50238	8.5	6	-	6.96	-	1389185758	-
MRE11	P49959	80.5	1	1	2.57	0	1824993.5	
RPL5	P46777	34.3	8	-	17.37	-	88415986	-
PLRG1	O43660	57.2	3	-	4.7	-	2703458.25	-
XRCC6	P12956	69.8	8	-	15.93	-	19451071.5	-
SRRT	Q9BXP5	100.6	6	-	7.4	-	24809717.75	-
PRMT1	Q99873	42.4	6	-	14.06	-	27359096.5	-
SNRPC	A0A0A0MRR7	19.7	1	1	3.45	3.44	10319533	
RBMXL1	Q96E39	42.1	1	-	10.77	-	6332439.5	-
EIF3A	Q14152	166.5	9	-	7.48	-	11132652.5	-
KHSRP	A0A087WTP3	73	6	-	13.42	-	16502376.5	-
-	Q53EU2	60	1	-	2.39	-	3579174.75	-
SQSTM1	Q13501	47.7	2	-	4.38	-	11177407	-
SNRPD2	P62316	13.5	4	2	8.87	5.22	18214041.5	
TIAL1	Q01085	41.6	4	-	9.32	-	44827934	-
NMD3	C9JA08	60.1	1	1	2.05	2.39	1926036.75	
PSME3	A0A024R203	30.9	7	-	11.54	-	22053946.25	-
-	A8K800	35.6	1	-	0	-	2907695	-
TMPRSS13	J3KQC6	63.1	1	-	0	-	569611.25	-
DNAJB6	A0A0J9YX62	36.6	2	2	2.94	2.31	4576298	
PTCD3	Q96EY7	78.5	3	-	5.05	-	11368357	-
PAIP1	Q9H074	53.5	2	-	4.19	-	9952793	-
MRM3	Q9HC36	47	2	-	2.13	-	1697561.5	-

ZCCHC8	Q6NZY4	78.5	4	-	4.91	-	1702265.75	-
KRT13	P13646	49.6	3	-	11.12	-	14626754	-
BCLAF1	E9PK91	100.3	1	-	40.86	-	8240813.5	-
-	A8K3Z5	34.8	2	-	2.78	-	27304689.5	-
ZFR	B2RNR6	116.9	3	-	5.85	-	6501287.5	-
MEF2D /DAZAP1	Q5IRN4	50.4	1	-	0	-	3877933.25	-
-	Q53HH4	52.1	11	-	23.28	-	160126892	-
TRAF4	A0A024QZ19	54.4	2	-	2.14	-	44903452.13	-
FLJ10292	A0A023T6R1	17.3	2	-	5.82	-	2649121.75	-
WDR12	Q53T99	47.7	3	-	8.41	-	2248183.75	-
TFAP2A	Q96SH1	48.3	3	-	1.61	-	7804239	-
NOP58	Q9Y2X3	59.5	5	-	9.64	-	11217767.5	-
PRDX3	P30048	27.7	2	-	2.39	-	6584015.5	-
PRIM2	P49643	58.8	1	-	0	-	4023172.25	-
SNRNP200	O75643	244.4	13	3	16.86	2.48	38843701.75	
HEL-S-275	V9HW55	30.2	1	1	0	1.89	2500490.5	
MRPL54	Q6P161	15.8	1	-	2.22	-	9734673	-
-	A0A1U9X9A1	140.4	2	-	4.59	-	1475178.125	-
PABPN1	Q86U42	32.7	2	-	0	-	64604872	-
-	A0A140VJT0	51.7	4	4	8.65	4.63	74641161	
FAM208A	Q9UK61	188.9	2	-	4.26	-	5954150.75	-
DGCR8	Q8WYQ5	86	2	-	3.65	-	3224784.5	-
SMTN	A0A087X1R1	108	1	1	1.82	2.16	2809596	
FIP1L1	Q6UN15	66.5	3	-	6.21	-	12210201.5	-
POTEF	A5A3E0	121.4	1	1	70.99	108.75	5175142.5	
ACAT1	P24752	45.2	2	1	3.07	3.55	4900356.5	
COPE	M0QXB4	36.9	1	1	0	0	3543351	
PES1	B2RDF2	68	3	-	2.36	-	2845925.75	-
DDX20	Q8IYV2	92.1	3	-	6.77	-	3518650.75	-
USP10	Q14694	87.1	6	1	9.15	0	21302337.5	
NUP93	H3BVG0	99.5	2	1	4	1.82	4863193	
XRN1	Q8IZH2	194	2	-	1.62	-	3144186	-
PACSIN3	D3DQR0	48.5	3	2	6.22	6.97	65249152.5	
-	Q53HB3	49.2	2	-	7.49	-	35147776.5	-
ZNF598	Q86UK7	98.6	4	1	7.21	1.69	25217308.5	
EBNA1BP2	H7C2Q8	40.7	4	2	2.17	3.09	26597661.75	
LTV1	Q96GA3	54.8	1	-	2.01	-	2532951.25	-
MYBBP1A	Q9BQG0	148.8	2	1	2.36	2.26	880600.0625	
-	B2RAQ8	88.3	6	2	10.04	0	6738164	
-	B2R983	27.5	1	-	0	-	1388547.875	-
ARPC1B	A4D275	40.9	6	-	15.48	-	53782606	-
DHX57	Q6P158	155.5	2	-	5.76	-	4267461	-
MRPS35	P82673	36.8	2	-	6.03	-	3832863	-
UTP3	Q9NQZ2	54.5	2	-	5.2	-	1653558.125	-
-	B2R791	77.5	1	-	2.37	-	9139294	-
-	B2RDG1	59.3	2	-	4.32	-	1535184.625	-
PRCP	A0A024R5L0	55.8	1	-	2.8	-	1827858.375	-
SFXN1	Q9H9B4	35.6	4	3	5.22	8.07	17416990	
STT3B	Q8TCJ2	93.6	5	1	2.49	0	4232509.5	
PHKB	Q93100	124.8	1	-	3.98	-	9587047	-
GOLGA2	Q08379	113	4	-	7.09	-	9326389	-
MCM4	A0A3B3IT92	100.7	1	-	3.05	-	3747509.25	-
-	B4DUQ1	48.5	1	-	59.01	-	1244656417	-
ARF1	A0A024R3Q3	20.7	4	-	10.65	-	42183074	-
PHKA2	P46019	138.3	7	-	14.22	-	19461784.5	-
PPP1CA	P62136	37.5	1	-	21.69	-	14173585	-
LUC7L2	A0A0A6YYJ8	54.2	3	-	5.03	-	9361889.25	-
ERH	P84090	12.3	4	1	11.26	1.75	89236320.06	
-	B4DL07	78.4	4	1	4.86	1.73	13155692.5	
NCBP1	Q09161	91.8	4	-	6.52	-	68203282.75	-
GCDH	A0A1L3A5U7	48.1	1	1	1.8	0	3933333.75	
RAB3C	Q96E17	25.9	1	-	4.38	-	4231116.5	-
-	A8K139	139.9	2	-	1.82	-	2674712	-
-	Q53G85	50.1	4	-	37.25	-	655957334	-

EIF3E	B2R806	52.2	4	5	8.1	4.17	5440121.5	
-	A8K946	57.7	2	1	2.03	2.22	3334587.75	
GIT2	Q14161	84.5	7	1	17.26	2.49	37878558.75	
FAM168A	Q92567	26.2	1	-	0	-	9340792	-
MRPS22	P82650	41.3	6	1	9.12	2.52	16371176	
-	Q59EK7	37.2	5	-	13.91	-	5272407.5	-
FHL1	Q13642	36.2	2	-	1.76	-	2875962	-
-	A8K964	81.5	7	1	10.34	2.37	13531613.75	
SBSN	Q6UWP8	60.5	2	2	4.3	2.01	633399.875	
APMAP	Q9HDC9	46.5	1	-	1.67	-	3235521.5	-
GEMIN5	Q8TEQ6	168.5	3	-	9.07	-	6549265.5	-
DDX39A	O00148	49.1	2	-	10.83	-	59889359	-
-	B2RB72	42.5	1	-	1.61	-	1876613.875	-
WDR77	A0A024R0H7	36.7	6	-	12.58	-	37959462	-
MRPL16	Q9NX20	28.4	2	1	5.77	0	7605036	
SEC61A1	B4DR61	52.9	2	-	0	-	7561890.5	-
EIF3D	O15371	63.9	2	-	4.67	-	19653751	-
ATP1B1	P05026	35	1	-	0	-	2766444	-
SPTBN1	A0A087WUZ3	274.7	18	-	47.17	-	48717491	-
IPO9	Q96P70	115.9	1	1	2.51	2.14	1205035.25	
PRC1	A0A024RC67	71.6	4	-	4	-	32603084.5	-
-	A8K6U7	95.6	14	-	41.33	-	145202495.2	-
FAM51A1	A0A024RBX2	28.6	1	-	2.27	-	2830880	-
MRTO4	Q9UKD2	27.5	4	-	6.77	-	18221783.75	-
PYM1	Q9BRP8	22.6	2	1	6.84	4.02	6005152	
NUP153	A0A024QZW7	153.9	3	3	1.8	2.13	5175576.5	
MRPS9	P82933	45.8	4	-	2.66	-	2368843.5	-
PSMD11	O00231	47.4	2	-	2.41	-	4222433.5	-
TECR	M0R2N5	39.9	4	-	5.31	-	36099846.25	-
NOL6	Q9H6R4	127.5	3	-	0	-	7541031.5	-
HDAC2	Q92769	55.3	2	-	3.52	-	5690306	-
RBM15	Q96T37	107.1	5	1	8.09	2.31	19616521.88	
ZGPAT	Q8N5A5	57.3	1	-	0	-	969972.375	-
NUP107	P57740	106.3	3	-	0	-	5259967.75	-
ALDH18A1	P54886	87.2	2	-	2.64	-	1410629.375	-
TOP3B	A0A024R1C2	96.6	2	-	4.6	-	6174596.75	-
-	B3KNJ3	52.5	1	1	0	3.38	2687929.75	
PSMC2	P35998	48.6	2	1	5.7	3.09	9788779.25	
HPSE	A0A024RDB8	61.1	1	-	0	-	5331419	-
PSMD3	O43242	60.9	3	-	5.81	-	6498938.25	-
NDUFA4	O00483	9.4	2	-	3.02	-	8836944.75	-
ASNS	P08243	64.3	3	-	0	-	4592861.5	-
PPHLN1	Q8NEY8	52.7	2	-	2.39	-	5641260.5	-
SF3B5	Q9BWJ5	10.1	1	-	1.61	-	3355726.75	-
RRS1	Q15050	41.2	2	-	0	-	16178333	-
CREB1	Q5U0J5	36.7	3	-	4.1	-	29922929	-
SAFB2	Q14151	107.4	1	-	2.44	-	5560981	-
PRMT5	O14744	72.6	7	-	15.04	-	247203757	-
DNTTIP2	Q5QJE6	84.4	1	-	2.91	-	1947040.25	-
CPEB4	D3DQM9	81.4	2	-	4.87	-	3313537.5	-
YTHDF3	A0A087WY31	64.5	1	-	12.98	-	3715717.75	-
EEF1E1	O43324	19.8	1	-	0	-	9890056	-
HACD3	H3BS72	47.1	1	-	2.27	-	6661505.5	-
RAB2B	Q8WUD1	24.2	1	-	1.96	-	5601793.5	-
GMPS	P49915	76.7	2	1	1.88	2.66	2677505.75	
NAA10	P41227	26.4	1	-	2.58	-	2771875	-
COIL	P38432	62.6	3	1	6.47	0	3294883	
RBM26	Q5T8P6	113.5	2	-	4.62	-	8101757.75	-
SUPT16H	Q9Y5B9	119.8	6	1	14.44	2.84	74422263	
LSM14A	Q8ND56	50.5	3	2	7.24	2.21	10927948	
-	Q59EC0	137.7	6	-	9.6	-	20382198.88	-
-	Q59EG8	100.5	4	1	4.19	1.66	5389183.5	
COX6C	A0A024R9B7	8.8	1	-	0	-	5304458	-
HLA-C	A0A1C3PHU3	41	1	-	1.68	-	3124874.25	-
EXOSC6	Q5RKV6	28.2	2	-	2.52	-	1180763.875	-

EIF3M	Q7L2H7	42.5	1	1	1.8	4.15	3078419.5	
EIF3S2	Q5U0F4	36.5	3	-	6.95	-	10886595.5	-
-	Q6ZP37	42.9	2	-	1.71	-	3791875.5	-
FN3K	Q9H479	35.1	1	-	2.49	-	933444.1875	-
CAMSAP2	Q08AD1	168	1	-	2.77	-	1908012	-
-	B2R6X2	74.7	4	-	4.24	-	2571263.375	-
LARP4B	Q92615	80.5	2	-	4.06	-	3485771.75	-
PRKACA	A0A024R7J0	40.6	4	-	7.89	-	132550565.3	-
-	A8K6V3	135.5	13	-	21.61	-	61108047.25	-
NFKB2	Q00653	96.7	1	-	3.8	-	13083653	-
-	Q59FC3	85.8	3	-	4.33	-	15717760.88	-
FARSA	K7ER00	62.4	3	1	2.1	3.08	4093483.5	
BCAS2	B2R7W3	26.1	1	-	1.84	-	1709615	-
RPS27L	H0YMV8	11.3	1	-	5.23	-	16284365.5	-
C12orf43	F5H7W8	31.7	1	-	0	-	2907979	-
AGO2	Q9UKV8	97.1	3	-	5.06	-	12639503	-
SRP68	Q9UHB9	70.7	1	-	2.42	-	4379055	-
MTA2	A0A024R534	75	2	-	4.32	-	2525164.5	-
CBR3	O75828	30.8	1	-	2.36	-	3015465.25	-
-	Q13344	53.3	3	4	16.47	8.41	435628616	
DHX37	Q8IY37	129.5	1	-	1.68	-	2614996.75	-
ATAD3A	H0Y2W2	64.3	6	-	15.11	-	164170088	-
-	B4DNE1	42.2	1	-	4.33	-	3271024.5	-
COPB1	P53618	107.1	3	-	4.3	-	1701043	-
GNL3L	Q9NVN8	65.5	1	-	2.07	-	4602595.5	-
CASKIN2	Q8WXE0	126.7	1	-	1.93	-	2574538.75	-
PLOD3	O60568	84.7	3	-	7.12	-	2955303.5	-
LARP4	Q71RC2	80.5	6	-	8.7	-	23771799	-
SNRPB2	P08579	25.5	4	-	3.7	-	7204838.25	-
YWHAZ	E7EX29	28	5	-	12.68	-	14124741.88	-
RAB35	Q15286	23	1	2	7.63	7.46	5400045.5	
SURF4	O15260	30.4	1	2	2.01	3.84	6442027.5	
CS	A0A024RB75	51.7	1	-	1.7	-	2666633	-
-	Q59FI9	21.5	5	-	11.45	-	53906737.75	-
MRPS2	A0A024R8D4	35.5	4	-	5.92	-	7494869.75	-
DKFZp686E2459	Q7Z3D7	110.3	2	-	5.41	-	5902107.5	-
BRI3BP	Q8WY22	27.8	1	-	2.25	-	1707396.625	-
-	A8K9K6	65.9	5	-	12.66	-	11903347	-
-	B3KQ33	118.7	1	-	2.13	-	679802.75	-
GLO1	Q04760	20.8	1	-	0	-	3068083.25	-
CDK5	Q00535	33.3	1	-	1.77	-	4678499.5	-
-	A8K4B4	49.4	2	-	4.18	-	3241336.5	-
SAMD1	Q6SPF0	56	2	-	4.8	-	14270207.5	-
DCD	P81605	11.3	1	-	0	-	52260012	-
PPP6C	O00743	35.1	2	-	4.65	-	3000419.5	-
-	B2RCM6	28.2	2	-	3.62	-	2544208.5	-
FMR1	X5D907	66.9	6	-	24.74	-	47762755.5	-
ZC3H11A	O75152	89.1	8	-	16.91	-	10862376	-
H1FX	Q92522	22.5	3	2	6.7	5.57	9310984	
DARS	P14868	57.1	5	1	9.7	1.82	4425882	
HNRNPM	P52272	77.5	10	-	52.91	-	420503764.4	-
-	A8K690	62.6	2	-	2.44	-	15055333.25	-
NUP98	P52948	197.5	2	1	4.03	2.74	3785462.25	
-	B2RDX7	59.7	1	-	1.82	-	6823075.5	-
ATXN10	Q9UBB4	53.5	1	4	1.88	6.61	1961054.75	
UBA1	P22314	117.8	15	7	36.27	15.89	62674467.5	
NRF	A3F768	77.5	3	-	4.26	-	12504304	-
-	A0A140VKF1	81.3	5	1	9.59	0	27994455.25	
SMN1	E7EQZ4	31.7	4	-	10.85	-	15639835	-
-	A0A140VKH3	71.6	5	3	9.41	5.52	12614271	
GIGYF2	I1E4Y6	152.4	6	-	8.33	-	155671103.6	-
CTPS2	Q9NRF8	65.6	1	1	4.91	4.67	2698293.25	
RAB5A	A0A024R2K1	23.6	1	2	8.67	8.7	7992977	
SSRP1	Q08945	81	7	1	12.34	1.83	25393532.75	

-	A8K088	46.1	8	-	23.87	-	31815768.75	-
RCC1L	Q96I51	50	2	-	0	-	4550966.25	-
SRPK2	A0A024R704	77.5	1	-	3.4	-	1515183.75	-
DNCL1	Q6FGH9	10.4	2	1	4.63	0	11150396.25	
hCG_ 30600	A0A024R8D5	20.1	1	2	2.28	2.43	11853117	
IMPDH	Q6ZNB1	57	11	-	23.49	-	134069932.5	-
PLOD1	A0A024R4H0	83.5	2	-	4.94	-	3670394.75	-
TMEM106	Q8N353	35.1	1	-	2.86	-	1373580	-
PUM2	Q8TB72	114.1	1	-	0	-	6332591	-
YTHDC1	J3QR07	85.5	1	1	2.49	1.6	687855.5	
CAD	P27708	242.8	3	-	4.18	-	1154389.25	-
RPS5	P46782	22.9	5	-	8.92	-	31379771.75	-
HEL-S-125m	V9HWP2	92.4	1	-	2.15	-	5328179	-
MRPS26	Q9BYN8	24.2	2	-	3.16	-	6284167.5	-
RSL1D1	O76021	54.9	4	1	5.25	2.64	7669710.5	
ABCD1	P33897	82.9	1	-	2.69	-	2839737	-
BANF1	A0A024R5H0	10.1	1	2	2.37	2.62	3512227.25	
BYSL	Q13895	49.6	2	2	2.57	3.88	1973641.75	
RPL36A	J3KQN4	16.4	1	-	0	-	3993750.75	-
COPG1	Q9Y678	97.7	5	2	2.65	4.86	20756064.75	
PTPN1	A8K3M3	49.9	2	1	1.87	1.66	11413131.25	
-	A8K6A2	47.8	4	-	3.97	-	10612904.5	-
-	B2R7P8	64.6	5	-	3.82	-	1789994.125	-
-	B3KUJ0	44.5	1	-	3.36	-	12868323	-
RPL22	P35268	14.8	1	-	2.15	-	62532384	-
EIF3CL	B5ME19	105.4	7	3	16.25	5.32	32731328	
TRIM56	Q9BRZ2	81.4	3	-	5.66	-	4452985.5	-
PCNA	P12004	28.8	4	-	9.59	-	13253289.5	-
PPP1R10	Q96QC0	99	1	-	1.72	-	3652274.75	-
-	A0A384MEF1	85.6	2	1	2.15	1.68	3406114.5	
TXLNG	Q9NUQ3	60.5	1	-	1.68	-	1927028	-
RICTOR	Q6R327	192.1	1	-	0	-	5483471.5	-
ILK	A0A0A0MTH3	54.6	5	4	6.12	2.39	24839647	
FAM98A	Q8NCA5	55.4	7	-	19.53	-	67031874.5	-
RAB11FIP5	A0A1B0GTL5	138.3	8	-	87.2	-	346705777.4	-
-	M0R2C6	65.7	1	-	2.6	-	3722704.25	-
NHP2	Q9NX24	17.2	1	-	0	-	5640372.5	-
-	S6B2A6	32.1	1	-	3.43	-	5706875	-
DRG1	Q9Y295	40.5	3	2	4.28	4.35	5694089.25	
LENG8	A0A087WUE4	95.5	2	-	3.83	-	2564406.5	-
-	B4DJ30	112.9	3	-	3.86	-	7157313.5	-
NDUFA10	E7ESZ7	44.7	2	2	2.75	4.12	11063281	
QKI	Q96PU8	37.6	1	-	2.18	-	2522525.5	-
-	B2RBA0	68.3	4	-	6.97	-	4403991.5	-
ARHGEF7	A0A2R8YG42	97.1	15	-	36.4	-	95193899	-
ARPC4-TTLL3	F8WCF6	21	4	-	6.22	-	54444262	-
-	B3KM36	23.7	4	1	8.73	1.78	3646943.25	
C14orf166	Q549M8	28.1	6	5	13.76	13.67	43528279.5	
GDA	A0A024R231	51	2	-	3	-	5824859.75	-
YARS	P54577	59.1	2	1	3.92	1.66	1365718.5	
SLC27A2	O14975	70.3	2	1	2.67	1.77	1614022.875	
RRP9	O43818	51.8	1	-	1.64	-	80616232	-
SAP18	X6RAL5	19.5	2	-	2.55	-	3414994	-
ARL8B	Q9NVJ2	21.5	3	-	6.04	-	9173711.25	-
CALD1	A0A140VKA0	62.6	1	-	28.78	-	1880656.25	-
DKFZp686E1893	Q5HYL4	69.4	1	-	2.45	-	609824.1875	-
CASC3	A0A024R1X0	80.4	1	-	0	-	1495365.375	-
DKC1	O60832	57.6	3	-	7.01	-	15117209.75	-
SNRPF	P62306	9.7	2	-	0	-	6170226	-
RANGAP1	A0A024R1U0	63.5	2	-	3.77	-	6611494	-
CYC1	P08574	35.4	3	1	4.93	1.72	3780131.25	

EIF4E	D6RBW1	28.5	3	1	5.71	0	7623119.75	
KIF22	Q14807	73.2	3	-	6	-	18054930.75	-
RANBP2	P49792	358	4	-	4.45	-	14080231.5	-
RIOK1	Q9BRS2	65.5	1	-	3.5	-	4155944.25	-
TBL3	Q12788	89	3	-	8.35	-	7845743	-
DDX46	A0A0C4DG89	117.4	1	1	1.79	0	4410980	
HIST1H2BJ	P06899	13.9	2	-	14.75	-	496077880	-
MATR3	A8MXP9	99.9	18	5	31.46	1.67	119041852.5	
-	Q53GS0	73.8	6	-	8.87	-	26133008.25	-
hCG_2026745	A0A024R9P1	34.1	2	1	5.16	0	15856297	
CHERP	J3QK89	104.9	4	-	11.68	-	32440219.5	-
NAT10	Q9H0A0	115.7	10	1	13.25	2.06	102473926.5	
RBM28	A0A024R753	85.7	3	-	4.21	-	2436976.75	-
MKI67	P46013	358.5	3	-	6.71	-	759326.4375	-
DEK	P35659	42.6	1	-	1.76	-	4555643.5	-
RPL19	P84098	23.5	3	1	6.15	2.27	47489722	
DCUN1D5	A0A024R3A2	27.5	1	-	1.64	-	2719034.5	-
PRKAR1A	P10644	43	5	-	8.49	-	60496339.5	-
TFAM	E5KSU5	29.1	1	-	0	-	13154076	-
TJP2	A0A2R8YDH4	147.8	1	-	35.22	-	86348686	-
RAE1	P78406	40.9	3	-	2.08	-	15823104	-
DROSHA	Q9NRR4	159.2	15	-	33.06	-	27341104.5	-
SDCBP	G5EA09	34.8	2	-	0	-	19891108.25	-
NUP37	Q8NFH4	36.7	1	-	0	-	12965625	-
TPM4	K7ENT6	28.5	3	-	13.4	-	12883124	-
CBX3	A4D177	20.8	2	2	2.7	5.84	6338785.25	
EXOSC1	Q9Y3B2	21.4	1	-	2.36	-	12342772	-
MOV10	Q9HCE1	113.6	10	-	25.26	-	38669062.25	-
PYCR3	A0A0A0MQS1	29.9	2	-	2.31	-	10765123	-
RRP1B	Q14684	84.4	1	-	2.42	-	4135275.75	-
GTF3C2	Q8WUA4	100.6	1	-	2.27	-	2988635	-
-	A8KAP3	109.4	14	2	27.31	1.74	30599103.88	
-	Q59GW7	39.6	2	-	4.79	-	9145125	-
FYTTD1	Q96QD9	35.8	3	-	6.75	-	4405910.25	-
-	B2RAR2	46.6	2	-	2.29	-	8498739.25	-
PARN	O95453	73.4	2	-	1.8	-	4613558.5	-
AKAP8	O43823	76.1	6	-	11.58	-	16547726	-
RPTOR	Q8N122	148.9	1	-	2.17	-	431747.4375	-
BAG3	O95817	61.6	1	-	0	-	2742178	-
RAB18	Q9NP72	23	1	-	0	-	3048829	-
TCERG1	O14776	123.8	3	-	4.61	-	2583603.25	-
RFC2	B5BUD2	39.2	2	-	4.39	-	18304504	-
AARS	P49588	106.7	2	1	4.49	1.69	3765567.25	
BAIAP2L1	Q9UHR4	56.8	1	-	2.94	-	2831535.5	-
-	B4DRS6	36.3	4	-	5.8	-	13636163.5	-
C19orf70	A0A140TA86	15.4	1	-	0	-	3654242.5	-
ATXN2	Q99700	140.2	4	-	9.7	-	5754519	-
-	A0A140VJS3	45.6	2	-	7.43	-	3578765	-
-	I1SRC5	34	1	-	2.32	-	5802232	-
-	A8K588	39.5	7	1	7.83	2.38	130821171.4	
CTSD	P07339	44.5	2	-	2.35	-	2367586.25	-
THUMPD3	Q9BV44	57	1	-	2.25	-	2178092.5	-
NUP205	Q92621	227.8	3	-	3.25	-	6543528.25	-
NOP2	P46087	89.2	4	-	6.32	-	32604736	-
LOC392896	A4D2F6	60.6	1	-	1.85	-	23127024	-
NCOA5	Q9HCD5	65.5	1	-	0	-	3087465	-
EIF2B4	E7ERK9	59.7	1	-	2.28	-	1380881	-
-	A8K787	33.1	3	-	13.32	-	3475951.5	-
RPS15	K7ELC2	17.7	2	1	2.42	3.77	23898292.63	
EXOSC9	A5PLM5	48.9	2	-	2.52	-	5404962.5	-
-	B3KN49	40.6	2	-	5.75	-	4852960	-
TRAFD1	O14545	64.8	1	-	2.59	-	1867295.5	-
-	E5KT65	24.5	2	-	3.83	-	5881229	-
PRPF40A	A0A3F2YNY6	112.3	4	1	3.96	1.88	4841722.5	

MAGT1	A0A087WU53	41.5	3	1	5.79	2.54	1148845973	
IPO7	O95373	119.4	6	2	10.1	5.99	17511718.5	
YWHAE/FAM22A	G9K388	41.2	4	-	7.34	-	32501130.75	-
U2AF1	B5BU08	27.9	3	1	5.38	0	11970304.5	
MTERF3	Q96E29	47.9	2	-	0	-	5273412.5	-
-	B2RCM2	134.4	3	-	7.9	-	3423731.25	-
YWHAB	B5BU24	28.1	2	-	9.66	-	9812809	-
GAR1	Q9NY12	22.3	1	1	2.67	2.2	7207815	
-	A8K492	101.1	6	2	6.93	1.95	61812930	
-	Q59HG1	140.3	1	-	0	-	2264682.25	-
C8orf33	A0A3B3IT54	25.6	2	-	5.7	-	8763077	-
HNRNPC	G3V4C1	32.2	9	-	21.24	-	201994843	-
SNRP70	A0A024QZD5	51.5	4	1	6.34	1.99	4987552.5	
ZCCHC9	A0A024RAL5	30.5	1	-	2.67	-	1632564.5	-
CKAP5	Q14008	225.4	4	-	3.88	-	6133034.25	-
HLA-A	A0A0S4XRJ3	40.9	2	-	3.83	-	14544472.5	-
S100A7	P31151	11.5	1	-	0	-	6726910.5	-
ALKBH5	Q6P6C2	44.2	1	-	2.19	-	3694507.25	-
U2SURP	O15042	118.2	7	-	11.1	-	18080003.75	-
-	A0A384NY64	19.8	2	2	26.86	25.26	6520151.5	
-	B7Z481	51.2	1	-	5.18	-	1730091.25	-
-	Q53G19	20.6	4	-	9.98	-	20060216	-
H2AFY	O75367	39.6	3	-	7.08	-	21666684.5	-
LMO7	Q8WWI1	192.6	33	-	85.52	-	409216948.8	-
ATXN2L	Q8WWM7	113.3	10	2	17.73	2.98	54755597	
RPS26	A0A024RB14	13	2	-	2.93	-	33964456	-
-	A8K8K1	39.3	1	-	2.42	-	2501535.25	-
DKFZp667O1614	Q5JPC1	35.5	1	1	1.64	0	1485888	
PIPSL	A2A3N6	95	2	-	2.9	-	2996340	-
SNU13	A0A3B3IUA2	30.1	1	-	2.52	-	19530060	-
HAUS6	Q7Z4H7	108.6	1	-	2.51	-	2632522.75	-
EPPK1	P58107	555.3	2	1	5.24	5.64	2032325.625	
-	Q53GF9	25.6	1	-	0	-	1373967.125	-
OSTC	Q9NRP0	16.8	1	1	2.38	2.82	1538306	
SERBP1	Q8NC51	44.9	15	-	26.1	-	185946150.5	-
FXR1	A0A0F7KYT8	76.2	15	1	47.94	0	163135795.5	
-	B3KML1	58.4	2	-	2.31	-	1569643.375	-
LIMCH1	Q9UPQ0	121.8	14	-	31.27	-	52441050	-
SCCPDH	Q8NBX0	47.1	2	-	0	-	8031908	-
LSM12	K7ELG9	24.9	2	-	1.89	-	25641813	-
LMNB1	P20700	66.4	6	1	9.13	5.52	15540870.38	
ATP6	A0A023I889	24.8	1	1	2.93	2.24	2643280.5	
LRRC47	Q8N1G4	63.4	3	1	8.44	2.34	3020287.25	
-	B4DTK7	108.9	6	-	12.12	-	109206492.9	-
PCYOX1	Q9UHG3	56.6	5	3	6.08	6.12	11394865.5	
CDC27	P30260	91.8	2	-	5.2	-	4049828.75	-
hCG_18987	A0A384MDS4	49.8	5	1	8.71	2.22	46967512.25	
MSH6	P52701	152.7	10	1	19.38	2.83	29338887.25	
PRPF6	O94906	106.9	12	-	28.72	-	36529533.31	-
KPNA3	O00505	57.8	2	1	3.88	0	1980509.375	
CDKN2AIP	Q9NXV6	61.1	4	1	6.58	3.12	5426649.75	
EIF4E2	O60573	28.3	5	2	14.59	3.13	13590779.88	
-	A8K9U0	40.3	1	-	12.96	-	31134726	-
MFAP1	P55081	51.9	5	-	9.11	-	29318004.88	-
-	A8K2W7	55.7	2	-	2.49	-	4392244.5	-
-	E9PLN8	17.9	1	-	2.08	-	4283447.5	-
PRPF8	Q6P2Q9	273.4	14	1	16.1	1.74	4375797.125	
PHKG2	J3KNN3	46.9	2	-	2.08	-	4193969.5	-
SLC25A10	F6RGN5	43.6	3	-	3.72	-	33295572	-
CHTOP	Q9Y3Y2	26.4	5	1	13.01	3.57	59072859	
LDHB	P07195	36.6	7	2	15.9	2.77	64658370.5	
-	Q59EL4	60	4	-	12.59	-	40440064.5	-
SRRM1	A0A0S2Z4Z6	103.9	2	-	4.85	-	1752028.25	-

TRIP13	Q15645	48.5	3	1	4.11	2.14	6437862.5	
EWSR1	Q01844	68.4	5	-	18.5	-	29458388.63	-
-	A0A140VK53	299.4	4	-	5.72	-	3169524.75	-
-	B4DT57	50	1	1	0	2.26	1900884.875	
RBM25	P49756	100.1	4	1	7.83	3.91	11164081	
POLR2B	P30876	133.8	5	-	11.07	-	1553295.875	-
-	Q53HB9	61.5	3	-	5.71	-	6422895	-
RBM27	Q9P2N5	118.6	4	-	9.73	-	30670348	-
DKFZp686 L20222	Q5H9N4	34.8	2	-	5.22	-	8401545	-
PSMC3	P17980	49.2	3	1	1.69	2.58	7349994.75	
TNPO1	Q92973	102.3	3	-	6.08	-	2060435.375	-
-	B4DEG7	65.4	2	1	5.02	2.65	10801525.5	
LOC102724 159	A0A0B4J2E5	102.4	2	-	2.81	-	2732846.25	-
SMU1	A0MNN4	57.5	4	1	9.77	2.88	20934553.5	
NCBP2	P52298	18	1	-	0	-	3842167.75	-
YTHDF1	Q9BYJ9	60.8	2	-	17.55	-	17701879	-
RPL37A	P61513	10.3	2	1	4.38	3.26	5153323.5	
-	B3KY60	92.2	6	-	12.67	-	10257795.75	-
-	A8K7N0	23.6	1	-	6.7	-	37823259	-
-	A0A384NQ03	59.3	4	-	10.63	-	11511273	-
MRPL22	J3KQY1	26.5	2	-	2.45	-	1633205.875	-
-	Q53F37	22.4	1	-	4.01	-	3987651	-
APOBEC3B	B0QYD3	57.2	4	-	3.72	-	6113730	-
C10orf70	A0A024QZN7	12.2	1	-	2.27	-	4305315	-
GFPT1	Q06210	78.8	3	2	2.51	4.55	3967034.375	
SCAMP3	O14828	38.3	2	-	6.88	-	4994843.5	-
RNH1	P13489	49.9	4	2	7.59	5.63	12279994	
-	B3KMC9	108.5	5	1	11.75	0	15708735.5	
GARS	A0A090N8G0	77.5	9	-	18.69	-	61200193.75	-
-	B2R5Y4	65.6	5	1	10.9	0	24639517.25	
MPHOSPH 10	O00566	78.8	1	-	0	-	2308409.5	-
SNRPGP15	A8MWD9	8.5	1	-	3.31	-	1440531.5	-
SLAIN2	Q9P270	62.5	1	-	3.41	-	4171880	-
-	A8K335	36	1	-	2.34	-	2522232.25	-
MRPS7	J3QLS3	31.7	5	-	9.74	-	11265850	-
ATP1A1	P05023	112.8	2	-	2.19	-	3952636.25	-
WDR33	Q9C0J8	145.8	2	-	2.02	-	1242058.75	-
NFIB	Q5VW26	63.4	1	-	1.83	-	2931682	-
G3BP2	A0A024RDB2	50.8	11	-	21.37	-	225144614	-
LUC7L3	J3KPP4	58.2	2	-	2.92	-	8028746	-
DDX27	Q96GQ7	89.8	2	1	2.98	1.7	2410427.75	
CDC20	Q12834	54.7	1	-	0	-	1877050	-
SERPINB1	P30740	42.7	1	-	0	-	1741337.625	-
SNRPA1	P09661	28.4	3	2	5.18	2.21	11313527	
FASN	P49327	273.3	8	-	10.59	-	29991570	-
CEP170	Q5SW79	175.2	2	1	5.75	2.92	4564600	
EEF1B2	A0A024R3W7	24.7	2	2	5	3.89	4519961.5	
-	Q59G75	146.3	5	-	5.22	-	2442229.25	-
-	Q6U8A4	128.9	3	-	5.19	-	3056493	-
CSE1L	P55060	110.3	9	-	15.43	-	16897186	-
WDR3	Q9UNX4	106	3	-	4.55	-	8826965.5	-
-	B3KMR5	143.6	3	-	5.88	-	5041345.5	-
CPSF2	A0A024R6H0	88.4	2	-	0	-	4395752.375	-
RFC3	A0A024RDQ8	40.5	4	-	4.91	-	1353113.5	-
FGFR2-BICC1	V5YQU3	172.6	1	-	2.01	-	34542052	-
TRMT10C	Q7L0Y3	47.3	4	-	4.27	-	31001195.88	-
-	B2R6L0	49.9	1	1	73.54	68.19	7735514.5	
ARHGEF2	V9GYM8	116	9	3	20.93	4.45	22035841.25	
SNRPD3	P62318	13.9	3	2	5.1	5.08	33322369.5	
ZC3H14	Q6PJT7	82.8	1	-	2.68	-	3838365.5	-
ANAPC7	Q9UJX3	66.8	3	-	1.65	-	3838733.75	-

DDX23	A0A024R0Z3	95.5	10	-	8.14	-	18847113.75	-
hCG_23373	Q53YD8	20.9	1	-	2.53	-	2610443.75	-
SNX33	Q8WV41	65.2	4	6	2.98	4.11	8035942.5	
FXR2	P51116	74.2	8	-	28.41	-	54468461	-
CD2BP2	A0A024QZC1	37.6	1	-	0	-	10009150	-
CSRP2	F8VW96	26.7	1	-	2.88	-	3226012	-
OSBPL3	Q9H4L5	101.2	1	-	0	-	875018.875	-
RPL13A	M0QYS1	24.2	4	-	8.55	-	51791780	-
-	B4DJ38	84.1	5	2	5.45	2.4	9327304.688	
-	Q59GW5	72.2	8	1	16.38	2.15	28763562.75	
MTREX	P42285	117.7	11	-	10.22	-	41753093.75	-
LIMA1	F8VQE1	67	1	-	53.63	-	8000190.5	-
USP39	Q53GS9	65.3	2	-	1.81	-	11257040	-
CSDE1	A0A024R0E2	88.8	8	1	11.79	0	12142012.75	
EL52	K9JA46	84.6	4	1	30.49	16.54	14767669.75	
RBM4B	Q9BQ04	40.1	1	-	12.14	-	5526995.5	-
BCLAF1	Q9NYF8	106.1	1	-	43.5	-	192552982.4	-
SRSF10	O75494	31.3	4	1	7.44	2.24	46176365.5	
RAB14	P61106	23.9	4	2	13	4.5	26682713.5	
LBR	A0A024R3R5	70.7	4	-	3.93	-	5418038	-
TMCO1	J9JIE6	27.1	1	1	2.65	1.68	10258282	
FAM98B	Q52LJ0	37.2	1	2	2.89	6.17	2637869	
-	Q53GZ6	70.9	14	-	34.16	-	232722843.8	-
TARDBP	A0A024R4E2	44.7	1	3	0	7	11042558	

Table S6. Expression of Forty-five total cytokines and chemokines in the CRIP1 knockdown conditioned medium

Cytokine	Concentration（pg/ml）		P-value
	sh-NC	sh-CRIP1	
CD40 ligand	57.419	207.189	0.074
	0	57.419	
	0	133.657	
EGF	0	0	—
	0	0	
	0	0	
Eotaxin	18.543	16.342	0.441
	18.543	18.543	
	16.899	16.899	
FGF basic	5.363	16.812	0.862
	8.075	0	
	0	0	
Flt-3 ligand	33.063	30.397	0.089
	23.229	42.758	
	25.03	41.004	
Fractalkine	124.223	158.102	0.092
	91.293	135.42	
	135.42	181.134	
G-CSF	6.938	6.176	0.721
	5.394	5.394	
	5.394	6.938	
GM-CSF	10.291	8.648	0.373
	8.648	10.291	
	8.648	11.949	
Granzyme B	1.548	0	0.968
	1.214	0.223	
	0.551	3.228	
GROa	140.19	77.707	0.907
	55.434	92.58	
	57.266	93.181	
GROb	95.072	62.816	0.704
	53.491	77.416	
	51.998	78.684	
IFN-α	0.588	0.588	0.374
	0.588	0.982	
	0.588	0.588	
IFN-β	0	0	—
	0	0	
	0	0	
IFN-γ	0	0	—
	0	0	
	0	0	
IL-1α	7.87	7.113	0.418
	7.113	6.339	
	8.612	8.242	
IL-1β	0	0	0.374
	0.241	0	
	0	0	
IL-1rα	0.903	0	0.374
	0	0	
	0	0	

IL-2	3.788	3.788	0.116
	2.752	3.788	
	2.752	3.788	
IL-3	0	0	—
	0	0	
	0	0	
IL-4	0	0.18	0.101
	0.048	0.18	
	0.18	0.314	
IL-5	0	0	—
	0	0	
	0	0	
IL-6	1110.761	1108.465	0.065
	1029.776	1294.262	
	1016.195	1349.449	
IL-7	0.391	0.886	0.873
	1.389	0.637	
	0.391	0.391	
IL-8	502.979	462.481	0.092
	354.019	597.584	
	374.942	631.501	
IL-10	0	0	—
	0	0	
	0	0	
IL-12p70	11.406	14.044	0.024
	11.406	16.722	
	8.815	14.044	
IL-13	0	0	0.859
	26.295	16.909	
	16.909	33.312	
IL-15	2.032	2.032	0.244
	2.183	2.183	
	1.586	2.564	
IL-17A	1.379	1.835	0.719
	1.835	1.835	
	2.764	1.835	
IL-17E	0	0	—
	0	0	
	0	0	
IL-33	5.38	2.045	0.865
	2.045	3.739	
	1.164	2.045	
IP-10	0.351	0.561	0.169
	0.561	0.561	
	0.099	0.561	
CCL2	54.729	56.707	0.107
	54.604	67.054	
	56.338	74.98	
CCL3	10.846	8.979	0.289
	8.979	11.687	
	8.979	13.238	
CCL4	131.362	150.464	0.374
	150.464	150.464	
	150.464	150.464	
CCL20	37.097	34.268	0.103
	29.347	42.548	

	28.588	43.673	
CCL19	1.459	1.459	0.118
	0.963	1.459	
	0.447	1.942	
PDGF-AA	5945.327	6067.583	0.926
	7611.553	6819.741	
	7277.713	8184.41	
PDGF-AB/BB	67.358	65.748	0.96
	73.106	69.828	
	74.181	78.376	
PD-L1	18.082	26.232	0.248
	2.554	18.082	
	18.082	18.082	
CCL5	146.432	42.674	0.007
	206.904	80.175	
	206.904	80.175	
TGF-α	2.305	2.931	0.035
	1.676	2.931	
	1.676	4.175	
TNF-α	0	0	0.374
	0	0	
	0	0.441	
TRAIL	11.389	7.83	0.743
	6.96	11.389	
	9.595	10.489	
VEGF	920.081	983.637	0.606
	1047.421	1082.405	
	1117.963	1144.089	

Table S7. Primer sequences for real-time PCR.

Name	Sequence (5' - 3')
**CRIP1 Forward**	CAAGTGCAACAAGGAGGTGT
**CRIP1 Reverse**	TTCCCACATTTCTCGCACTT
**VEGFC Forward**	GAGGAGCAGTTACGGTCTGTG
**VEGFC Reverse**	TCCTTTCCTTAGCTGACACTTGT
**CCL5 Forward**	CCAGCAGTCGTCTTTGTCAC
**CCL5 Reverse**	CTCTGGGTTGGCACACACTT
**ACTB Forward**	GGGAAATCGTGCGTGACATTAAG
**ACTB Reverse**	TGTGTTGGCGTACAGGTCTTTG
**VEGFC-promoter -p1-Forward**	GCACTGCATCCTGAGAACTG
**VEGFC-promoter -p1-Reverse**	GCCCTGCTCAAAGTTTGGAA
**VEGFC-promoter -p2-Forward**	CCTGTGGGTGTTGGAAATGC
**VEGFC-promoter -p2-Reverse**	GGCAGACTGGGGTAGGATTT
**CCL5-promoter -p1-Forward**	GTTGTCCCCAAGAAAGCGG
**CCL5-promoter -p1-Reverse**	TCTCTCCCTCACTGCTCTCT
**CCL5-promoter -p2-Forward**	AATGAGTTGACACGCGCTAG
**CCL5-promoter -p2-Reverse**	TCCCATTTACTTGCTAAGCAGC

Table S8. Antibodies used in this study.

Name	Source	Identifier
**Ki67**	Cell Signaling Technology	9129
**LYVE1**	abcam	ab14917
**Mouse LYVE1**	angiobio	11-034
**Human LYVE1**	angiobio	11-032
**VEGFC**	abcam	ab9546
**VEGFD**	abcam	ab155288
**CREB1**	Santa cruz	sc-377154
**CREB1**	Cell Signaling Technology	9197
**p-CREB1(Ser133)**	Cell Signaling Technology	9198
**TNF-α**	Cell Signaling Technology	3707S
**CCL5**	abcam	ab189841
**CRIP1**	abcam	ab183029
**GST**	Cell Signaling Technology	2625S
**Flag**	SIGMA	F3165
**GAPDH**	SAB signalway	6711
**β-actin**	SAB signalway	21800
**Horseradish enzyme- labeled goat anti-rabbit IgG (H+L)**	ZSGB-BIO	ZB2301
**Horseradish enzyme- labeled goat anti-mouse IgG (H+L)**	ZSGB-BIO	ZB2305
**Biotin-labeled goat anti-rabbit IgG**	ZSGB-BIO	SP-9001
**Biotin-labeled goat anti-mouse IgG**	ZSGB-BIO	SP-9002
**Streptavidin-horseradish peroxidase conjugate working solution**	ZSGB-BIO	SP-9001/9002
**Goat anti-Rabbit IgG (H+L) Cross-Adsorbed Secondary Antibody, Alexa Fluor 488**	Invitrogen	A11008
**Goat anti-Mouse IgG (H+L) Cross-Adsorbed Secondary Antibody, Alexa Fluor 594**	Invitrogen	A11005
